# Supplementary material for: Electrochemical deoxygenative amination of stabilized alkyl radicals from activated alcohols
Source: Nat Commun. 2024 Jul 20;15:6116. doi: 10.1038/s41467-024-50596-3 (PMC11271281; doi:10.1038/s41467-024-50596-3)
Supplement: Supplementary file 1 — Supplementary Information [file 41467_2024_50596_MOESM1_ESM.pdf]

Supplementary Information

Electrochemical Deoxygenative Amination of Stabilized Alkyl Radicals from Activated Alcohols

Jia Xu, Yilin Liu, Qing Wang, Xiangzhang Tao, Shengyang Ni, Lei Yu, Yi Pan and Yi Wang*

[a] State Key Laboratory of Coordination Chemistry, Jiangsu Key Laboratory of Advanced Organic Materials, Collaborative Innovation Center of Advanced Microstructures, School of Chemistry and Chemical Engineering, Nanjing University, Nanjing 210023, China

Supplementary Methods 2

1. General Considerations..2
2. Graphical Guide for the set-up..3
3. General Procedure for Synthesis of the carbazates [1]..3
4. Optimization Tables 5
5.General Procedure..10
6. Electrochemical Continuous-Flow System...12
7. Mechanistic Investigations..14
8. Characterization Data for Products [2-6]...19
9. NMR Spectra 53

Supplementary References...132

Supplementary Methods

1. General Considerations

All commercial reagents were used without additional purification unless otherwise specified. Solvents were purified and dried according to standard methods prior to use. All reactions were run under argon, unless otherwise noted. All experiments were monitored by thin layer chromatography (TLC) using UV light as visualizing agent. TLC was performed on pre-coated silica gel plated. Column chromatography was performed using silica gel 60 (300-400 mesh). The instrument for electrolysis is dual display potentiostat (DJS-292B) (made in China). The anode electrode is carbon anode (10 mm×10 mm×0.3 mm) and the cathode electrode is platinum plate electrodes (10 mm×10 mm ×3 mm). ^{1}H NMR (400 MHz) and ^{13}C NMR (101 MHz) were measured on a Bruker AVANCE III-400 spectrometer. Chemical shifts are reported in ppm (δ) relative to internaltetramethylsilane (TMS, δ 0.0 ppm) or with the solvent reference relative to TMS employed as the internal standard. Data are reported as follows: chemical shift (multiplicity [singlet (s), doublet (d), triplet (t), quartet (q), broad (br) and multiplet (m)], coupling constants [Hz], integration). Melting points are uncorrected. Infrared spectra were obtained on an Agilent Cary 630 instrument on a diamond plate by way of technology Attenuated Total Reflection (ATR). HRMS were conducted on an Agilent 6540Q-TOF LC/MS equipped with an electrospray ionization (ESI) probe operating in positive ion mode.

2. Graphical Guide for the set-up

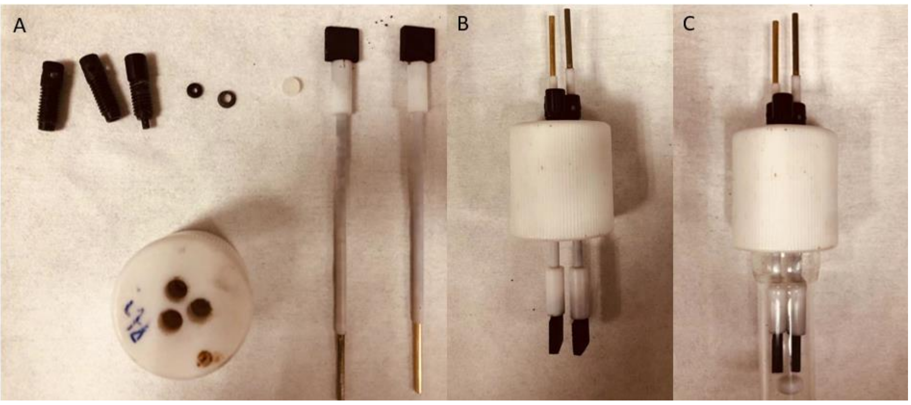

Supplementary Figure 1: A electrochemical reaction bottle

3. General Procedure for Synthesis of the carbazates [1]

General Procedure for Synthesis of the α-CF_3 carbazates (Procedure A)

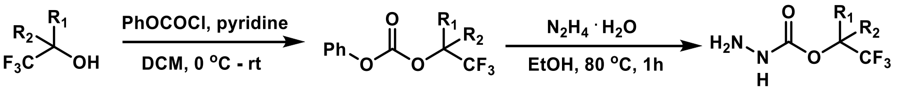

A round-bottom flask was charged with α-CF_3 alcohol (1.0 ml, 10.0 mmol, 1 equiv), followd by the addition of dichloromethane (10 ml) and pyridine (1.5 equiv), The solution was cooled to 0 °C, A solution of phenyl chloroformate (1.38 mL, 11 mmol, 1.1 equiv) in dichloromethane (10 ml) was added. then cooled to room temperature and allowed to stir for overnight. The reaction was quenched with 1 M hydrochloric acid. The aqueous layer was washed with methylene chloride, dried over Na_2SO_4 and concentrated in vacuo to afford the crude product carbonate. Next, hydrazine hydrate (2.0 equiv.) was added to the solution of the corresponding carbonate in EtOH (20 ml) and then stirred for about 1 h at 80 °C. Once complete, the reaction solvent was evaporated under reduced pressure (The product is sensitive to water). The corresponding carbazates was purified by silica gel column chromatography. (eluent: petroleum ether/ethyl acetate= 1:1).

General Procedure for Synthesis of the general carbazates (Procedure B)

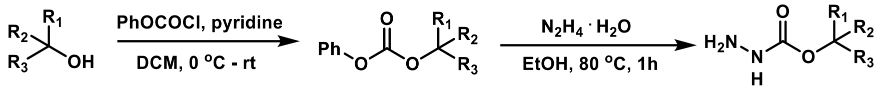

A round-bottom flask was charged with alcohol (1.0 ml, 10.0 mmol, 1 equiv), followd by the addition of dichloromethane (10 ml) and pyridine (1.5 equiv), The solution was cooled to 0 °C, A solution of phenyl chloroformate (1.38 mL, 11 mmol, 1.1 equiv) in dichloromethane (10 ml) was added. then cooled to room temperature and allowed to stir for overnight. The reaction was quenched with 1 M hydrochloric acid. The aqueous layer was washed with methylene chloride, dried over Na_2SO_4 and concentrated in vacuo to afford the crude product carbonate. Next, hydrazine hydrate (2.0 equiv.) was added to the solution of the corresponding carbonate in EtOH (20 ml) and then stirred for about 1 h at 80 °C. Once complete, the reaction was quenched with 1 M Sodium hydroxide solution. and extracted with EtOAc (50 mL × 3), the organic solvent was dried over Na_2SO_4. The solvent was evaporated under reduced pressure. The corresponding carbazates was purified by silica gel column chromatography. (eluent: petroleum ether/ethyl acetate= 1:1).

4. Optimization Tables

Supplementary Table 1. Optimization of Electrode

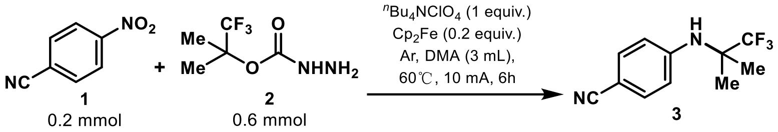

Entry	Electrode	Yield[a] (%)
1	C(+)/C(-)	84
2	C(+)/Fe(-)	37
3	C(+)/Cu(-)	41
4	C(+)/Pt(-)	13
5	Pt(+)/C(-)	NR

[a] GC yield. Reaction condition: **1** (0.2 mmol), **2** (0.6 mmol), ^{n}Bu4NClO4 (0.2 mmol), Cp2Fe (0.04 mmol), DMA (3 mL), under argon atmosphere, 60 °C, R = 1000 rpm, 10 mA, 6 h.

Supplementary Table 2. Optimization of Solvent

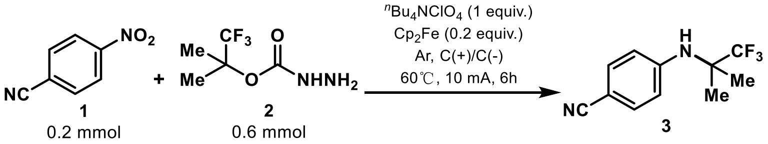

Entry	Solvent	Yield[a] (%)
1	DMA	84
2	DMF	72
3	MeCN	trace
4	DMSO	21
5	DCM	11
6	THF	24
7	acetone	NR

[a] GC yield. Reaction condition: **1** (0.2 mmol), **2** (0.6 mmol), ^{n}Bu4NClO4 (0.2 mmol), Cp2Fe (0.2 equiv.), C(+)/C(-), under argon atmosphere, 60 °C, R = 1000 rpm, 10 mA, 6 h.

Supplementary Table 3. Optimization of Electrolyte

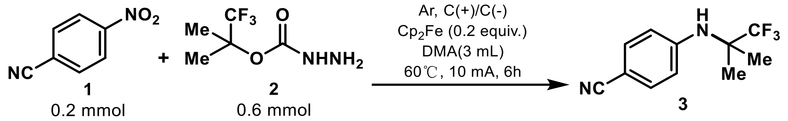

Entry	Electrolyte	Yield (%)
1	${}^{n}Bu_4NClO_4$	84
2	${}^{n}Bu_4NPF_6$	28
3	${}^{n}Bu_4NBF_4$	10
4	${}^{n}Bu_4NI$	53
5	${}^{n}Bu_4NBr$	38
6	$LiClO_4$	44

[a] GC yield. Reaction condition: **1** (0.2 mmol), **2** (0.6 mmol), Cp2Fe (0.2 equiv.), DMA (3 mL), C(+)/C(-), under argon atmosphere, 60 °C, R = 1000 rpm, 10 mA, 6 h.

Supplementary Table 4. Optimization of stirring speed

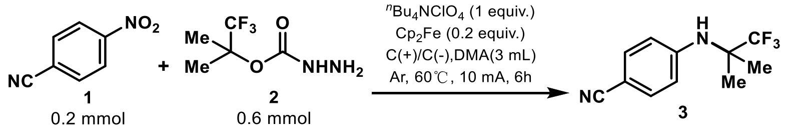

Entry	Stirring speed (rpm)	Yield (%)
1	0	trace
2	250	24
3	500	32
4	750	58
5	1000	84
6	1250	83

[a] GC yield. Reaction condition: **1** (0.2 mmol), **2** (0.6 mmol), ${}^{n}Bu_4NClO_4$ (0.2 mmol), Cp2Fe (0.2 equiv.), C(+)/C(-), DMA (3 mL) under argon atmosphere, 10 mA, 6 h.

Supplementary Table 5. Optimization of reaction time

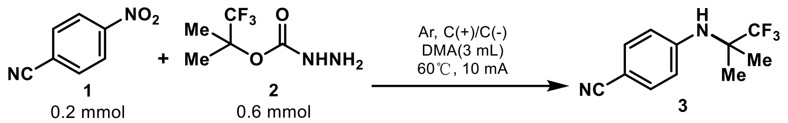

Entry	Time (h)	Yield (%)
1	2	47
2	4	77
3	6	84
4	8	71

[a] GC yield. Reaction condition: **1** (0.2 mmol), **2** (0.6 mmol), $^{n}Bu_4NClO_4$ (0.2 mmol), Cp_2Fe (0.2 equiv.), C(+)/C(-), DMA (3 mL) under argon atmosphere, R = 1000 rpm, 10 mA, 6 h.

Supplementary Table 6. Optimization of Additive

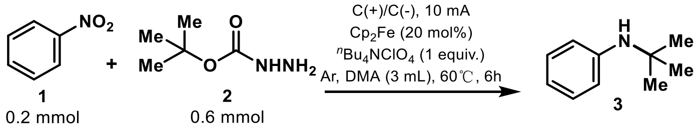

Entry	Additive	Yield(%)
1	Cp_2Fe (0.2 equiv.)	83
2	Cp_2Fe (0.5 equiv.)	83
3	Cp_2Fe (1 equiv.)	82
4	Cp_2Fe (2 equiv.)	74
5	Tempo (0.2 equiv.)	13
6	Tempo (0.5 equiv.)	23
7	Tempo (1 equiv.)	11
8	Tempo (2 equiv.)	NR

[a] GC yield. Reaction condition: **1** (0.2 mmol), **2** (0.6 mmol), $^{n}Bu_4NClO_4$ (0.2 mmol), C(+)/C(-), DMA (3 mL) under Ar, 60 °C, R = 1000 rpm, 10 mA, 6 h.

Supplementary Table 7. Optimization of Current

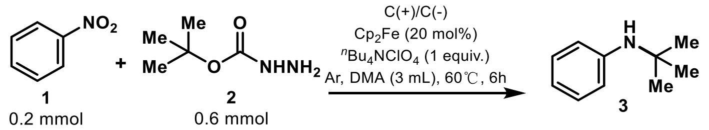

Entry	Current	Yield(%)
1	0 mA	NR
2	5 mA	24
3	10 mA	83
4	15 mA	77
5	20 mA	53

[a] GC yield. Reaction condition: **1** (0.2 mmol), **2** (0.6 mmol), $^{n}Bu_4NClO_4$ (0.2 mmol), Cp_2Fe (0.2 equiv.), C(+)/C(-), DMA (3 mL) under argon atmosphere, 60℃, R = 1000 rpm, 10 mA, 6 h.

Supplementary Table 8. Optimization of cyclohexyl hydrazinecarboxylate

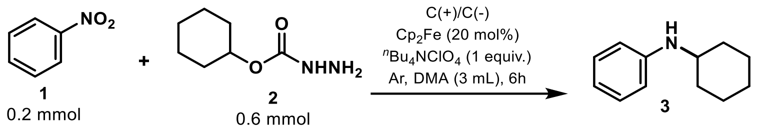

Entry	Temperature	Yield(%)
1	60 °C	39
2	70 °C	51
3	80 °C	58

[a] Isolated yield. Reaction condition: **1** (0.2 mmol), **2** (0.6 mmol), $^{n}Bu_4NClO_4$ (0.2 mmol), Cp_2Fe (0.2 equiv.), C(+)/C(-), DMA (3 mL) under argon atmosphere, R = 1000 rpm, 10 mA, 6 h.

Optimization of activated alcohol

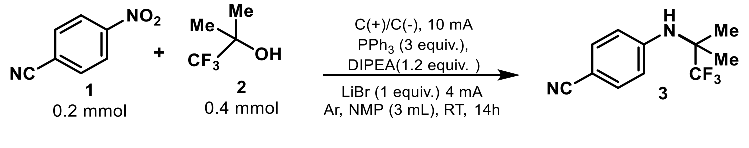

An undivided bottle was equipped with carbon anode and cathode (10 mm×10 mm×3

mm) connected to a DC regulated power supply. To the bottle was added nitrobenzene (26.2 mg, 0.2 mmol), 2-Trifluoromethyl-2-propanol (100.1 mg, 0.4 mmol), PPh_3 (156.3 mg, 0.6 mmol), DIPEA (31.1 mg, 0.24 mmol), LiBr (17.4 mg, 0.2 mmol) and 4 mL of NMP. The reaction mixture was stirred and electrolyzed at constant current conditions 4 mA at RT under argon atmosphere (The dual display potentiostat was operating in constant current mode) for 14 h. The reaction cannot proceed.

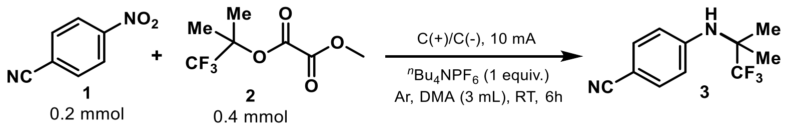

An undivided bottle was equipped with carbon anode and cathode (10 mm×10 mm×3 mm) connected to a DC regulated power supply. To the bottle was added nitrobenzene (26.2 mg, 0.2 mmol), oxalate (143.2 mg, 0.4 mmol), $^{n}Bu_4NPF_6$ (68.4 mg, 0.2 mmol) and 3 mL of DMA. The reaction mixture was stirred and electrolyzed at constant current conditions 10 mA at RT under argon atmosphere (The dual display potentiostat was operating in constant current mode) for 6 h. Only trace amount of the product was detected.

5.General Procedure

a. Procedure for Deoxygenative α-CF_3 Ammoniation

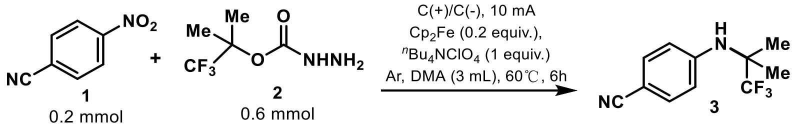

An undivided bottle was equipped with carbon anode and cathode (10 mm×10 mm×3 mm) connected to a DC regulated power supply. To the bottle was added nitrobenzene (26.2 mg, 0.2 mmol), α-CF_3 carbazate (111.6 mg, 0.6 mmol), Cp_2Fe (7.6 mg, 0.04 mmol), tetrabutylammonium perchlorate (68.3 mg, 0.2 mmol) and 3 mL of DMA. The reaction mixture was stirred and electrolyzed at constant current conditions 10 mA at 60 °C under argon atmosphere (The dual display potentiostat was operating in constant current mode) for 6 h. The reaction was quenched with aqueous $NaHCO_3$ and extracted with EtOAc (50 mL × 3), the organic solvent was dried over Na_2SO_4. The solvent was evaporated under reduced pressure. The crude product was purified by silica gel column chromatography. (eluent: hexane /ethyl acetate= 10:1)

b. Procedure for Deoxygenative α-CF_3 Ammoniation

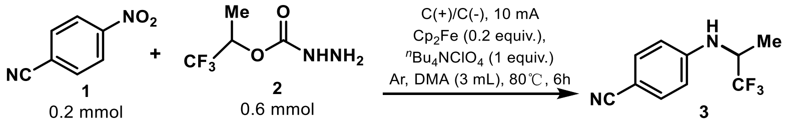

An undivided bottle was equipped with carbon anode and cathode (10 mm×10 mm×3 mm) connected to a DC regulated power supply. To the bottle was added nitrobenzene (26.2 mg, 0.2 mmol), α-CF_3 carbazate (98.6 mg, 0.6 mmol), Cp_2Fe (7.6 mg, 0.04 mmol), tetrabutylammonium perchlorate (68.3 mg, 0.2 mmol) and 3 mL of DMA. The reaction mixture was stirred and electrolyzed at constant current conditions 10 mA at 80 °C under argon atmosphere (The dual display potentiostat was operating in constant current mode) for 6 h. The reaction was quenched with aqueous $NaHCO_3$ and extracted with EtOAc (50 mL × 3), the organic solvent was dried over Na_2SO_4. The solvent was evaporated under reduced pressure. The crude product was purified by silica gel column

chromatography. (eluent: hexane /ethyl acetate= 10:1)

c. Procedure for Deoxygenative Tertiary Ammoniation

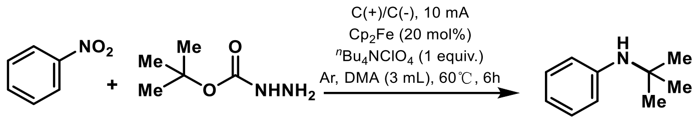

An undivided bottle was equipped with carbon anode and cathode (10 mm×10 mm×3 mm) connected to a DC regulated power supply. To the bottle was added nitrobenzene (26.2 mg, 0.2 mmol), tert-Butyl carbazate (79.3 mg, 0.6 mmol), Cp_2Fe (7.6 mg, 0.04 mmol), tetrabutylammonium perchlorate (68.3 mg, 0.2 mmol) and 3 mL of DMA. The reaction mixture was stirred and electrolyzed at constant current conditions 10 mA at 60 °C under argon atmosphere (The dual display potentiostat was operating in constant current mode) for 6 h. Once complete, the reaction was quenched with aqueous $NaHCO_3$ and extracted with EtOAc (50 mL × 3), the organic solvent was dried over Na_2SO_4. The solvent was evaporated under reduced pressure. The crude product was purified by silica gel column chromatography. (eluent: hexane /ethyl acetate= 10:1)

d. Procedure for Deoxygenative Primary and Secondary Ammoniation

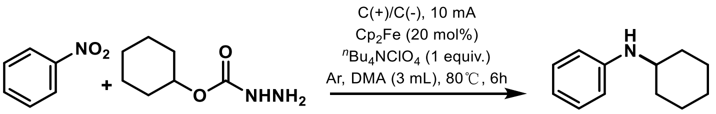

An undivided bottle was equipped with carbon anode and cathode (10 mm×10 mm×3 mm) connected to a DC regulated power supply. To the bottle was added nitrobenzene (26.2 mg, 0.2 mmol), cyclohexyl carbazate (94.8 mg, 0.6 mmol), Cp_2Fe (7.6 mg, 0.04 mmol), tetrabutylammonium perchlorate (68.3 mg, 0.2 mmol) and 3 mL of DMA. The reaction mixture was stirred and electrolyzed at constant current conditions 10 mA at 80 °C under argon atmosphere (The dual display potentiostat was operating in constant current mode) for 6 h. Once complete, the reaction was quenched with aqueous $NaHCO_3$ and extracted with EtOAc (50 mL × 3), the organic solvent was dried over Na_2SO_4. The solvent was evaporated under reduced pressure. The crude product was purified by silica gel column chromatography. (eluent : hexane /ethyl acetate= 10:1)

6. Electrochemical Continuous-Flow System

The continuous-flow system was purchased from the EZONE company. As shown in the Supplementary Figure 2, the reaction solution is added through an injection pump (Supplementary Figure 2, A). The solvent of the continuous-flow reactor is 3 mL, and the electrode spacing is 1 mm (Supplementary Figure 2, B). Temperature is controlled by heater and the current is controlled by the DC box (Supplementary Figure 2, D).

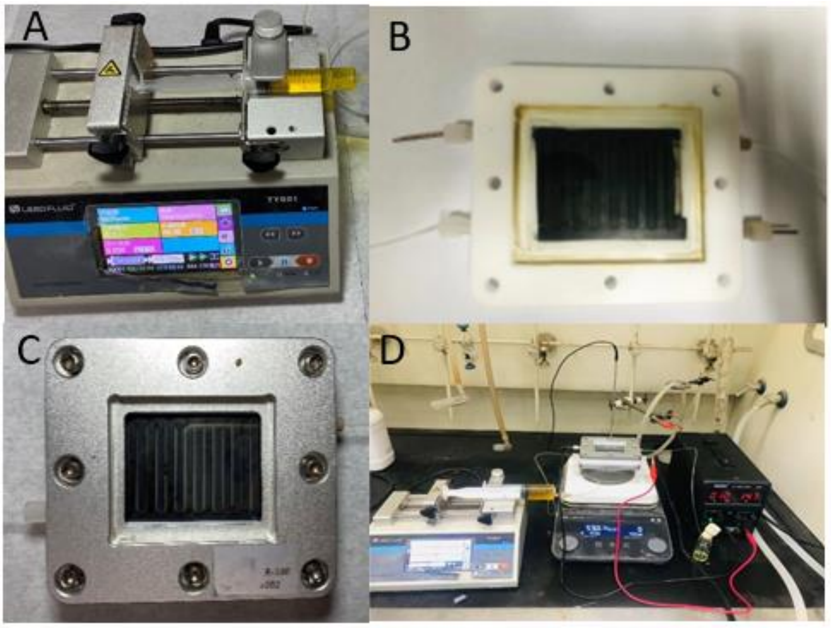

Supplementary Figure 2: Electrochemical Continuous-Flow System

A Scale-up Continuous Flow reaction

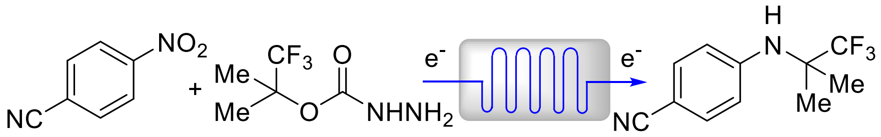

1 10 mmol 1.23 g | **2** 30 mmol 4.05 g | **60℃, 20 mA, 20 h** | **3, 71% 1.21g**

First, assembled and installed the flow electrochemistry device, the anode as graphite plate, cathode as graphite plate and the cell volume was 3 mL. Second, **1** (10 mmol, 1.23g), **2** (30 mmol, 4.05 g), Cp_2Fe (376 mg, 2 mmol) and $^nBu_4NClO_4$ (10 mmol, 3.84 g) were dissolved in DMA (30 mL). The reaction mixture was pumped into the flow cell via a syringe and electrolyzed at a constant current of 20 mA at 60 °C. The flow

rate was 0.025 mL/min and residence time was 2 h. The out flow of the reaction mixture was collected. The reaction was quenched with aqueous $NaHCO_3$ and extracted with EtOAc (50 mL × 3), the organic solvent was dried over Na_2SO_4. The solvent was evaporated under reduced pressure. The crude product was purified by silica gel column chromatography. (eluent: hexane /ethyl acetate= 10:1)

7. Mechanistic Investigations

a. Radical trap experiments:

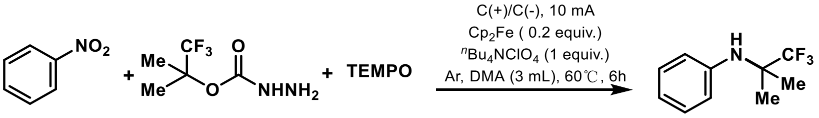

An undivided bottle was equipped with carbon anode and cathode (10 mm×10 mm×3 mm) connected to a DC regulated power supply. To the bottle was added nitrobenzene (26.2 mg, 0.2 mmol), α-CF_3 carbazate (111.6 mg, 0.6 mmol), Cp_2Fe (0.2 equiv.), tetrabutylammonium perchlorate (68.3 mg, 0.2 mmol), TEMPO (93.6 mg, 0.6 mmol) and 3 mL of DMA. The reaction mixture was stirred and electrolyzed at constant current conditions 10 mA at 60 °C under argon atmosphere (The dual display potentiostat was operating in constant current mode) for 6 h. The GCMS results indicate that the reaction cannot be occurred.

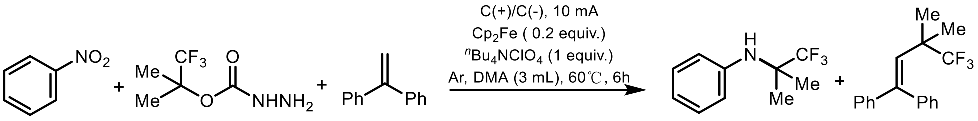

An undivided bottle was equipped with carbon anode and cathode (10 mm×10 mm×3 mm) connected to a DC regulated power supply. To the bottle was added nitrobenzene (26.2 mg, 0.2 mmol), α-CF_3 carbazate (111.6 mg, 0.6 mmol), Cp_2Fe (0.2 equiv.), tetrabutylammonium perchlorate (68.3 mg, 0.2 mmol), 1,1-diphenylethylene (108.2 mg, 0.6 mmol) and 3 mL DMA. The reaction mixture was stirred and electrolyzed at constant current conditions 10 mA at 60 °C under argon atmosphere (The dual display potentiostat was operating in constant current mode) for 6 h. The GCMS results indicate that the reaction cannot be occurred, and the alkyl radical intermediate was observed.

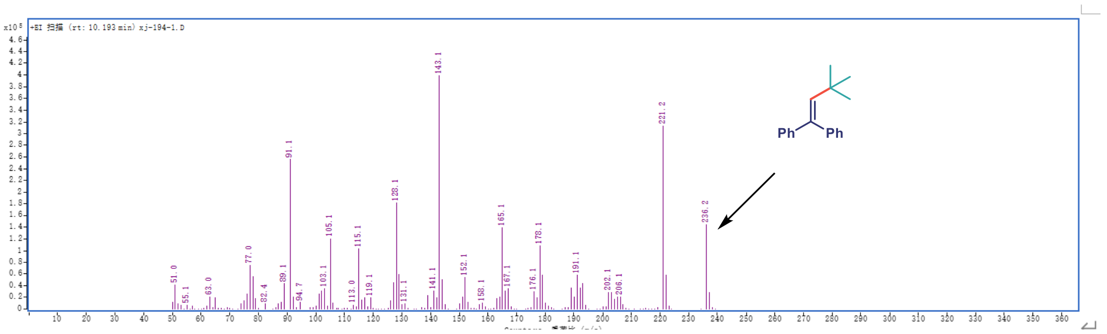

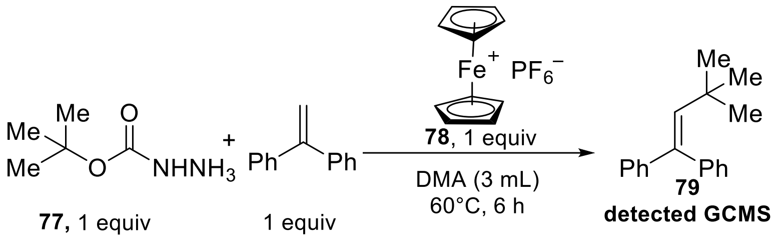

To the bottle was added tert-Butyl carbazate (27.8 mg, 0.2 mmol), $Fc^{+}PF_6^{-}$ (54.6 mg, 0.2 mmol), 1,1-diphenylethylene (108.2 mg, 0.6 mmol) and 3 mL DMA. The reaction mixture was stirred at 60 °C under argon atmosphere for 6 h. The GCMS results indicate that the reaction cannot be occurred, and the alkyl radical intermediate was observed.

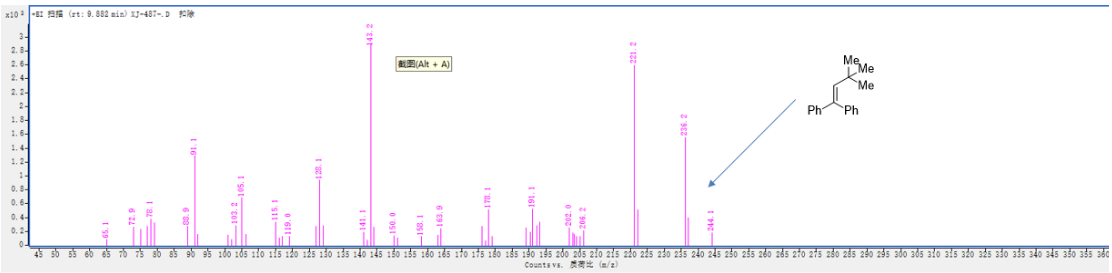

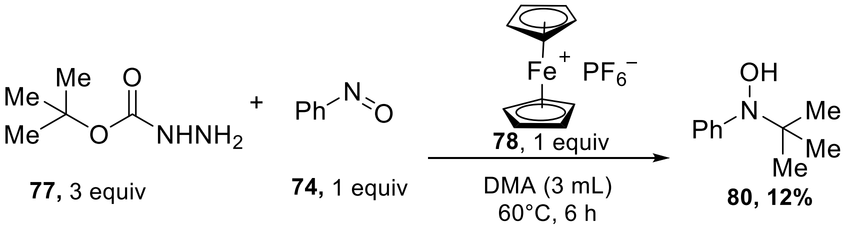

To the bottle was added nitrosobenzene (22.6 mg, 0.2 mmol), tert-Butyl carbazate (27.8 mg, 0.2 mmol), $Fc^{+}PF_6^{-}$ (54.6 mg, 0.2 mmol), and 3 mL DMA. The reaction mixture was stirred at 60 °C under argon atmosphere for 6 h. The GCMS results indicate that the reaction cannot be occurred, and the alkyl radical intermediate was observed.

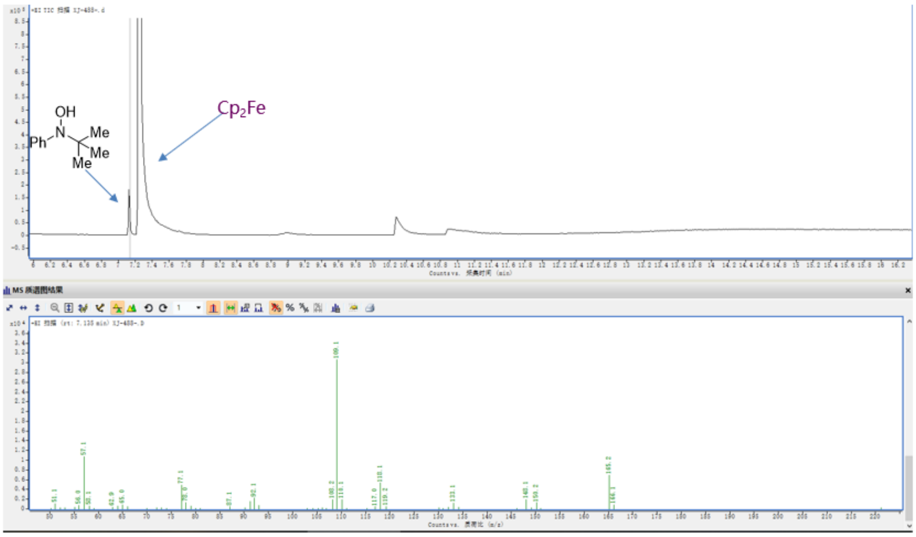

b. Procedure for cyclic voltammetry (CV)

Cyclic voltammetry was performed in a three-electrode cell connected to a schlenk line under nitrogen at room temperature. The working electrode was a steady glassy carbon disk electrode, the counter electrode a platinum wire. The reference was an Ag/AgCl electrode submerged in saturated aqueous KCl solution.

Oxidation potential:

(1) $^{n}Bu_4NClO_4$ (0.4 mmol) and a solvent (DMA, 10 mL) were poured into the electrochemical cell in cyclic voltammetry experiments. The scan rate was 0.025 V/s, ranging from 0V to 1.5 V. (2) Cp_2Fe (1 mM) and a mixed solvent (DMA, 10 mL) containing $^{n}Bu_4NClO_4$ (0.4 mmol) were poured into the electrochemical cell in cyclic voltammetry experiments. The scan rate was 0.025 V/s, ranging from 0V to 0.8 V. (3) **2a** (3 mM) and a mixed solvent (DMA, 10 mL) containing $^{n}Bu_4NClO_4$ (0.4 mmol) were poured into the electrochemical cell in cyclic voltammetry experiments. The scan rate was 0.025 V/s, ranging from 0 V to 1.5 V. (4) tert-Butyl carbazate (3 mM) and a mixed solvent (DMA, 10 mL) containing $^{n}Bu_4NClO_4$ (0.4 mmol) were poured into the electrochemical cell in cyclic voltammetry experiments. The scan rate was 0.025 V/s, ranging from 0V to 1.5 V. (5) tert-Butyl carbazate (3 mM), Cp_2Fe (1 mM) and a mixed solvent (DMA, 10 mL) containing $^{n}Bu_4NClO_4$ (0.4 mmol) were poured into the electrochemical cell in cyclic voltammetry experiments. The scan rate was 0.025 V/s, ranging from 0V to 1.5 V. (Supplementary Figure 3).

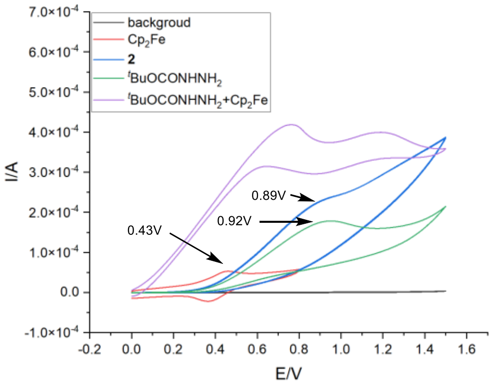

Supplementary Figure 3: Cyclic voltammetry of oxidation

Reduction potential:

(1) $^{n}Bu_4NClO_4$ (0.4 mmol) and a solvent (DMA, 10 mL) were poured into the electrochemical cell in cyclic voltammetry experiments. The scan rate was 0.025 V/s, ranging from 0 V to -2.0 V. (2) $PhNO_2$ (3 mM) and a mixed solvent (DMA, 10 mL) containing $^{n}Bu_4NClO_4$ (0.4 mmol) were poured into the electrochemical cell in cyclic voltammetry experiments. The scan rate was 0.025 V/s, ranging from 0 V to -2.0 V. (3) 4-ipr-$PhNO_2$ (3 mM) and a mixed solvent (DMA, 10 mL) containing $^{n}Bu_4NClO_4$ (0.4 mmol) were poured into the electrochemical cell in cyclic voltammetry experiments. The scan rate was 0.025 V/s, ranging from 0 V to -2.0 V. (4) 4-CN-$PhNO_2$ (3 mM) and a mixed solvent (DMA, 10 mL) containing $^{n}Bu_4NClO_4$ (0.4 mmol) were poured into the electrochemical cell in cyclic voltammetry experiments. The scan rate was 0.025 V/s, ranging from 0 V to -2.0 V. (5) PhNO (3 mM) and a mixed solvent (DMA, 10 mL) containing $^{n}Bu_4NClO_4$ (0.4 mmol) were poured into the electrochemical cell in cyclic voltammetry experiments. The scan rate was 0.025 V/s, ranging from 0 V to -2.0 V. (6) PhNHOH (3 mM) and a mixed solvent (DMA, 10 mL) containing $^{n}Bu_4NClO_4$ (0.4 mmol) were poured into the electrochemical cell in cyclic voltammetry experiments. The scan rate was 0.025 V/s, ranging from 0 V to -2.0 V. (7) $PhNH_2$ (3 mM) and a mixed solvent (DMA, 10 mL) containing $^{n}Bu_4NClO_4$ (0.4 mmol) were poured into the electrochemical cell in cyclic voltammetry experiments. The scan rate was 0.025 V/s, ranging from 0 V to -2.0 V. (Supplementary Figure 4).

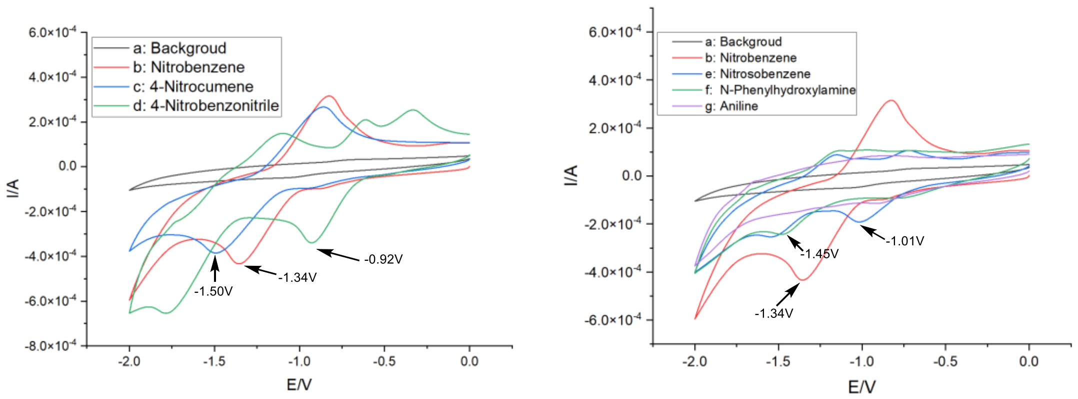

Supplementary Figure 4: Cyclic voltammetry of reduction

c. Computational studies

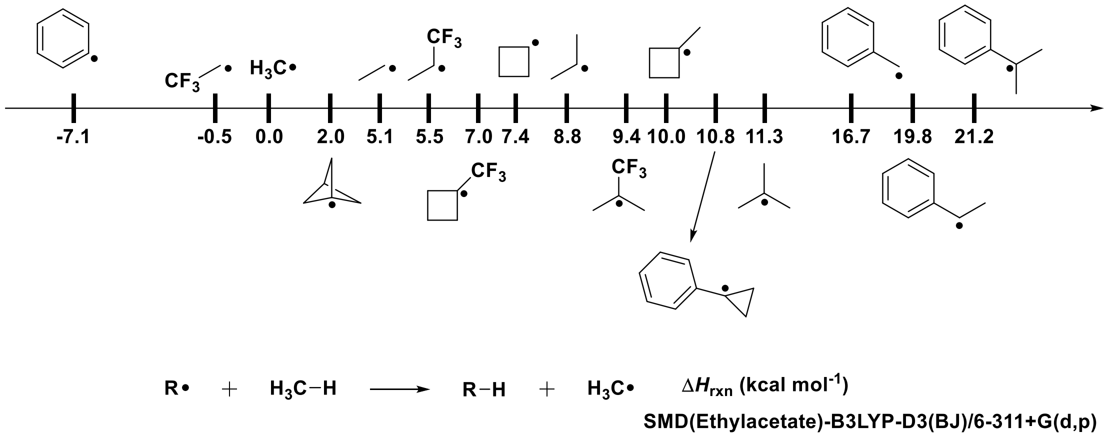

Supplementary Figure 5: The relative stability of representative radicals

As shown in Supplementary Figure 5, a more positive value for the radical exchange reaction means that the formed radical is more stable than the methyl radical. The fluorine substitution makes the corresponding alkyl radical less stable. For instance, the reaction enthalpy of trifluoroethyl radical (-0.5 kcal/mol) is much smaller than that of ethyl radical (5.1 kcal/mol).

8. Characterization Data for Products [2-6]

Compound 3

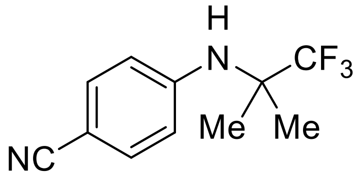

Following **Procedure E** on 0.20 mmol scale. Purification by PTLC (petroleum ether/ethyl acetate = 10:1) afforded 31.0 mg (68%).

Physical State: colorless oil.

^{1}H NMR (400 MHz, CDCl$_3$): δ 7.45 (dd, J = 8.7, 1.9 Hz, 2H), 6.83 (d, J = 7.0 Hz, 2H), 4.17 (s, 1H), 1.58 (s, 6H). **^{13}C NMR (101 MHz, CDCl$_3$):** δ 148.6, 133.3, 127.1 (q, J = 228 Hz), 119.8, 116.9, 101.6, 57.5 (q, J = 22 Hz), 22.8. **^{19}F NMR (376 MHz, Chloroform-d):** δ -79.28. **HRMS (ESI-TOF):** calculated for $C_{11}H_{12}F_3N_2$ $[M+H]^+$: 229.0947, found: 229.0949.

Compound 4

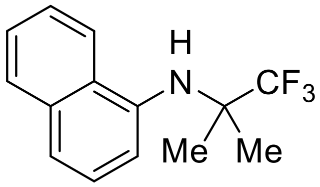

Following **Procedure E** on 0.20 mmol scale. Purification by PTLC (petroleum ether/ethyl acetate = 10:1) afforded 25.8 mg (51%).

Physical State: colorless oil.

^{1}H NMR (400 MHz, CDCl$_3$): 1H NMR (400 MHz, Chloroform-d) δ 7.96 (d, J = 7.7 Hz, 1H), 7.74 (dd, J = 7.0, 2.3 Hz, 1H), 7.45 (d, J = 8.2 Hz, 1H), 7.43 – 7.35 (m, 2H), 7.29 (t, J = 7.8 Hz, 1H), 7.08 (d, J = 7.4 Hz, 1H), 4.24 – 3.25 (m, 1H), 1.40 (s, 6H). **^{13}C NMR (101 MHz, CDCl$_3$):** δ 139.3, 134.6, 129.5, 128.6, 127.8 (q, J = 284 Hz), 125.7, 125.6, 125.6, 123.0, 121.8, 118.9 (d, J = 1 Hz), 58.6 (q, J = 26 Hz), 22.4. **^{19}F NMR (376 MHz, Chloroform-d):** δ -80.22. **HRMS (ESI-TOF):** calculated for $C_{14}H_{15}F_3N$ $[M+H]^+$: 254.1151, found: 254.1153.

Compound 5

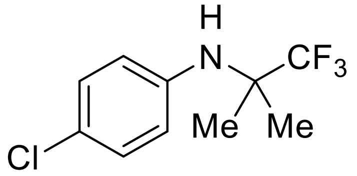

Following **Procedure E** on 0.20 mmol scale. Purification by PTLC (petroleum ether/ethyl acetate = 10:1) afforded 30.3 mg (64%).

Physical State: colorless oil.

^{1}H NMR (400 MHz, $CDCl_3$): δ 7.16 (d, J = 8.8 Hz, 2H), 6.82 (d, J = 8.8 Hz, 2H), 3.47 (s, 1H), 1.43 – 1.42 (m, 6H). **^{13}C NMR (101 MHz, $CDCl_3$):** δ 142.3, 128.8, 127.4 (q, J = 285 Hz), 127.0, 122.8 (d, J = 1 Hz), 58.0 (q, J = 27 Hz), 22.6. **^{19}F NMR (376 MHz, Chloroform-d):** δ -79.52. **HRMS (ESI-TOF):** calculated for $C_{10}H_{12}ClF_3N$ $[M+H]^+$: 238.0605, found: 238.0607.

Compound 6

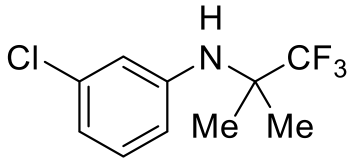

Following **Procedure E** on 0.20 mmol scale. Purification by PTLC (petroleum ether/ethyl acetate = 10:1) afforded 27.1 mg (57%).

Physical State: colorless oil.

^{1}H NMR (400 MHz, $CDCl_3$): δ 7.10 (t, J = 8.0 Hz, 1H), 6.88 (ddd, J = 7.9, 1.9, 0.9 Hz, 1H), 6.86 (t, J = 2.1 Hz, 1H), 6.73 (ddd, J = 8.2, 2.3, 0.9 Hz, 1H), 3.54 (s, 1H), 1.45 (q, J = 0.8 Hz, 6H). **^{13}C NMR (101 MHz, $CDCl_3$):** δ 145.7, 134.5, 127.4 (q, J = 295 Hz), 121.4, 120.4, 118.7, 57.9 (q, J = 27 Hz), 22.7. **^{19}F NMR (376 MHz, Chloroform-d):** δ -79.47. **HRMS (ESI-TOF):** calculated for $C_{10}H_{16}N$ $[M+H]^+$: 238.0605, found: 238.0602.

Compound 7

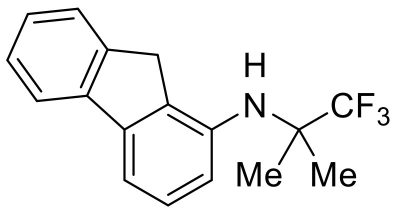

Following **Procedure E** on 0.20 mmol scale. Purification by PTLC (petroleum ether/ethyl acetate = 10:1) afforded 36.8 mg (63%).

Physical State: colorless oil.

^{1}H NMR (400 MHz, $CDCl_3$): δ 7.67 (d, J = 7.6 Hz, 1H), 7.60 (d, J = 8.1 Hz, 1H), 7.48 (d, J = 7.4 Hz, 1H), 7.32 (t, J = 7.5 Hz, 1H), 7.22 (t, J = 7.0 Hz, 1H), 7.09 (s, 1H), 6.91 (dd, J = 8.1, 1.9 Hz, 1H), 3.83 (s, 2H), 3.48 (s, 1H), 1.46 (s, 6H). **^{13}C NMR (101 MHz, $CDCl_3$):** δ 144.3, 143.2, 142.8, 141.6, 136.2, 127.6 (q, J = 285 Hz), 126.7, 125.8, 124.9, 121.1, 120.1, 119.2, 118.9, 58.3 (q, J = 27 Hz), 36.9, 22.7. **^{19}F NMR (376 MHz, Chloroform-d):** δ -79.54. **HRMS (ESI-TOF):** calculated for $C_{17}H_{17}F_3N$ $[M+H]^+$: 292.1308, found: 292.1310.

Compound 8

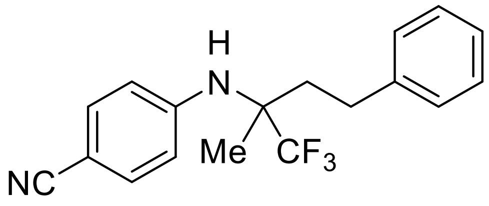

Following **Procedure E** on 0.20 mmol scale. Purification by PTLC (petroleum ether/ethyl acetate = 10:1) afforded 49.1 mg (77%).

Physical State: colorless oil.

^{1}H NMR (400 MHz, $CDCl_3$): δ 7.45 (d, J = 8.6 Hz, 2H), 7.29 (t, J = 7.3 Hz, 2H), 7.25 – 7.19 (m, 1H), 7.10 (d, J = 7.3 Hz, 2H), 6.78 (d, J = 8.6 Hz, 2H), 4.08 (s, 1H), 2.72 (dt, J = 12.3, 6.7 Hz, 1H), 2.62 (td, J = 13.5, 12.8, 4.8 Hz, 1H), 2.38 (td, J = 13.3, 12.0, 4.8 Hz, 1H), 2.08 (ddd, J = 17.1, 13.2, 5.5 Hz, 1H), 1.60 (s, 3H). **^{13}C NMR (101 MHz, $CDCl_3$):** δ 148.8, 140.5, 133.5, 128.8, 128.2, 127.1 (q, J = 285 Hz), 126.5, 119.7, 116.4, 101.6, 60.3 (q, J = 27 Hz), 35.5, 29.0, 20.7. **^{19}F NMR (376 MHz, Chloroform-d):** δ -

77.95. **HRMS (ESI-TOF):** calculated for $C_{18}H_{18}F_3N_2$ $[M+H]^+$: 319.1417, found: 319.1414.

Compound 9

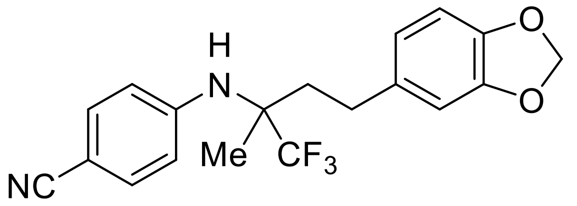

Following **Procedure E** on 0.20 mmol scale. Purification by PTLC (petroleum ether/ethyl acetate = 10:1) afforded 60.3 mg (83%).

Physical State: colorless oil.

^{1}H NMR (400 MHz, CDCl$_3$): δ 7.48 – 7.43 (m, 2H), 6.79 (d, J = 8.8 Hz, 2H), 6.72 (d, J = 7.9 Hz, 1H), 6.58 (d, J = 1.6 Hz, 1H), 6.54 (dd, J = 7.9, 1.7 Hz, 1H), 5.93 (s, 2H), 4.06 (s, 1H), 2.68 – 2.60 (m, 1H), 2.55 (dt, J = 13.7, 6.9 Hz, 1H), 2.37 – 2.29 (m, 1H), 2.06 – 1.99 (m, 1H), 1.58 (s, 3H). **^{13}C NMR (101 MHz, CDCl$_3$):** δ 148.7, 147.9, 146.1, 134.2, 133.5, 127.1 (q, J = 286 Hz), 121.0, 119.7, 116.3, 108.7, 108.4, 101.8, 101.0, 60.3 (q, J = 26 Hz), 35.7, 28.8, 20.8. **^{19}F NMR (376 MHz, Chloroform-d):** δ -77.98. **HRMS (ESI-TOF):** calculated for $C_{19}H_{18}F_3N_2O_2$ $[M+H]^+$: 363.1315, found: 363.1313.

Compound 10

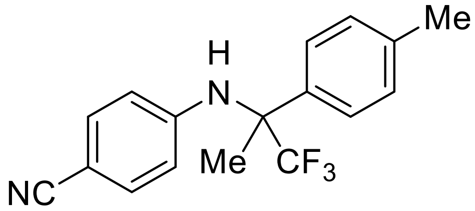

Following **Procedure E** on 0.20 mmol scale. Purification by PTLC (petroleum ether/ethyl acetate = 10:1) afforded 43.9 mg (72%).

Physical State: colorless oil.

^{1}H NMR (400 MHz, CDCl$_3$): δ 7.46 (d, J = 8.7 Hz, 2H), 7.40 (d, J = 8.1 Hz, 2H), 7.19 (d, J = 8.0 Hz, 2H), 6.94 (s, 1H), 6.91 (d, J = 8.6 Hz, 2H), 2.32 (s, 3H), 1.82 (s, 3H). **^{13}C NMR (101 MHz, CDCl$_3$):** δ 152.0, 139.4, 133.4, 132.8, 129.5, 127.0, 124.7 (q, J = 284 Hz), 119.3, 113.9, 104.8, 84.1 (q, J = 27 Hz), 21.1, 17.4. **^{19}F NMR (376 MHz,

Chloroform-d): δ -77.62. **HRMS (ESI-TOF):** calculated for $C_{17}H_{16}F_3N_2$ $[M+H]^+$: 305.1260, found: 305.1259.

Compound 11

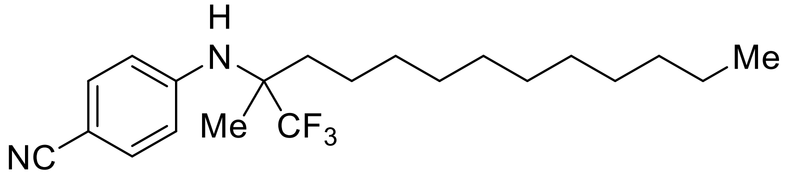

Following **Procedure E** on 0.20 mmol scale. Purification by PTLC (petroleum ether/ethyl acetate = 10:1) afforded 59.8 mg (81%).

Physical State: colorless oil.

^{1}H NMR (400 MHz, CDCl$_3$): δ 7.36 (d, J = 8.9 Hz, 2H), 6.71 (d, J = 8.8 Hz, 2H), 3.88 (s, 1H), 1.99 – 1.90 (m, 1H), 1.70 – 1.62 (m, 1H), 1.52 (s, 1H), 1.42 (s, 3H), 1.20 (d, J = 22.0 Hz, 17H), 0.81 (t, J = 6.9 Hz, 3H). **^{13}C NMR (101 MHz, CDCl$_3$):** δ 149.0, 133.3, 127.2 (q, J = 286 Hz), 119.7, 116.3, 101.5, 60.3 (q, J = 27 Hz), 33.9, 31.9, 29.6, 29.6, 29.66, 29.5, 29.4, 29.3, 22.7, 22.4, 20.5, 14.1. **^{19}F NMR (376 MHz, Chloroform-d):** δ -78.10. **HRMS (ESI-TOF):** calculated for $C_{21}H_{32}F_3N_2$ $[M+H]^+$: 369.2512, found: 369.2513.

Compound 12

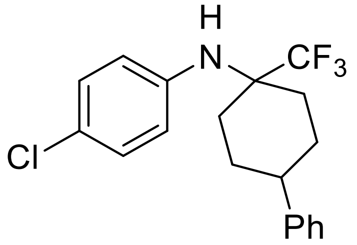

Following **Procedure E** on 0.20 mmol scale. Purification by PTLC (petroleum ether/ethyl acetate = 10:1) afforded 52.4 mg (74%).

Physical State: colorless oil.

^{1}H NMR (400 MHz, CDCl$_3$): δ 7.25 – 7.17 (m, 3H), 7.16 – 7.12 (m, 2H), 7.10 (d, J = 8.7 Hz, 4H), 6.80 – 6.75 (m, 2H), 3.25 (s, 1H), 2.58 – 2.48 (m, 1H), 2.14 (dd, J = 14.2, 2.6 Hz, 2H), 1.85 – 1.65 (m, 6H). **^{13}C NMR (101 MHz, CDCl$_3$):** δ 145.8, 143.4, 129.1,

128.5, 127.8 (q, J = 285 Hz), 126.7, 126.4, 120.6, 59.4 (q, J = 26 Hz), 43.2, 29.2, 27.7. **^{19}F NMR (376 MHz, Chloroform-d):** δ -78.96. **HRMS (ESI-TOF):** calculated for $C_{19}H_{20}ClF_3N$ $[M+H]^+$: 354.1231, found: 354.1233.

Compound 13

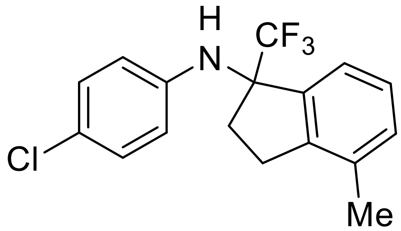

Following **Procedure E** on 0.20 mmol scale. Purification by PTLC (petroleum ether/ethyl acetate = 10:1) afforded 44.9 mg (69%).

Physical State: colorless oil.

^{1}H NMR (400 MHz, $CDCl_3$): δ 7.48 (d, J = 7.6 Hz, 1H), 7.19 (t, J = 7.5 Hz, 1H), 7.11 (d, J = 7.4 Hz, 1H), 7.02 – 6.95 (m, 2H), 6.77 – 6.72 (m, 2H), 5.90 (s, 1H), 2.55 (dd, J = 15.7, 9.0 Hz, 2H), 2.35 – 2.27 (m, 1H), 2.07 (s, 3H), 1.77 (dt, J = 16.6, 8.3 Hz, 1H). **^{13}C NMR (101 MHz, $CDCl_3$):** δ 146.6, 145.1, 135.9, 134.1, 131.5, 130.5, 127.6, 126.7, 126.4 (q, J = 285 Hz), 125.8, 124.6, 80.2 (q, J = 26 Hz), 28.5, 28.0, 18.8. **^{19}F NMR (376 MHz, Chloroform-d):** δ -71.03. **HRMS (ESI-TOF):** calculated for $C_{17}H_{16}ClF_3N$ $[M+H]^+$: 326.0918, found: 326.0917.

Compound 14

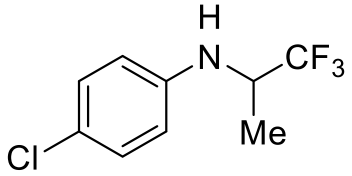

Following **Procedure E** on 0.20 mmol scale. Purification by PTLC (petroleum ether/ethyl acetate = 10:1) afforded 17.4 mg (39%).

Physical State: colorless oil.

^{1}H NMR (400 MHz, $CDCl_3$): δ 7.23 – 7.17 (m, 2H), 6.48 – 6.44 (m, 2H), 3.88 (s, 1H), 3.52 (s, 1H), 1.31 (d, J = 6.7 Hz, 3H). **^{13}C NMR (101 MHz, $CDCl_3$):** δ 145.0, 132.1, 126.1 (q, J = 281 Hz), 115.1, 110.5, 51.4 (q, J = 30 Hz), 15.1. **^{19}F NMR (376 MHz, Chloroform-d):** δ -77.38. **HRMS (ESI-TOF):** calculated for $C_9H_{10}ClF_3N$ $[M+H]^+$:

224.0448, found: 224.0449.

Compound 15

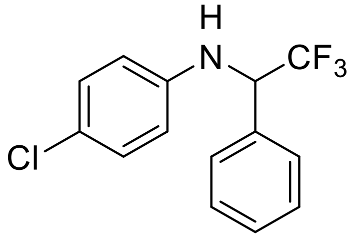

Following **Procedure E** on 0.20 mmol scale. Purification by PTLC (petroleum ether/ethyl acetate = 10:1) afforded 18.9 mg (33%).

Physical State: colorless oil.

^{1}H NMR (400 MHz, CDCl$_3$): δ 7.50 – 7.33 (m, 5H), 7.09 (d, J = 8.8 Hz, 2H), 6.55 (d, J = 8.8 Hz, 2H), 4.85 (p, J = 7.0 Hz, 1H), 4.33 (d, J = 5.4 Hz, 1H). **^{13}C NMR (101 MHz, CDCl$_3$):** δ 144.0, 133.6, 129.3, 129.0, 127.9, 124.9 (q, J = 281 Hz), 124.0, 115.1, 60.7 (q, J = 30 Hz). **^{19}F NMR (376 MHz, Chloroform-d):** δ -73.99. **HRMS (ESI-TOF):** calculated for $C_{14}H_{12}ClF_3N$ [M+H]$^+$: 286.0605, found: 286.0604.

Compound 16

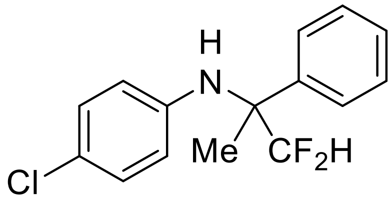

Following **Procedure E** on 0.20 mmol scale. Purification by PTLC (petroleum ether/ethyl acetate = 10:1) afforded 41.7 mg (74%).

Physical State: colorless oil.

^{1}H NMR (400 MHz, CDCl$_3$): δ 7.46 (d, J = 7.5 Hz, 2H), 7.30 (dt, J = 13.4, 6.9 Hz, 3H), 6.88 (d, J = 8.7 Hz, 2H), 6.20 (d, J = 8.7 Hz, 2H), 5.70 (t, J = 56.4 Hz, 1H), 4.15 (s, 1H), 1.66 (s, 3H). **^{13}C NMR (101 MHz, CDCl$_3$):** δ 143.1, 138.8, 129.0, 128.7, 128.2, 127.2, 123.5, 117.4 (t, J = 250 Hz), 117.4, 61.4 (t, J = 21 Hz), 17.9. **^{19}F NMR (376 MHz, Chloroform-d):** δ -129.32, -130.16 (ABq, J = 274 Hz, 2F). **HRMS (ESI-TOF):** calculated for $C_{15}H_{15}ClF_2N$ [M+H]$^+$: 282.0856, found: 282.0857.

Compound 17

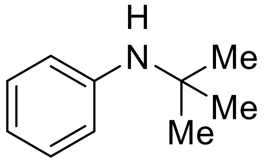

Following **Procedure E** on 0.20 mmol scale. Purification by PTLC (petroleum ether/ethyl acetate = 10:1) afforded 21.5 mg (72%).

Physical State: colorless oil.

^{1}H NMR (400 MHz, CDCl$_3$): δ 7.19 – 7.12 (m, 2H), 6.78 – 6.73 (m, 3H), 3.09 (d, J = 42.9 Hz, 1H), 1.33 (s, 9H). **^{13}C NMR (101 MHz, CDCl$_3$):** δ 146.8, 128.9, 118.4, 117.6, 51.6, 30.1. **HRMS (ESI-TOF):** calculated for $C_{10}H_{16}N$ $[M+H]^+$: 150.1277, found: 150.1276.

Compound 18

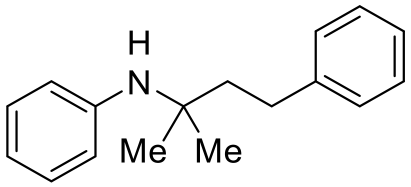

Following **Procedure E** on 0.20 mmol scale. Purification by PTLC (petroleum ether/ethyl acetate = 10:1) afforded 35.4 mg (74%).

Physical State: colorless oil.

^{1}H NMR (400 MHz, CDCl$_3$): δ 7.27 – 7.22 (m, 2H), 7.18 – 7.09 (m, 5H), 6.82 – 6.67 (m, 3H), 3.29 (s, 1H), 2.68 – 2.63 (m, 2H), 1.98 – 1.93 (m, 2H), 1.36 (s, 6H). **^{13}C NMR (101 MHz, CDCl$_3$):** 142.7, 129.1, 128.5, 128.4, 128.4, 125.7, 118.2, 116.9, 53.9, 43.5, 30.7, 28.6. **HRMS (ESI-TOF):** calculated for $C_{17}H_{22}N$ $[M+H]^+$: 240.1747, found: 240.1748.

Compound 19

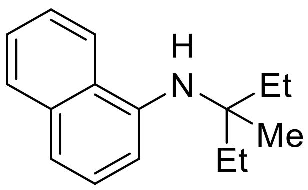

Following **Procedure E** on 0.20 mmol scale. Purification by PTLC (petroleum ether/ethyl acetate = 8:1) afforded 30.9 mg (68%).

Physical State: colorless oil.

^{1}H NMR (400 MHz, CDCl$_3$): δ 7.84 – 7.71 (m, 2H), 7.47 – 7.35 (m, 2H), 7.27 (t, J = 7.9 Hz, 1H), 7.18 (d, J = 8.1 Hz, 1H), 6.84 (d, J = 7.6 Hz, 1H), 4.15 (s, 1H), 1.88 (dt, J = 14.8, 7.3 Hz, 2H), 1.75 (dt, J = 14.2, 7.4 Hz, 2H), 1.35 (s, 3H), 0.89 (t, J = 7.5 Hz, 6H). **^{13}C NMR (101 MHz, CDCl$_3$):** δ 141.7, 134.7, 128.9, 126.3, 125.4, 124.5, 124.4, 119.9, 116.8, 107.4, 56.6, 31.4, 25.0, 8.1. **HRMS (ESI-TOF):** calculated for $C_{16}H_{22}N$ $[M+H]^+$: 228.1747, found: 228.1749.

Compound 20

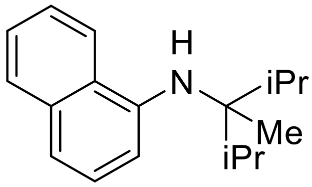

Following **Procedure E** on 0.20 mmol scale. Purification by PTLC (petroleum ether/ethyl acetate = 10:1) afforded 19.4 mg (38%).

Physical State: colorless oil.

^{1}H NMR (400 MHz, CDCl$_3$): δ 7.83 – 7.74 (m, 2H), 7.43 – 7.38 (m, 2H), 7.25 – 7.23 (m, 1H), 7.16 (d, J = 8.1 Hz, 1H), 6.88 (d, J = 7.7 Hz, 1H), 4.27 (s, 1H), 2.28 (p, J = 6.8 Hz, 2H), 1.33 (s, 3H), 1.02 (dd, J = 6.8, 3.0 Hz, 12H). **^{13}C NMR (101 MHz, CDCl$_3$):** 134.8, 129.0, 126.3, 125.3, 124.5, 124.5, 119.9, 116.6, 116.5, 108.7, 62.3, 35.8, 18.7, 18.2. **HRMS (ESI-TOF):** calculated for $C_{18}H_{26}N$ $[M+H]^+$: 256.2060, found: 256.2063.

Compound 21

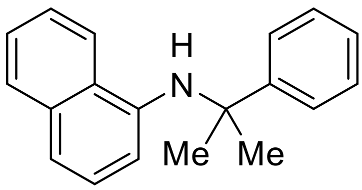

Following **Procedure E** on 0.20 mmol scale. Purification by PTLC (petroleum ether/ethyl acetate = 15:1) afforded 37.6 mg (72%).

Physical State: colorless oil.

^{1}H NMR (400 MHz, CDCl$_3$): δ 7.91 – 7.88 (m, 1H), 7.75 (dd, J = 7.0, 2.5 Hz, 1H), 7.51 (dd, J = 8.3, 1.1 Hz, 2H), 7.48 – 7.40 (m, 2H), 7.31 (t, J = 7.6 Hz, 2H), 7.23 (dt, J = 8.2, 1.6 Hz, 1H), 7.11 (d, J = 8.1 Hz, 1H), 7.03 (t, J = 7.9 Hz, 1H), 6.10 – 6.04 (m, 1H), 4.82 (s, 1H), 1.77 (s, 6H). **^{13}C NMR (101 MHz, CDCl$_3$):** δ 147.0, 140.3, 134.4, 128.9, 126.4, 126.0, 125.5, 125.4, 124.6, 123.8, 119.7, 116.7, 108.7, 56.0, 30.7. **HRMS (ESI-TOF):** calculated for $C_{19}H_{20}N$ $[M+H]^+$: 262.1590, found: 262.1593.

Compound 22

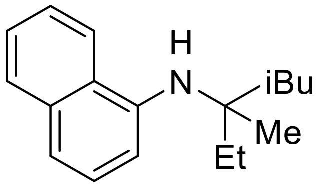

Following **Procedure E** on 0.20 mmol scale. Purification by PTLC (petroleum ether/ethyl acetate = 15:1) afforded 30.1 mg (59%).

Physical State: colorless oil.

^{1}H NMR (400 MHz, CDCl$_3$):δ 7.79 – 7.73 (m, 2H), 7.46 – 7.37 (m, 2H), 7.27 (dd, J = 8.0, 1.8 Hz, 1H), 7.15 (d, J = 8.1 Hz, 1H), 6.80 (d, J = 7.7 Hz, 1H), 4.26 (s, 1H), 2.09 (dddt, J = 14.2, 11.5, 7.3, 3.6 Hz, 1H), 1.97 (dq, J = 14.7, 7.3 Hz, 1H), 1.84 – 1.75 (m, 1H), 1.65 (dtd, J = 17.8, 8.4, 7.3, 3.6 Hz, 1H), 1.26 (d, J = 4.0 Hz, 3H), 1.05 (dddt, J = 13.3, 10.3, 6.9, 2.6 Hz, 2H), 0.96 – 0.92 (m, 3H), 0.91 – 0.82 (m, 6H). **^{13}C NMR (101 MHz, CDCl$_3$):** δ 141.6, 134.8, 128.9, 126.4, 125.4, 124.5, 124.1, 119.8, 116.4, 106.9, 59.6, 59.5, 41.5, 41.4, 29.1, 29.0, 24.4, 23.9, 21.7, 21.6, 13.9, 13.4, 13.3, 13.1, 8.1, 8.0. **HRMS (ESI-TOF):** calculated for $C_{18}H_{26}N$ $[M+H]^+$: 256.2060, found: 256.2061.

Compound 23

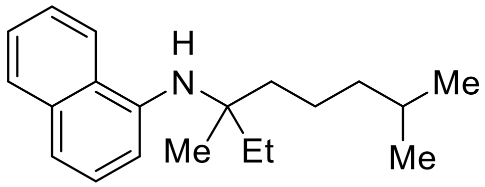

Following **Procedure E** on 0.20 mmol scale. Purification by PTLC (petroleum ether/ethyl acetate = 15:1) afforded 37.4 mg (72%).

Physical State: colorless oil.

^{1}H NMR (400 MHz, CDCl$_3$): δ 7.81 – 7.73 (m, 2H), 7.44 – 7.37 (m, 2H), 7.28 (t, J = 7.9 Hz, 1H), 7.18 (d, J = 8.1 Hz, 1H), 6.84 (d, J = 7.6 Hz, 1H), 4.19 (s, 1H), 1.87 (tt, J = 13.4, 6.7 Hz, 1H), 1.81 – 1.71 (m, 2H), 1.70 – 1.63 (m, 1H), 1.50 (dt, J = 13.3, 6.7 Hz, 1H), 1.36 (s, 3H), 1.34 – 1.24 (m, 2H), 1.15 (q, J = 7.4 Hz, 2H), 0.88 (t, J = 7.5 Hz, 3H), 0.82 (d, J = 6.6 Hz, 6H). **^{13}C NMR (101 MHz, CDCl$_3$):** δ 141.7, 134.7, 128.9, 126.3, 125.3, 124.5, 124.5, 119.9, 116.8, 107.5, 56.5, 39.6, 39.5, 31.7, 27.8, 25.5, 22.6, 21.4, 8.2. **HRMS (ESI-TOF):** calculated for $C_{20}H_{30}N$ $[M+H]^+$: 284.2373, found: 284.2376.

Compound 24

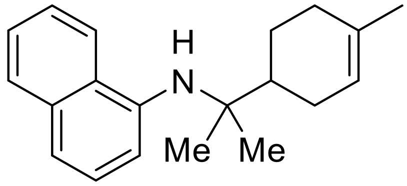

Following **Procedure E** on 0.20 mmol scale. Purification by PTLC (petroleum ether/ethyl acetate = 15:1) afforded 37.5 mg (67%).

Physical State: colorless oil.

^{1}H NMR (400 MHz, CDCl$_3$): δ 7.82 – 7.73 (m, 2H), 7.45 – 7.36 (m, 2H), 7.30 – 7.25 (m, 1H), 7.20 (d, J = 8.1 Hz, 1H), 6.89 (dd, J = 7.6, 0.9 Hz, 1H), 5.38 (s, 1H), 4.30 (s, 1H), 2.22 (tdd, J = 11.9, 4.7, 2.2 Hz, 1H), 2.14 – 1.82 (m, 6H), 1.65 (s, 3H), 1.41 (d, J = 14.3 Hz, 6H). **^{13}C NMR (101 MHz, CDCl$_3$):** δ 141.5, 134.7, 134.1, 128.9, 126.3,

125.4, 124.9, 124.6, 120.9, 120.1, 117.1, 108.6, 56.3, 42.0, 31.4, 26.9, 25.4, 25.2, 24.5, 23.3. **HRMS (ESI-TOF):** calculated for $C_{20}H_{26}N$ $[M+H]^+$: 280.2060, found: 280.2104.

Compound 25

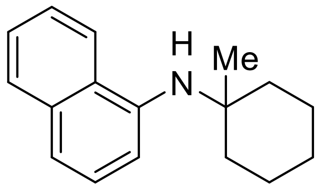

Following **Procedure E** on 0.20 mmol scale. Purification by PTLC (petroleum ether/ethyl acetate = 15:1) afforded 27.7 mg (58%).

Physical State: colorless oil.

^{1}H NMR (400 MHz, $CDCl_3$): δ 7.79 – 7.73 (m, 1H), 7.71 – 7.67 (m, 1H), 7.38 – 7.30 (m, 2H), 7.22 (t, J = 7.9 Hz, 1H), 7.13 (d, J = 8.1 Hz, 1H), 6.81 (d, J = 7.5 Hz, 1H), 4.00 (s, 1H), 2.00 (d, J = 10.1 Hz, 2H), 1.56 – 1.43 (m, 8H), 1.37 (s, 3H). **^{13}C NMR (101 MHz, $CDCl_3$):** δ 140.3, 133.7, 127.8, 125.1, 124.3, 124.0, 123.6, 119.0, 116.0, 107.8, 52.3, 37.1, 25.7, 24.8, 21.3. **HRMS (ESI-TOF):** calculated for $C_{17}H_{22}N$ $[M+H]^+$: 240.1747, found: 240.1748.

Compound 26

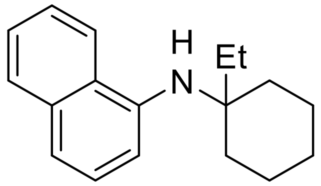

Following **Procedure E** on 0.20 mmol scale. Purification by PTLC (petroleum ether/ethyl acetate = 15:1) afforded 30.9 mg (61%).

Physical State: colorless oil.

^{1}H NMR (400 MHz, $CDCl_3$): δ 7.86 – 7.81 (m, 1H), 7.79 – 7.73 (m, 1H), 7.42 (dt, J = 6.7, 3.4 Hz, 2H), 7.26 (t, J = 7.9 Hz, 1H), 7.16 (d, J = 8.1 Hz, 1H), 6.80 (d, J = 7.6 Hz, 1H), 4.21 (s, 1H), 2.13 (d, J = 12.8 Hz, 2H), 1.86 (q, J = 7.5 Hz, 2H), 1.54 (tdd, J = 21.0, 12.0, 3.3 Hz, 7H), 1.33 (ddt, J = 16.3, 9.6, 4.1 Hz, 1H), 0.82 (t, J = 7.5 Hz, 3H). **^{13}C**

NMR (101 MHz, $CDCl_3$): δ 141.4, 134.8, 128.9, 126.3, 125.3, 124.5, 124.3, 119.8, 116.4, 107.1, 55.7, 35.8, 31.1, 26.1, 22.0, 7.4. **HRMS (ESI-TOF):** calculated for $C_{18}H_{24}N$ $[M+H]^+$: 254.1903, found: 154.1906.

Compound 27

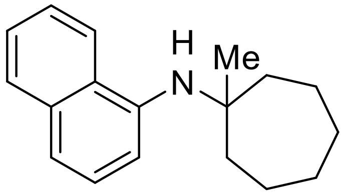

Following **Procedure E** on 0.20 mmol scale. Purification by PTLC (petroleum ether/ethyl acetate = 10:1) afforded 30.9 mg (61%).

Physical State: colorless oil.

^{1}H NMR (400 MHz, $CDCl_3$): δ 7.13 (t, J = 7.9 Hz, 2H), 6.67 (t, J = 8.1 Hz, 3H), 3.34 (s, 1H), 1.97 – 1.90 (m, 2H), 1.78 – 1.66 (m, 6H), 1.40 (s, 3H). **^{13}C NMR (101 MHz, $CDCl_3$):** δ 146.8, 129.0, 117.0, 115.2, 61.5, 40.7, 26.0, 24.4. **HRMS (ESI-TOF):** calculated for $C_{18}H_{24}N$ $[M+H]^+$: 254.1903, found: 254.1904.

Compound 28

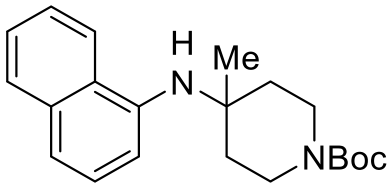

Following **Procedure E** on 0.20 mmol scale. Purification by PTLC (petroleum ether/ethyl acetate = 10:1) afforded 49.0 mg (72%).

Physical State: colorless oil.

^{1}H NMR (400 MHz, $CDCl_3$): δ 7.85 – 7.76 (m, 2H), 7.46 – 7.41 (m, 2H), 7.33 – 7.25 (m, 2H), 6.87 (dd, J = 7.2, 1.2 Hz, 1H), 4.14 (s, 1H), 3.65 (s, 2H), 3.37 (ddd, J = 13.3, 10.2, 3.0 Hz, 2H), 2.11 (d, J = 13.8 Hz, 2H), 1.75 – 1.67 (m, 2H), 1.47 (s, 3H), 1.45 (s, 9H). **^{13}C NMR (101 MHz, $CDCl_3$):** δ 154.9, 140.7, 134.7, 128.9, 126.0, 125.6, 125.4,

124.9, 120.0, 118.2, 109.8, 79.5, 69.6, 51.8, 37.4, 28.5, 26.1. **HRMS (ESI-TOF):** calculated for $C_{21}H_{29}N_2O_2$ $[M+H]^+$: 341.2224, found: 341.2226.

Compound 29

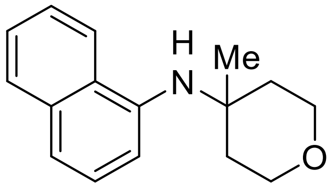

Following **Procedure E** on 0.20 mmol scale. Purification by PTLC (petroleum ether/ethyl acetate = 10:1) afforded 31.8 mg (66%).

Physical State: colorless oil.

^{1}H NMR (400 MHz, $CDCl_3$): 1H NMR (400 MHz, Chloroform-d) δ 7.84 (dd, J = 6.2, 3.4 Hz, 1H), 7.78 (dd, J = 6.0, 3.5 Hz, 1H), 7.44 (dd, J = 6.4, 3.3 Hz, 2H), 7.33 – 7.24 (m, 2H), 6.86 (dd, J = 7.3, 1.1 Hz, 1H), 4.17 (s, 1H), 3.86 – 3.80 (m, 2H), 3.76 – 3.68 (m, 2H), 2.11 (dt, J = 15.4, 3.4 Hz, 2H), 1.84 (ddd, J = 13.4, 9.0, 4.0 Hz, 2H), 1.50 (s, 3H). **^{13}C NMR (101 MHz, $CDCl_3$):** δ 140.5, 134.7, 128.9, 126.0, 125.6, 125.3, 124.9, 120.0, 118.1, 109.7, 69.6, 64.0, 38.3, 26.2. **HRMS (ESI-TOF):** calculated for $C_{16}H_{20}NO$ $[M+H]^+$: 242.1539, found: 242.1538.

Compound 30

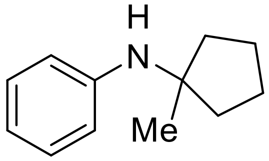

Following **Procedure E** on 0.20 mmol scale. Purification by PTLC (petroleum ether/ethyl acetate = 10:1) afforded 24.2 mg (69%).

Physical State: colorless oil.

^{1}H NMR (400 MHz, $CDCl_3$): δ 7.13 (t, J = 7.9 Hz, 2H), 6.67 (t, J = 8.1 Hz, 3H), 3.34 (s, 1H), 1.97 – 1.90 (m, 2H), 1.78 – 1.66 (m, 6H), 1.40 (s, 3H). **^{13}C NMR (101 MHz,**

CDCl3): δ 146.8, 129.0, 117.0, 115.2, 61.5, 40.7, 26.0, 24.4. **HRMS (ESI-TOF):** calculated for $C_{12}H_{18}N$ $[M+H]^+$: 176.1434, found: 176.1435.

Compound 31

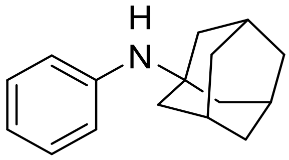

Following **Procedure E** on 0.20 mmol scale. Purification by PTLC (petroleum ether/ethyl acetate = 10:1) afforded 32.3 mg (71%).

Physical State: colorless oil.

^{1}H NMR (400 MHz, CDCl3): δ 7.18 – 7.12 (m, 2H), 6.83 – 6.77 (m, 3H), 3.09 (s, 1H), 2.10 (s, 3H), 1.87 (d, J = 2.8 Hz, 6H), 1.67 (s, 6H). **^{13}C NMR (101 MHz, CDCl3):** δ 145.9, 128.7, 119.2, 119.2, 52.3, 43.5, 36.5, 29.7. **HRMS (ESI-TOF):** calculated for $C_{16}H_{22}N$ $[M+H]^+$: 228.1747, found: 228.1745.

Compound 32a + 32b

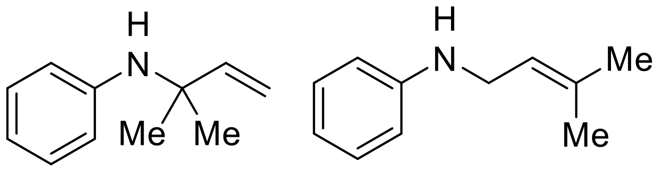

Following **Procedure E** on 0.20 mmol scale. Purification by PTLC (petroleum ether/ethyl acetate = 10:1) afforded 17.4 mg (30% +24%).

Physical State: colorless oil.

^{1}H NMR (400 MHz, CDCl3, 22a, 22b): δ 7.20 – 7.14 (m, 2H), 7.10 (tt, J = 7.4, 2.1 Hz, 2H), 6.70 (dd, J = 8.4, 2.0 Hz, 3H), 6.61 (dd, J = 8.6, 1.0 Hz, 2H), 6.01 (dd, J = 17.5, 10.7 Hz, 1H), 5.36 – 5.30 (m, 1H), 5.18 (dd, J = 17.5, 1.0 Hz, 1H), 5.10 (dd, J = 10.7, 1.1 Hz, 1H), 3.68 (d, J = 6.7 Hz, 4H), 1.74 (s, 3H), 1.71 (s, 3H), 1.38 (s, 6H). **^{13}C NMR (101 MHz, CDCl3):** δ 148.4, 146.0, 135.6, 129.2, 128.7, 121.7, 117.7, 117.3, 116.0, 112.9, 112.8, 54.8, 42.0, 28.2, 25.7, 18.0. **HRMS (ESI-TOF):** calculated for $C_{11}H_{16}N$ $[M+H]^+$: 162.1277, found: 162.1278.

Compound 33

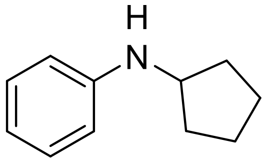

Following **Procedure E** on 0.20 mmol scale. Purification by PTLC (petroleum ether/ethyl acetate = 10:1) afforded 20.0 mg (62%).

Physical State: colorless oil.

^{1}H NMR (400 MHz, CDCl$_3$): δ 7.16 (dd, J = 8.6, 7.3 Hz, 2H), 6.65 (s, 1H), 6.59 (dd, J = 8.6, 1.0 Hz, 2H), 3.83 – 3.75 (m, 1H), 3.56 (s, 1H), 2.01 (dq, J = 13.8, 7.4 Hz, 2H), 1.77 – 1.67 (m, 2H), 1.66 – 1.58 (m, 2H), 1.46 (td, J = 12.5, 11.2, 6.8 Hz, 2H). **^{13}C NMR (101 MHz, CDCl$_3$):** δ 148.0, 129.2, 116.9, 113.2, 54.7, 33.6, 24.1. **HRMS (ESI-TOF):** calculated for $C_{11}H_{16}N$ [M+H]$^+$: 162.1277, found: 162.1275.

Compound 34

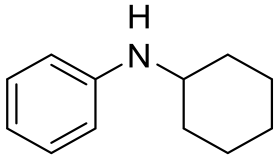

Following **Procedure E** on 0.20 mmol scale. Purification by PTLC (petroleum ether/ethyl acetate = 10:1) afforded 20.3 mg (58%).

Physical State: colorless oil.

^{1}H NMR (400 MHz, CDCl$_3$): δ 7.17 – 7.07 (m, 2H), 6.63 (t, J = 7.3 Hz, 1H), 6.55 (d, J = 7.7 Hz, 2H), 3.39 (s, 1H), 3.22 (tt, J = 10.1, 3.7 Hz, 1H), 2.02 (dt, J = 12.1, 3.8 Hz, 2H), 1.73 (dq, J = 11.7, 3.9 Hz, 2H), 1.63 (dq, J = 11.4, 3.9, 2.8 Hz, 1H), 1.33 (ddd, J = 14.8, 9.2, 3.3 Hz, 2H), 1.21 (ddd, J = 15.6, 9.6, 3.3 Hz, 1H), 1.16 – 1.06 (m, 2H). **^{13}C NMR (101 MHz, CDCl$_3$):** δ 147.5, 129.4, 116.9, 113.3, 51.8, 33.6, 26.1, 25.2. **HRMS (ESI-TOF):** calculated for $C_{12}H_{18}N$ [M+H]$^+$: 176.1434, found: 176.1435.

Compound 35

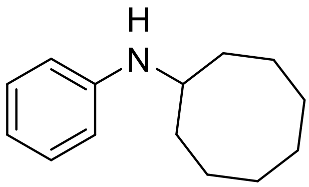

Following **Procedure E** on 0.20 mmol scale. Purification by PTLC (petroleum ether/ethyl acetate = 10:1) afforded 23.6 mg (58%).

Physical State: colorless oil.

^{1}H NMR (400 MHz, CDCl$_3$): δ 7.15 (dd, J = 8.5, 7.4 Hz, 2H), 6.65 (t, J = 7.3 Hz, 1H), 6.54 (d, J = 7.6 Hz, 2H), 3.49 (dt, J = 8.0, 4.1 Hz, 2H), 1.89 (td, J = 10.8, 8.9, 3.7 Hz, 2H), 1.76 – 1.69 (m, 2H), 1.62 – 1.51 (m, 10H). **^{13}C NMR (101 MHz, CDCl$_3$):** δ 147.3, 129.3, 116.8, 113.4, 52.6, 32.7, 27.2, 26.0, 24.1. **HRMS (ESI-TOF):** calculated for $C_{14}H_{22}N$ $[M+H]^+$: 204.1747, found: 204.1748.

Compound 36

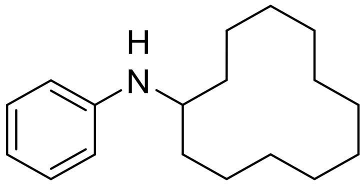

Following **Procedure E** on 0.20 mmol scale. Purification by PTLC (petroleum ether/ethyl acetate = 10:1) afforded 27.0 mg (52%).

Physical State: colorless oil.

^{1}H NMR (400 MHz, CDCl$_3$): δ 7.18 – 7.12 (m, 2H), 6.65 (t, J = 7.3 Hz, 1H), 6.57 (d, J = 7.7 Hz, 2H), 3.50 (dt, J = 7.0, 3.0 Hz, 2H), 1.64 – 1.58 (m, 2H), 1.43 – 1.33 (m, 22H). **^{13}C NMR (101 MHz, CDCl$_3$):** δ 147.8, 129.3, 116.8, 113.1, 49.5, 29.8, 24.4, 24.0, 23.4, 23.3, 21.3. **HRMS (ESI-TOF):** calculated for $C_{18}H_{30}N$ $[M+H]^+$: 260.2373, found: 260.2372.

Compound 37

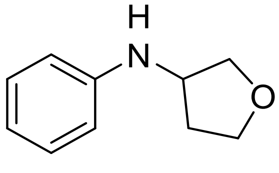

Following **Procedure E** on 0.20 mmol scale. Purification by PTLC (petroleum ether/ethyl acetate = 10:1) afforded 15.9 mg (49%).

Physical State: colorless oil.

^{1}H NMR (400 MHz, CDCl$_3$): δ 7.18 (dd, J = 8.5, 7.4 Hz, 2H), 6.73 (t, J = 7.3 Hz, 1H), 6.59 (d, J = 7.7 Hz, 2H), 4.11 – 4.06 (m, 1H), 3.97 – 3.92 (m, 2H), 3.86 – 3.81 (m, 1H), 3.70 (dd, J = 9.1, 3.0 Hz, 2H), 2.29 – 2.21 (m, 1H), 1.90 – 1.83 (m, 1H). **^{13}C NMR (101 MHz, CDCl$_3$):** δ 147.1, 129.4, 117.8, 113.4, 73.8, 67.1, 53.9, 33.3. **HRMS (ESI-TOF):** calculated for $C_{10}H_{14}NO$ [M+H]$^+$: 164.1070, found: 164.1072.

Compound 38

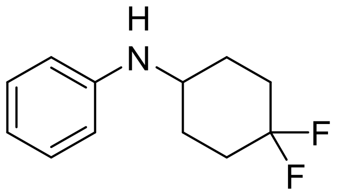

Following **Procedure E** on 0.20 mmol scale. Purification by PTLC (petroleum ether/ethyl acetate = 10:1) afforded 19.8 mg (47%).

Physical State: colorless oil.

^{1}H NMR (400 MHz, CDCl$_3$): δ 7.19 – 7.13 (m, 2H), 6.69 (t, J = 7.3 Hz, 1H), 6.57 (d, J = 7.7 Hz, 2H), 3.38 (td, J = 10.2, 9.7, 5.9 Hz, 2H), 2.13 – 2.02 (m, 4H), 1.84 (ddt, J = 21.2, 15.3, 8.8 Hz, 2H), 1.52 (q, J = 11.6, 10.4 Hz, 2H). **^{13}C NMR (101 MHz, CDCl$_3$):** δ 147.0, 129.5, 123.0 (t, J = 239 Hz), 117.6, 113.3, 49.6, 32.1 (t, J = 25 Hz), 28.9 (d, J = 5 Hz). **^{19}F NMR (376 MHz, CDCl$_3$):** δ -95.19 (d, J = 236.3 Hz), -99.78 (d, J = 241.6 Hz). **HRMS (ESI-TOF):** calculated for $C_{12}H_{16}F_2N$ [M+H]$^+$: 212.1245, found: 212.1246.

Compound 39

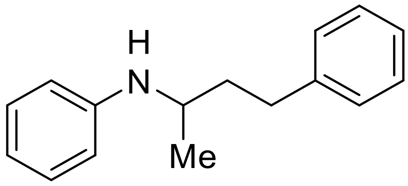

Following **Procedure E** on 0.20 mmol scale. Purification by PTLC (petroleum ether/ethyl acetate = 10:1) afforded 22.9 mg (51%).

Physical State: colorless oil.

^{1}H NMR (400 MHz, CDCl$_3$): δ 7.27 (td, J = 6.7, 1.8 Hz, 2H), 7.21 – 7.10 (m, 5H), 6.66 (t, J = 7.3 Hz, 1H), 6.56 – 6.52 (m, 2H), 3.49 (h, J = 6.3 Hz, 2H), 2.72 (t, J = 7.9 Hz, 2H), 1.93 – 1.84 (m, 1H), 1.81 – 1.73 (m, 1H), 1.21 (d, J = 6.3 Hz, 3H). **^{13}C NMR (101 MHz, CDCl$_3$):** δ 147.4, 142.0, 129.3, 128.4, 128.4, 125.9, 117.1, 113.3, 48.0, 38.8, 32.5, 20.8. **HRMS (ESI-TOF):** calculated for $C_{16}H_{20}N$ $[M+H]^+$: 226.1590, found: 226.1592.

Compound 40

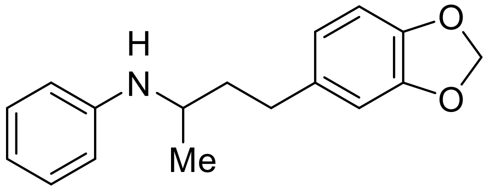

Following **Procedure E** on 0.20 mmol scale. Purification by PTLC (petroleum ether/ethyl acetate = 10:1) afforded 25.3 mg (47%).

Physical State: colorless oil.

^{1}H NMR (400 MHz, CDCl$_3$): δ 7.14 (dd, J = 8.5, 7.4 Hz, 2H), 6.71 (d, J = 7.9 Hz, 1H), 6.69 – 6.63 (m, 2H), 6.60 (dd, J = 7.9, 1.7 Hz, 1H), 6.54 – 6.51 (m, 2H), 5.89 (s, 2H), 3.46 (q, J = 6.3 Hz, 1H), 2.63 (t, J = 7.8 Hz, 2H), 1.81 (dd, J = 14.2, 6.6 Hz, 1H), 1.74 – 1.67 (m, 1H), 1.19 (d, J = 6.3 Hz, 3H). **^{13}C NMR (101 MHz, CDCl$_3$):** δ 147.6, 147.6, 145.7, 135.9, 129.4, 121.2, 117.0, 113.3, 108.9, 108.2, 100.8, 47.9, 39.1, 32.3, 20.9. **HRMS (ESI-TOF):** calculated for $C_{17}H_{20}NO_2$ $[M+H]^+$: 270.1489, found: 270.1488.

Compound 41

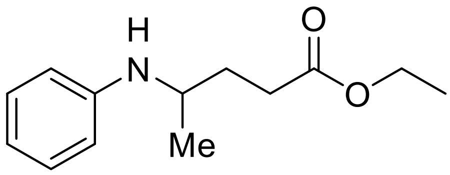

Following **Procedure E** on 0.20 mmol scale. Purification by PTLC (petroleum

ether/ethyl acetate = 10:1) afforded 19.5 mg (44%).

Physical State: colorless oil.

^{1}H NMR (400 MHz, CDCl$_3$): δ 7.18 – 7.11 (m, 2H), 6.65 (t, J = 7.8 Hz, 1H), 6.59 – 6.54 (m, 2H), 4.15 – 4.07 (m, 2H), 3.52 (h, J = 6.3 Hz, 2H), 2.41 (t, J = 7.4 Hz, 2H), 1.87 – 1.79 (m, 2H), 1.22 (t, J = 7.1 Hz, 3H), 1.18 (d, J = 6.3 Hz, 3H). **^{13}C NMR (101 MHz, CDCl$_3$):** δ 173.8, 147.5, 129.3, 117.0, 113.1, 60.4, 48.1, 32.0, 31.2, 20.9, 14.2. **HRMS (ESI-TOF):** calculated for $C_{13}H_{20}NO_2$ [M+H]$^+$: 222.1489, found: 222.1488.

Compound 42

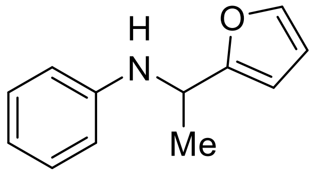

Following **Procedure E** on 0.20 mmol scale. Purification by PTLC (petroleum ether/ethyl acetate = 10:1) afforded 11.6 mg (31%).

Physical State: colorless oil.

^{1}H NMR (400 MHz, CDCl$_3$): 7.32 (dd, J = 1.8, 0.8 Hz, 1H), 7.17 – 7.12 (m, 2H), 6.70 (tt, J = 7.4, 1.0 Hz, 1H), 6.62 (dd, J = 8.6, 1.0 Hz, 2H), 6.27 (dd, J = 3.2, 1.8 Hz, 1H), 6.14 (dt, J = 3.2, 0.7 Hz, 1H), 4.66 – 4.60 (m, 1H), 3.80 (s, 1H), 1.54 (d, J = 6.7 Hz, 3H). **^{13}C NMR (101 MHz, CDCl$_3$):** δ 157.2, 147.0, 141.5, 129.2, 117.8, 113.5, 110.1, 105.1, 47.4, 20.9. **HRMS (ESI-TOF):** calculated for $C_{12}H_{14}NO$ [M+H]$^+$: 188.1070, found: 188.1073.

Compound 43

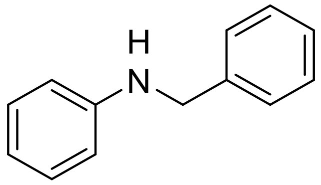

Following **Procedure E** on 0.20 mmol scale. Purification by PTLC (petroleum ether/ethyl acetate = 10:1) afforded 15.0 mg (41%).

Physical State: colorless oil.

^{1}H NMR (400 MHz, CDCl$_3$): δ 7.31 (q, J = 7.5 Hz, 4H), 7.25 (d, J = 8.0 Hz, 1H), 7.14 (t, J = 7.9 Hz, 2H), 6.70 (d, J = 7.3 Hz, 1H), 6.59 (d, J = 8.4 Hz, 2H), 4.27 (s, 2H), 3.74 (s, 1H). **^{13}C NMR (101 MHz, CDCl$_3$):** δ 148.3, 139.6, 129.4, 128.8, 127.6, 127.4, 117.7, 113.0, 48.4. **HRMS (ESI-TOF):** calculated for $C_{13}H_{14}N$ $[M+H]^+$: 184.1121, found: 184.1124.

Compound 44

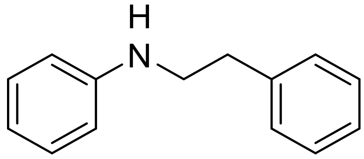

Following **Procedure E** on 0.20 mmol scale. Purification by PTLC (petroleum ether/ethyl acetate = 10:1) afforded 13.8 mg (35%).

Physical State: colorless oil.

^{1}H NMR (400 MHz, CDCl$_3$): δ 7.31 (t, J = 7.4 Hz, 2H), 7.24 – 7.20 (m, 3H), 7.20 – 7.15 (m, 2H), 6.70 (t, J = 7.2 Hz, 1H), 6.65 – 6.56 (m, 2H), 3.40 (t, J = 7.0 Hz, 3H), 2.91 (t, J = 7.0 Hz, 2H). **^{13}C NMR (101 MHz, CDCl$_3$):** δ 148.0, 139.3, 129.3, 128.8, 128.6, 126.4, 117.5, 113.0, 45.0, 35.5. **HRMS (ESI-TOF):** calculated for $C_{14}H_{16}N$ $[M+H]^+$: 198.1277, found: 198.1275.

Compound 45

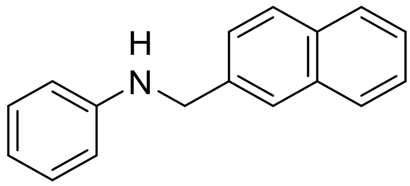

Following **Procedure E** on 0.20 mmol scale. Purification by PTLC (petroleum ether/ethyl acetate = 10:1) afforded 17.2 mg (37%).

Physical State: colorless oil.

^{1}H NMR (400 MHz, CDCl$_3$): δ 7.80 – 7.71 (m, 4H), 7.45 – 7.36 (m, 3H), 7.18 – 7.09 (m, 2H), 6.70 (t, J = 7.3 Hz, 1H), 6.63 – 6.58 (m, 2H), 4.38 (s, 2H), 3.90 (s, 1H). **^{13}C NMR (101 MHz, CDCl$_3$):** δ 137.1, 133.6, 132.9, 129.4, 128.5, 127., 127.9, 126.3,

126.1, 125.9, 117.8, 113.1, 48.6. **HRMS (ESI-TOF):** calculated for $C_{17}H_{16}N$ $[M+H]^+$: 234.1277, found: 234.1276.

Compound 46

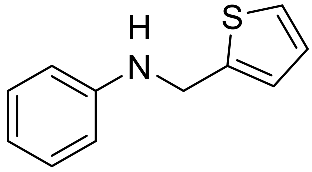

Following **Procedure E** on 0.20 mmol scale. Purification by PTLC (petroleum ether/ethyl acetate = 10:1) afforded 10.2 mg (27%).

Physical State: colorless oil.

^{1}H NMR (400 MHz, $CDCl_3$): δ 7.19 – 7.11 (m, 3H), 6.96 (dd, J = 3.4, 1.1 Hz, 1H), 6.92 (dd, J = 5.0, 3.5 Hz, 1H), 6.74 – 6.69 (m, 1H), 6.62 (dd, J = 8.6, 1.0 Hz, 2H), 4.43 – 4.42 (m, 2H), 3.89 (s, 1H). **^{13}C NMR (101 MHz, $CDCl_3$):** δ 147.5, 142.9, 129.2, 126.8, 125.0, 124.5, 118.1, 113.2, 43.4. **HRMS (ESI-TOF):** calculated for $C_{11}H_{12}NS$ $[M+H]^+$: 190.0685, found: 190.0687.

Compound 47

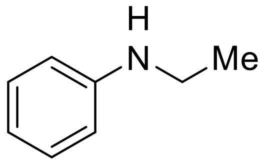

Following **Procedure E** on 0.20 mmol scale. Purification by PTLC (petroleum ether/ethyl acetate = 10:1) afforded 4.1 mg (17%).

Physical State: colorless oil.

^{1}H NMR (400 MHz, $CDCl_3$): δ 7.21 – 7.11 (m, 2H), 6.68 (t, J = 7.3 Hz, 1H), 6.61 – 6.56 (m, 2H), 3.40 (s, 1H), 3.11 (t, J = 7.1 Hz, 2H), 1.22 (t, J = 7.1 Hz, 3H). **^{13}C NMR (101 MHz, $CDCl_3$):** δ 148.5, 129.3, 117.3, 112.9, 77.5, 77.2, 76.9, 38.6, 14.9. **HRMS (ESI-TOF):** calculated for $C_8H_{12}N$ $[M+H]^+$: 122.0964, found: 122.0967.

Compound 48

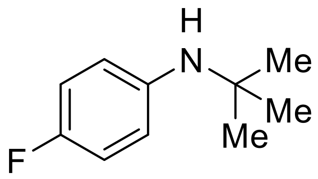

Following **Procedure E** on 0.20 mmol scale. Purification by PTLC (petroleum ether/ethyl acetate = 10:1) afforded 24.4 mg (73%).

Physical State: colorless oil.

^{1}H NMR (400 MHz, $CDCl_3$): δ 6.88 (t, J = 8.7 Hz, 2H), 6.75 (dd, J = 8.8, 4.7 Hz, 2H), 2.97 (s, 1H), 1.27 (s, 9H). **^{13}C NMR (101 MHz, $CDCl_3$):** δ 157.4(d, J = 237 Hz), 120. 9(d, J = 8 Hz), 115.4, 115.2, 52.1, 30.1. **^{19}F NMR (376 MHz, $CDCl_3$):** δ -125.16. **HRMS (ESI-TOF):** calculated for $C_{10}H_{15}FN$ $[M+H]^+$: 168.1183, found: 168.1184.

Compound 49

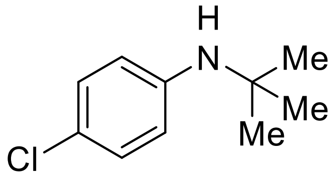

Following **Procedure E** on 0.20 mmol scale. Purification by PTLC (petroleum ether/ethyl acetate = 10:1) afforded 24.9 mg (68%).

Physical State: colorless oil.

^{1}H NMR (400 MHz, $CDCl_3$): δ 7.09 (d, J = 8.9 Hz, 2H), 6.66 (d, J = 8.9 Hz, 2H), 3.29 (s, 1H), 1.31 (s, 9H). **^{13}C NMR (101 MHz, $CDCl_3$):** δ 145.4, 128.8, 123.1, 118.5, 51.6, 30.0. **HRMS (ESI-TOF):** calculated for $C_{10}H_{15}ClN$ $[M+H]^+$: 184.0888, found: 184.0889.

Compound 50

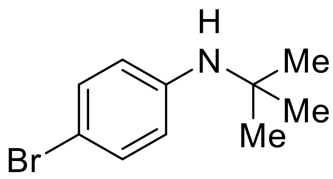

Following **Procedure E** on 0.20 mmol scale. Purification by PTLC (petroleum ether/ethyl acetate = 10:1) afforded 27.2 mg (60%).

Physical State: colorless oil.

^{1}H NMR (400 MHz, CDCl$_3$): δ 7.25 – 7.20 (m, 2H), 6.63 – 6.60 (m, 2H), 3.39 (s, 1H), 1.32 (s, 9H). **^{13}C NMR (101 MHz, CDCl$_3$):** δ 145.8, 131.7, 118.7, 110.2, 51.6, 30.0. **HRMS (ESI-TOF):** calculated for $C_{10}H_{15}BrN$ $[M+H]^+$: 228.0382, found: 228.0381.

Compound 51

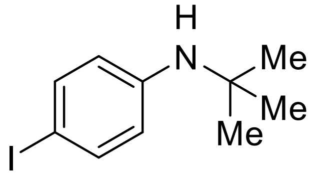

Following **Procedure E** on 0.20 mmol scale. Purification by PTLC (petroleum ether/ethyl acetate = 10:1) afforded 30.3 mg (55%).

Physical State: colorless oil.

^{1}H NMR (400 MHz, CDCl$_3$): δ 7.42 – 7.36 (m, 2H), 6.52 – 6.49 (m, 2H), 3.30 (s, 1H), 1.32 (s, 9H). **^{13}C NMR (101 MHz, CDCl$_3$):** δ 146. 5, 137.6, 125.5, 118.9, 51.5, 29.9. **HRMS (ESI-TOF):** calculated for $C_{10}H_{15}IN$ $[M+H]^+$: 276.0244, found: 276.0241.

Compound 52

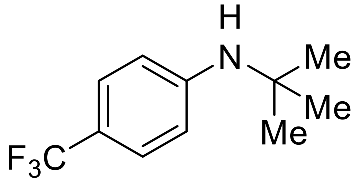

Following **Procedure E** on 0.20 mmol scale. Purification by PTLC (petroleum ether/ethyl acetate = 10:1) afforded 27.8 mg (64%).

Physical State: colorless oil.

^{1}H NMR (400 MHz, CDCl$_3$): δ 7.35 (d, J = 8.5 Hz, 2H), 6.68 (d, J = 8.5 Hz, 2H), 3.94 (s, 1H), 1.38 (s, 9H). **^{13}C NMR (101 MHz, CDCl$_3$):** δ 149.6, 126.3(q, J = 4 Hz), 125.1(q, J = 268 Hz), 118.4 (q, J = 32 Hz), 114.3, 51.2, 29.7. **^{19}F NMR (376 MHz, CDCl$_3$)** δ -61.06. **HRMS (ESI-TOF):** calculated for $C_{11}H_{15}F_3N$ $[M+H]^+$: 218.1151, found: 218.1153.

Compound 53

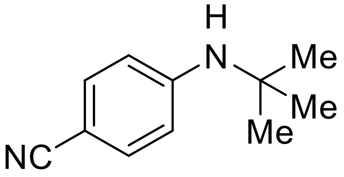

Following **Procedure E** on 0.20 mmol scale. Purification by PTLC (petroleum ether/ethyl acetate = 10:1) afforded 21.2 mg (61%).

Physical State: colorless oil.

^{1}H NMR (400 MHz, $CDCl_3$): δ 7.39 – 7.35 (m, 2H), 6.64 – 6.61 (m, 2H), 4.25 (s, 1H), 1.40 (s, 9H). **^{13}C NMR (101 MHz, $CDCl_3$):** δ 150.3, 133.4, 120.6, 114.2, 98.0, 51.4, 29.5. **HRMS (ESI-TOF):** calculated for $C_{11}H_{15}N_2$ $[M+H]^+$: 175.1230, found: 175.1231.

Compound 54

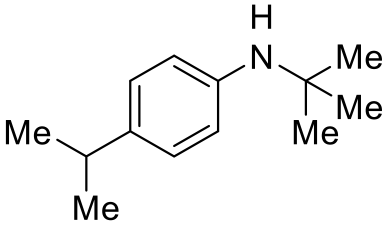

Following **Procedure E** on 0.20 mmol scale. Purification by PTLC (petroleum ether/ethyl acetate = 10:1) afforded 18.0 mg (47%).

Physical State: colorless oil.

^{1}H NMR (400 MHz, $CDCl_3$): δ 7.02 (d, J = 8.3 Hz, 2H), 6.71 (d, J = 8.5 Hz, 2H), 3.14 (s, 1H), 1.42 (s, 1H), 1.30 (s, 9H), 1.21 (d, J = 6.9 Hz, 6H). **^{13}C NMR (101 MHz, $CDCl_3$):** δ 144.4, 139.4, 126.7, 118.5, 51.7, 33.2, 30.1, 24.2. **HRMS (ESI-TOF):** calculated for $C_{13}H_{22}N$ $[M+H]^+$: 192.1747, found: 192.1749.

Compound 55

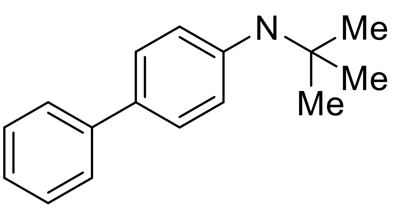

Following **Procedure E** on 0.20 mmol scale. Purification by PTLC (petroleum ether/ethyl acetate = 10:1) afforded 26.1 mg (58%).

Physical State: colorless oil.

^{1}H NMR (400 MHz, CDCl$_3$): δ 7.54 (dd, J = 8.2, 1.1 Hz, 2H), 7.43 – 7.36 (m, 4H), 7.26 (d, J = 8.2 Hz, 1H), 6.81 (d, J = 8.6 Hz, 2H), 3.15 (s, 1H), 1.38 (s, 9H). **^{13}C NMR (101 MHz, CDCl$_3$):** δ 146.2, 141.2, 131.0, 128.6, 127.6, 126.3, 126.1, 117.3, 51.6, 30.0. **HRMS (ESI-TOF):** calculated for $C_{16}H_{20}N$ $[M+H]^+$: 226.1590, found: 226.1591.

Compound 56

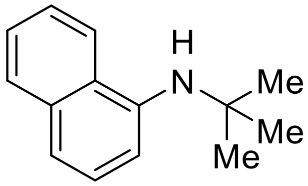

Following **Procedure E** on 0.20 mmol scale. Purification by PTLC (petroleum ether/ethyl acetate = 10:1) afforded 27.1 mg (68%).

Physical State: colorless oil.

^{1}H NMR (400 MHz, CDCl$_3$): δ 7.83 – 7.75 (m, 2H), 7.46 – 7.37 (m, 2H), 7.32 (t, J = 7.8 Hz, 1H), 7.25 (d, J = 9.8 Hz, 1H), 6.94 (dd, J = 7.5, 1.2 Hz, 1H), 3.70 (s, 1H), 1.47 (s, 9H). **^{13}C NMR (101 MHz, CDCl$_3$):** δ 141.8, 134.7, 128.8, 126.1, 125.5, 124.7, 120.4, 117.9, 110.1, 51.7, 30.0. **HRMS (ESI-TOF):** calculated for $C_{14}H_{18}N$ $[M+H]^+$: 200.1434, found: 200.1437.

Compound 57

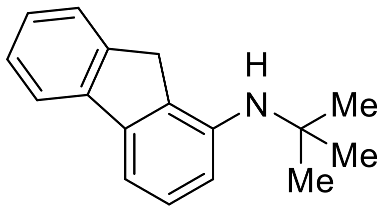

Following **Procedure E** on 0.20 mmol scale. Purification by PTLC (petroleum ether/ethyl acetate = 10:1) afforded 27.0 mg (57%).

Physical State: colorless oil.

^{1}**H NMR (400 MHz, CDCl$_3$):** δ 7.62 (d, J = 7.5 Hz, 1H), 7.56 (d, J = 8.2 Hz, 1H), 7.45 (d, J = 7.4 Hz, 1H), 7.30 (t, J = 7.4 Hz, 1H), 7.17 (t, J = 7.4 Hz, 1H), 6.97 (s, 1H), 6.78 (d, J = 8.0 Hz, 1H), 3.81 (s, 2H), 2.82 (s, 1H), 1.37 (s, 9H). ^{13}**C NMR (101 MHz, CDCl$_3$):** δ 144.6, 142.4, 126.6, 125.1, 124.7, 120.2, 118.6, 117.0, 114.4, 52.0, 37.0, 30.2. **HRMS (ESI-TOF):** calculated for $C_{17}H_{20}N$ [M+H]$^+$: 238.1590, found: 238.1592.

Compound 58

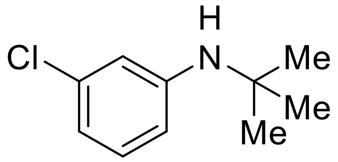

Following **Procedure E** on 0.20 mmol scale. Purification by PTLC (petroleum ether/ethyl acetate = 10:1) afforded 18.7 mg (51%).

Physical State: colorless oil.

^{1}**H NMR (400 MHz, CDCl$_3$):** δ 7.04 (t, J = 8.0 Hz, 1H), 6.70 (t, J = 2.1 Hz, 1H), 6.67 (dd, J = 7.4, 1.5 Hz, 1H), 6.57 (ddd, J = 8.2, 2.2, 0.8 Hz, 1H), 3.24 (s, 1H), 1.34 (s, 9H). ^{13}**C NMR (101 MHz, CDCl$_3$):** δ 148.1, 134.6, 129.9, 117.5, 116.0, 114.7, 51.5, 29.9. **HRMS (ESI-TOF):** calculated for $C_{10}H_{15}ClN$ [M+H]$^+$: 184.0888, found: 184.0886.

Compound 59

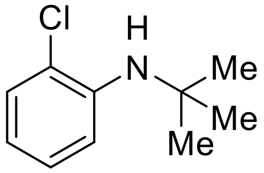

Following **Procedure E** on 0.20 mmol scale. Purification by PTLC (petroleum ether/ethyl acetate = 10:1) afforded 22.7 mg (62%).

Physical State: colorless oil.

^{1}**H NMR (400 MHz, CDCl$_3$):** δ 7.24 (dd, J = 7.9, 1.6 Hz, 1H), 7.08 (ddd, J = 8.8, 7.3, 1.6 Hz, 1H), 6.96 (dd, J = 8.3, 1.5 Hz, 1H), 6.60 (td, J = 7.8, 1.5 Hz, 1H), 4.29 (s, 1H), 1.39 (s, 9H). ^{13}**C NMR (101 MHz, CDCl$_3$):** δ 142.9, 129.4, 127.2, 121.0, 117.3, 115.0,

51.4, 29.9. **HRMS (ESI-TOF):** calculated for $C_{10}H_{15}ClN$ $[M+H]^+$: 184.0888, found: 184.0886.

Compound 60

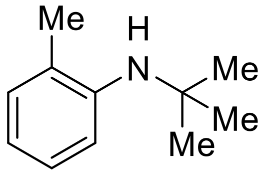

Following **Procedure E** on 0.20 mmol scale. Purification by PTLC (petroleum ether/ethyl acetate = 10:1) afforded 18.9 mg (58%).

Physical State: colorless oil.

^{1}H NMR (400 MHz, CDCl$_3$): δ 7.11 – 7.03 (m, 2H), 6.91 (d, J = 8.0 Hz, 1H), 6.65 (t, J = 7.0 Hz, 1H), 3.19 (s, 1H), 2.12 (s, 3H), 1.39 (s, 9H). **^{13}C NMR (101 MHz, CDCl$_3$):** δ 144.9, 130.5, 126.6, 123.8, 117.2, 114.4, 51.4, 30.2, 18.2. **HRMS (ESI-TOF):** calculated for $C_{11}H_{18}N$ $[M+H]^+$: 164.1434, found:164.1435.

Compound 61

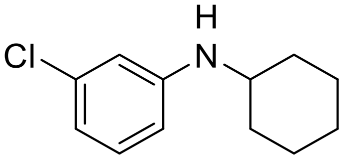

Following **Procedure E** on 0.20 mmol scale. Purification by PTLC (petroleum ether/ethyl acetate = 10:1) afforded 22.6 mg (54%).

Physical State: colorless oil.

^{1}H NMR (400 MHz, CDCl$_3$): δ 6.96 (t, J = 8.0 Hz, 1H), 6.53 (ddd, J = 7.9, 2.0, 0.9 Hz, 1H), 6.47 (t, J = 2.1 Hz, 1H), 6.35 (ddd, J = 8.2, 2.3, 0.8 Hz, 1H), 3.52 (s, 1H), 3.14 (tt, J = 10.1, 3.7 Hz, 1H), 1.95 (dd, J = 13.1, 3.1 Hz, 2H), 1.68 (dt, J = 13.1, 3.7 Hz, 2H), 1.57 (dt, J = 12.7, 3.8 Hz, 1H), 1.33 – 1.25 (m, 2H), 1.20 – 1.14 (m, 1H), 1.10 – 1.01 (m, 2H). **^{13}C NMR (101 MHz, CDCl$_3$):** δ 147.5, 134.0, 129.1, 115.5, 111.4, 110.4, 50.5, 32.3, 24.8, 23.9. **HRMS (ESI-TOF):** calculated for $C_{12}H_{17}ClN$ $[M+H]^+$: 210.1044, found: 210.1046.

Compound 62

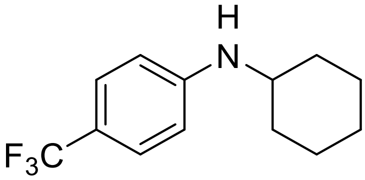

Following **Procedure E** on 0.20 mmol scale. Purification by PTLC (petroleum ether/ethyl acetate = 10:1) afforded 26.2 mg (52%).

Physical State: colorless oil.

^{1}H NMR (400 MHz, CDCl$_3$): δ 7.36 (d, J = 8.4 Hz, 2H), 6.56 (d, J = 8.4 Hz, 2H), 3.86 (s, 1H), 3.33 – 3.24 (m, 1H), 2.04 (d, J = 10.2 Hz, 2H), 1.80 – 1.73 (m, 2H), 1.70 – 1.63 (m, 1H), 1.42 – 1.33 (m, 2H), 1.28 – 1.23 (m, 1H), 1.22 – 1.15 (m, 2H). **^{13}C NMR (101 MHz, CDCl$_3$):** δ 149.8, 126.6 (q, J = 4 Hz), 125.1 (q, J = 268 Hz), 118.1 (q, J = 27 Hz), 112.0, 51.3, 33.2, 25.8, 24.9. **^{19}F NMR (376 MHz, Chloroform-d):** δ -60.90. **HRMS (ESI-TOF):** calculated for $C_{13}H_{17}F_3N$ [M+H]$^+$: 244.1380, found: 244.1382.

Compound 63

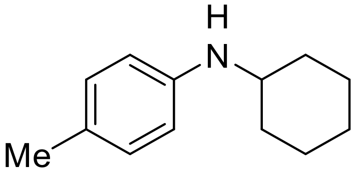

Following **Procedure E** on 0.20 mmol scale. Purification by PTLC (petroleum ether/ethyl acetate = 10:1) afforded 18.5 mg (49%).

Physical State: colorless oil.

^{1}H NMR (400 MHz, CDCl$_3$): δ 6.96 (d, J = 8.0 Hz, 2H), 6.52 (d, J = 8.4 Hz, 2H), 3.21 (tt, J = 10.1, 3.7 Hz, 2H), 2.22 (s, 3H), 2.04 (dd, J = 12.8, 3.2 Hz, 2H), 1.75 (dt, J = 13.0, 3.7 Hz, 2H), 1.64 (dt, J = 12.6, 3.8 Hz, 1H), 1.35 (ddd, J = 16.1, 10.9, 3.3 Hz, 2H), 1.27 – 1.23 (m, 1H), 1.16 – 1.09 (m, 2H). **^{13}C NMR (101 MHz, CDCl$_3$):** δ 145.1, 129.7, 126.1, 113.5, 52.1, 33.6, 26.0, 25.1, 20.4. **HRMS (ESI-TOF):** calculated for $C_{13}H_{20}N$ [M+H]$^+$: 190.1590, found: 190.1592.

Compound 64

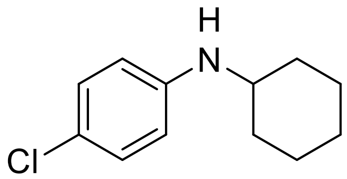

Following **Procedure E** on 0.20 mmol scale. Purification by PTLC (petroleum ether/ethyl acetate = 10:1) afforded 25.9 mg (62%).

Physical State: colorless oil.

^{1}H NMR (400 MHz, CDCl$_3$): δ 7.15 – 7.03 (m, 2H), 6.52 – 6.49 (m, 2H), 3.62 (s, 1H), 3.20 (ddt, J = 10.2, 6.4, 3.8 Hz, 1H), 2.02 (dd, J = 9.3, 4.2 Hz, 2H), 1.78 – 1.73 (m, 2H), 1.68 – 1.63 (m, 1H), 1.39 – 1.33 (m, 2H), 1.28 – 1.24 (m, 1H), 1.16 – 1.11 (m, 2H). **^{13}C NMR (101 MHz, CDCl$_3$):** δ 145.9, 129.1, 121.1, 114.3, 51.9, 33.3, 25.9, 25.0. **HRMS (ESI-TOF):** calculated for $C_{12}H_{17}ClN$ [M+H]$^+$: 210.1044, found: 210.1046.

Compound 65

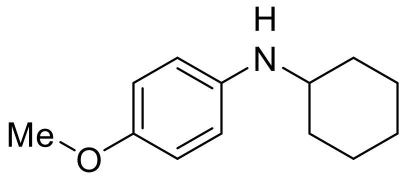

Following **Procedure E** on 0.20 mmol scale. Purification by PTLC (petroleum ether/ethyl acetate = 10:1) afforded 12.7 mg (31%).

Physical State: colorless oil.

^{1}H NMR (400 MHz, CDCl$_3$): δ 6.82 – 6.78 (m, 2H), 6.63 – 6.59 (m, 2H), 3.77 (s, 3H), 3.20 (tt, J = 10.2, 3.7 Hz, 2H), 2.08 (dd, J = 12.8, 3.1 Hz, 2H), 1.79 (dt, J = 13.3, 3.6 Hz, 2H), 1.68 (dt, J = 12.7, 3.7 Hz, 1H), 1.44 – 1.33 (m, 2H), 1.26 (tt, J = 12.1, 3.4 Hz, 1H), 1.19 – 1.11 (m, 2H). **^{13}C NMR (101 MHz, CDCl$_3$):** δ 151.9, 141.6, 114.9, 114.9, 55.8, 52.8, 33.6, 26.0, 25.1. **HRMS (ESI-TOF):** calculated for $C_{13}H_{20}NO$ [M+H]$^+$: 206.1539, found: 206.1538.

Compound 66

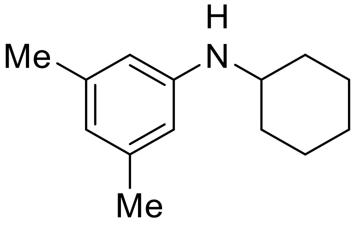

Following **Procedure E** on 0.20 mmol scale. Purification by PTLC (petroleum ether/ethyl acetate = 10:1) afforded 21.1 mg (52%).

Physical State: colorless oil.

^{1}H NMR (400 MHz, CDCl$_3$): δ 6.38 (s, 1H), 6.28 (s, 2H), 3.27 (ddd, J = 13.9, 10.2, 3.7 Hz, 2H), 2.27 (s, 6H), 2.08 (dq, J = 12.6, 3.8 Hz, 2H), 1.79 (dt, J = 13.3, 3.7 Hz, 2H), 1.68 (dt, J = 12.7, 3.7 Hz, 1H), 1.44 – 1.36 (m, 2H), 1.27 (ddd, J = 15.4, 8.0, 3.6 Hz, 1H), 1.21 – 1.14 (m, 2H). **^{13}C NMR (101 MHz, CDCl$_3$):** δ 147.2, 138.9, 119.1, 111.2, 51.8, 33.5, 26.0, 25.1, 21.6. **HRMS (ESI-TOF):** calculated for $C_{14}H_{22}N$ [M+H]$^+$: 204.1747, found: 204.1748.

Compound 67

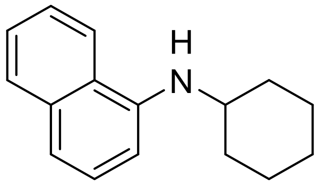

Following **Procedure E** on 0.20 mmol scale. Purification by PTLC (petroleum ether/ethyl acetate = 10:1) afforded 24.8 mg (55%).

Physical State: colorless oil.

^{1}H NMR (400 MHz, CDCl$_3$): δ 7.82 – 7.74 (m, 2H), 7.42 (qt, J = 7.0, 3.3 Hz, 2H), 7.32 (t, J = 7.9 Hz, 1H), 7.18 (d, J = 8.1 Hz, 1H), 6.67 – 6.61 (m, 1H), 4.28 (s, 1H), 3.46 (td, J = 9.9, 4.9 Hz, 1H), 2.18 (dd, J = 12.8, 3.0 Hz, 2H), 1.84 – 1.78 (m, 2H), 1.72 – 1.66 (m, 1H), 1.47 – 1.40 (m, 2H), 1.35 – 1.28 (m, 3H). **^{13}C NMR (101 MHz, CDCl$_3$):** δ142.3, 134.6, 128.7, 126.6, 125.6, 124.5, 123.4, 119.8, 116.7, 104.7, 51.8, 33.3, 26.1, 25.1. **HRMS (ESI-TOF):** calculated for $C_{16}H_{20}N$ [M+H]$^+$: 226.1590, found: 226.1591.

Compound 68

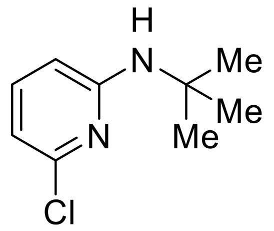

Following **Procedure E** on 0.20 mmol scale. Purification by PTLC (petroleum ether/ethyl acetate = 10:1) afforded 24.7 mg (67%).

Physical State: colorless oil.

^{1}H NMR (400 MHz, $CDCl_3$): δ 7.31 – 7.26 (m, 1H), 6.51 (d, J = 7.5 Hz, 1H), 6.31 (d, J = 8.3 Hz, 1H), 4.65 (s, 1H), 1.41 (s, 9H). **^{13}C NMR (101 MHz, $CDCl_3$):** δ 158.1, 149.3, 139.1, 111.2, 106.1, 50.9, 29.3. **HRMS (ESI-TOF):** calculated for $C_9H_{13}ClN_2$ $[M+H]^+$: 185.0840, found: 185.0843.

Compound 69

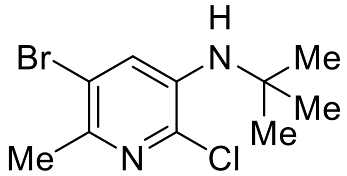

Following **Procedure E** on 0.20 mmol scale. Purification by PTLC (petroleum ether/ethyl acetate = 10:1) afforded 26.5 mg (48%).

Physical State: colorless oil.

^{1}H NMR (400 MHz, $CDCl_3$): δ 7.31 (s, 1H), 4.25 (s, 1H), 2.48 (s, 3H), 1.39 (s, 9H). **^{13}C NMR (101 MHz, $CDCl_3$):** δ 143.1, 138.3, 136.0, 124.1, 119.7, 51.6, 29.5, 23.0. **HRMS (ESI-TOF):** calculated for $C_{10}H_{15}BrClN_2$ $[M+H]^+$: 277.0102, found: 277.0104.

Compound 70

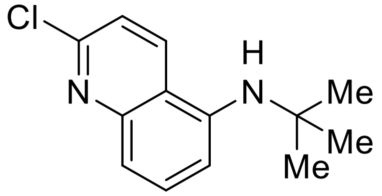

Following **Procedure E** on 0.20 mmol scale. Purification by PTLC (petroleum ether/ethyl acetate = 10:1) afforded 25.9 mg (55%).

Physical State: colorless oil.

^{1}H NMR (400 MHz, CDCl$_3$): δ 8.11 (d, J = 9.3 Hz, 1H), 7.55 (t, J = 8.1 Hz, 1H), 7.41 (d, J = 8.4 Hz, 1H), 7.28 – 7.25 (m, 1H), 6.95 (dd, J = 7.8, 0.8 Hz, 1H), 3.92 (s, 1H), 1.46 (s, 10H). **^{13}C NMR (101 MHz, CDCl$_3$):** δ 150.2, 149.3, 142.2, 132.5, 131.2, 120.1, 119.0, 118.1, 110.7, 52.0, 29.8. **HRMS (ESI-TOF):** calculated for $C_{13}H_{16}ClN_2$ [M+H]$^+$: 235.0997, found: 235.0998.

Compound 71

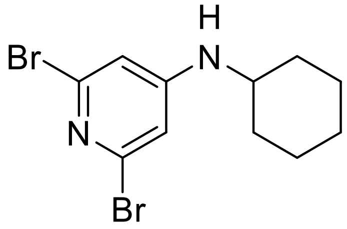

Following **Procedure E** on 0.20 mmol scale. Purification by PTLC (petroleum ether/ethyl acetate = 3:1) afforded 27.1 mg (41%).

Physical State: colorless oil.

^{1}H NMR (400 MHz, CDCl$_3$): δ 6.57 (s, 2H), 4.46 (s, 1H), 3.30 – 3.22 (m, 1H), 2.03 – 1.99 (m, 2H), 1.79 (dd, J = 13.6, 3.7 Hz, 2H), 1.69 (dt, J = 12.8, 3.7 Hz, 1H), 1.43 – 1.35 (m, 2H), 1.27 – 1.19 (m, 3H). **^{13}C NMR (101 MHz, CDCl$_3$):** δ 155.1, 140.5, 109.9, 51.3, 32.7, 25.4, 24.6. **HRMS (ESI-TOF):** calculated for $C_{11}H_{15}Br_2N_2$ [M+H]$^+$: 332.9602, found: 332.9598.

Compound 72

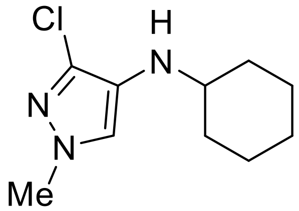

Following **Procedure E** on 0.20 mmol scale. Purification by PTLC (petroleum

ether/ethyl acetate = 2:1) afforded 12.4 mg (29%).

Physical State: colorless oil.

^{1}H NMR (400 MHz, $CDCl_3$): δ 7.17 (s, 1H), 3.76 (s, 3H), 2.97 – 2.85 (m, 1H), 2.51 (s, 1H), 1.99 (dd, J = 12.8, 3.3 Hz, 2H), 1.76 – 1.72 (m, 2H), 1.62 (dt, J = 12.7, 3.7 Hz, 1H), 1.28 (dd, J = 15.1, 10.4 Hz, 3H), 1.19 – 1.09 (m, 3H). **^{13}C NMR (101 MHz, $CDCl_3$):** δ 129.9, 127.4, 115.2, 56.0, 36.5, 33.8, 25.9, 24.9. **HRMS (ESI-TOF):** calculated for $C_{10}H_{17}ClN_3$ $[M+H]^+$: 214.1111, found: 214.1111.

9. NMR Spectra

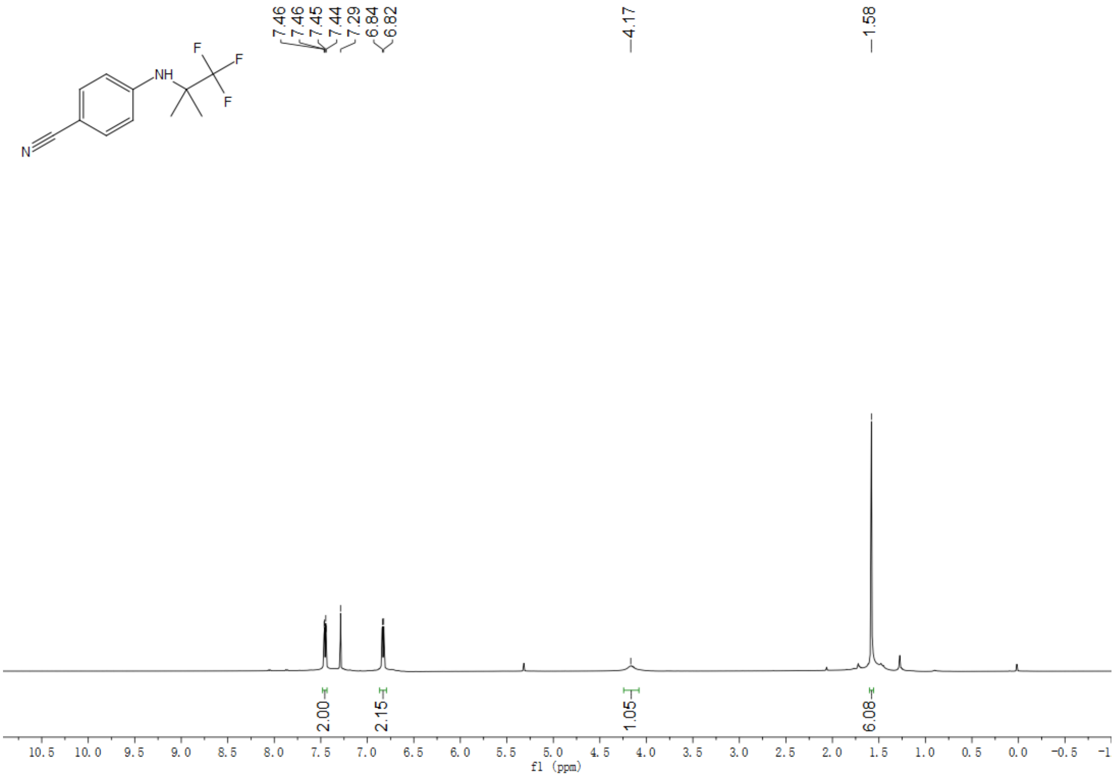

Supplementary Figure 6. ^{1}H NMR (400 MHz, room temperature, $CDCl_3$) spectra of product **3**

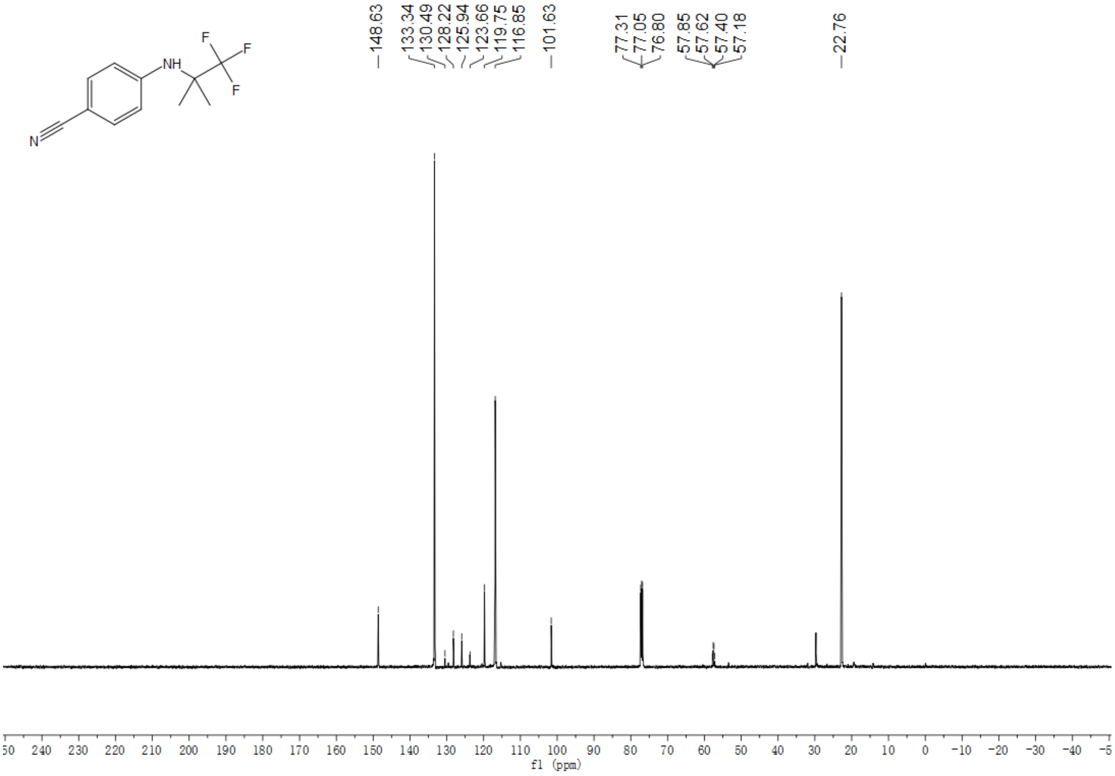

Supplementary Figure 7. ^{13}C NMR (101 MHz, room temperature, $CDCl_3$) spectra

of product **3**

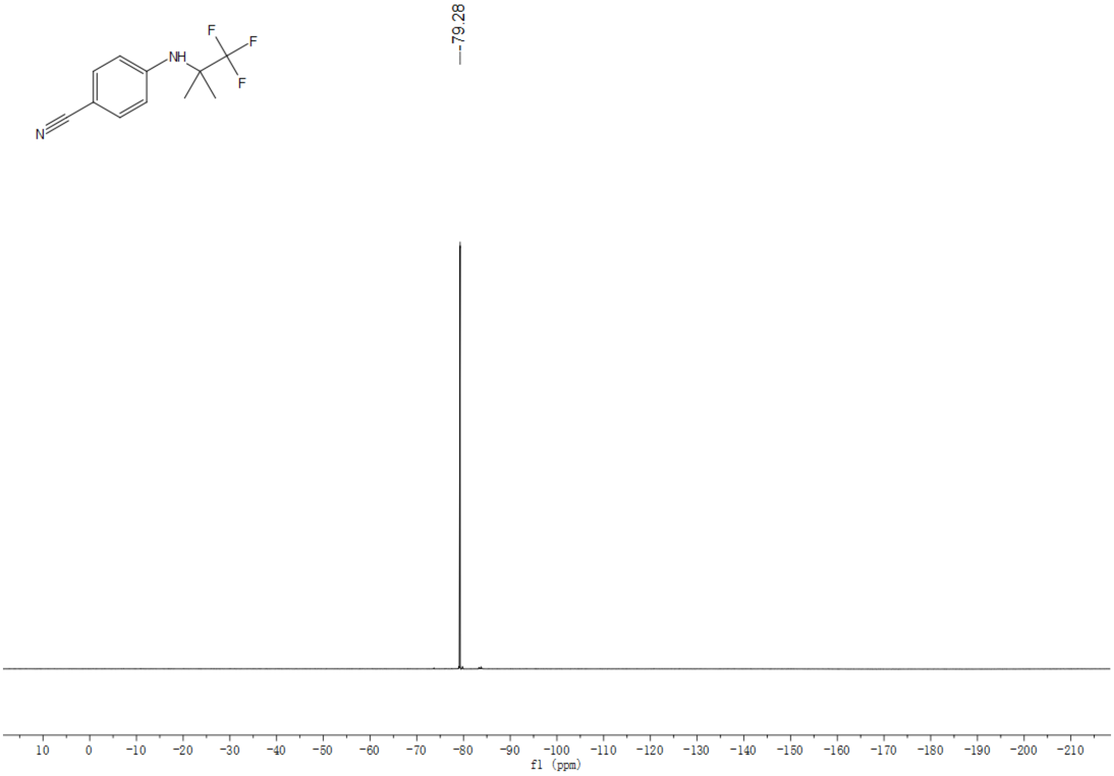

Supplementary Figure 8. ^{19}F NMR (376 MHz, room temperature, $CDCl_3$) spectra of product **3**

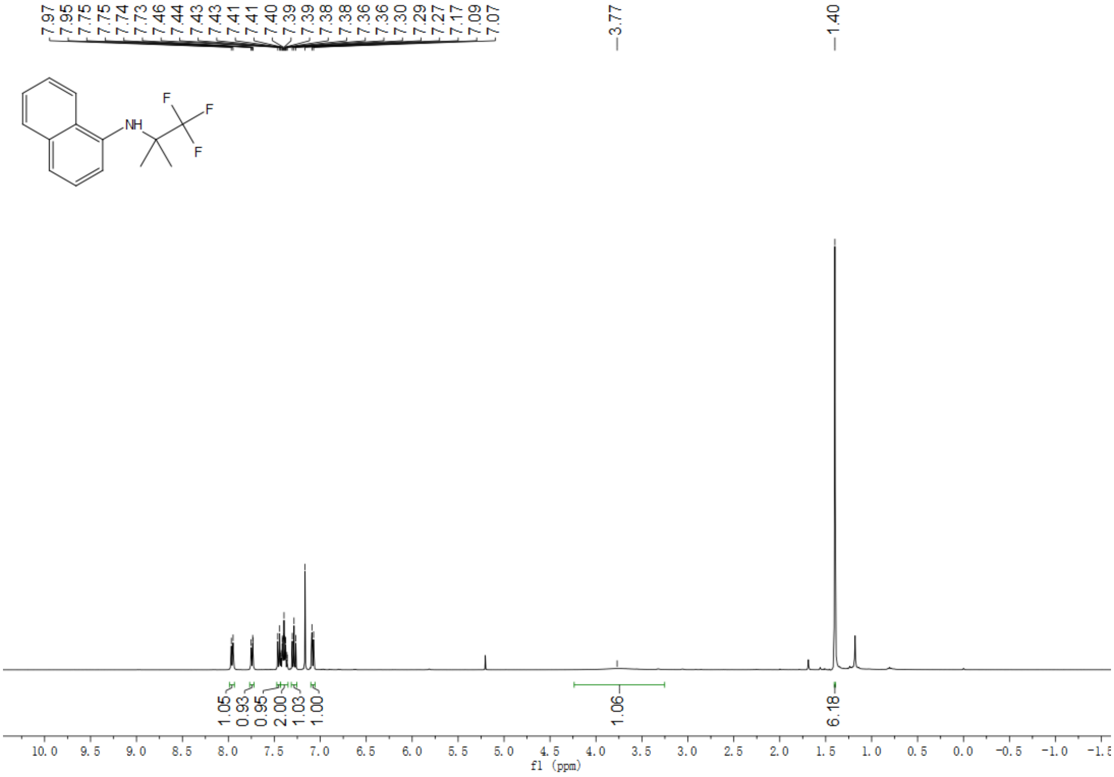

Supplementary Figure 9. ^{1}H NMR (400 MHz, room temperature, $CDCl_3$) spectra of product **4**

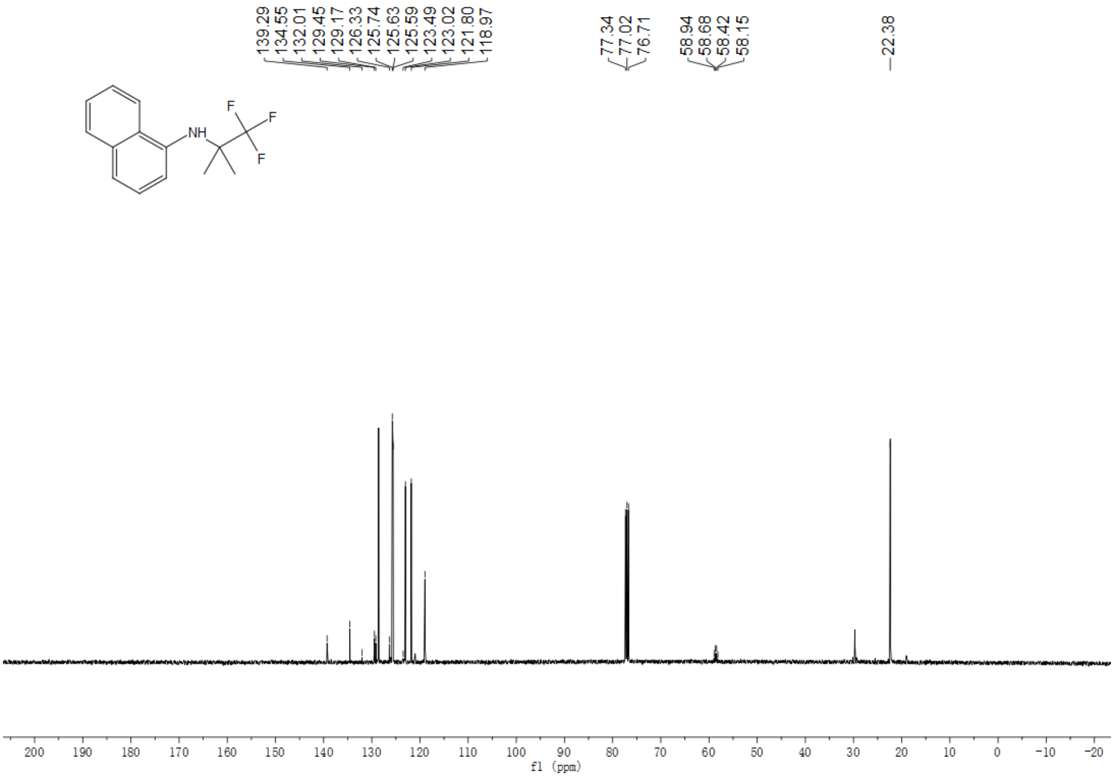

Supplementary Figure 10. ^{13}C NMR (101 MHz, room temperature, $CDCl_3$) spectra of product **4**

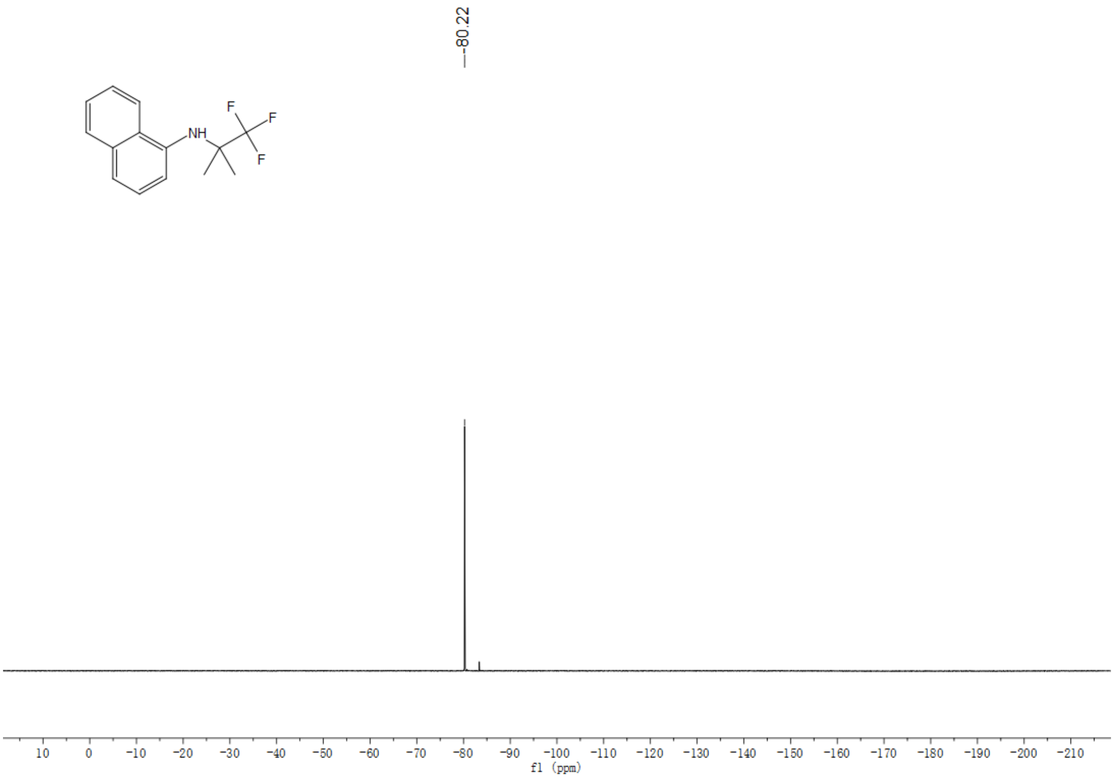

Supplementary Figure 11. ^{19}F NMR (376 MHz, room temperature, $CDCl_3$) spectra of product **4**

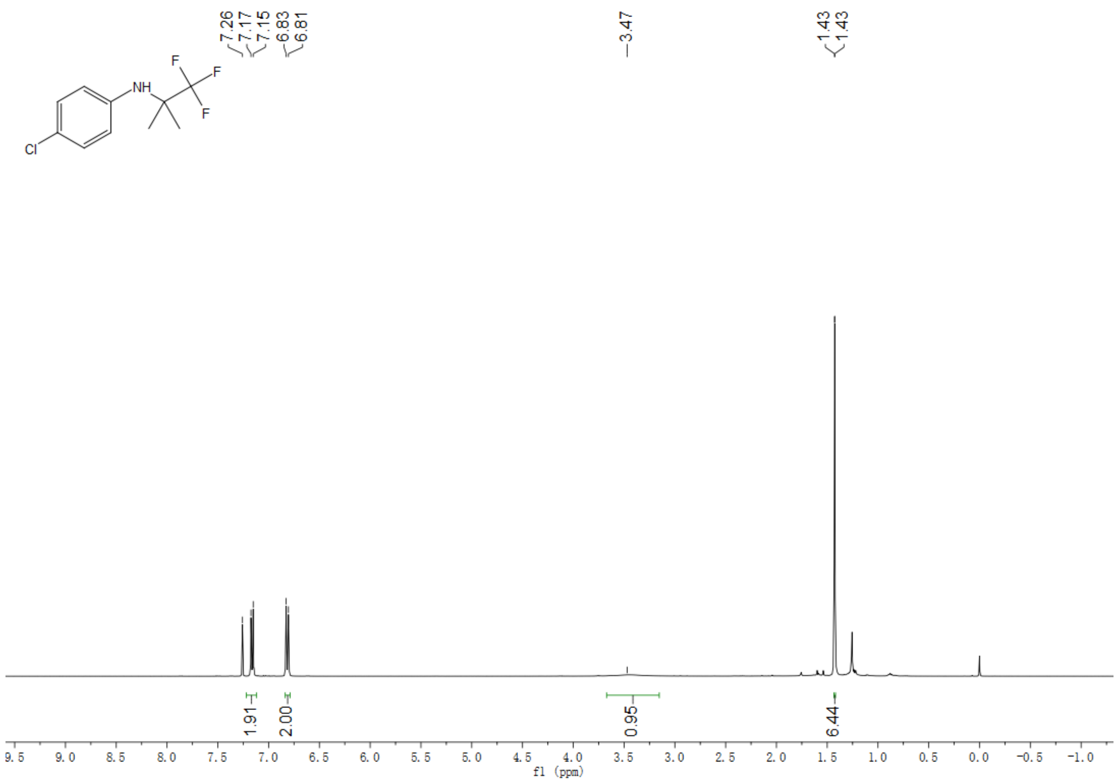

Supplementary Figure 12. ^{1}H NMR (400 MHz, room temperature, $CDCl_3$) spectra of product **5**

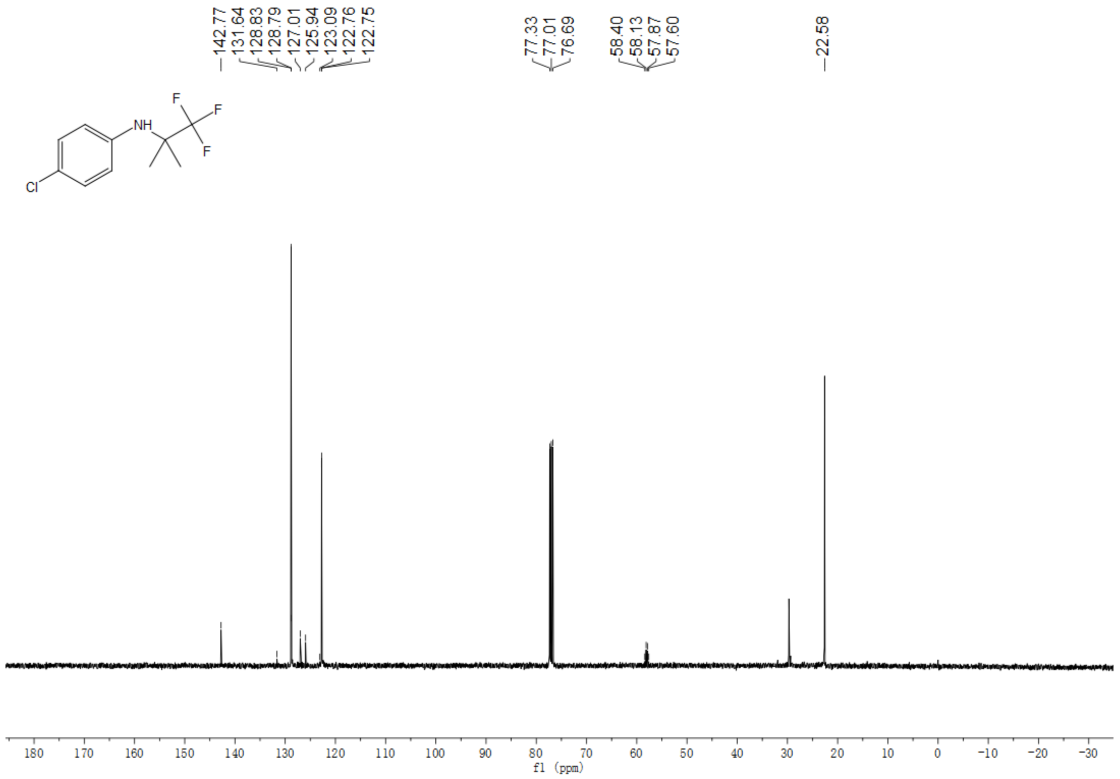

Supplementary Figure 13. ^{13}C NMR (101 MHz, room temperature, $CDCl_3$) spectra of product **5**

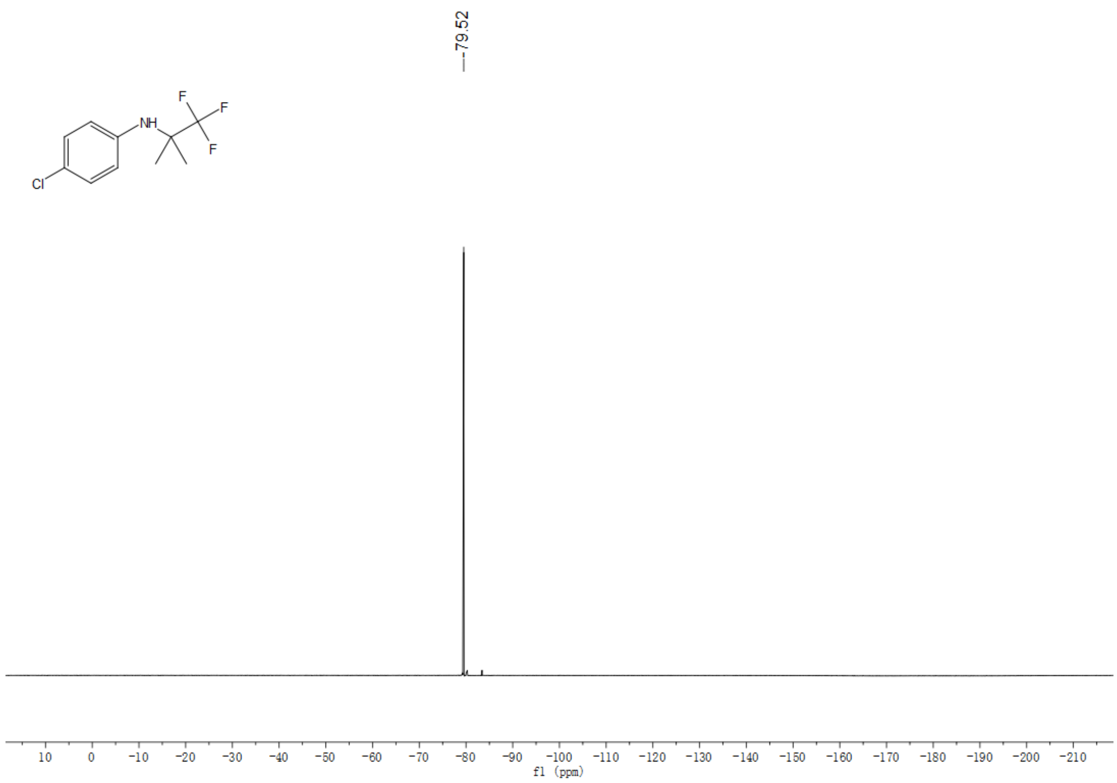

Supplementary Figure 14. ^{19}F NMR (376 MHz, room temperature, $CDCl_3$) spectra of product **5**

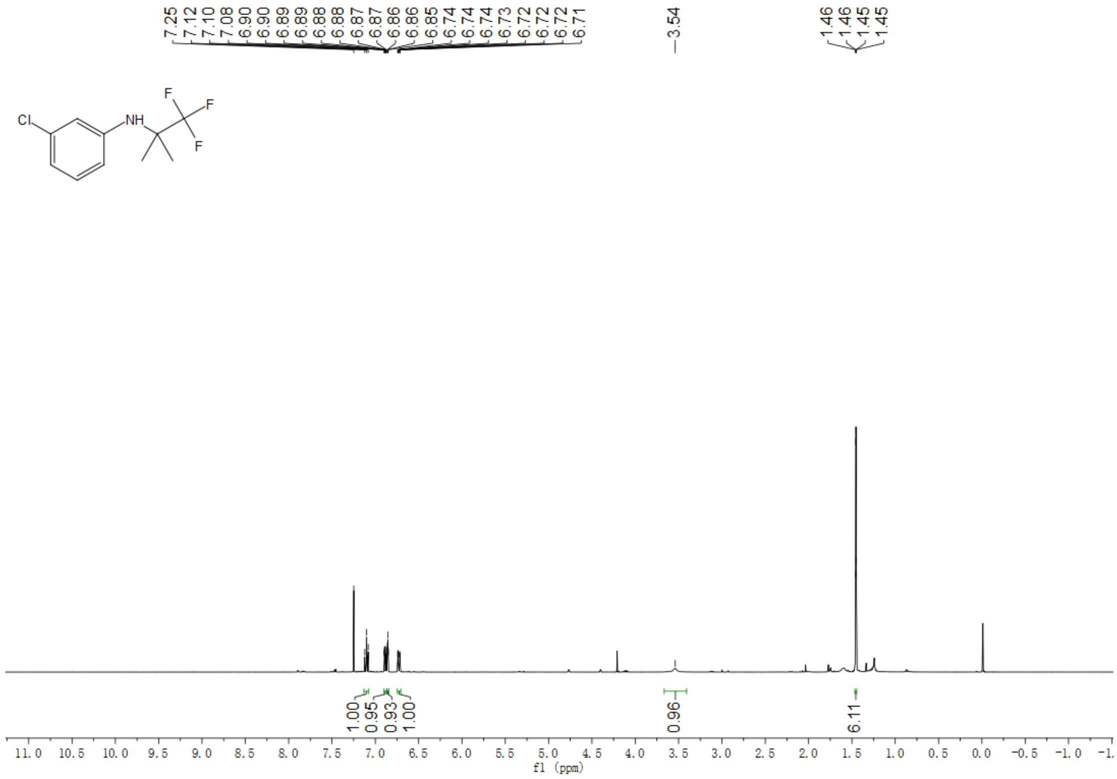

Supplementary Figure 15. ^{1}H NMR (400 MHz, room temperature, $CDCl_3$) spectra of product **6**

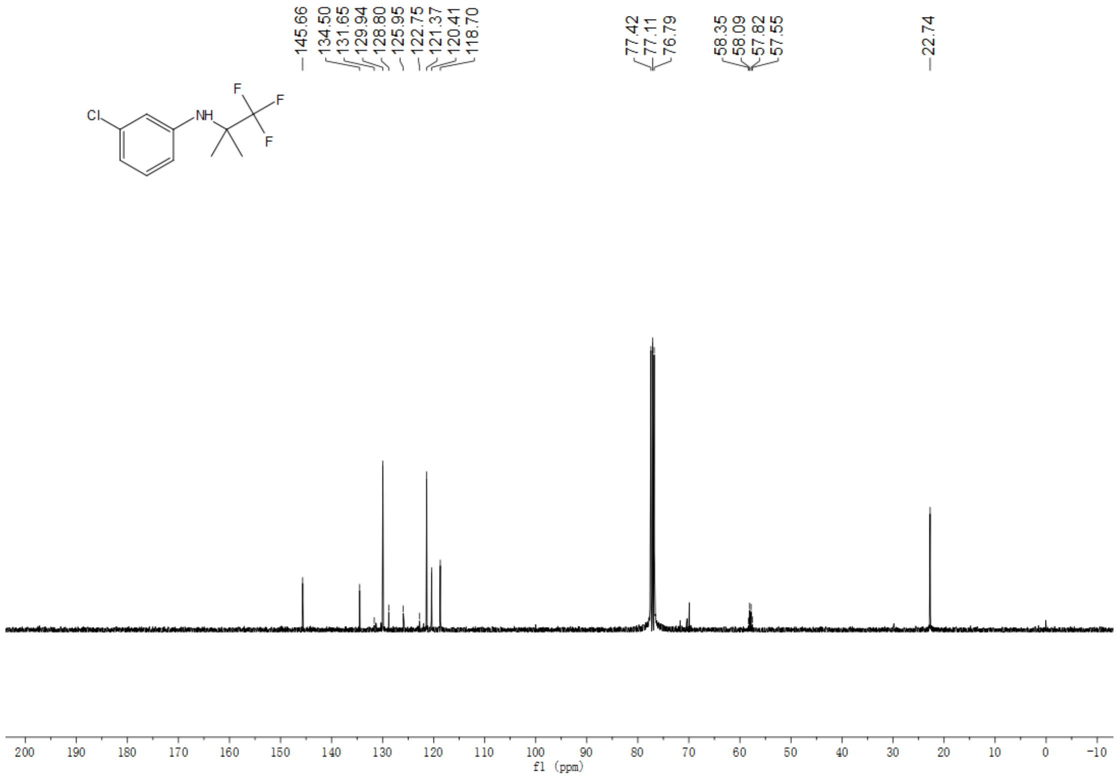

Supplementary Figure 16. ^{13}C NMR (101 MHz, room temperature, $CDCl_3$) spectra of product **6**

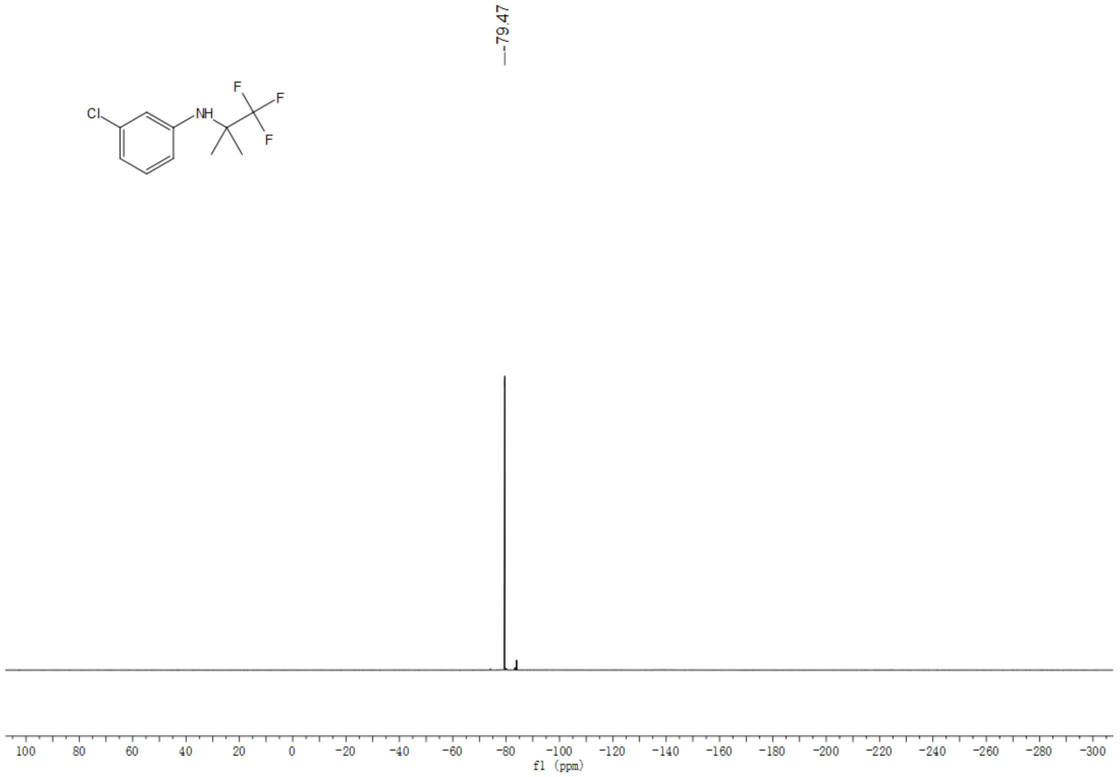

Supplementary Figure 17. ^{19}F NMR (376 MHz, room temperature, $CDCl_3$) spectra of product **6**

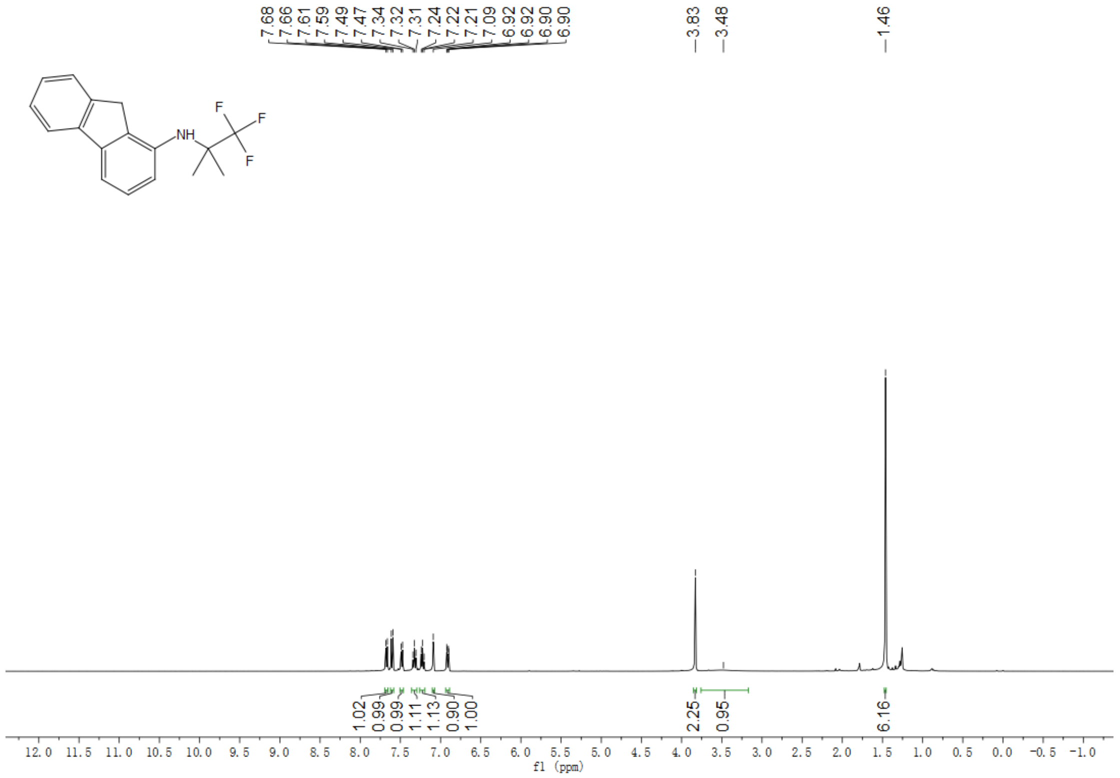

Supplementary Figure 18. ^{1}H NMR (400 MHz, room temperature, $CDCl_3$) spectra of product **7**

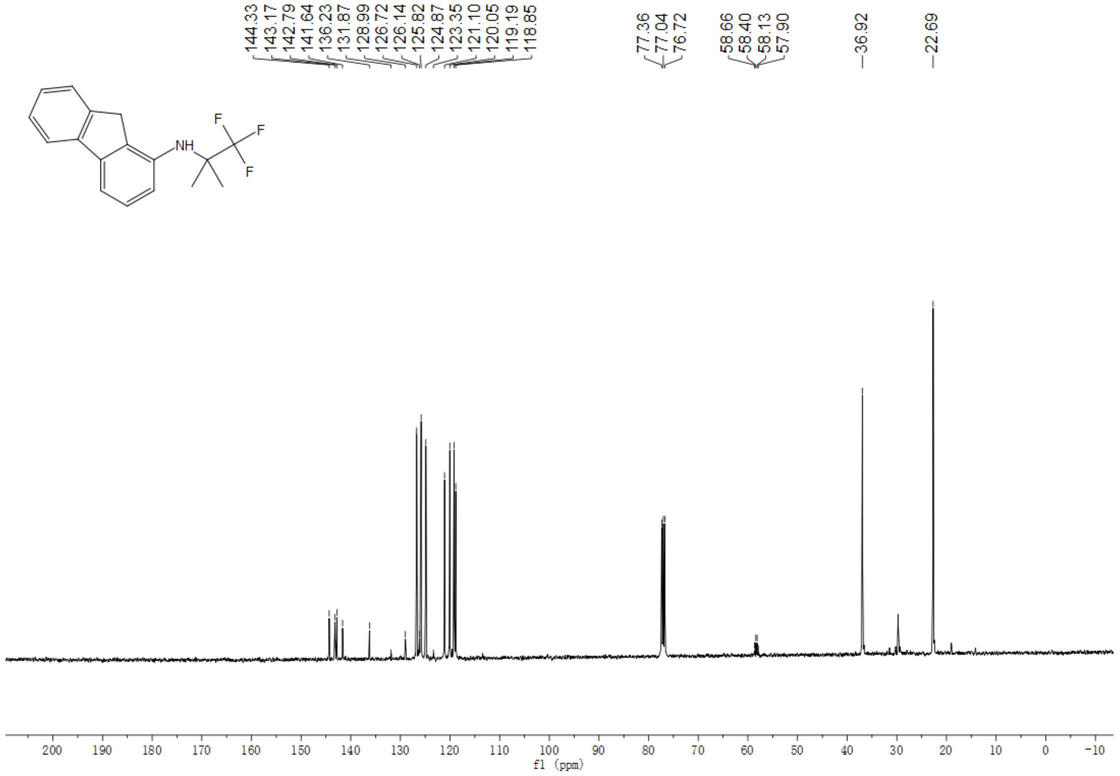

Supplementary Figure 19. ^{13}C NMR (101 MHz, room temperature, $CDCl_3$) spectra of product **7**

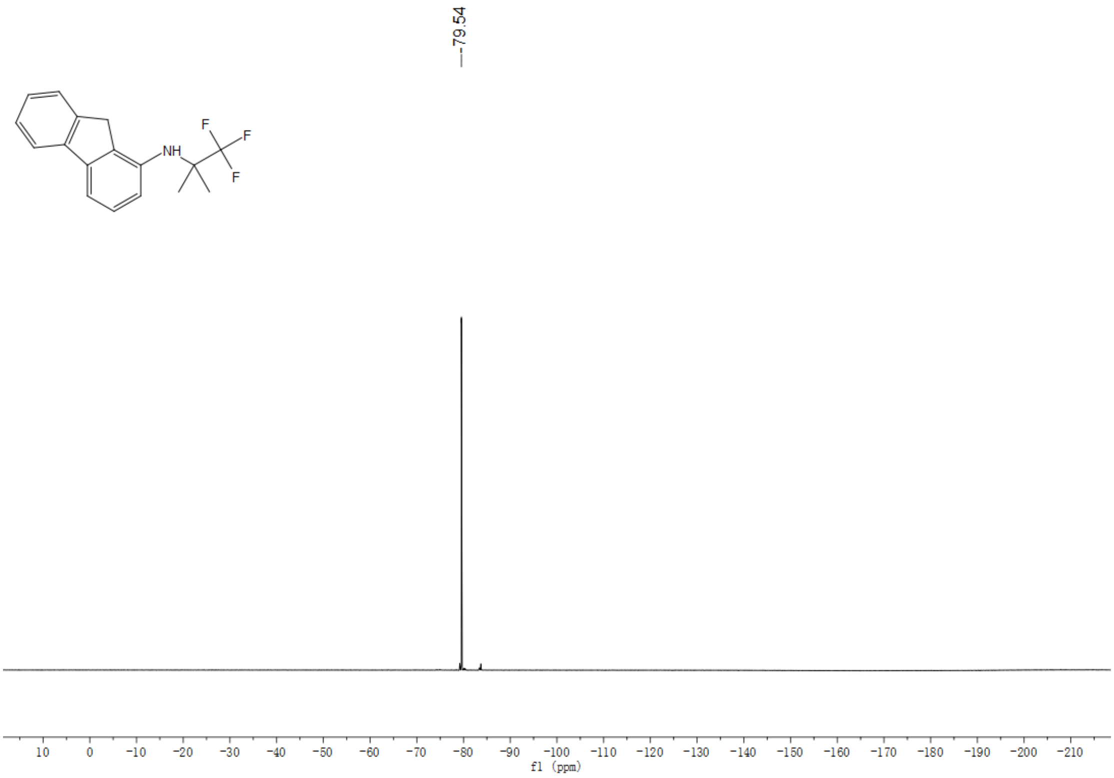

Supplementary Figure 20. ^{19}F NMR (376 MHz, room temperature, $CDCl_3$) spectra of product **7**

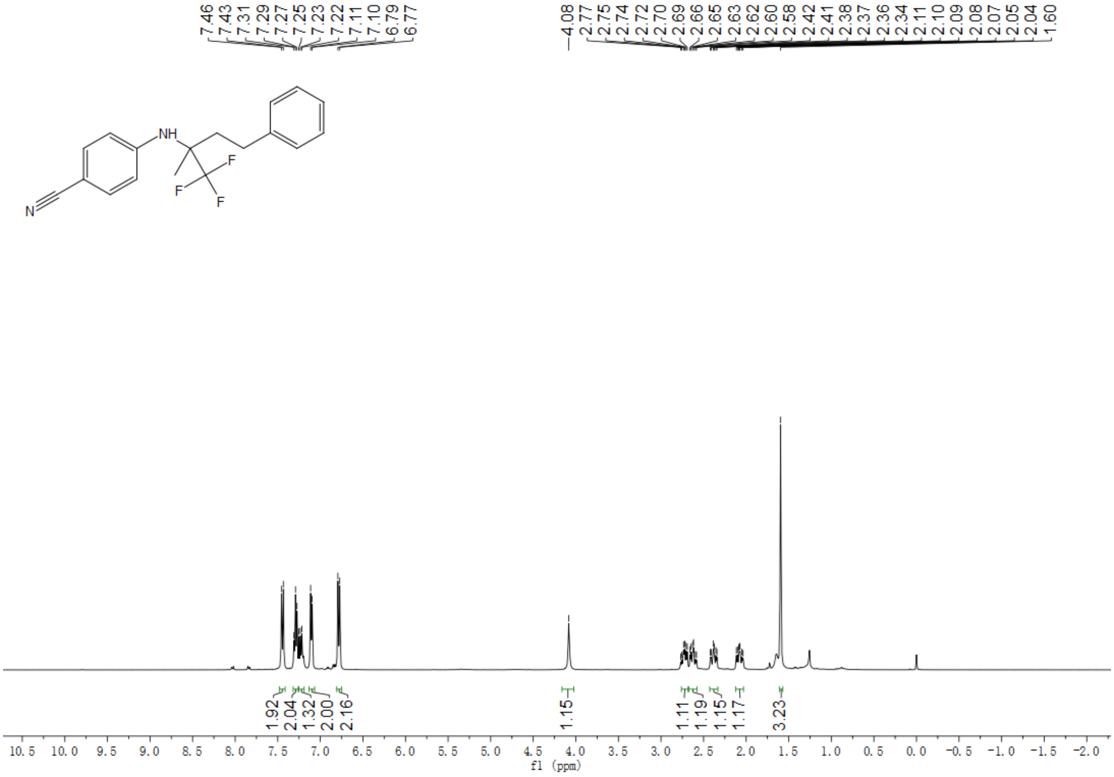

Supplementary Figure 21. ^{1}H NMR (400 MHz, room temperature, $CDCl_3$) spectra of product **8**

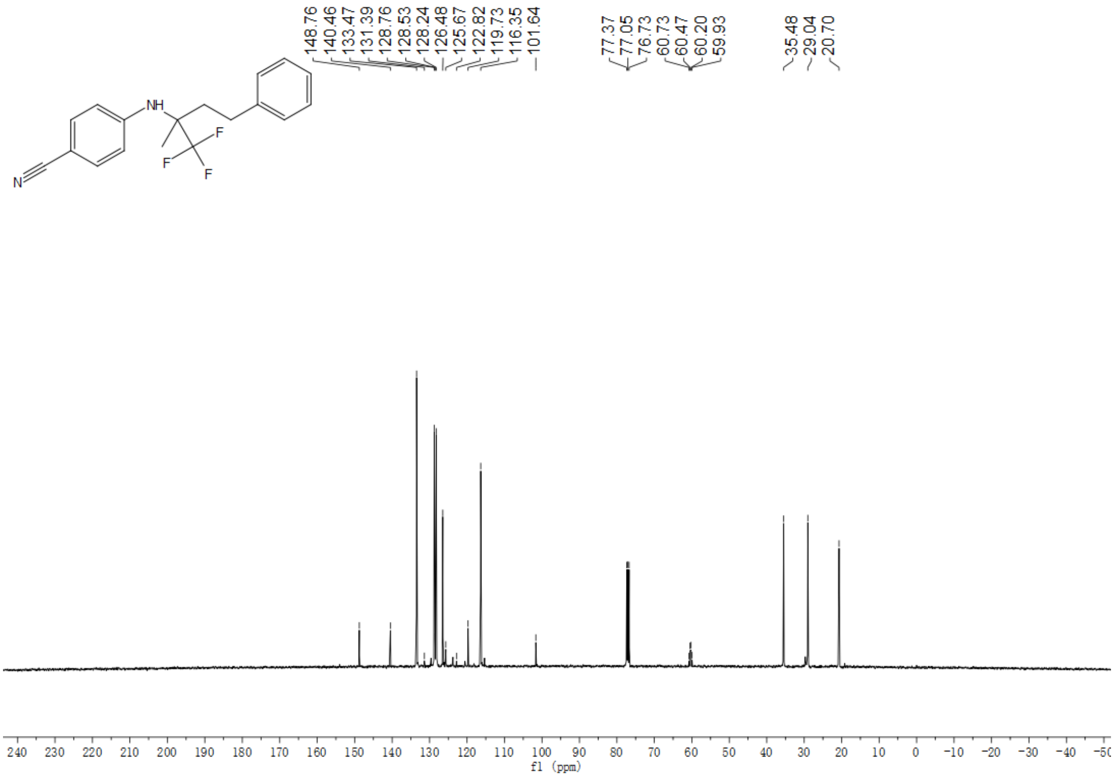

Supplementary Figure 22. ^{13}C NMR (101 MHz, room temperature, $CDCl_3$) spectra of product **8**

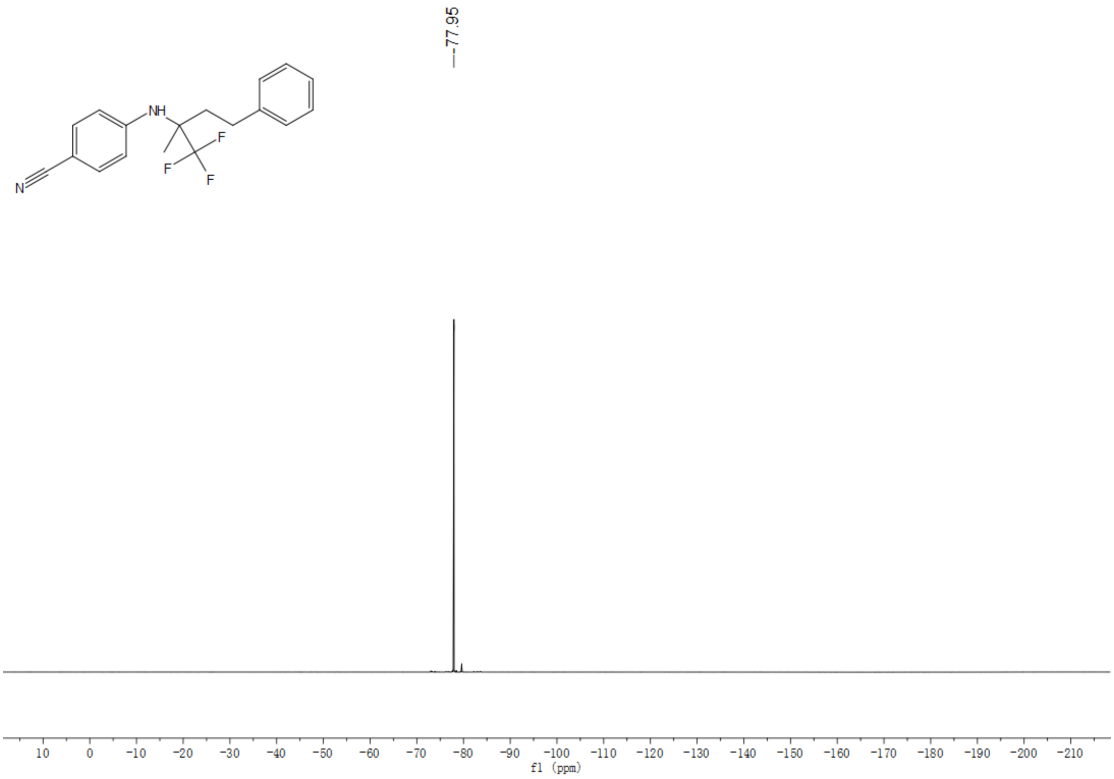

Supplementary Figure 23. ^{19}F NMR (376 MHz, room temperature, $CDCl_3$) spectra of product **8**

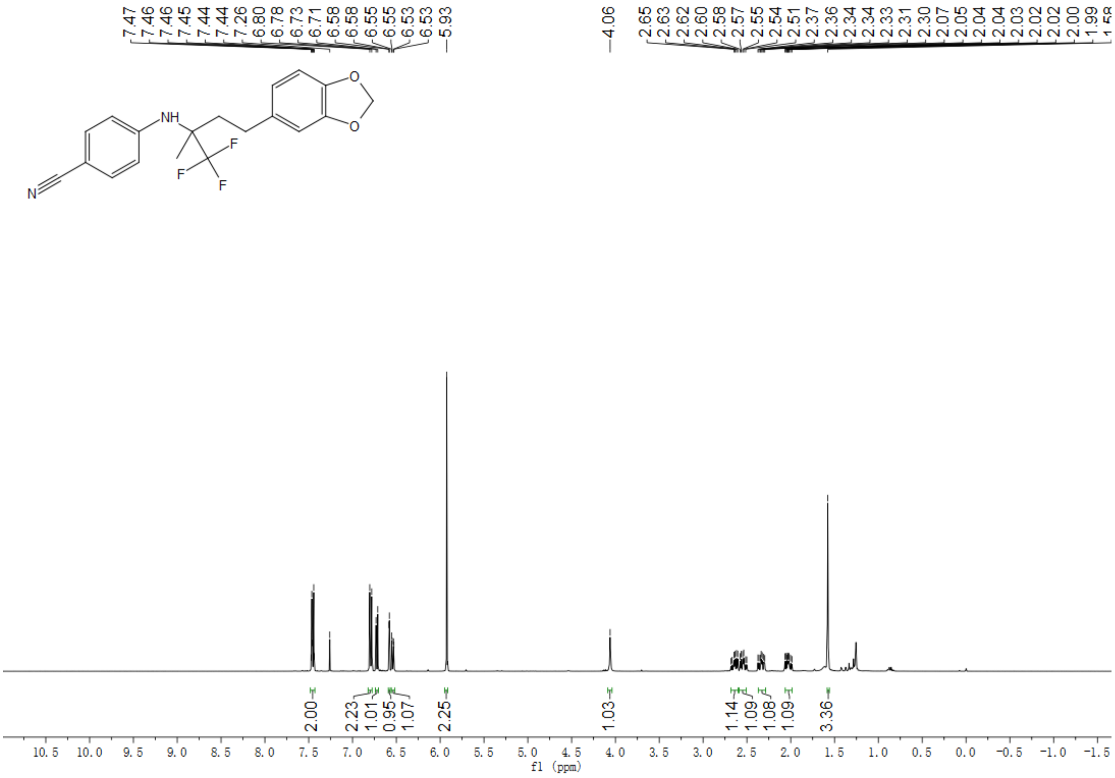

Supplementary Figure 24. ^{1}H NMR (400 MHz, room temperature, $CDCl_3$) spectra of product **9**

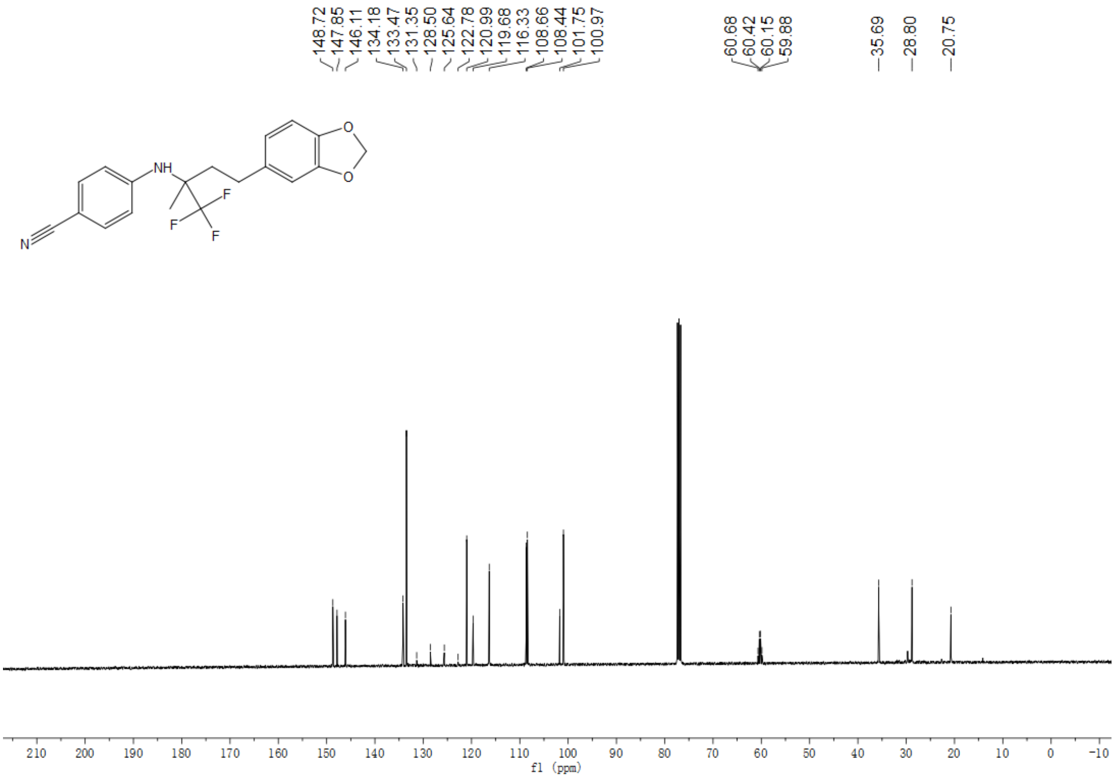

Supplementary Figure 25. ^{13}C NMR (101 MHz, room temperature, $CDCl_3$) spectra of product **9**

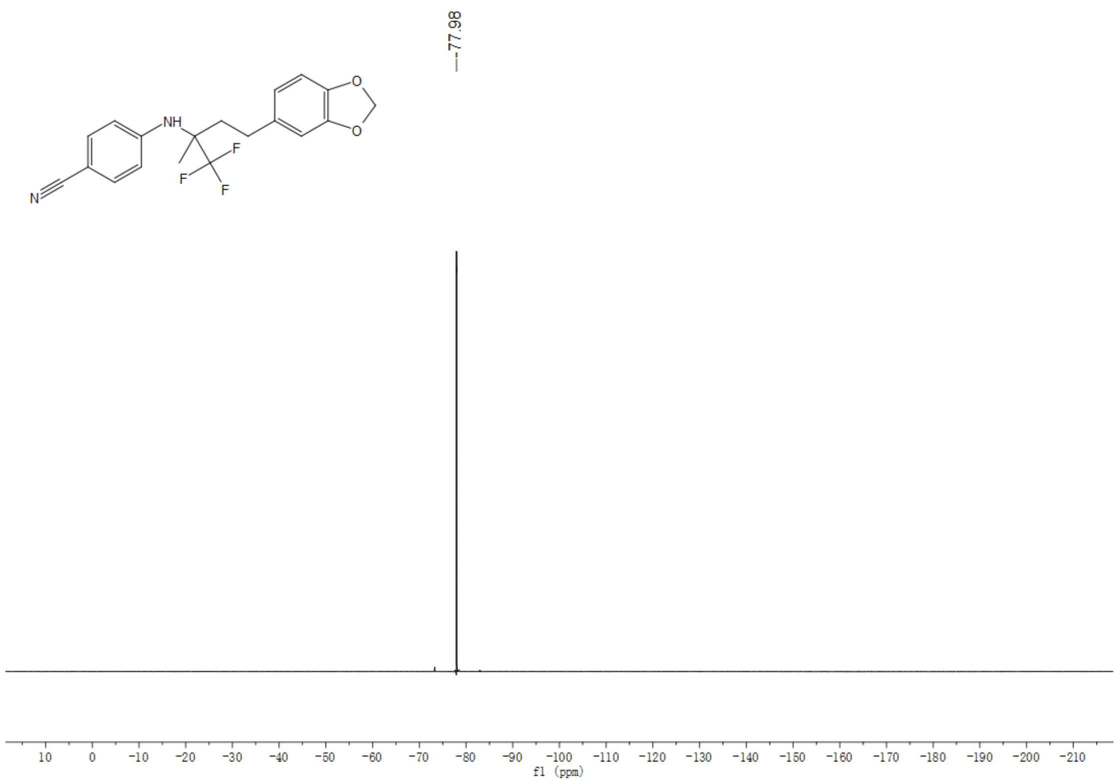

Supplementary Figure 26. ^{19}F NMR (376 MHz, room temperature, $CDCl_3$) spectra of product **9**

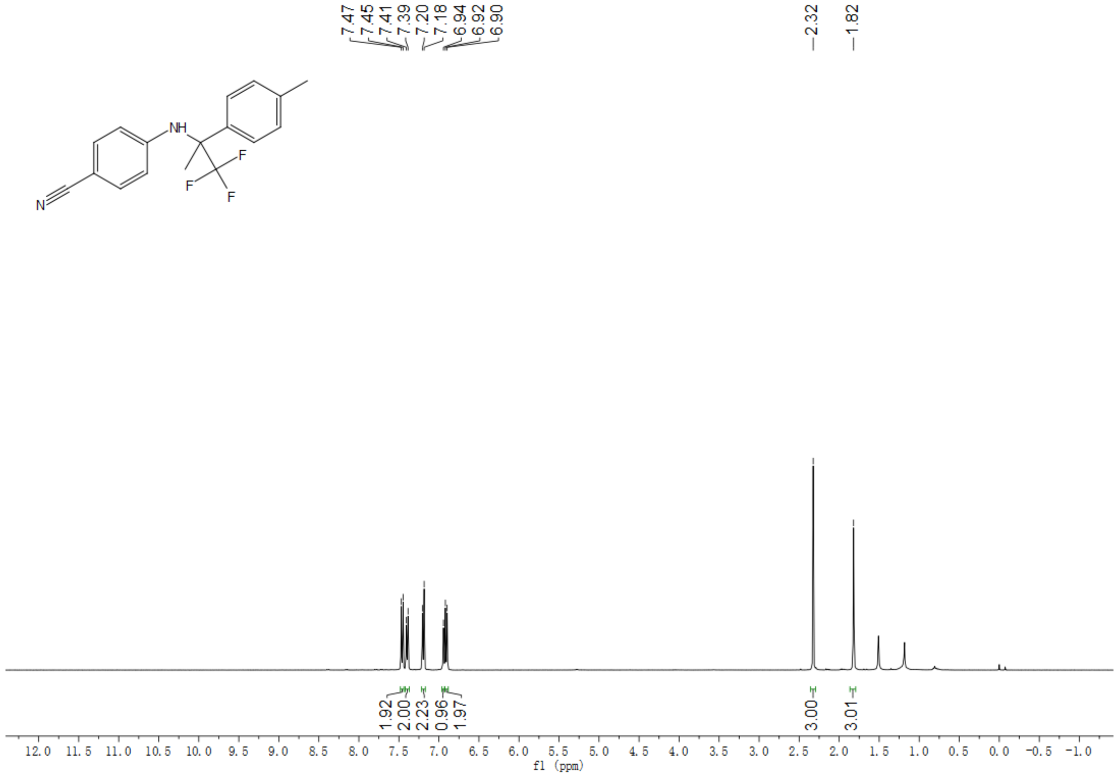

Supplementary Figure 27. ^{1}H NMR (400 MHz, room temperature, $CDCl_3$) spectra of product **10**

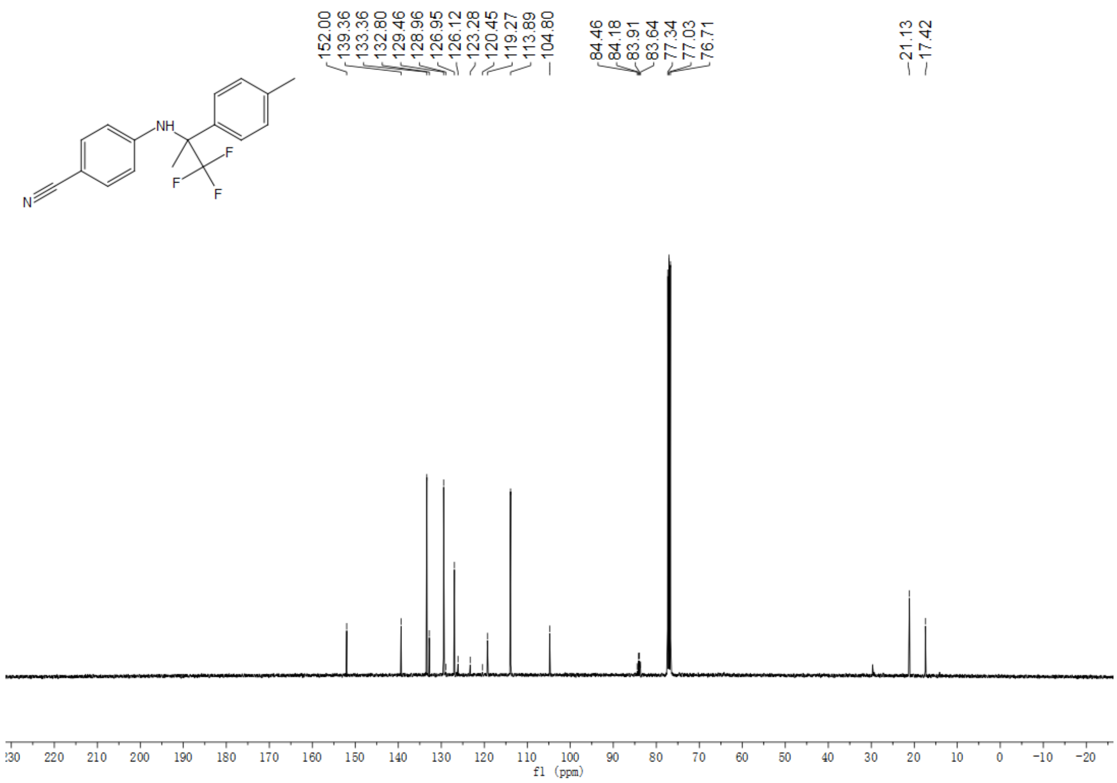

Supplementary Figure 28. ^{13}C NMR (101 MHz, room temperature, $CDCl_3$) spectra of product **10**

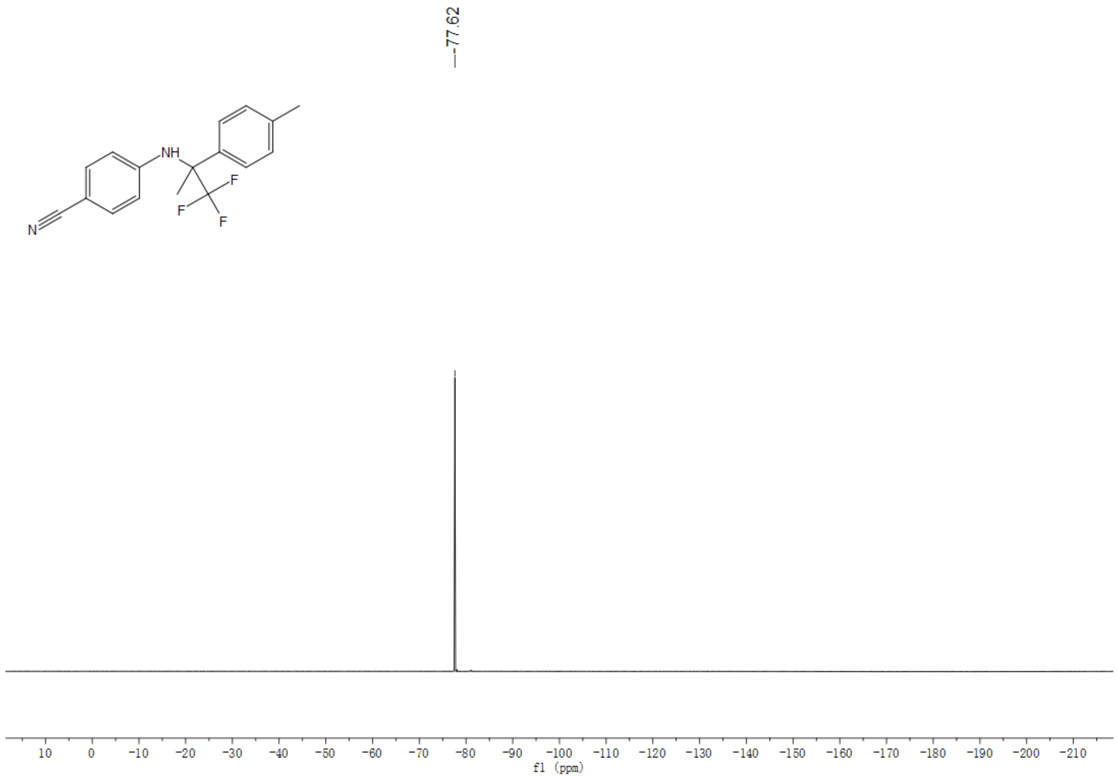

Supplementary Figure 29. ^{19}F NMR (376 MHz, room temperature, $CDCl_3$) spectra of product **10**

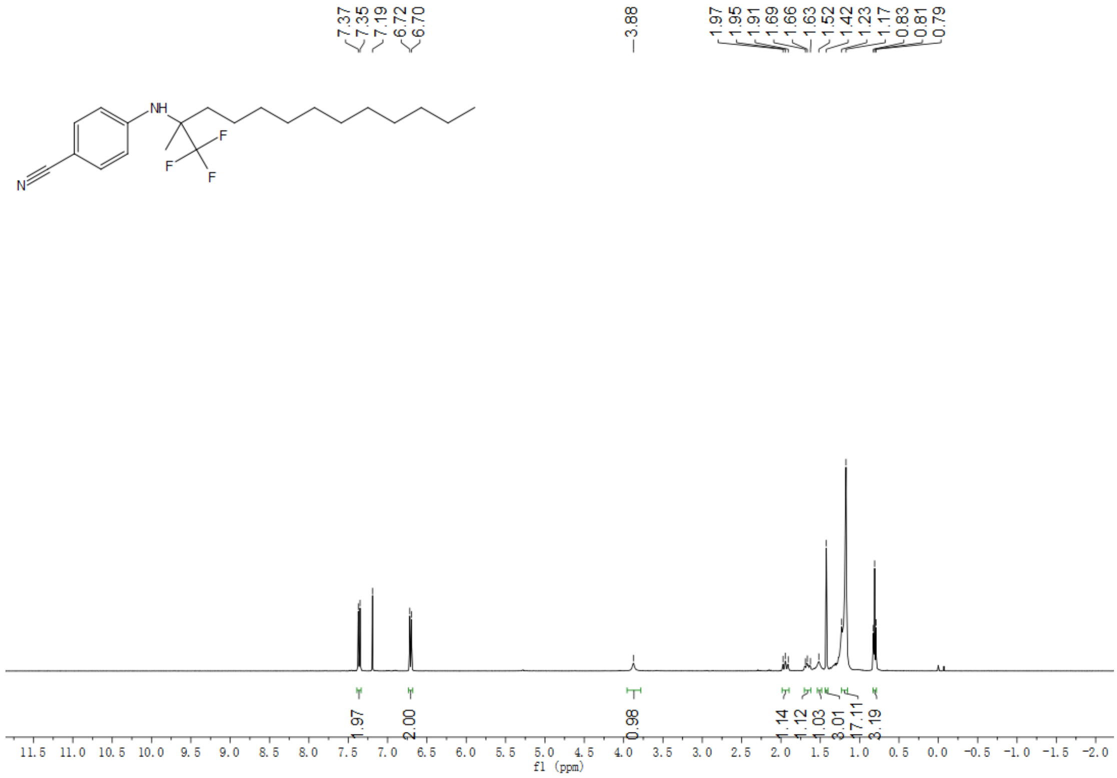

Supplementary Figure 30. ^{1}H NMR (400 MHz, room temperature, $CDCl_3$) spectra of product **11**

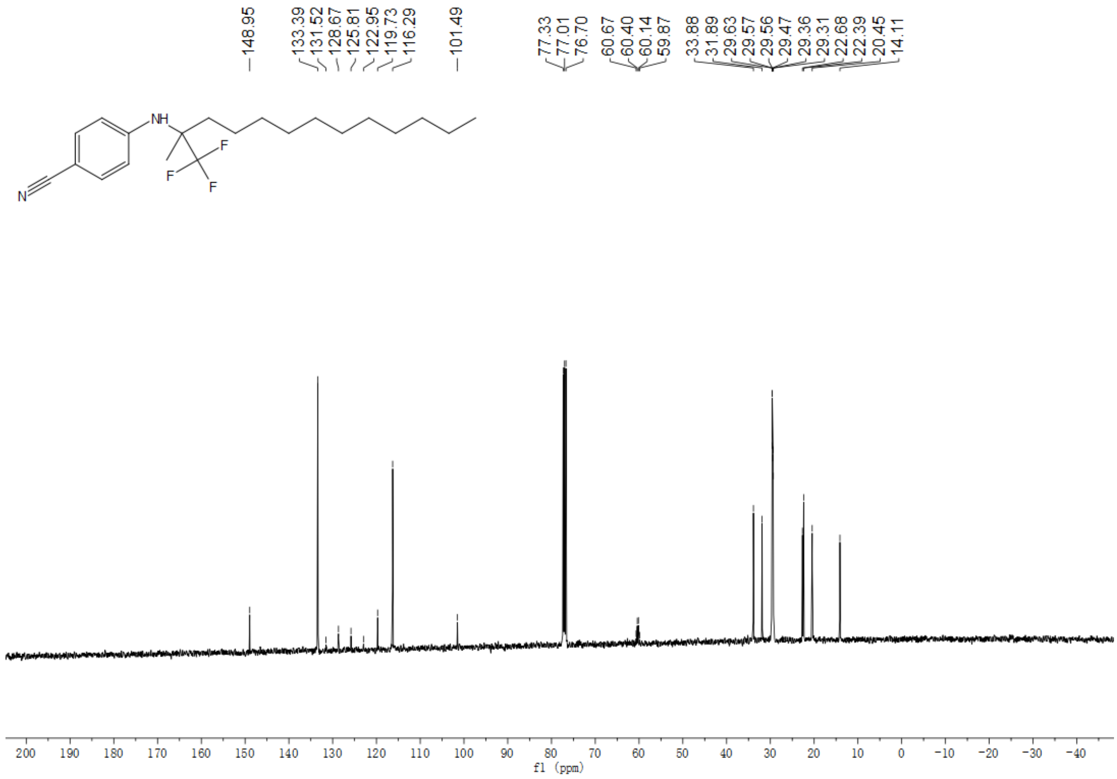

Supplementary Figure 31. ^{13}C NMR (101 MHz, room temperature, $CDCl_3$) spectra of product **11**

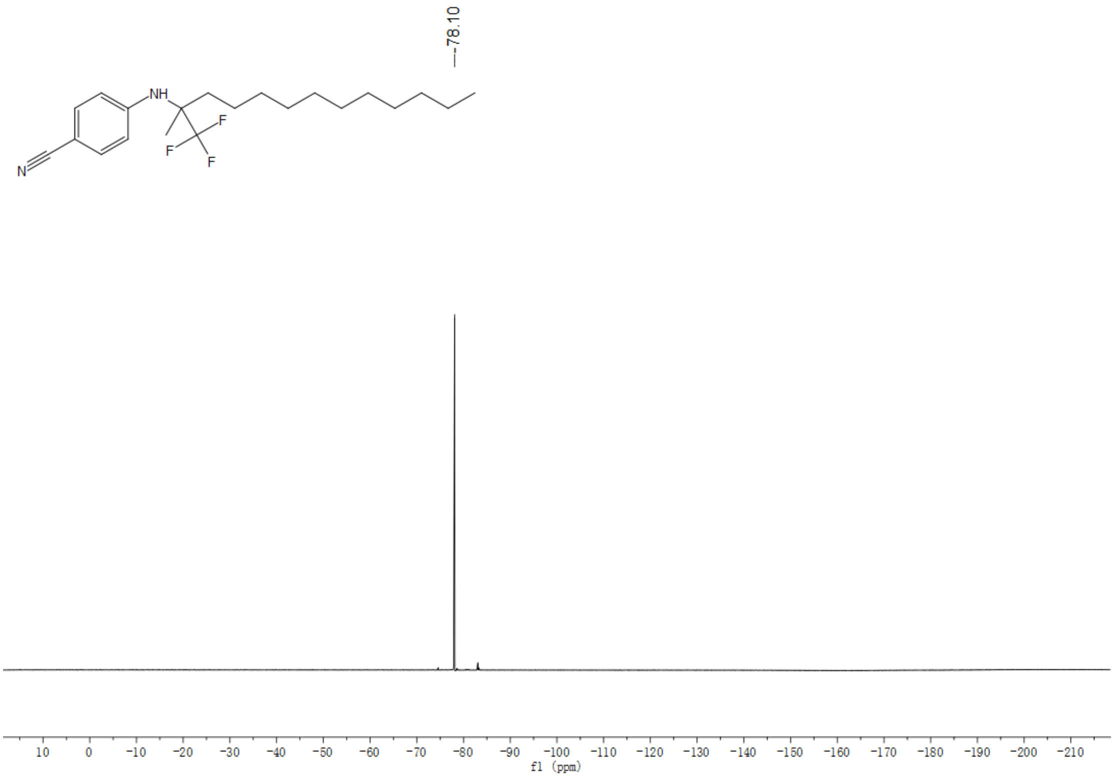

Supplementary Figure 32. ^{19}F NMR (376 MHz, room temperature, $CDCl_3$) spectra of product **11**

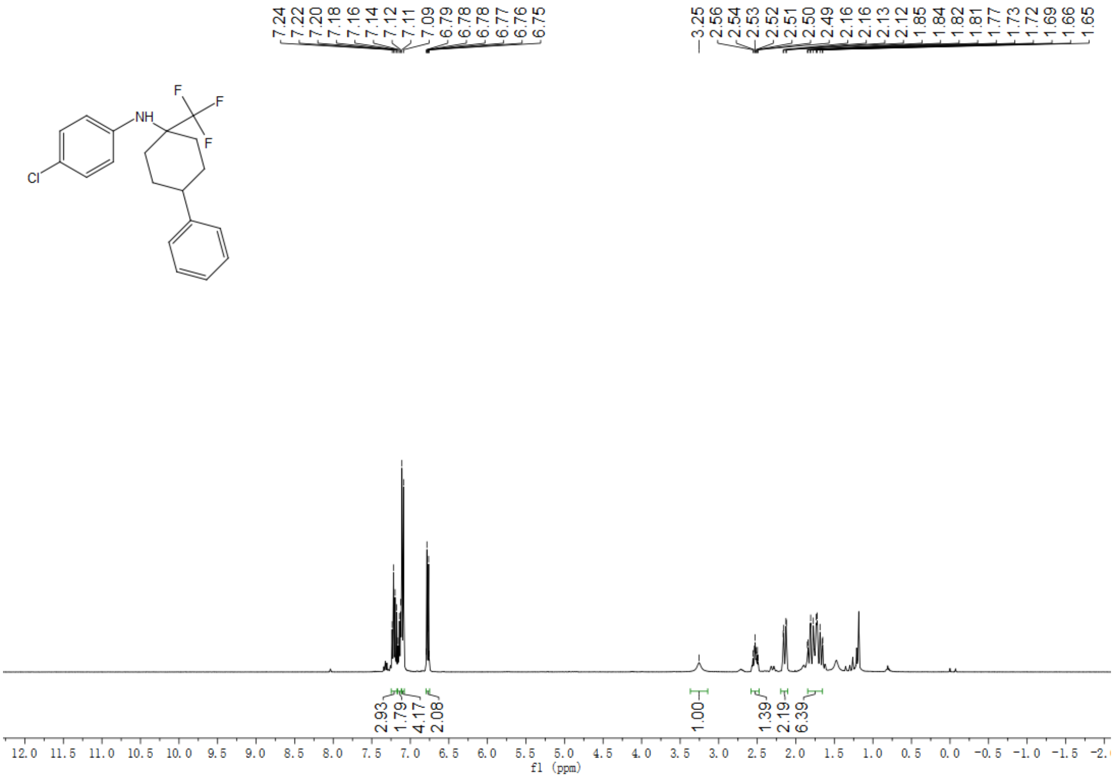

Supplementary Figure 33. ^{1}H NMR (400 MHz, room temperature, $CDCl_3$) spectra of product **12**

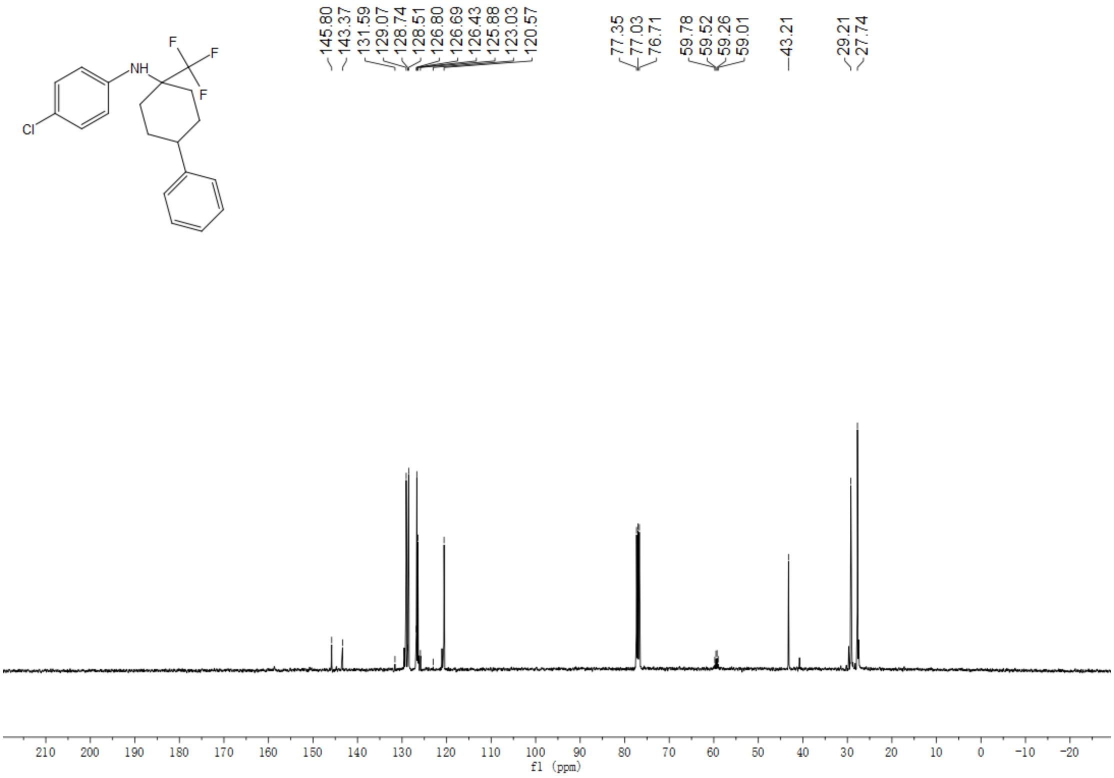

Supplementary Figure 34. ^{13}C NMR (101 MHz, room temperature, $CDCl_3$) spectra of product **12**

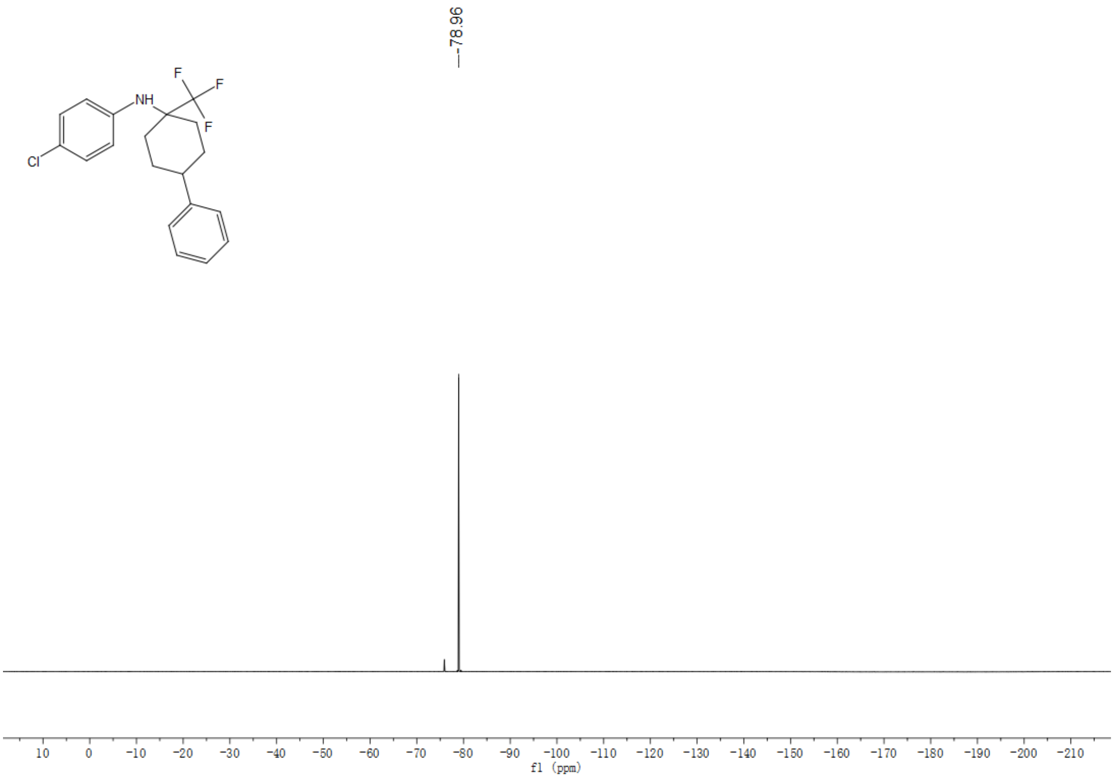

Supplementary Figure 35. ^{19}F NMR (376 MHz, room temperature, $CDCl_3$) spectra of product **12**

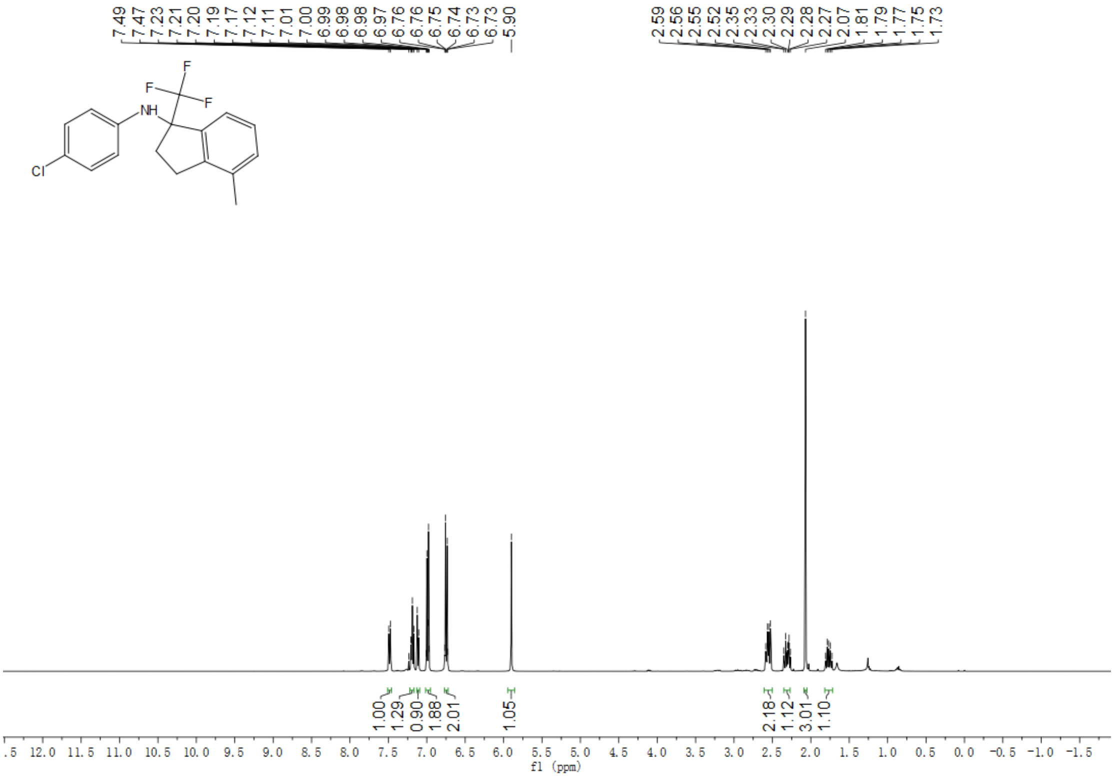

Supplementary Figure 36. ^{1}H NMR (400 MHz, room temperature, $CDCl_3$) spectra of product **13**

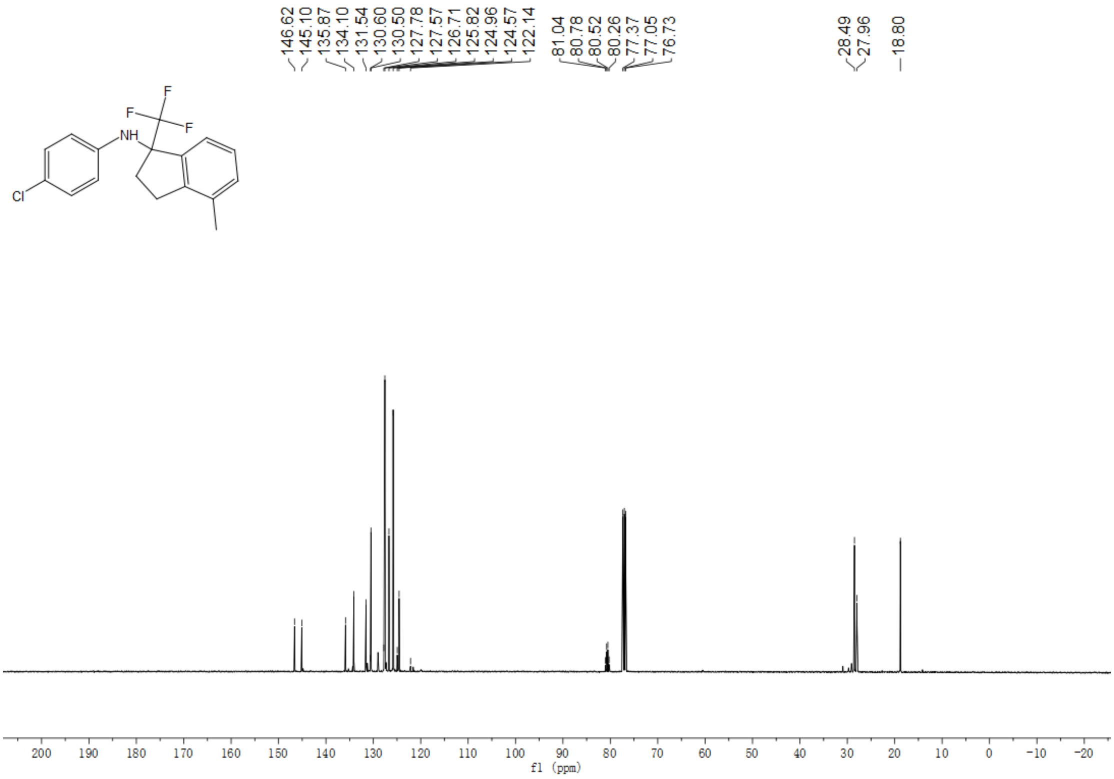

Supplementary Figure 37. ^{13}C NMR (101 MHz, room temperature, $CDCl_3$) spectra of product **13**

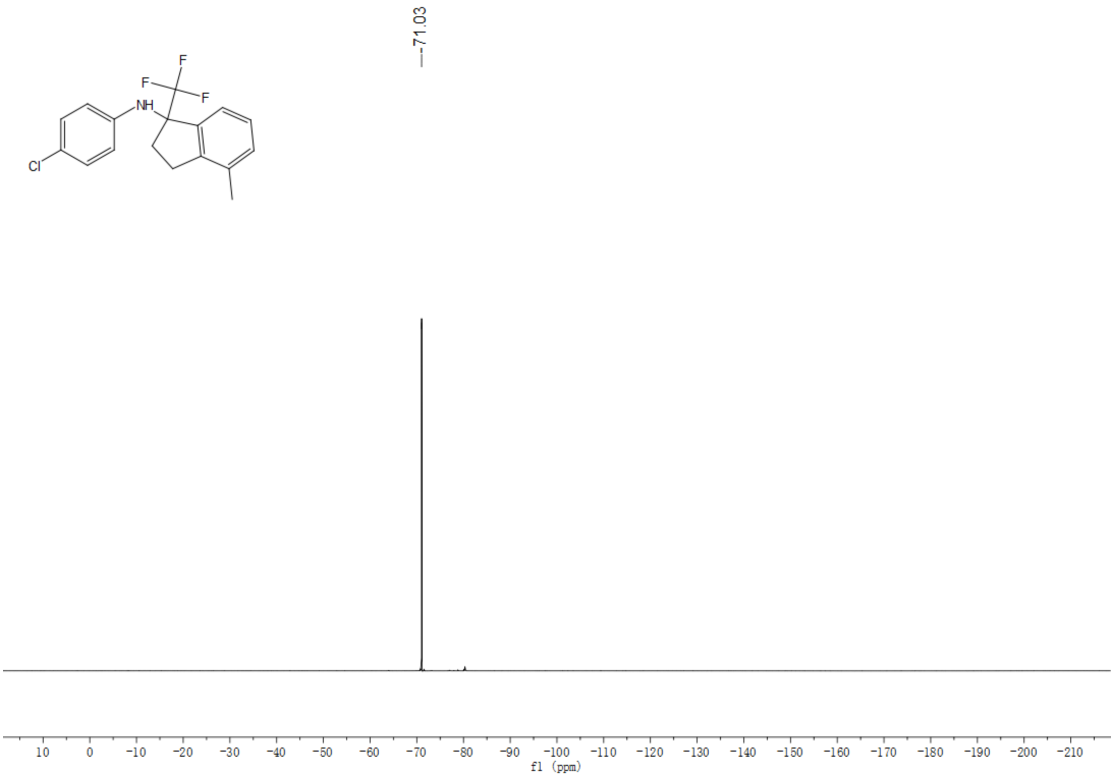

Supplementary Figure 38. ^{19}F NMR (376 MHz, room temperature, $CDCl_3$) spectra of product **13**

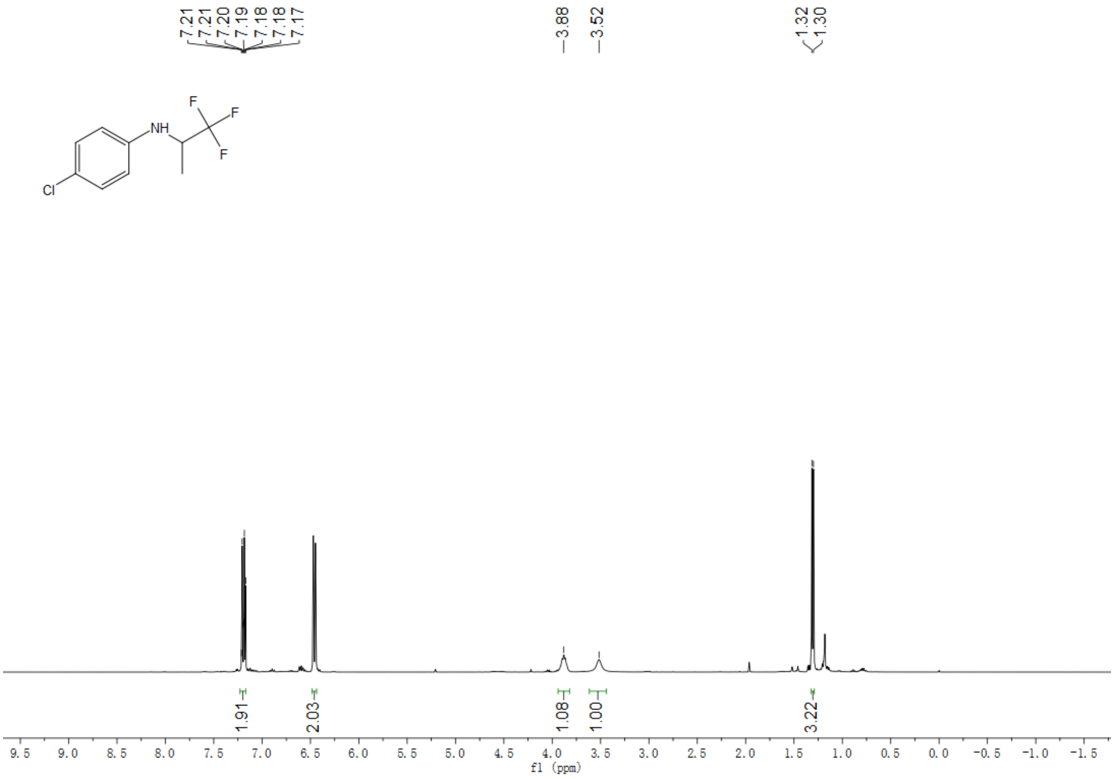

Supplementary Figure 39. ^{1}H NMR (400 MHz, room temperature, $CDCl_3$) spectra of product **14**

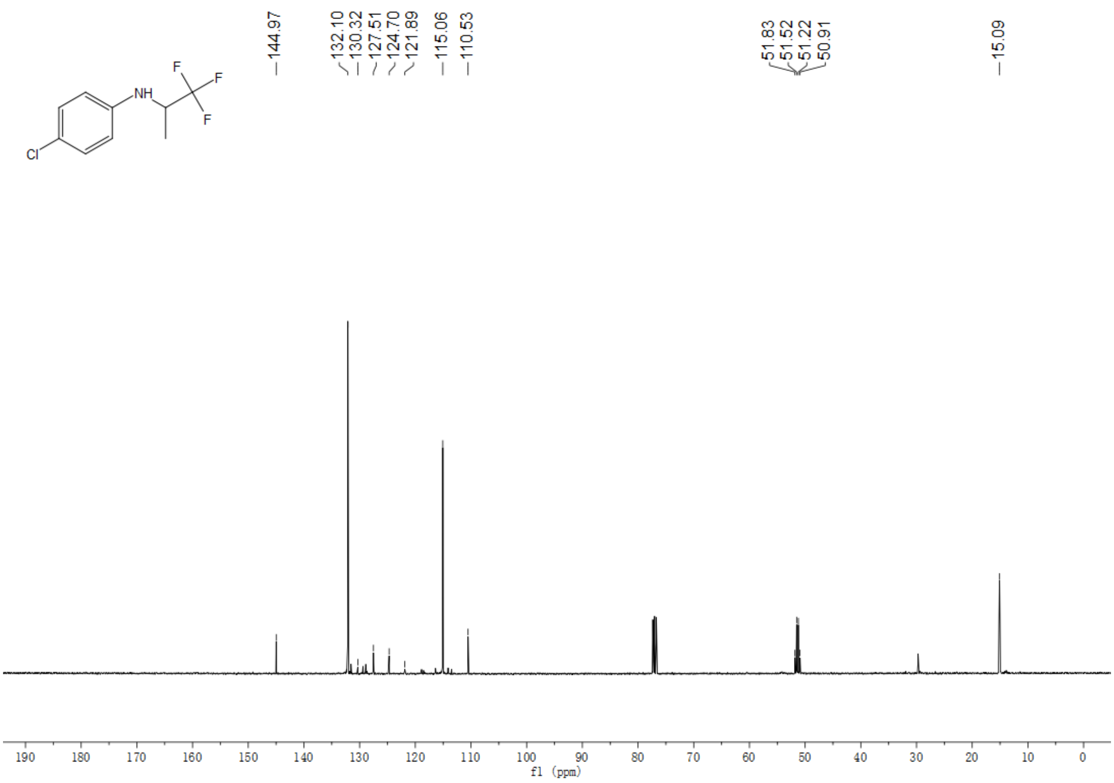

Supplementary Figure 40. ^{13}C NMR (101 MHz, room temperature, $CDCl_3$) spectra of product **14**

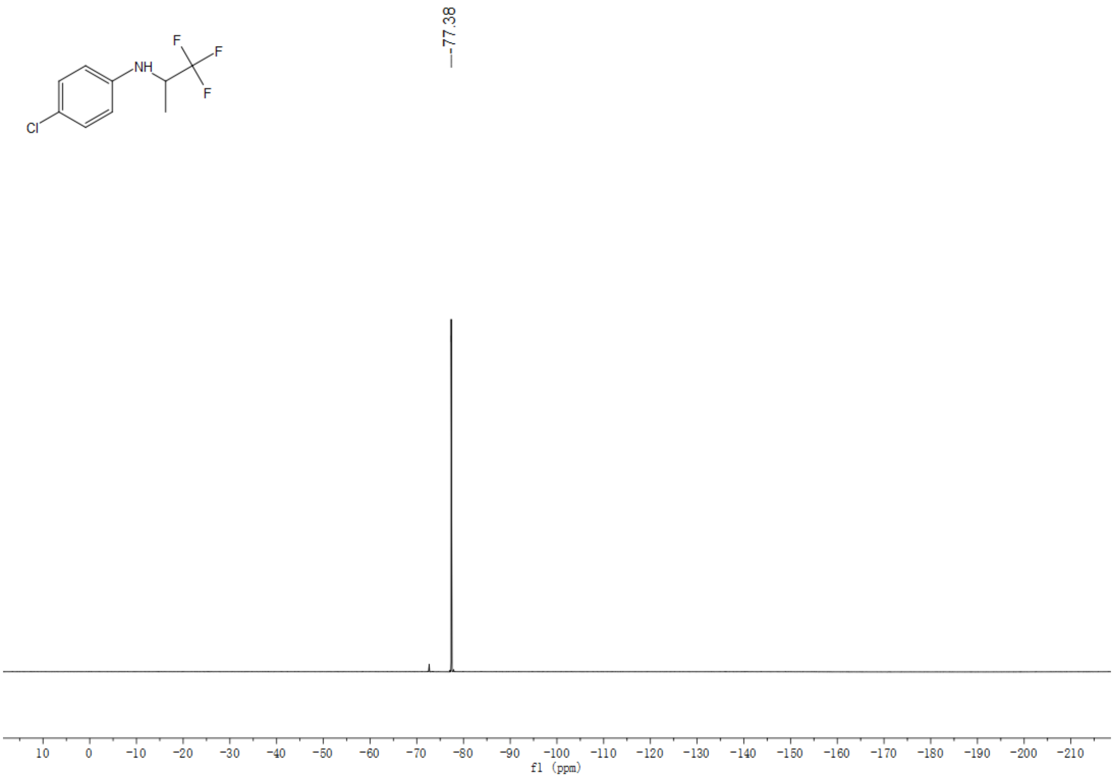

Supplementary Figure 41. ^{19}F NMR (376 MHz, room temperature, $CDCl_3$) spectra of product **14**

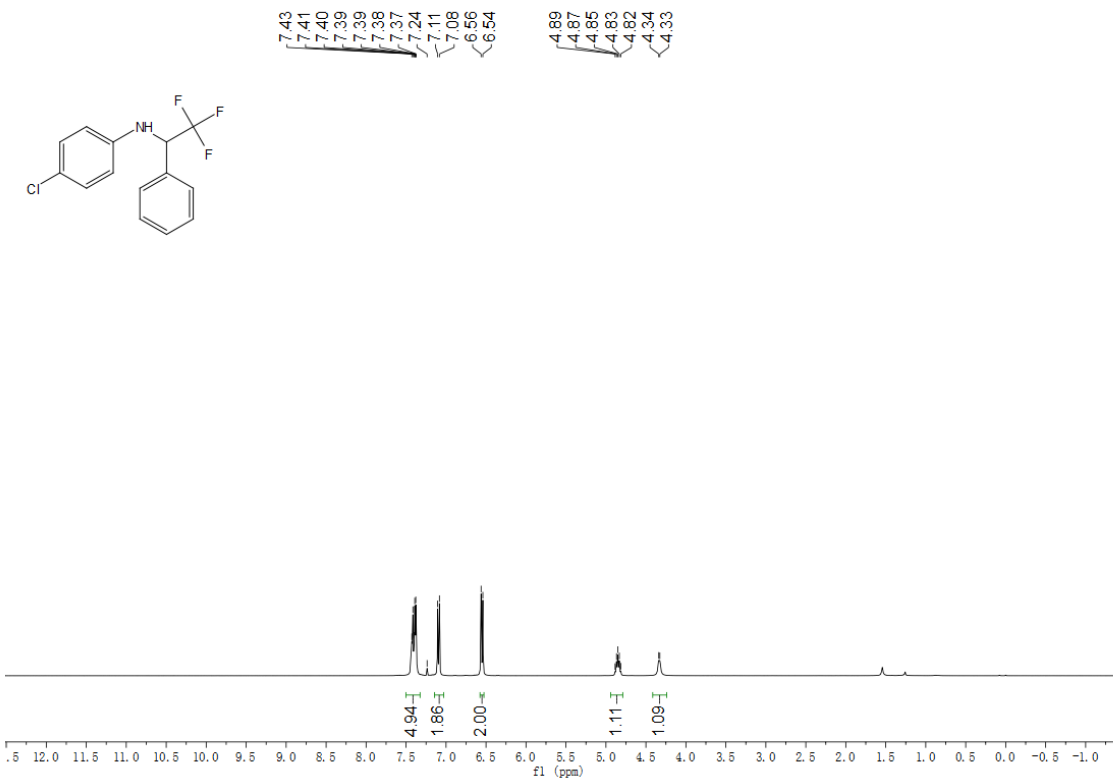

Supplementary Figure 42. ^{1}H NMR (400 MHz, room temperature, $CDCl_3$) spectra of product **15**

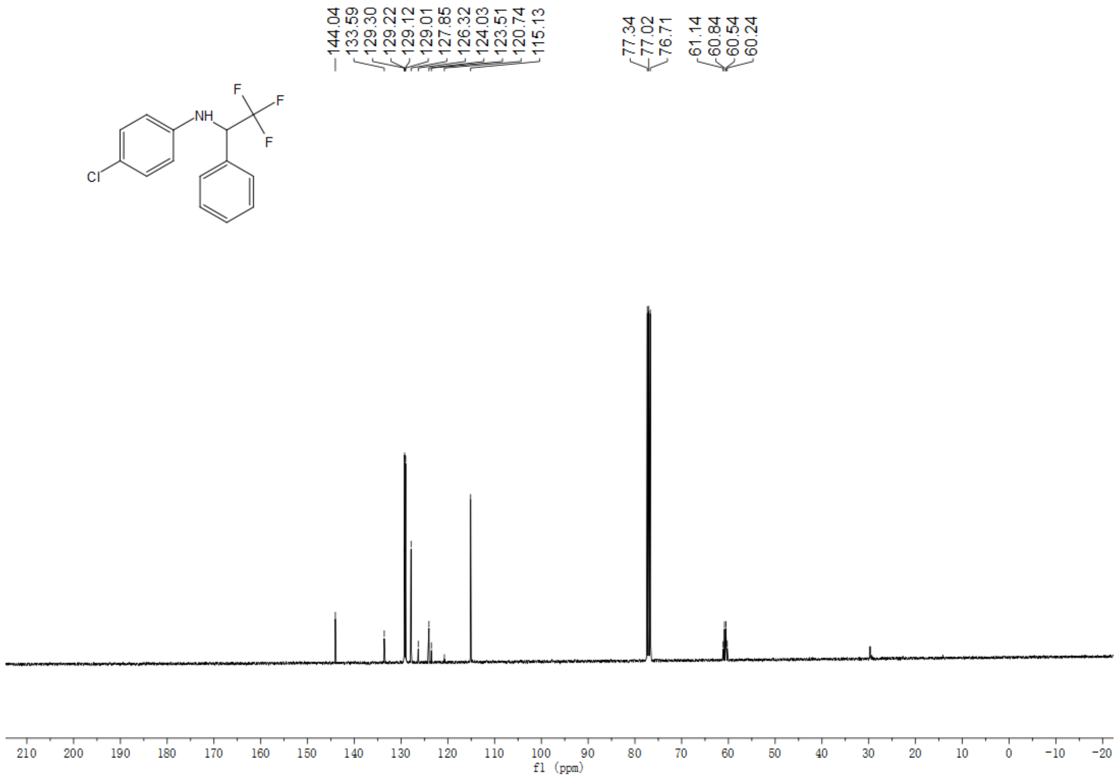

Supplementary Figure 43. ^{13}C NMR (101 MHz, room temperature, $CDCl_3$) spectra of product **15**

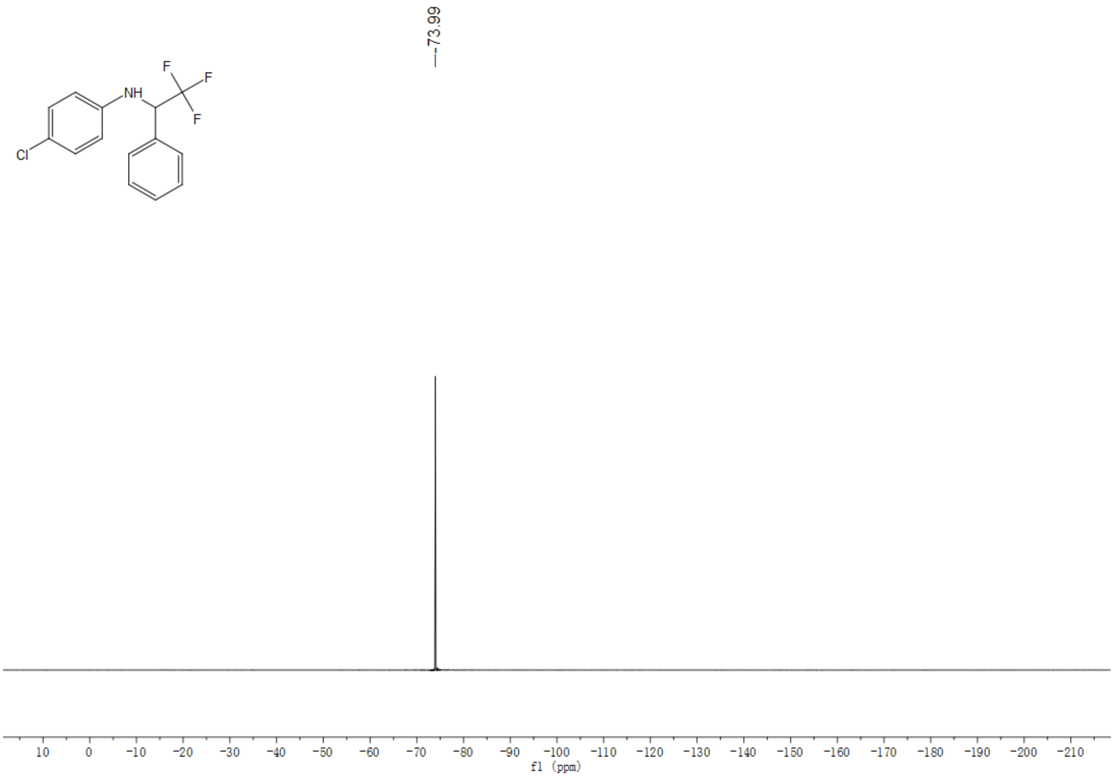

Supplementary Figure 44. ^{19}F NMR (376 MHz, room temperature, $CDCl_3$) spectra of product **15**

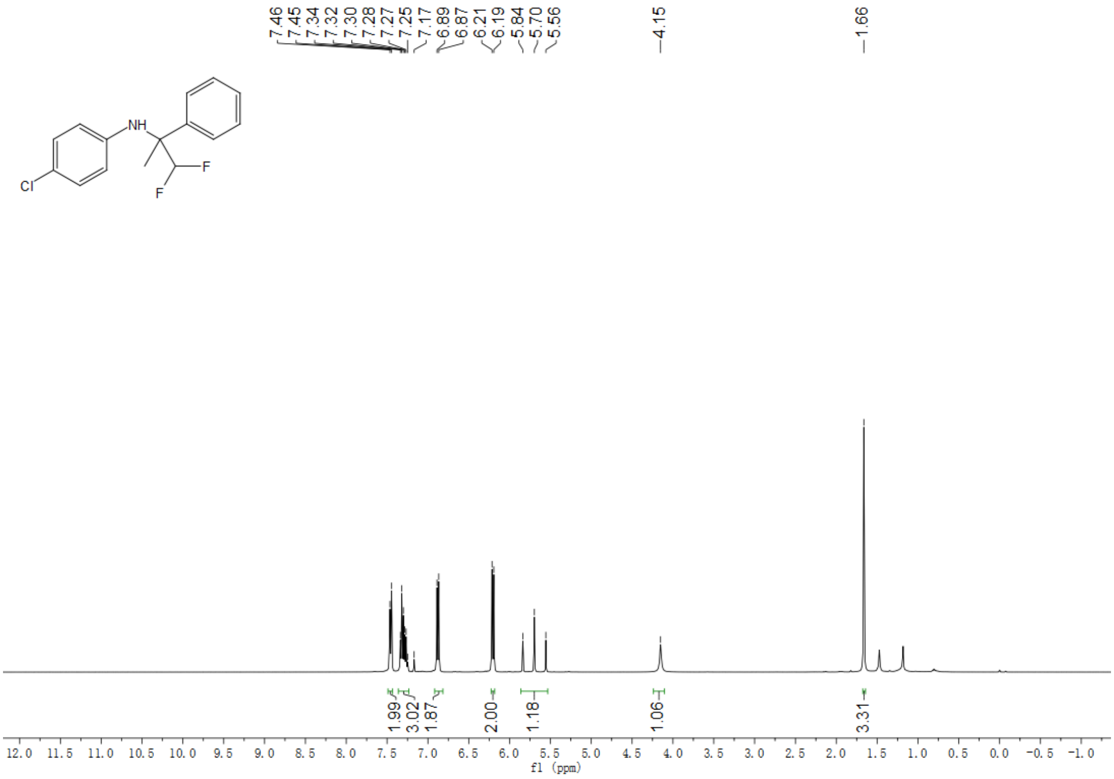

Supplementary Figure 45. ^{1}H NMR (400 MHz, room temperature, $CDCl_3$) spectra of product **16**

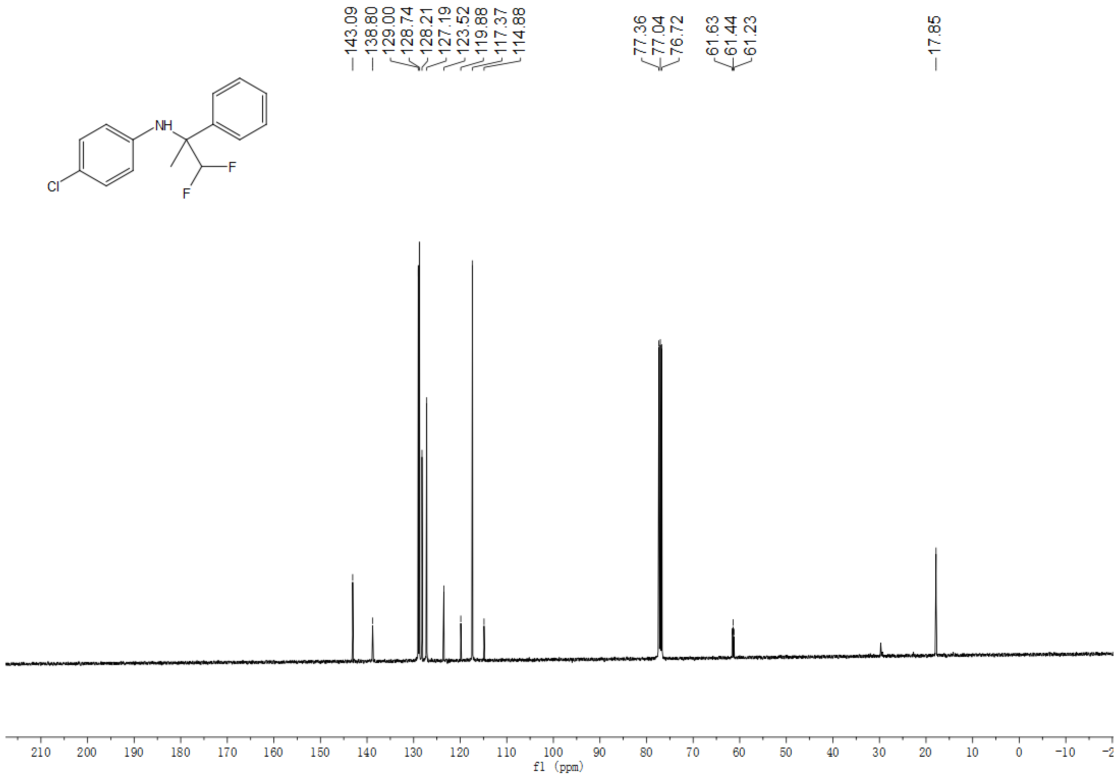

Supplementary Figure 46. ^{13}C NMR (101 MHz, room temperature, $CDCl_3$) spectra of product **16**

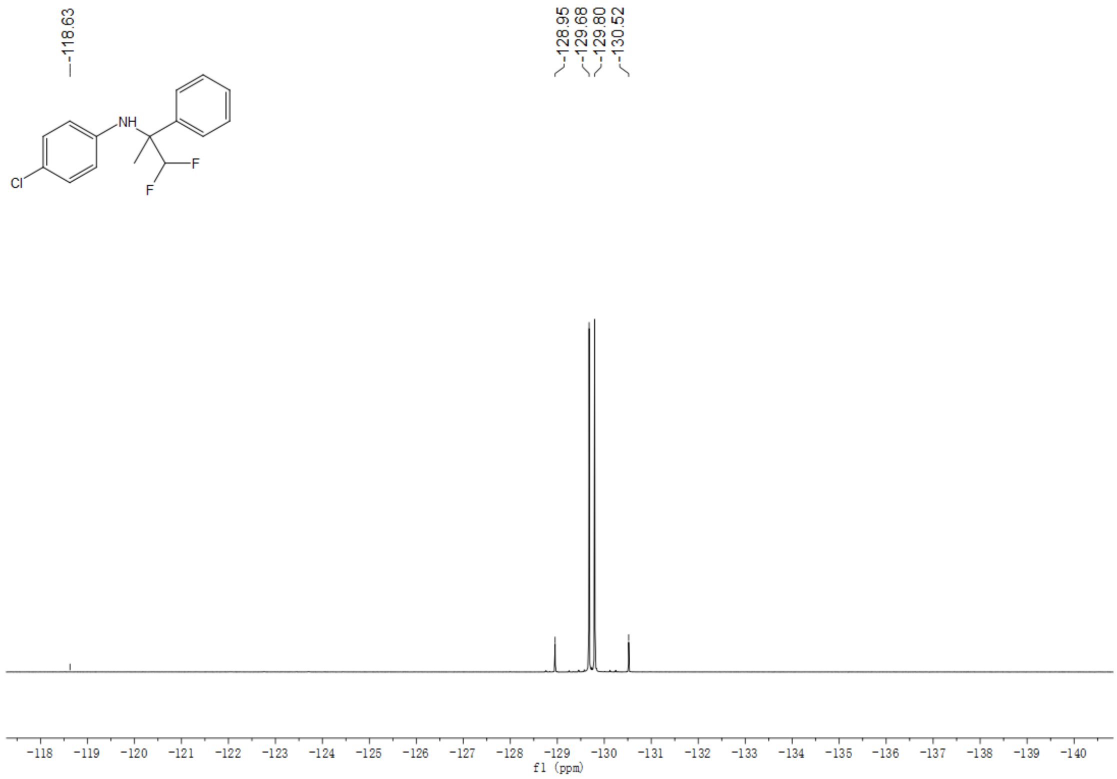

Supplementary Figure 47. ^{19}F NMR (376 MHz, room temperature, $CDCl_3$) spectra of product **16**

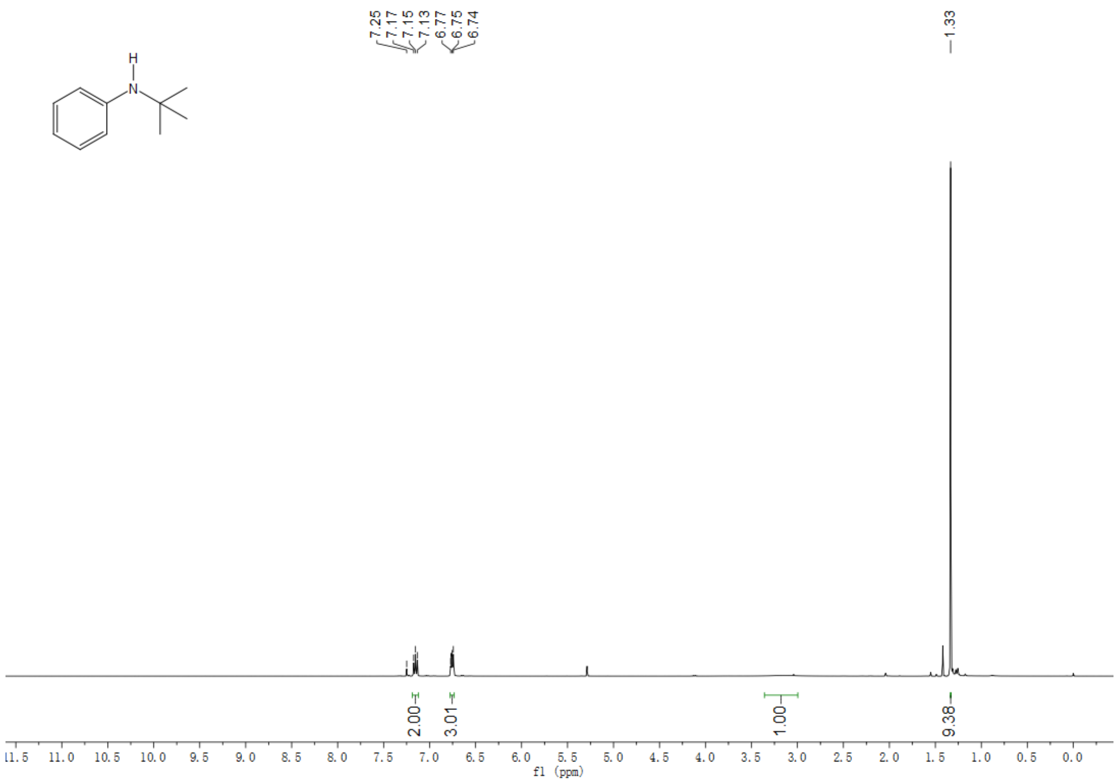

Supplementary Figure 48. ^{1}H NMR (400 MHz, room temperature, $CDCl_3$) spectra of product **17**

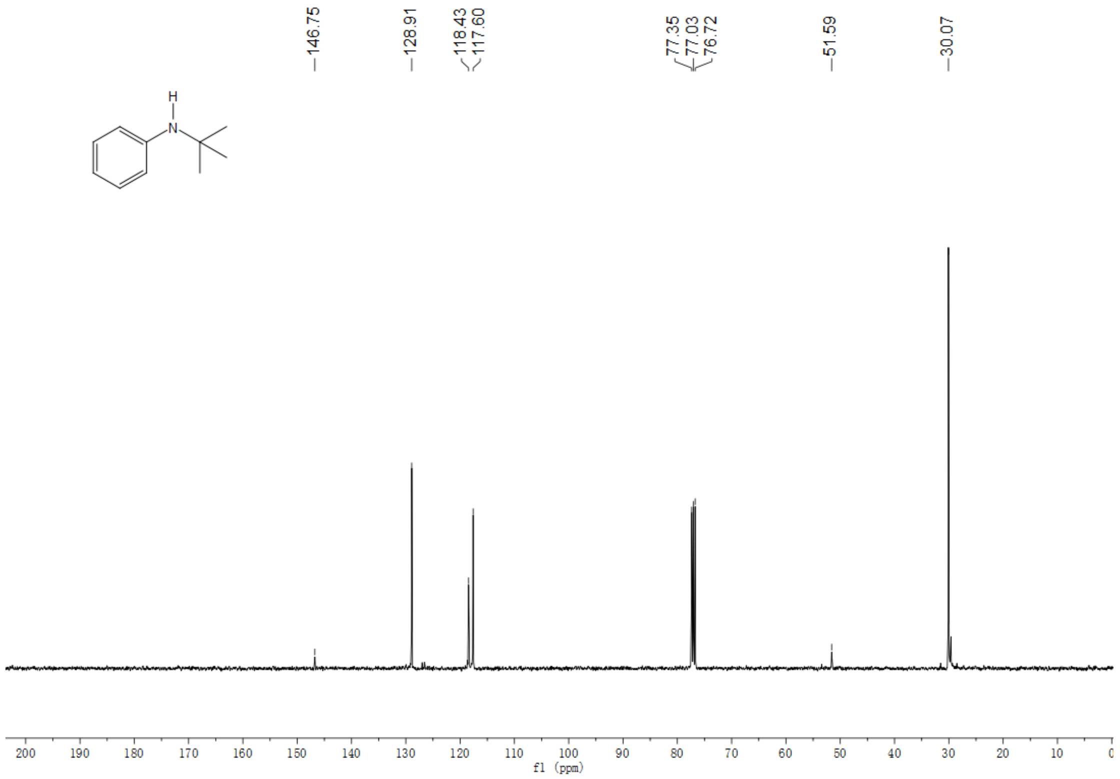

Supplementary Figure 49. ^{13}C NMR (101 MHz, room temperature, $CDCl_3$) spectra of product **17**

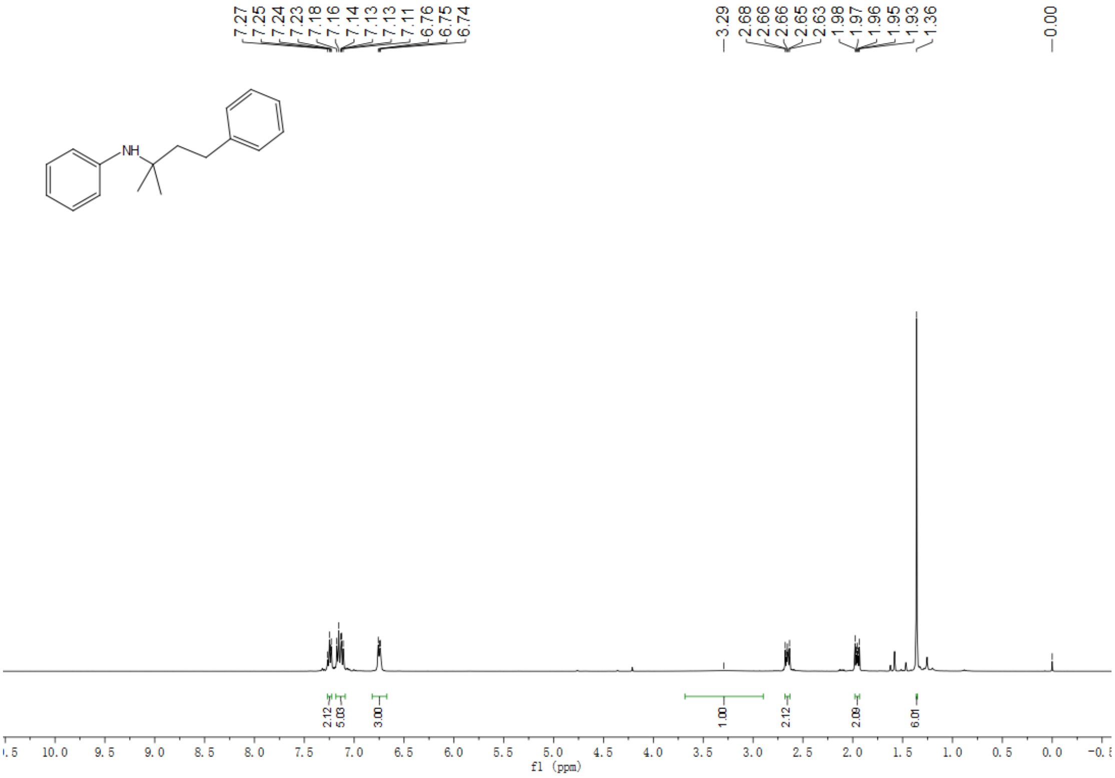

Supplementary Figure 50. ^{1}H NMR (400 MHz, room temperature, $CDCl_3$) spectra of product **18**

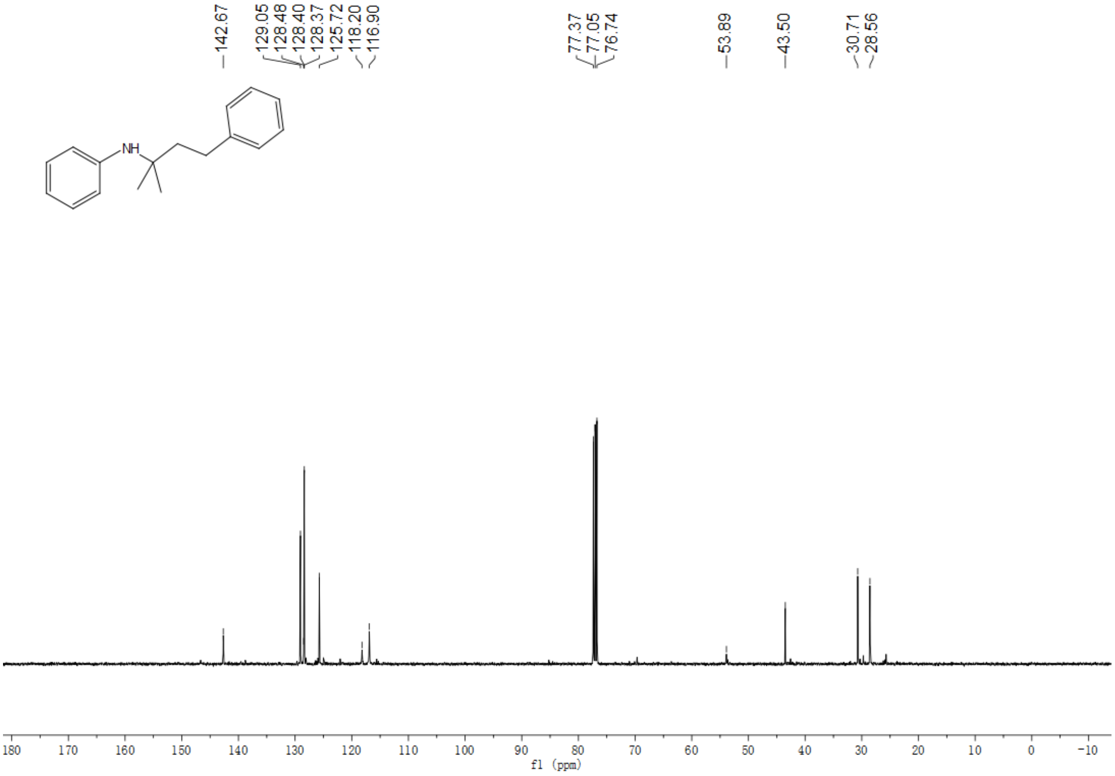

Supplementary Figure 51. ^{13}C NMR (101 MHz, room temperature, $CDCl_3$) spectra of product **18**

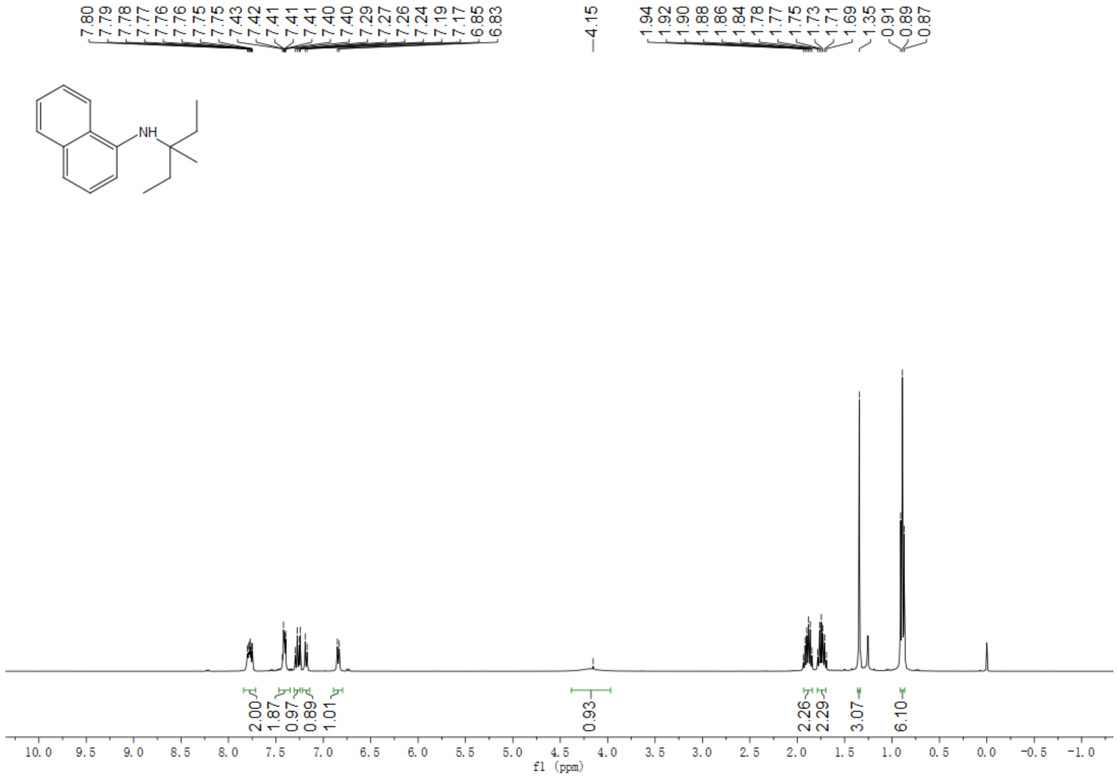

Supplementary Figure 52. ^{1}H NMR (400 MHz, room temperature, $CDCl_3$) spectra of product 1**9**

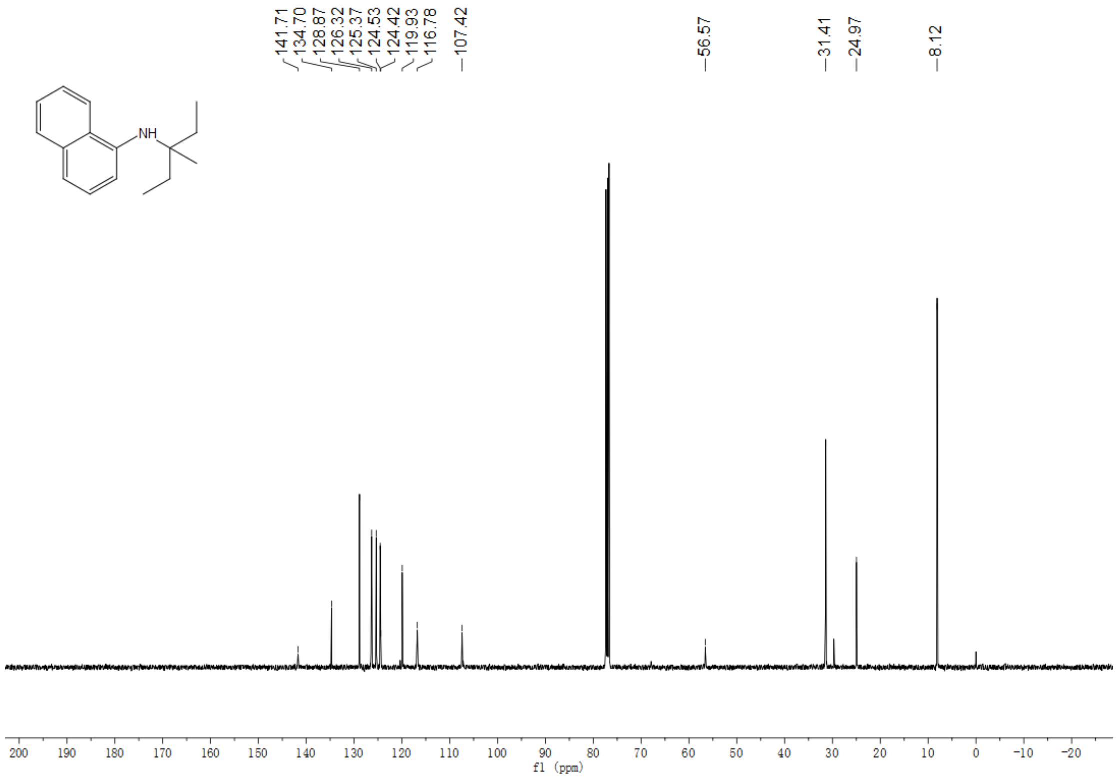

Supplementary Figure 53. ^{13}C NMR (101 MHz, room temperature, $CDCl_3$) spectra of product **19**

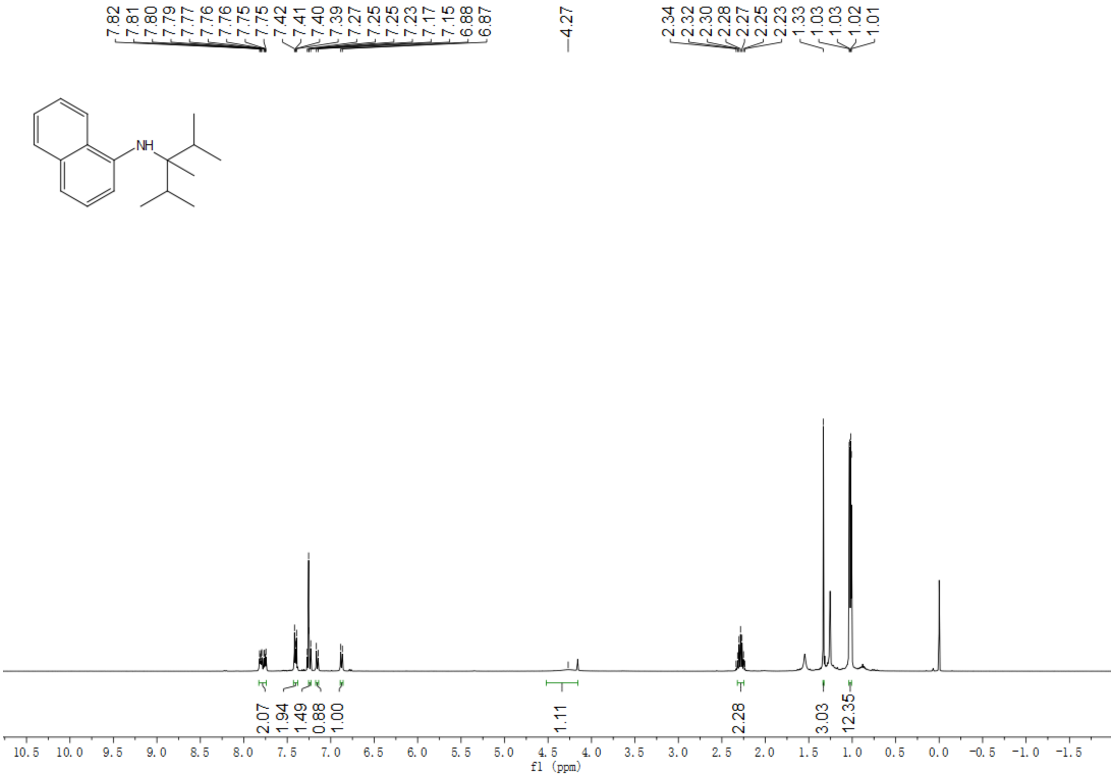

Supplementary Figure 54. ^{1}H NMR (400 MHz, room temperature, $CDCl_3$) spectra of product **20**

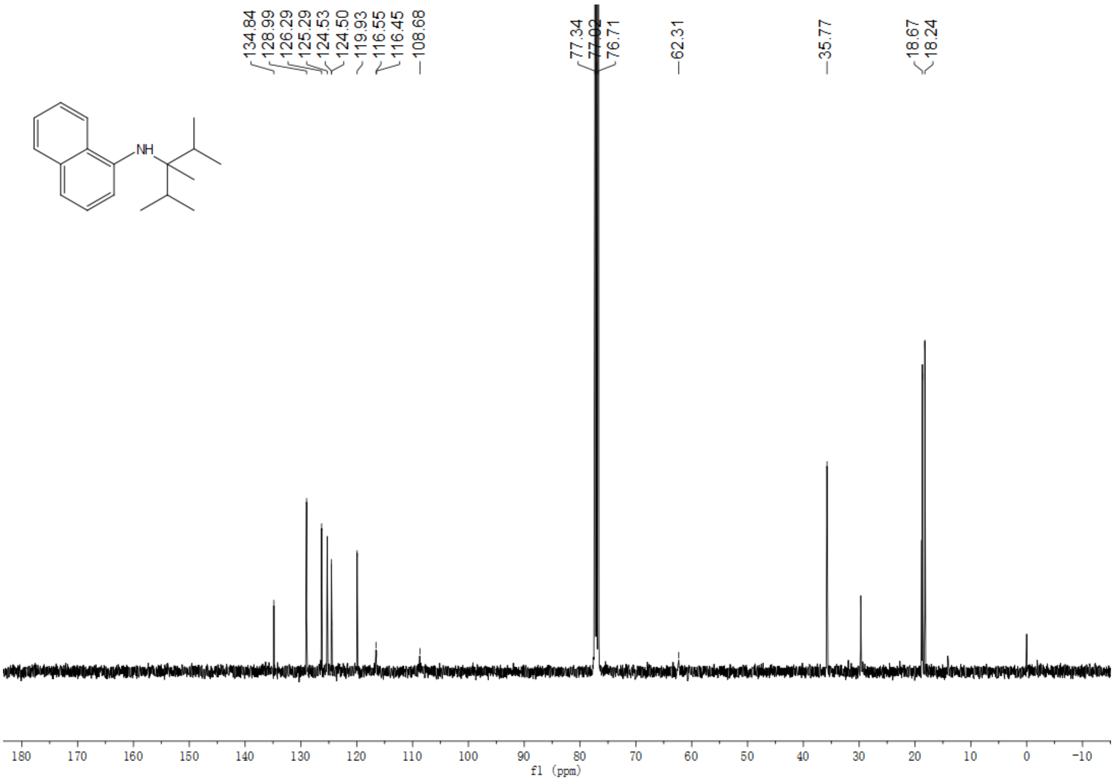

Supplementary Figure 55. ^{13}C NMR (101 MHz, room temperature, $CDCl_3$) spectra of product **20**

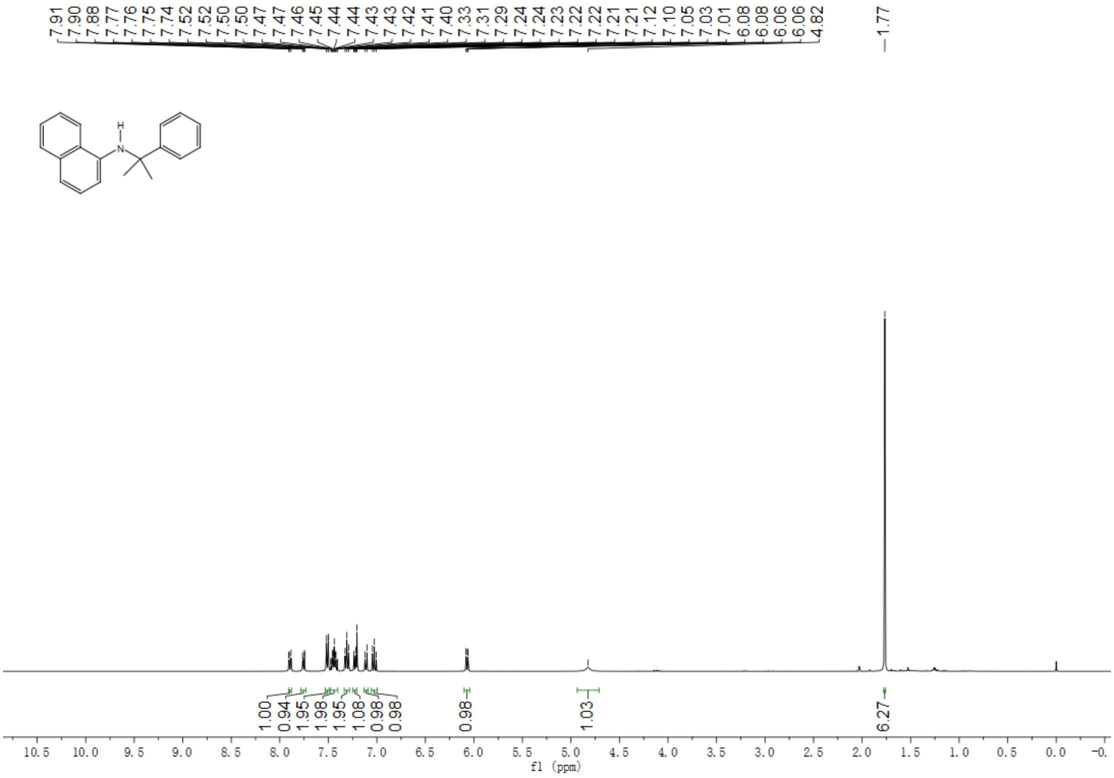

Supplementary Figure 56. ^{1}H NMR (400 MHz, room temperature, $CDCl_3$) spectra of product **21**

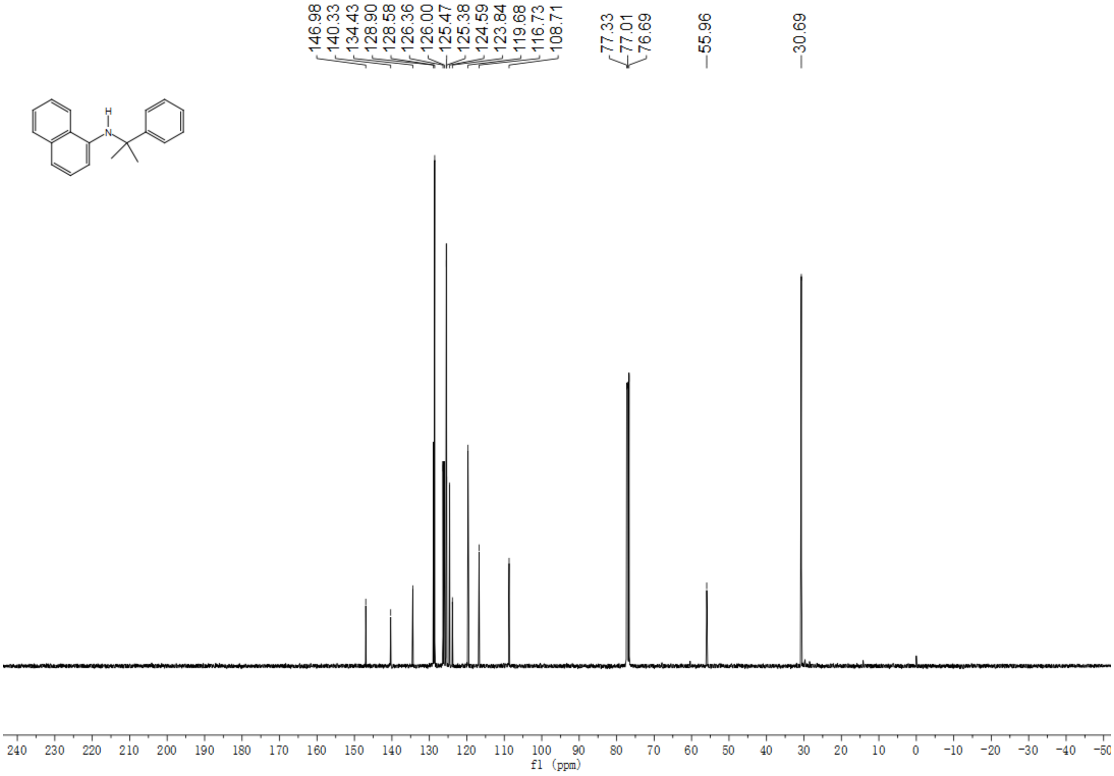

Supplementary Figure 57. ^{13}C NMR (101 MHz, room temperature, $CDCl_3$) spectra of product **21**

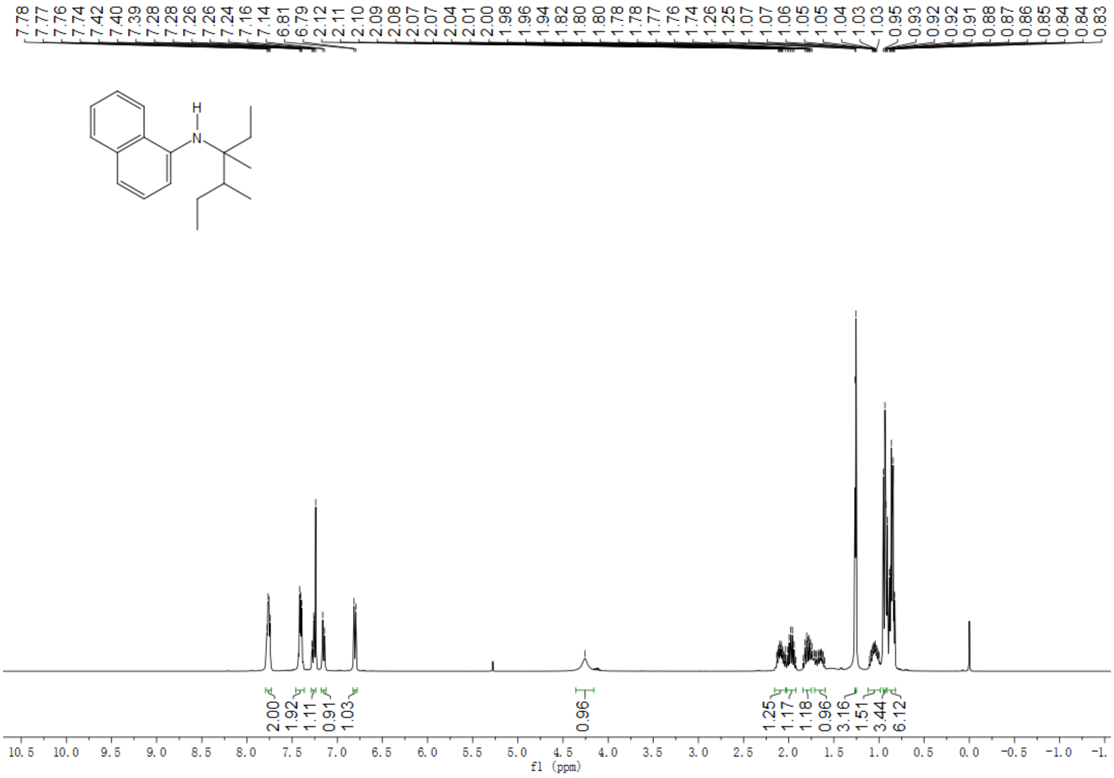

Supplementary Figure 58. ^{1}H NMR (400 MHz, room temperature, $CDCl_3$) spectra of product **22**

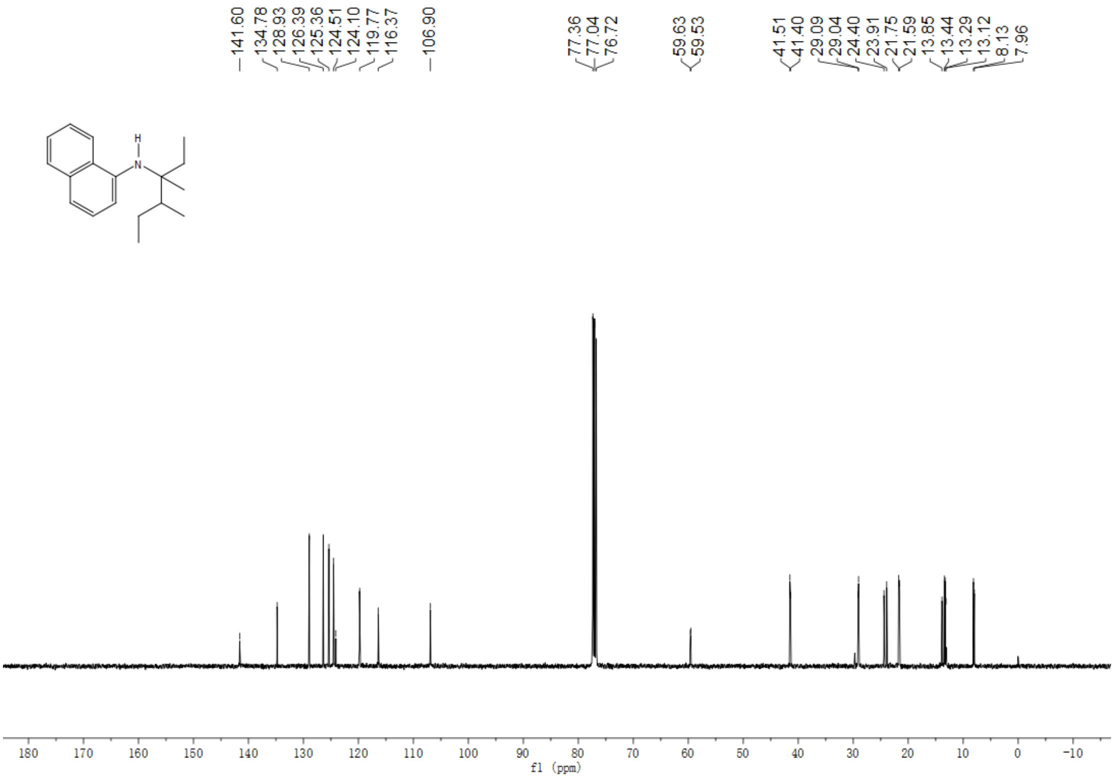

Supplementary Figure 59. ^{13}C NMR (101 MHz, room temperature, $CDCl_3$) spectra of product **22**

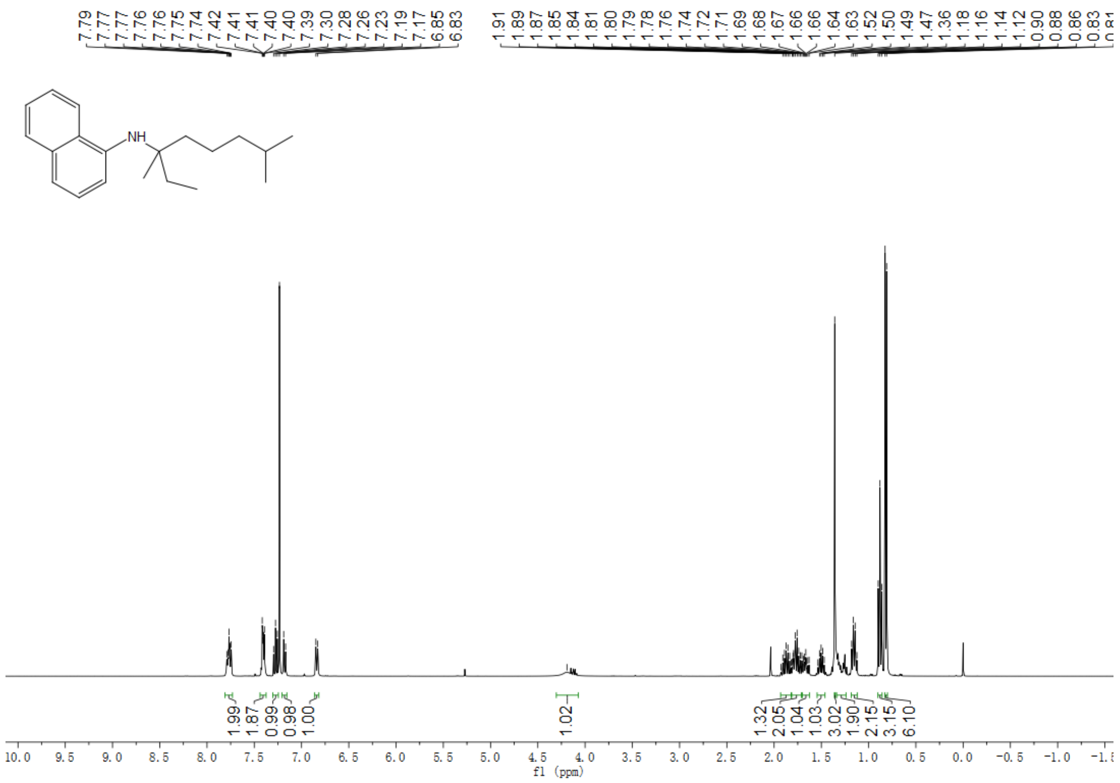

Supplementary Figure 60. ^{1}H NMR (400 MHz, room temperature, $CDCl_3$) spectra of product **23**

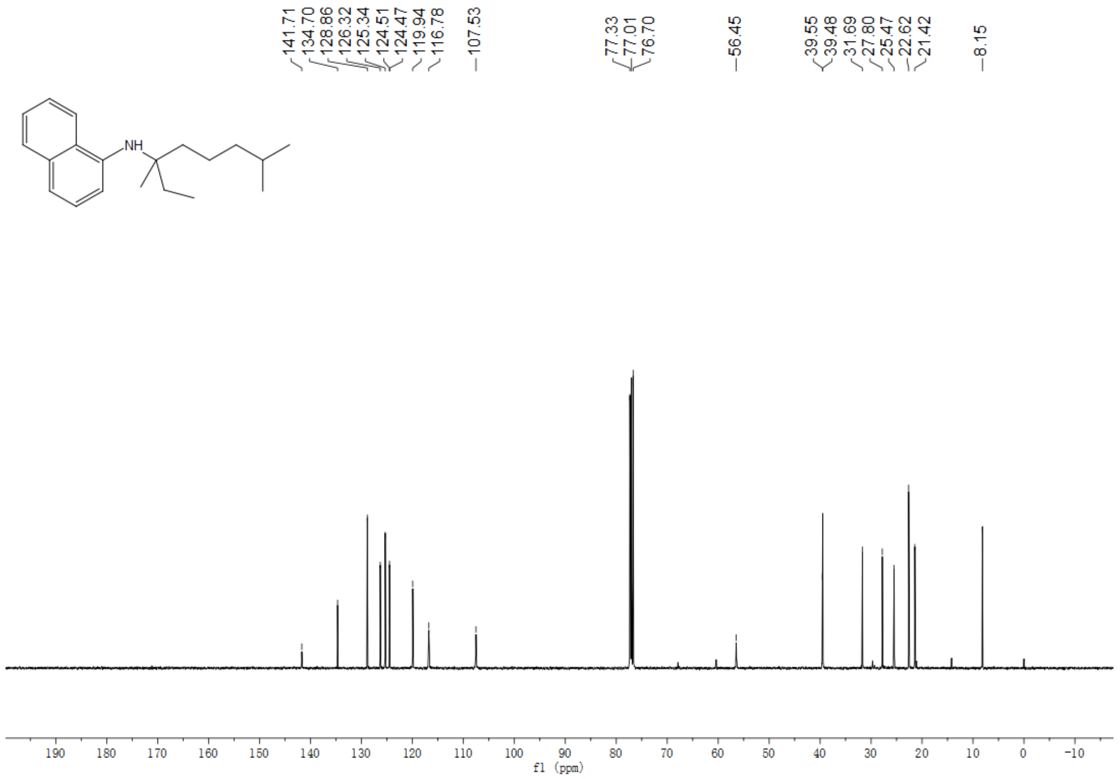

Supplementary Figure 61. ^{13}C NMR (101 MHz, room temperature, $CDCl_3$) spectra of product **23**

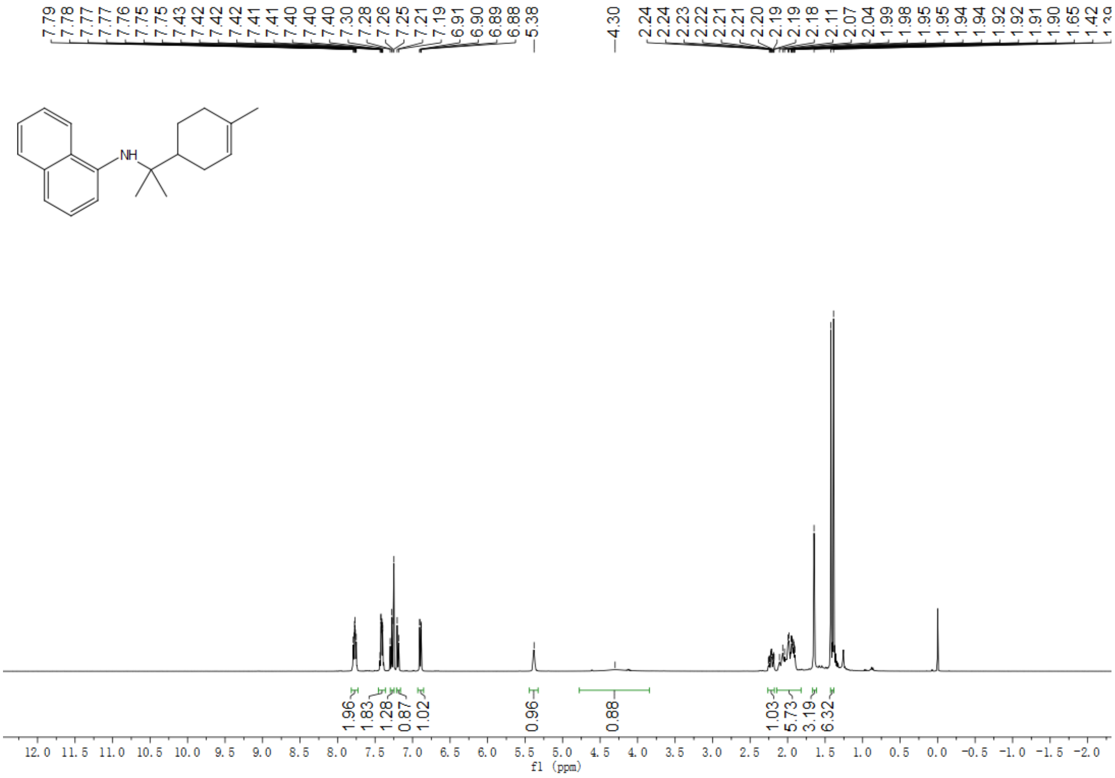

Supplementary Figure 62. ^{1}H NMR (400 MHz, room temperature, $CDCl_3$) spectra of product **24**

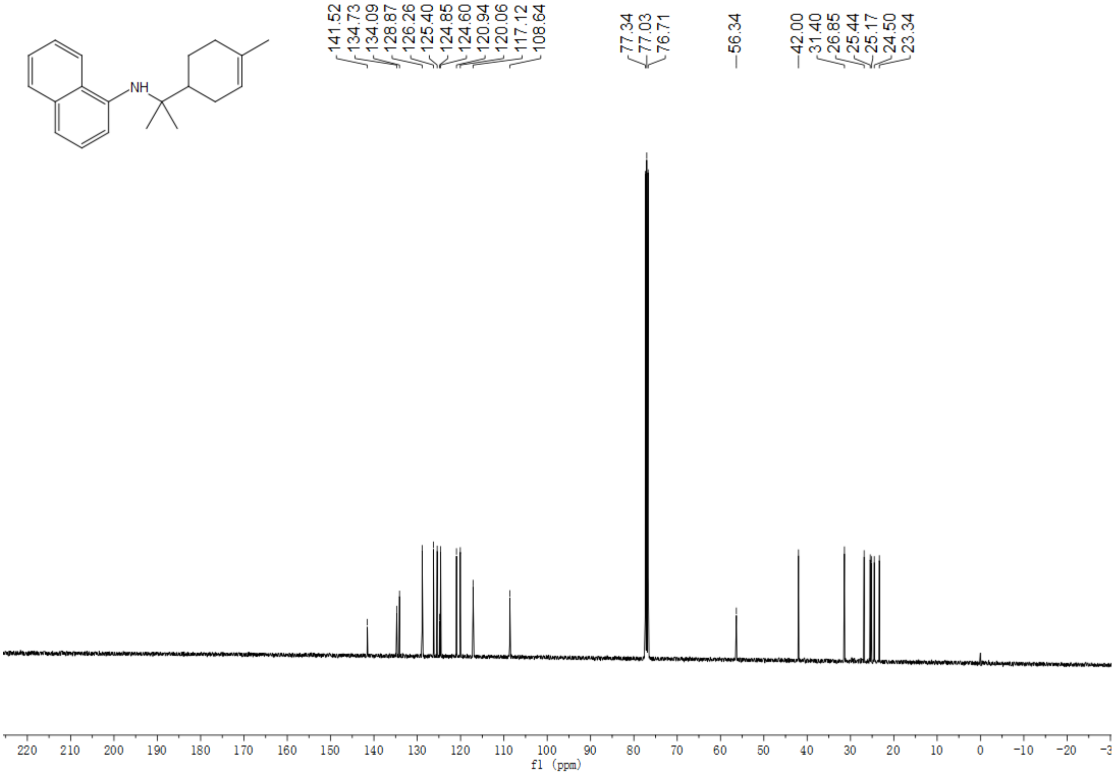

Supplementary Figure 63. ^{13}C NMR (101 MHz, room temperature, $CDCl_3$) spectra of product **24**

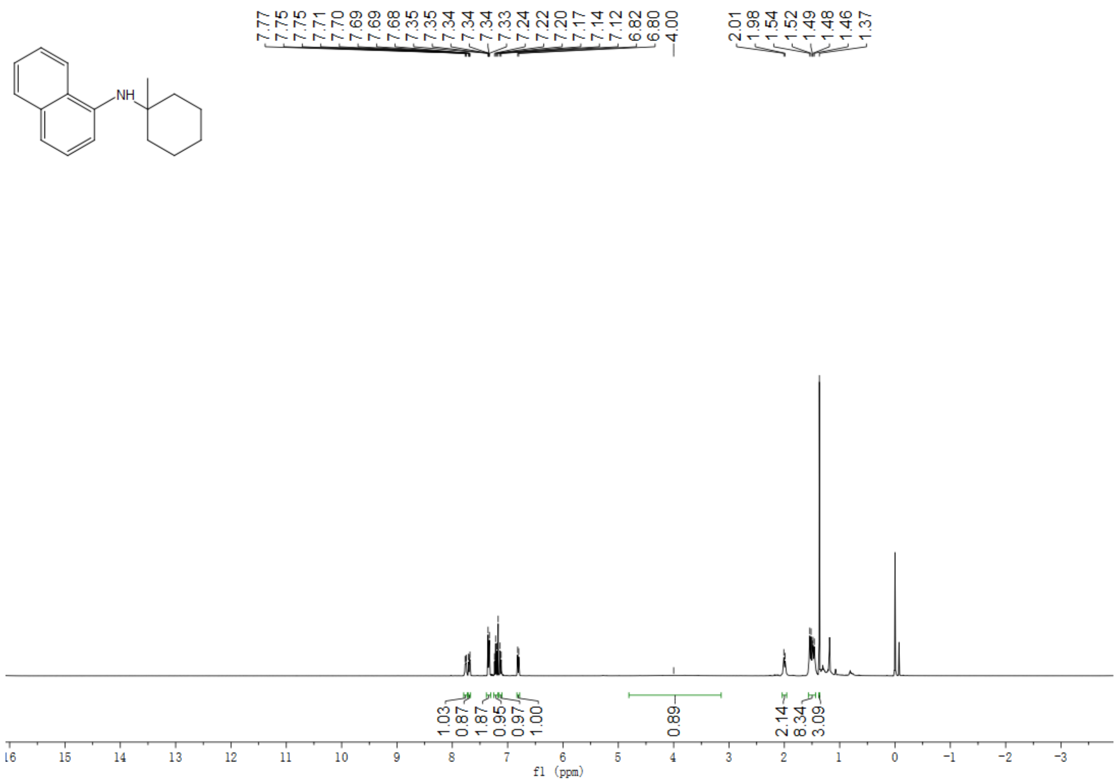

Supplementary Figure 64. ^{1}H NMR (400 MHz, room temperature, $CDCl_3$) spectra of product **25**

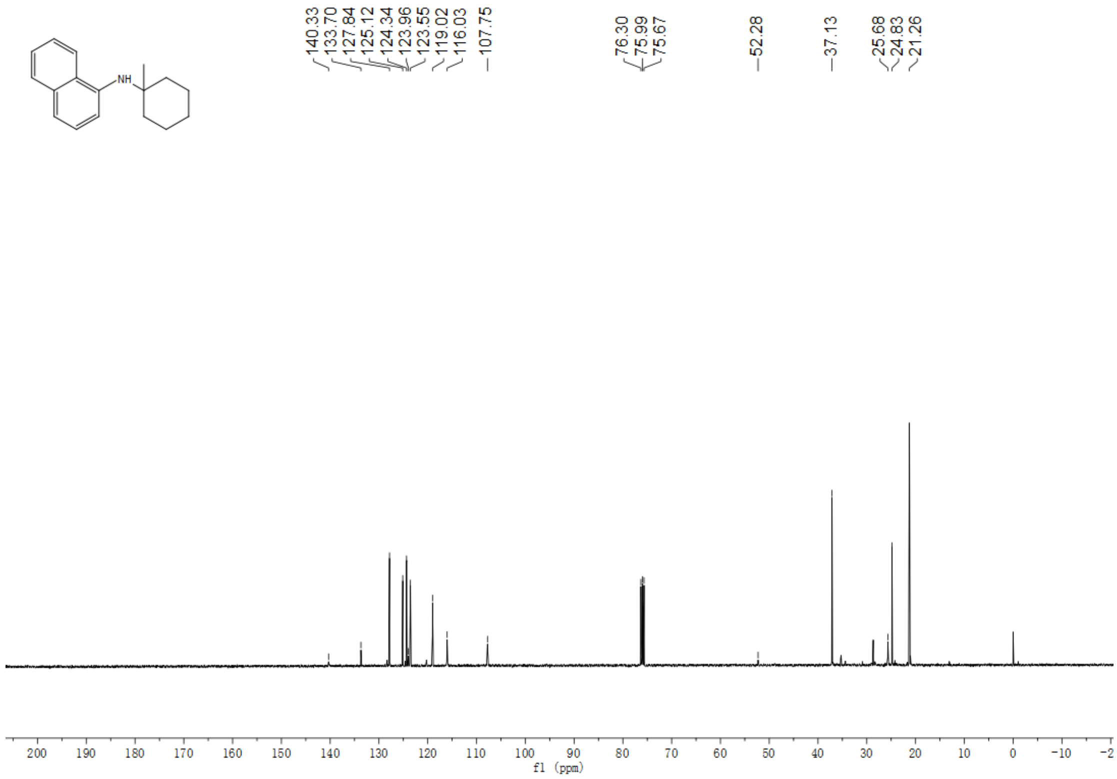

Supplementary Figure 65. ^{13}C NMR (101 MHz, room temperature, $CDCl_3$) spectra of product **25**

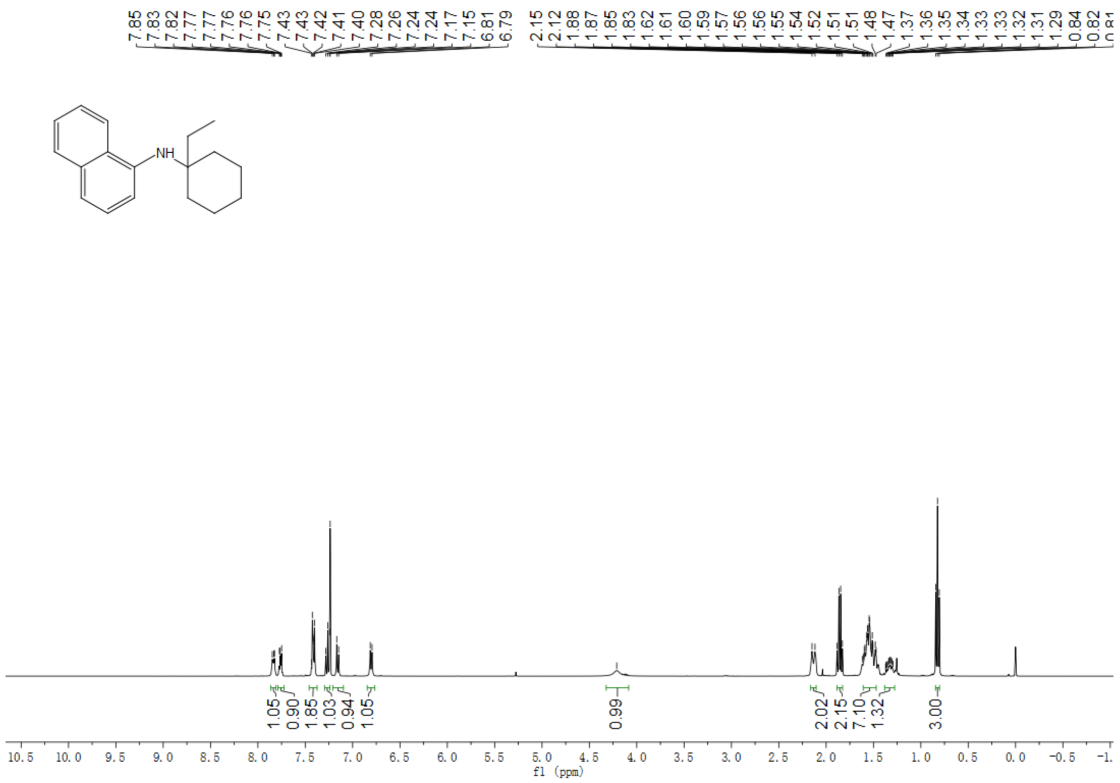

Supplementary Figure 66. ^{1}H NMR (400 MHz, room temperature, $CDCl_3$) spectra of product **26**

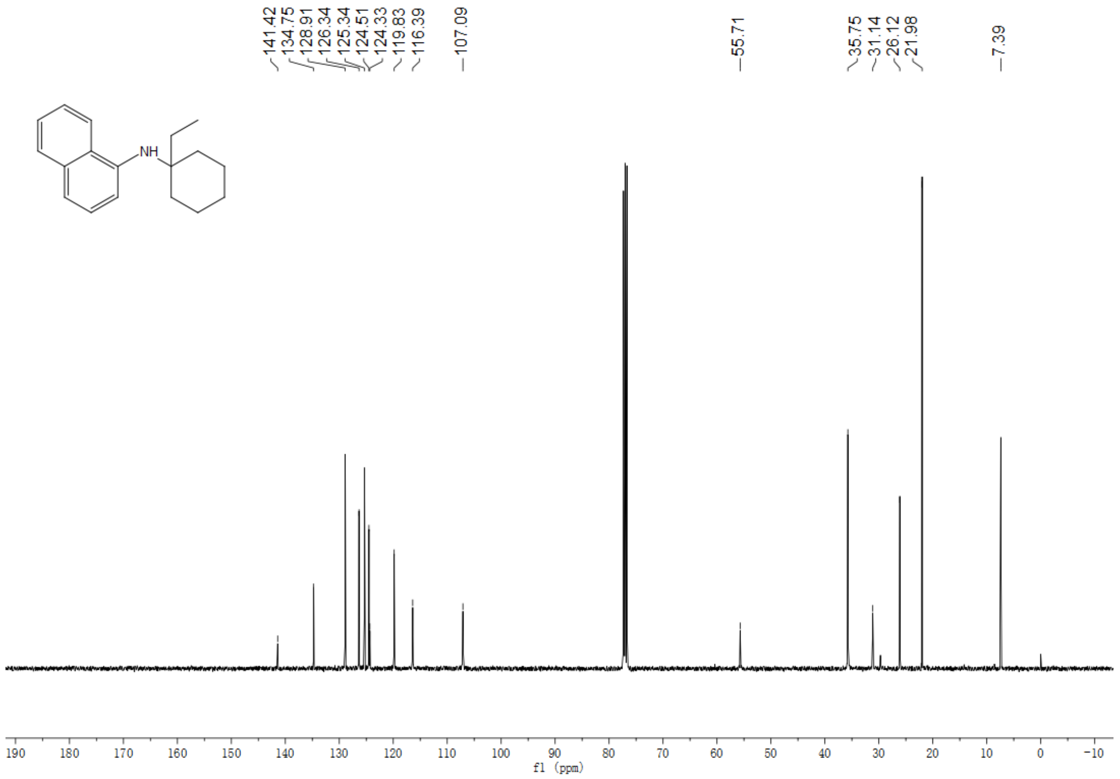

Supplementary Figure 67. ^{13}C NMR (101 MHz, room temperature, $CDCl_3$) spectra of product **26**

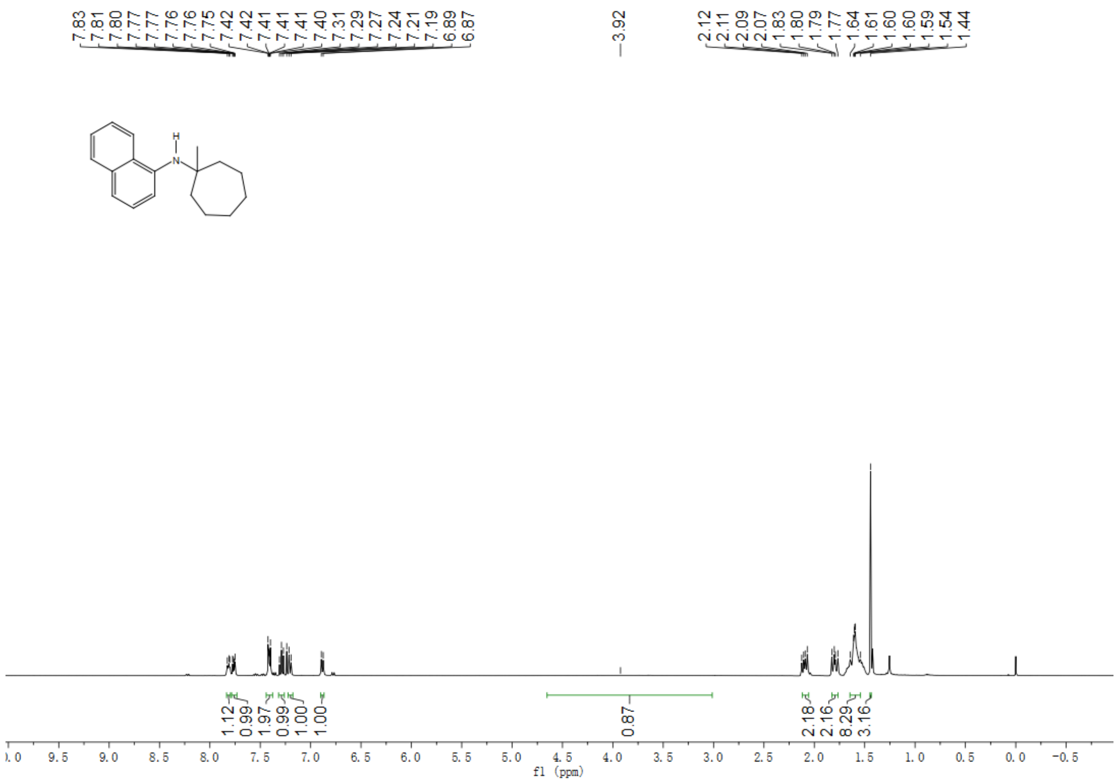

Supplementary Figure 68. ^{1}H NMR (400 MHz, room temperature, $CDCl_3$) spectra of product **27**

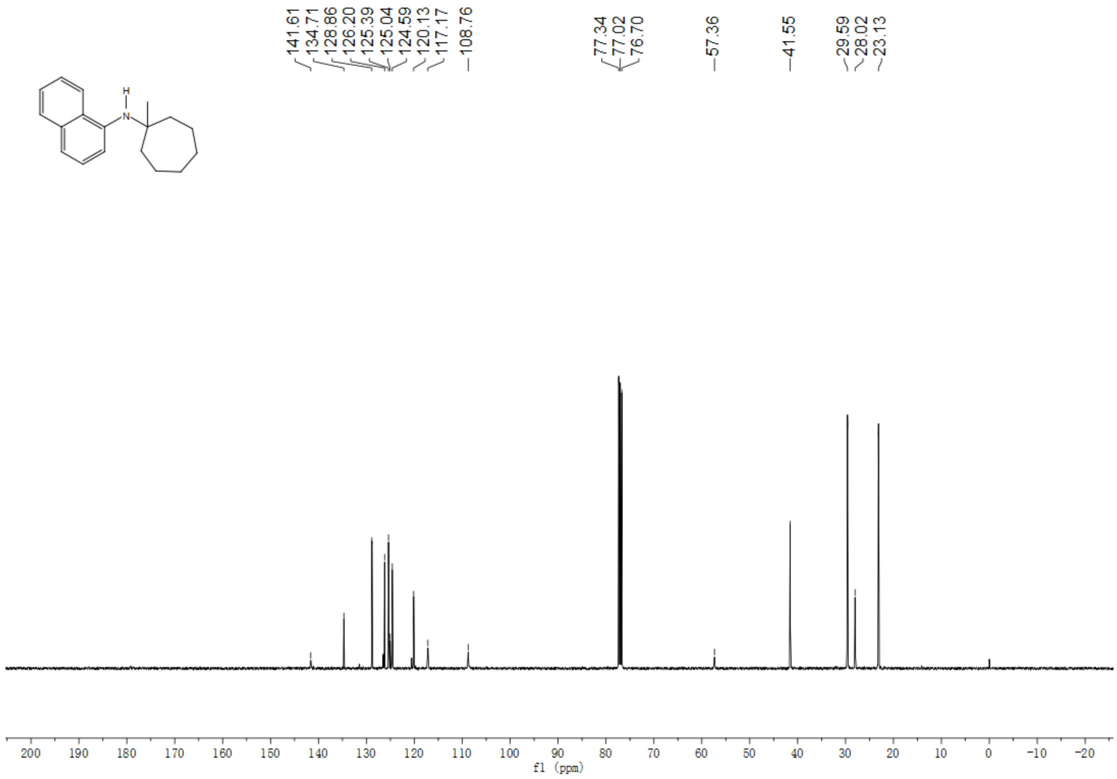

Supplementary Figure 69. ^{13}C NMR (101 MHz, room temperature, $CDCl_3$) spectra of product **27**

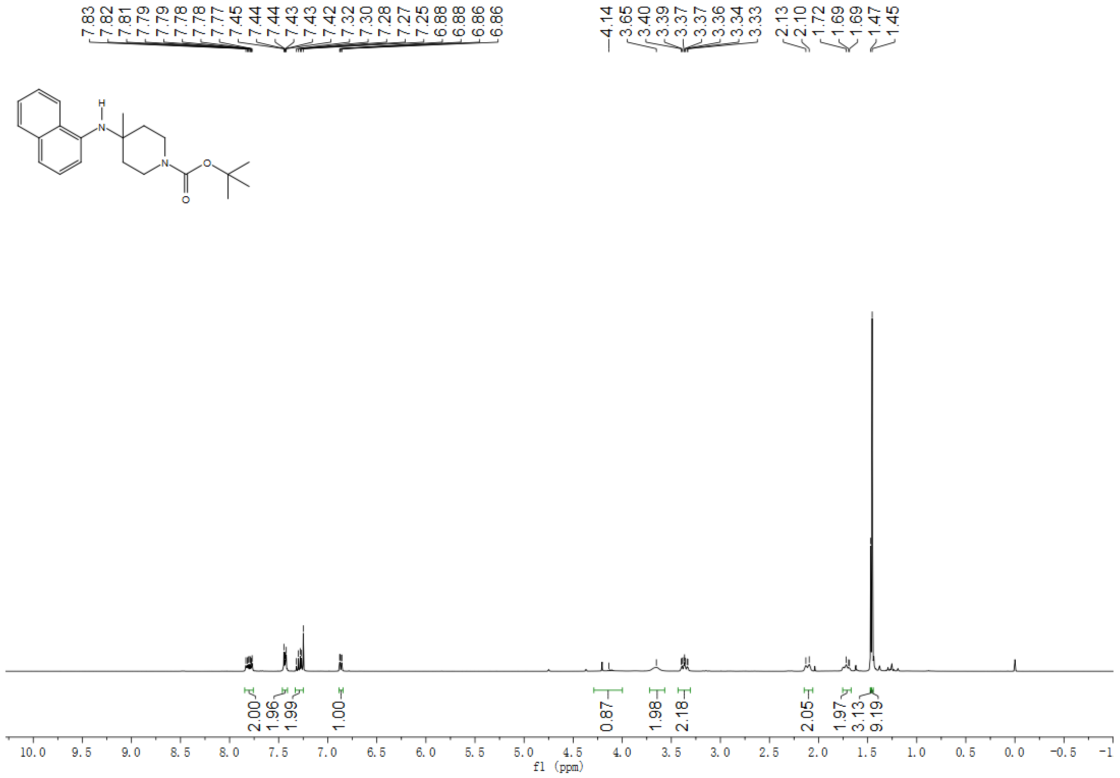

Supplementary Figure 70. ^{1}H NMR (400 MHz, room temperature, $CDCl_3$) spectra of product **28**

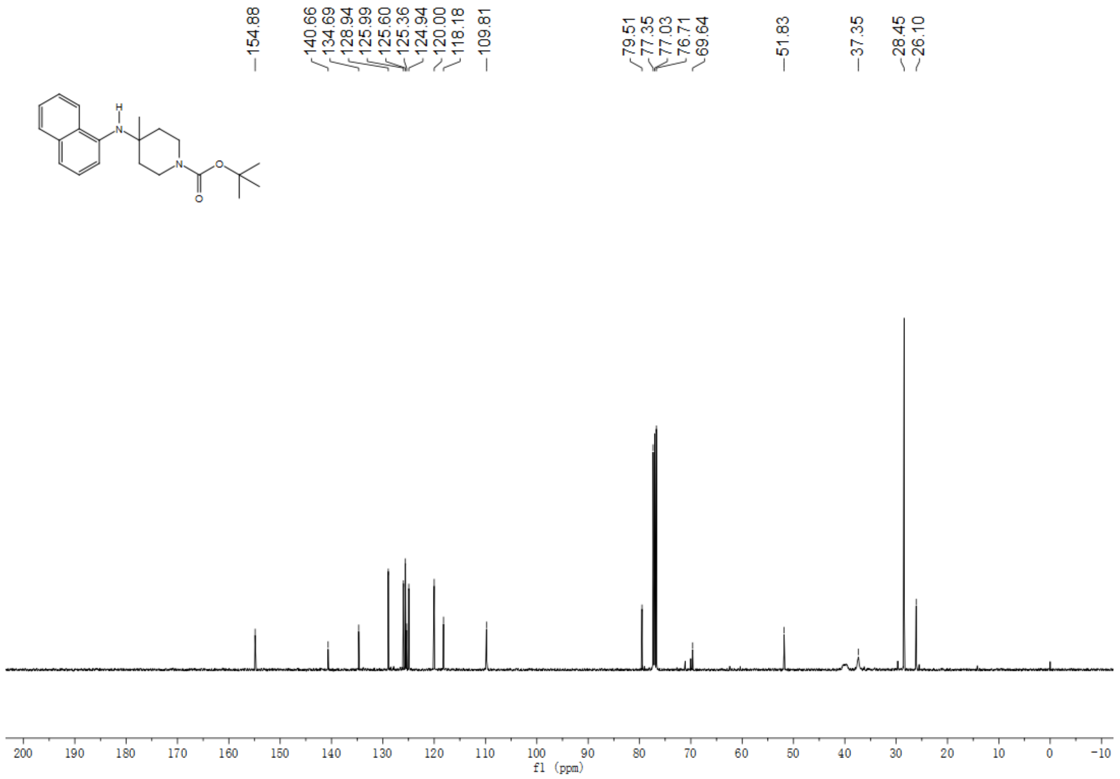

Supplementary Figure 71. ^{13}C NMR (101 MHz, room temperature, $CDCl_3$) spectra of product **28**

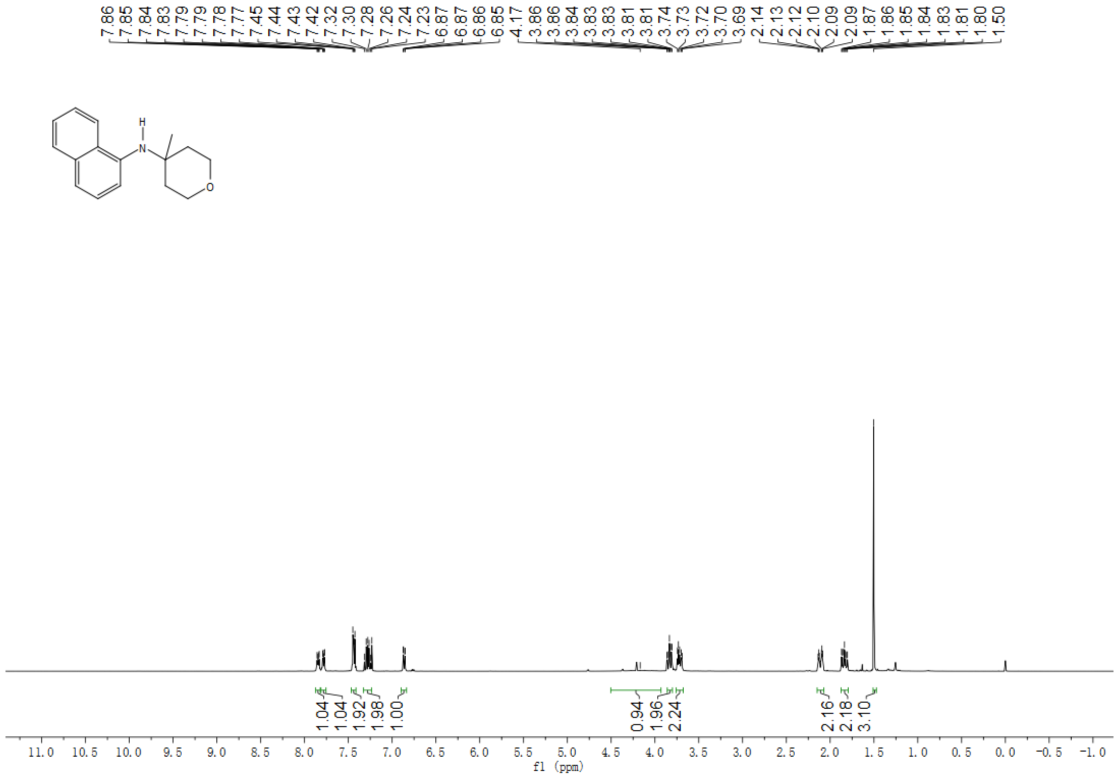

Supplementary Figure 72. ^{1}H NMR (400 MHz, room temperature, $CDCl_3$) spectra of product **29**

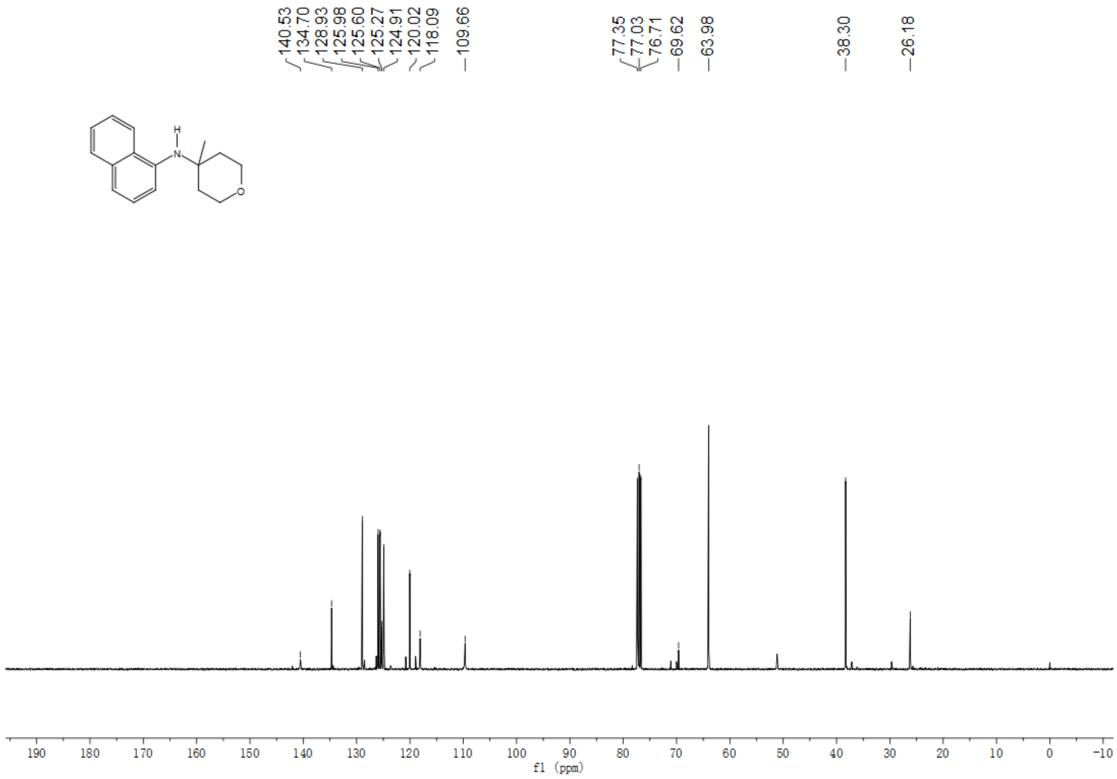

Supplementary Figure 73. ^{13}C NMR (101 MHz, room temperature, $CDCl_3$) spectra of product **29**

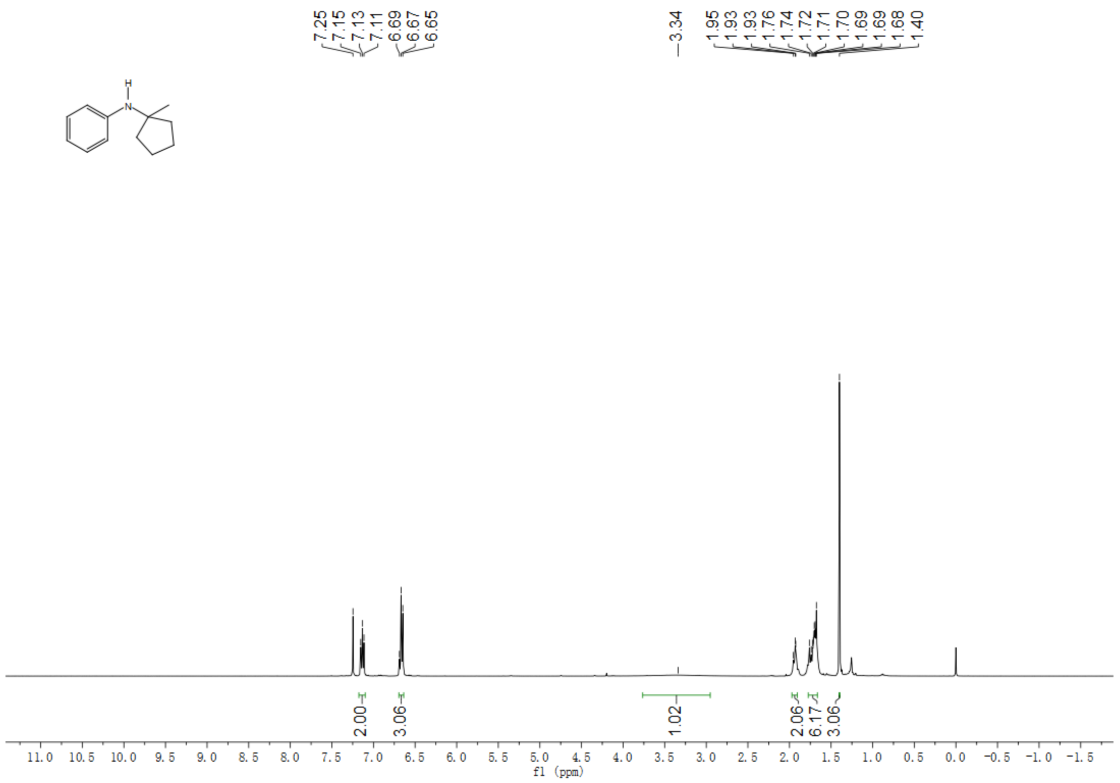

Supplementary Figure 74. ^{1}H NMR (400 MHz, room temperature, $CDCl_3$) spectra of product **30**

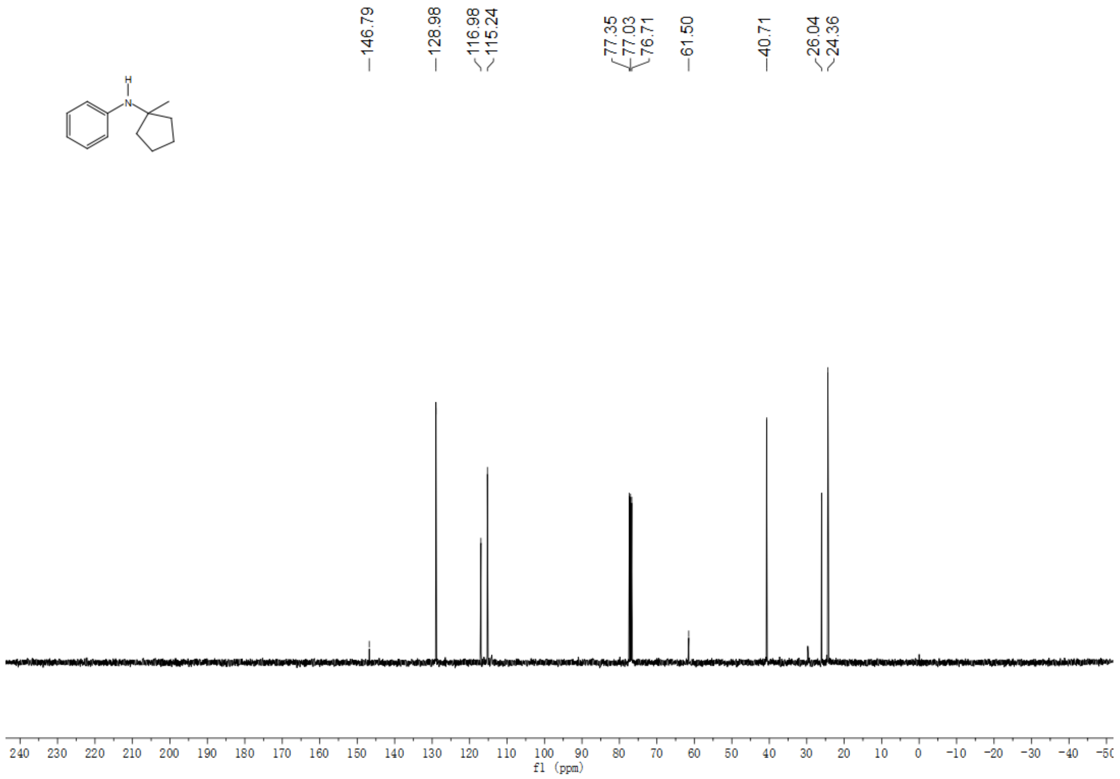

Supplementary Figure 75. ^{13}C NMR (101 MHz, room temperature, $CDCl_3$) spectra of product **30**

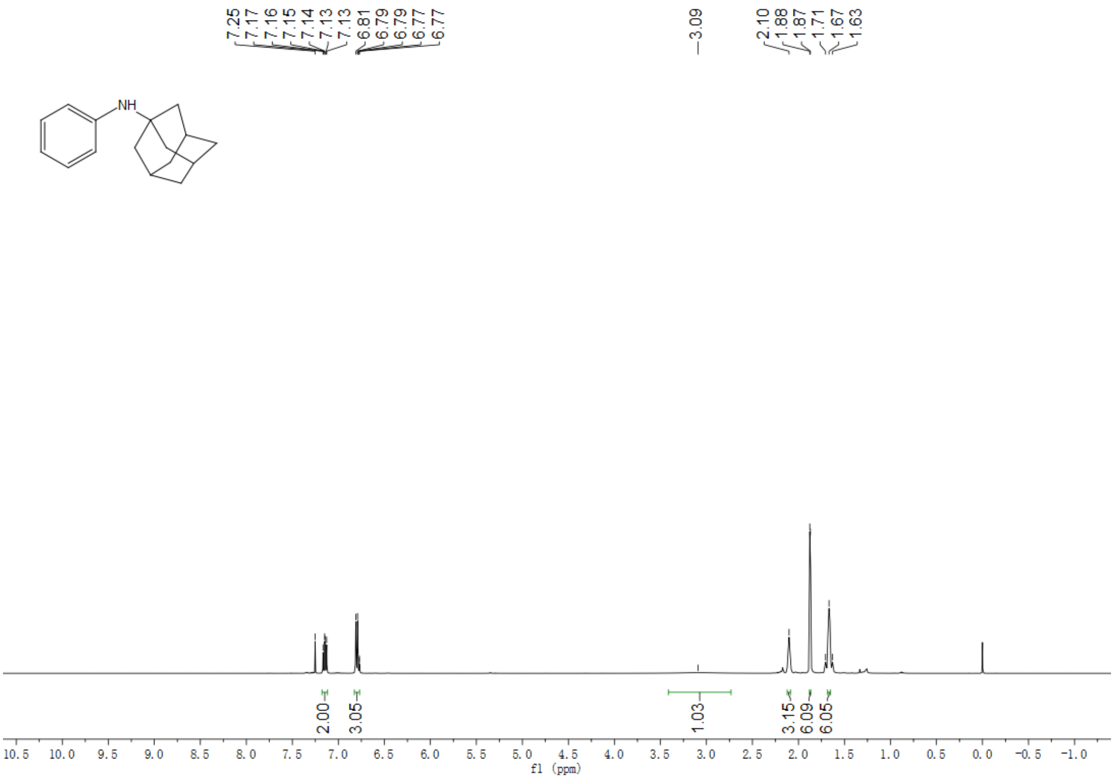

Supplementary Figure 76. ^{1}H NMR (400 MHz, room temperature, $CDCl_3$) spectra of product **31**

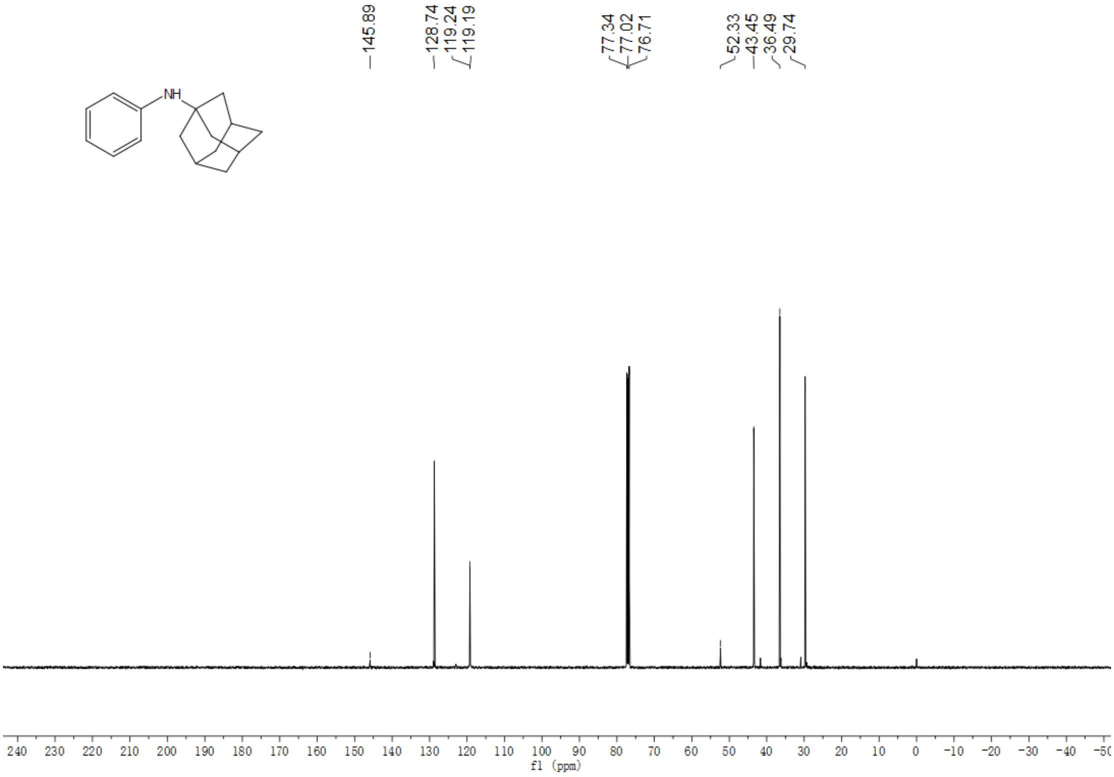

Supplementary Figure 77. ^{13}C NMR (101 MHz, room temperature, $CDCl_3$) spectra of product **31**

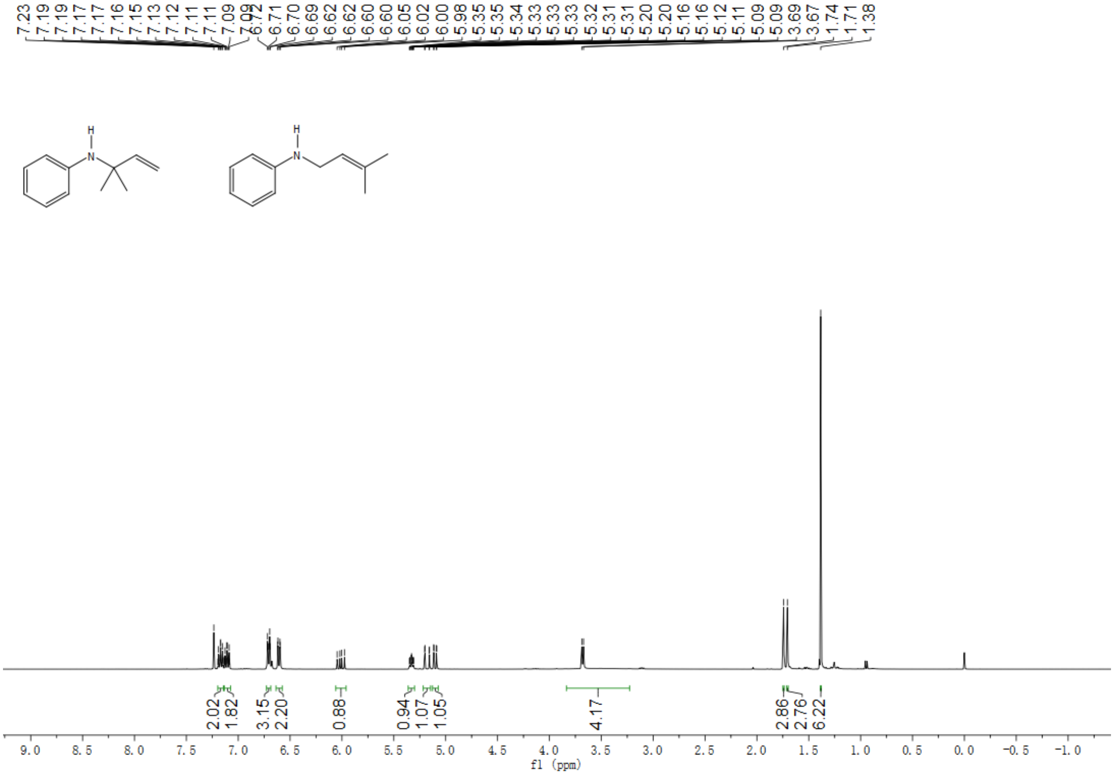

Supplementary Figure 78. ^{1}H NMR (400 MHz, room temperature, $CDCl_3$) spectra of product **32**

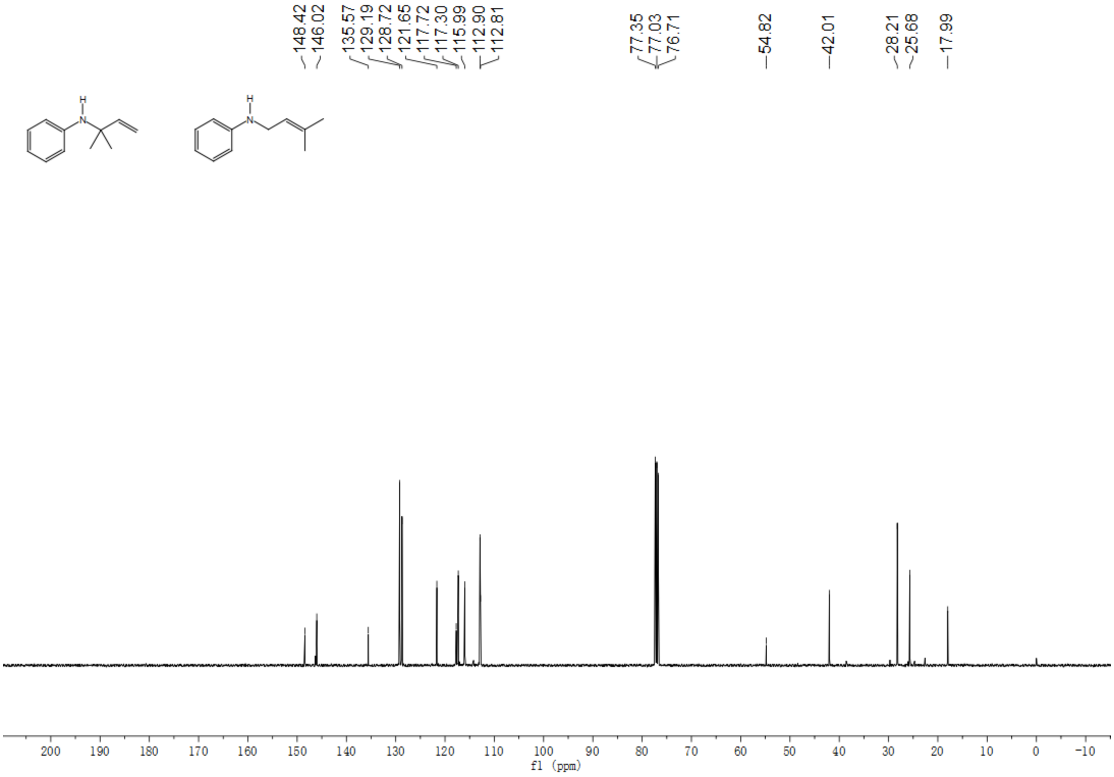

Supplementary Figure 79. ^{13}C NMR (101 MHz, room temperature, $CDCl_3$) spectra of product **32**

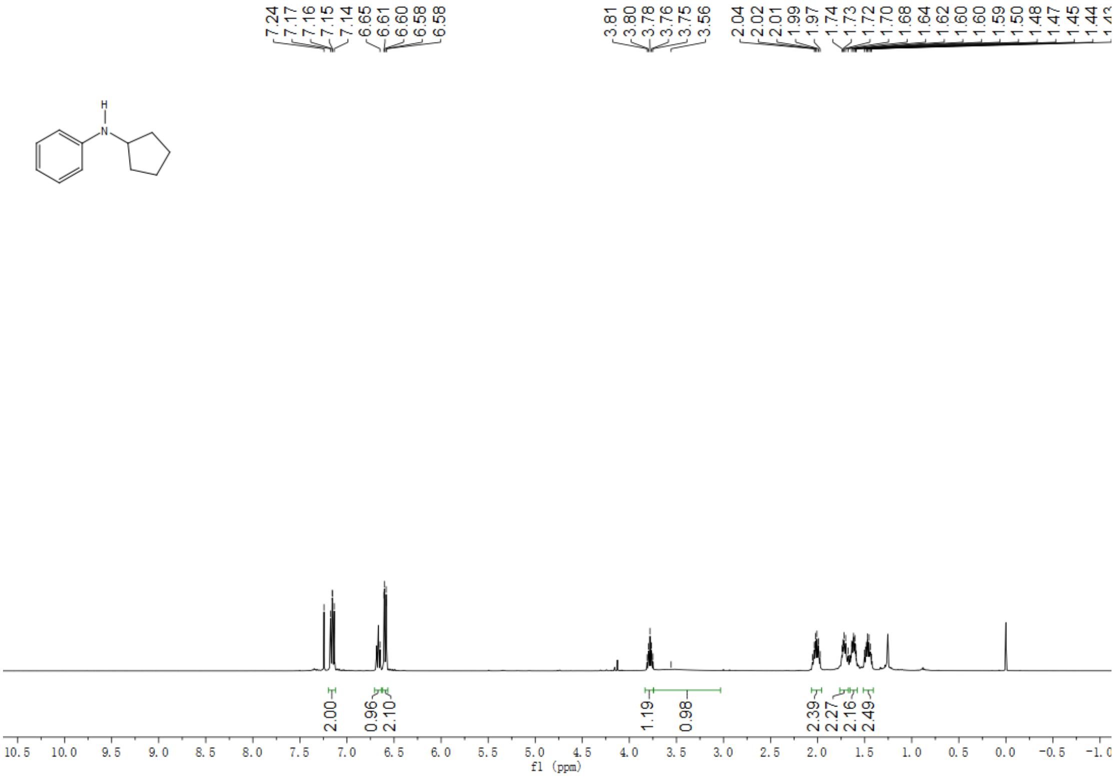

Supplementary Figure 80. ^{1}H NMR (400 MHz, room temperature, $CDCl_3$) spectra of product **33**

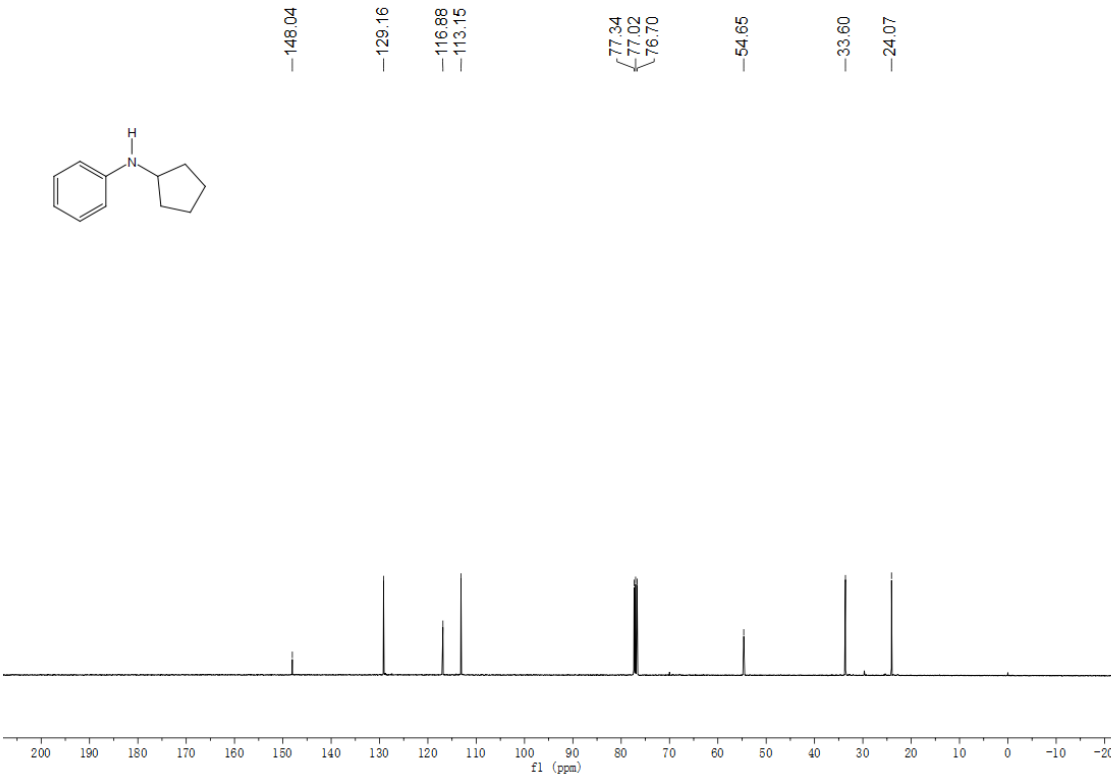

Supplementary Figure 81. ^{13}C NMR (101 MHz, room temperature, $CDCl_3$) spectra of product **33**

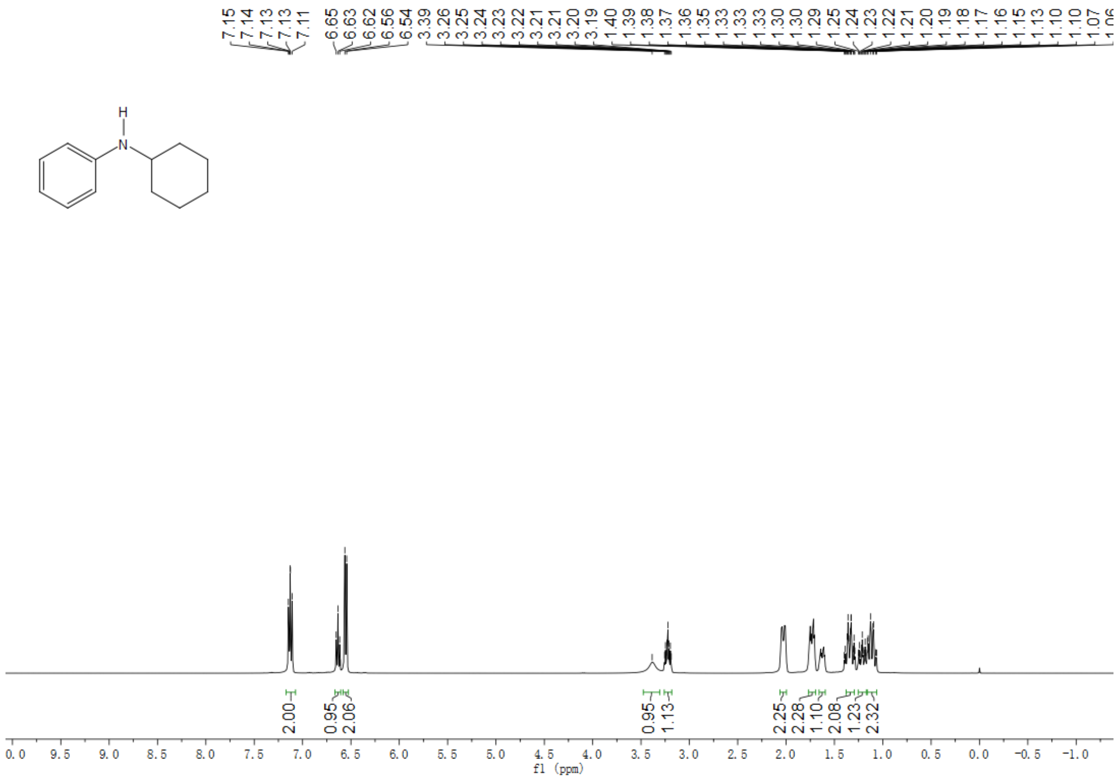

Supplementary Figure 82. ^{1}H NMR (400 MHz, room temperature, $CDCl_3$) spectra of product **34**

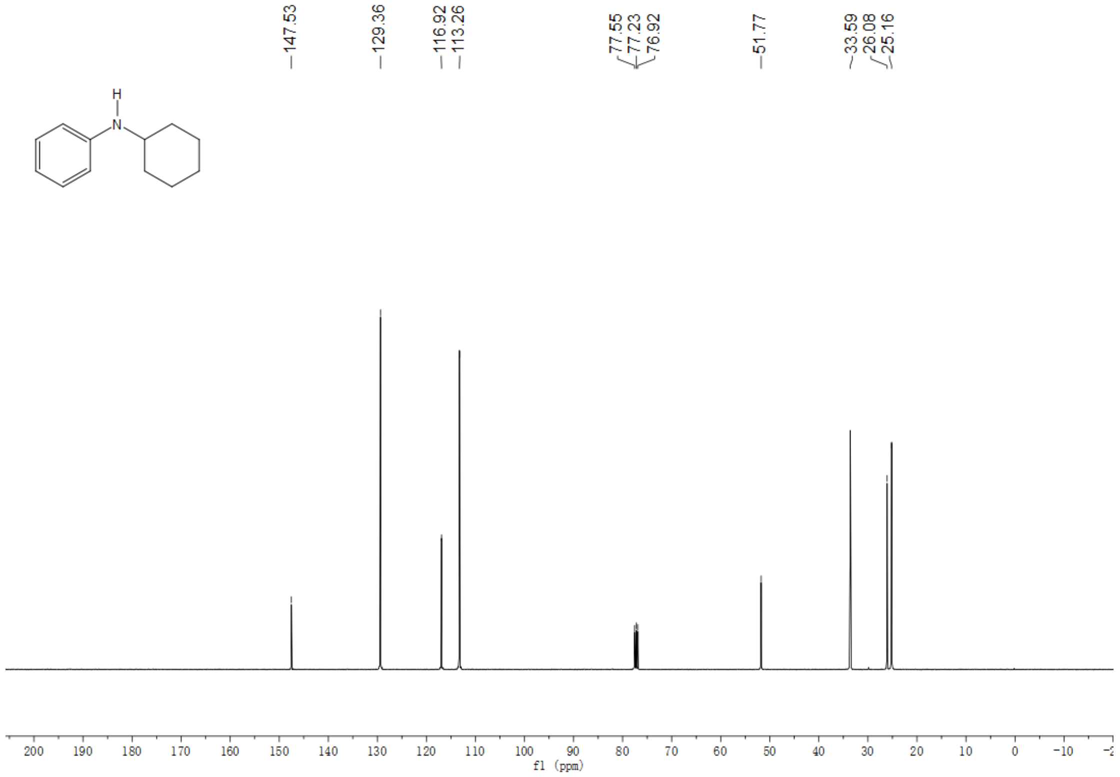

Supplementary Figure 83. ^{13}C NMR (101 MHz, room temperature, $CDCl_3$) spectra of product **34**

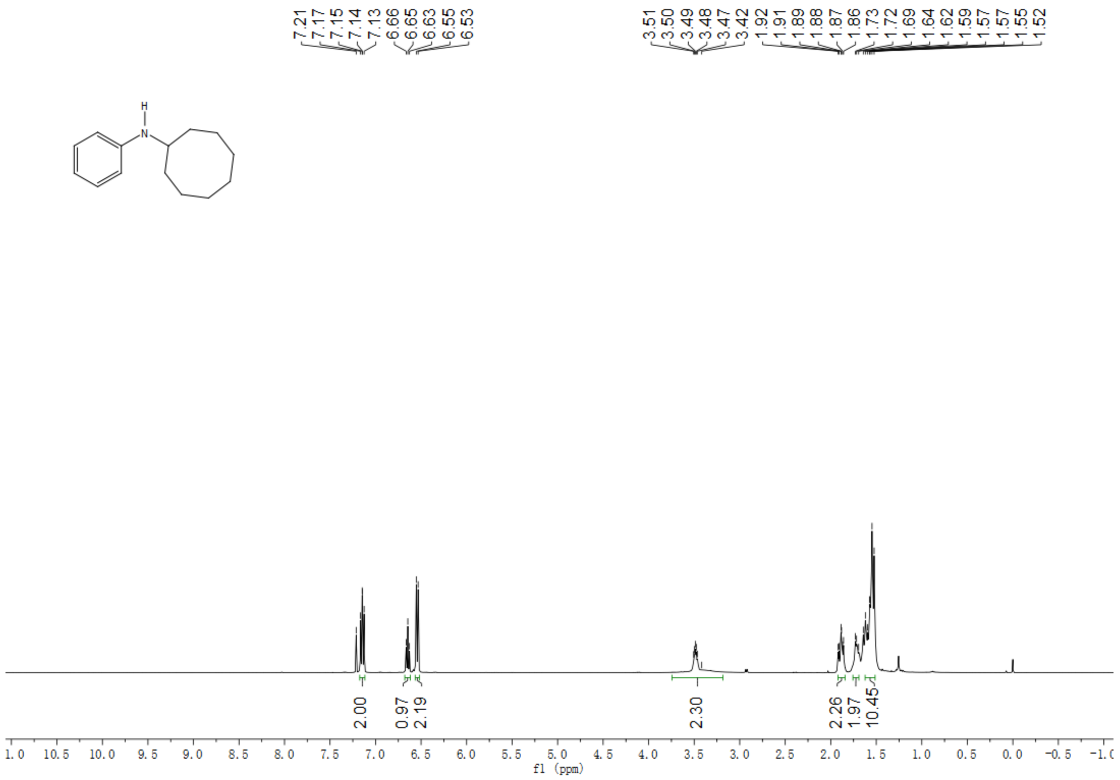

Supplementary Figure 84. ^{1}H NMR (400 MHz, room temperature, $CDCl_3$) spectra of product **35**

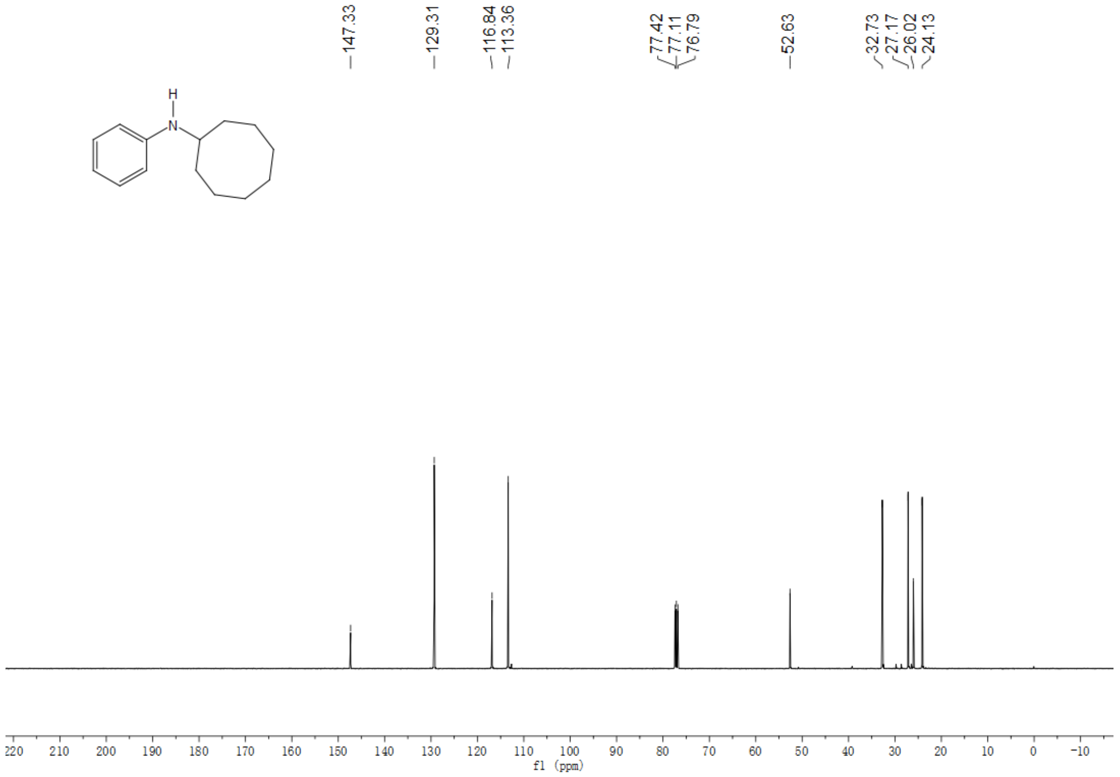

Supplementary Figure 85. ^{13}C NMR (101 MHz, room temperature, $CDCl_3$) spectra of product **35**

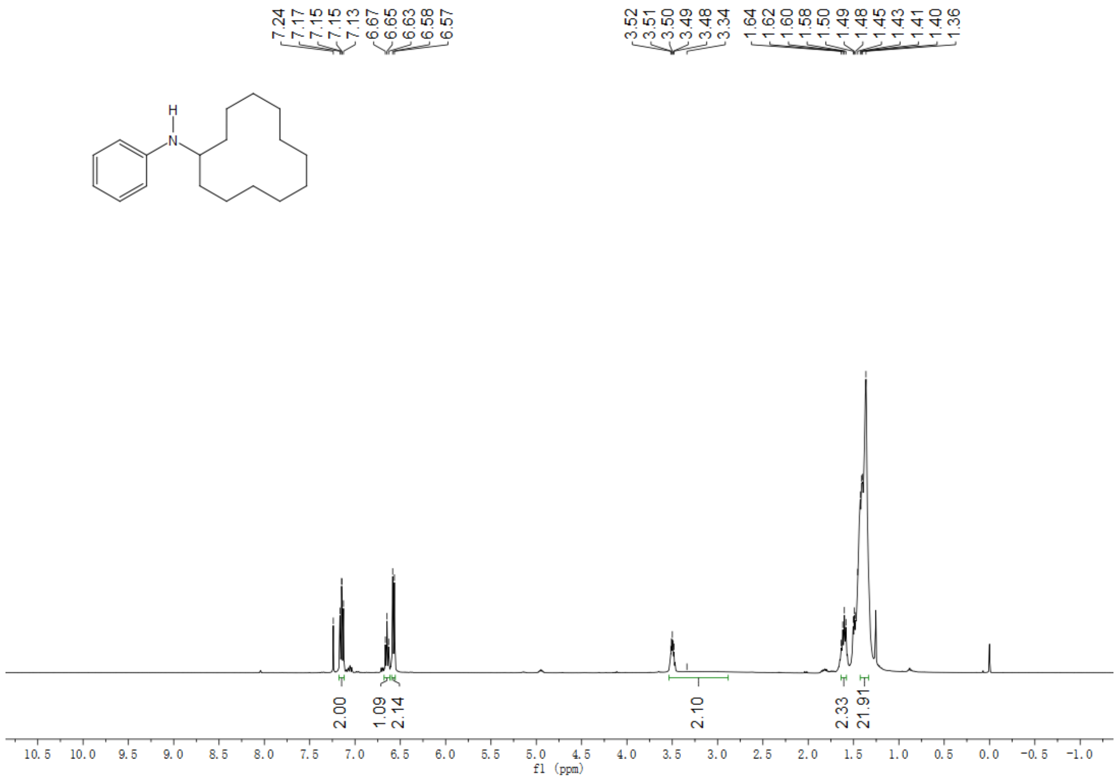

Supplementary Figure 86. ^{1}H NMR (400 MHz, room temperature, $CDCl_3$) spectra of product **36**

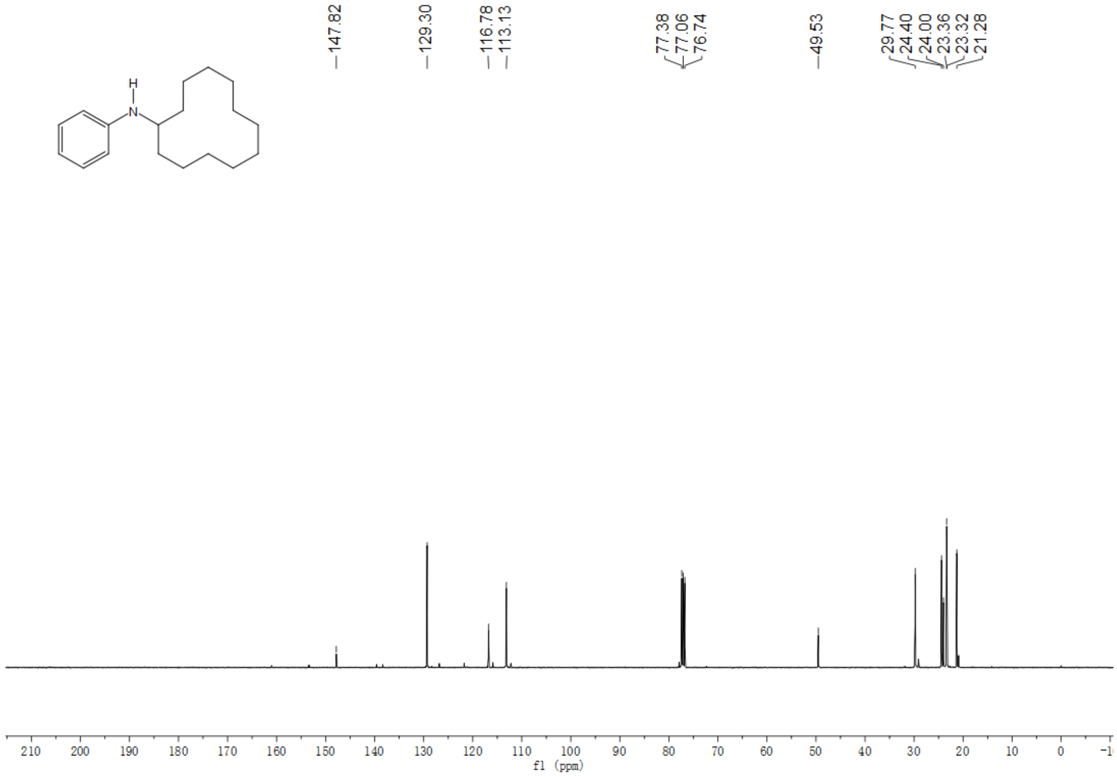

Supplementary Figure 87. ^{13}C NMR (101 MHz, room temperature, $CDCl_3$) spectra of product **36**

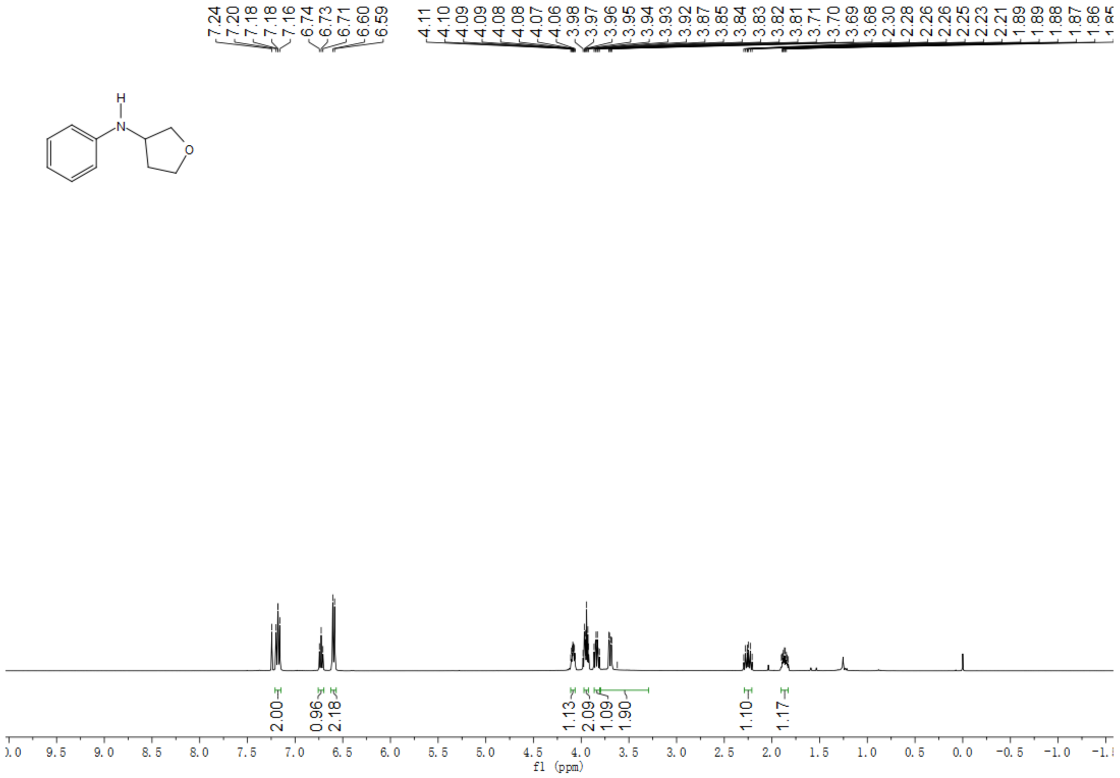

Supplementary Figure 88. ^{1}H NMR (400 MHz, room temperature, $CDCl_3$) spectra of product **37**

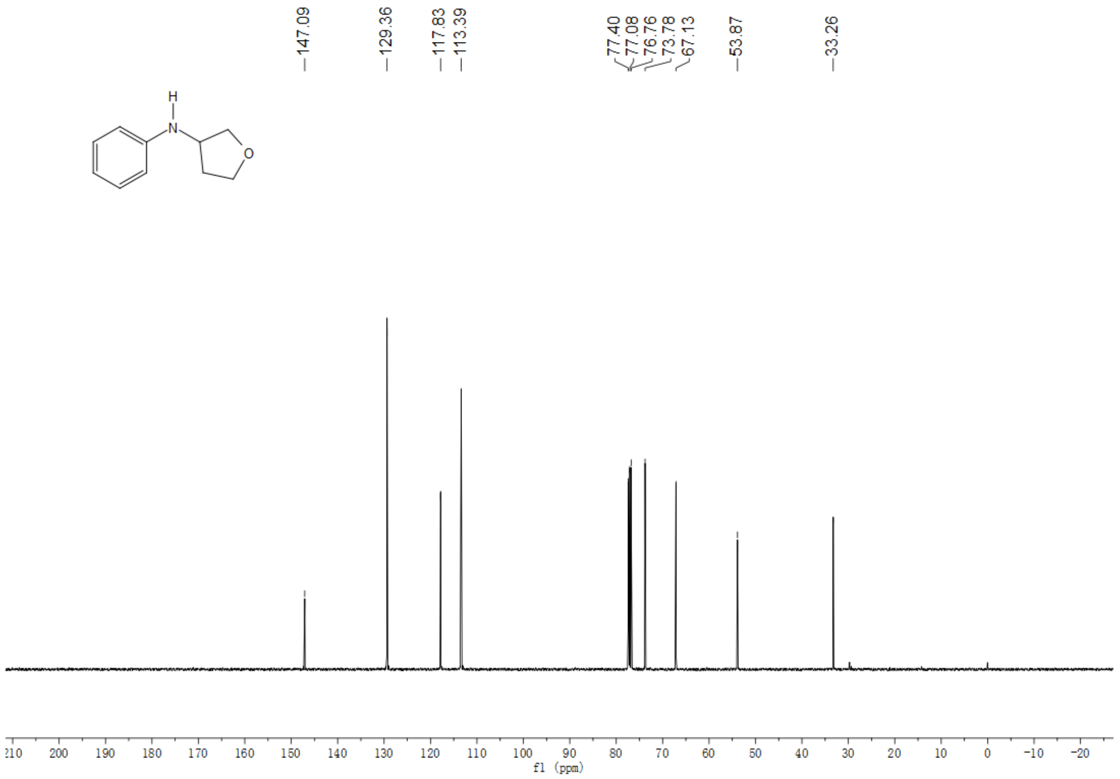

Supplementary Figure 89. ^{13}C NMR (101 MHz, room temperature, $CDCl_3$) spectra of product **37**

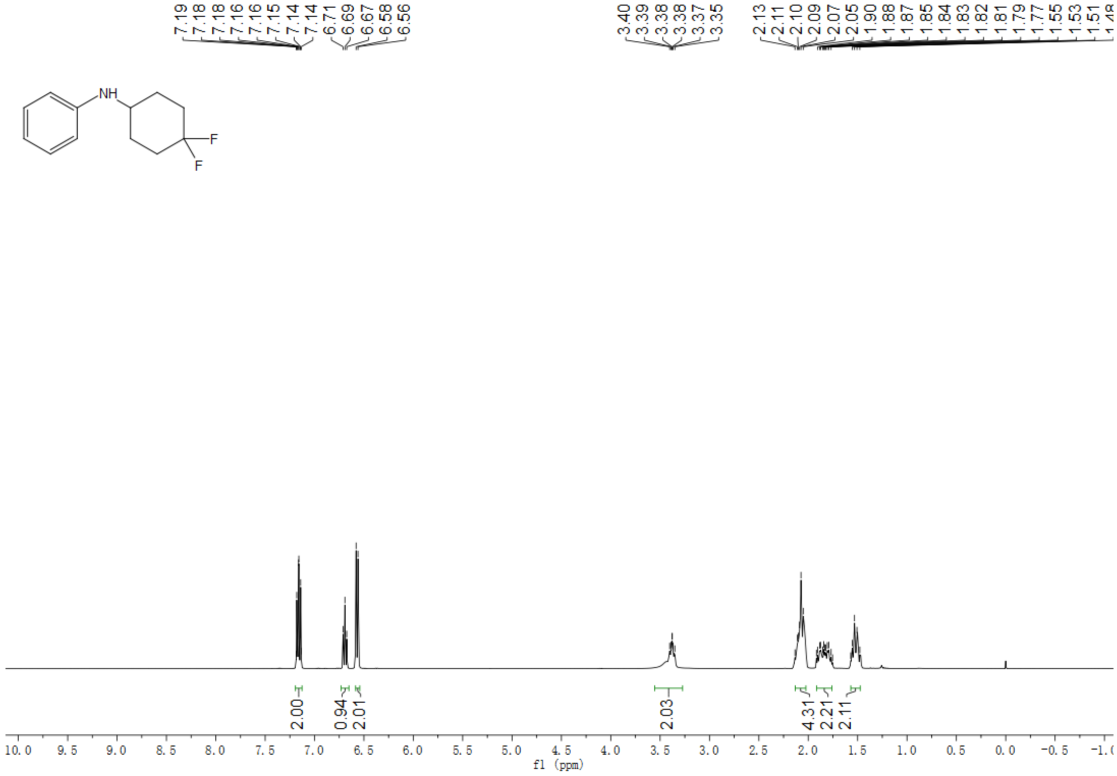

Supplementary Figure 90. ^{1}H NMR (400 MHz, room temperature, $CDCl_3$) spectra of product **38**

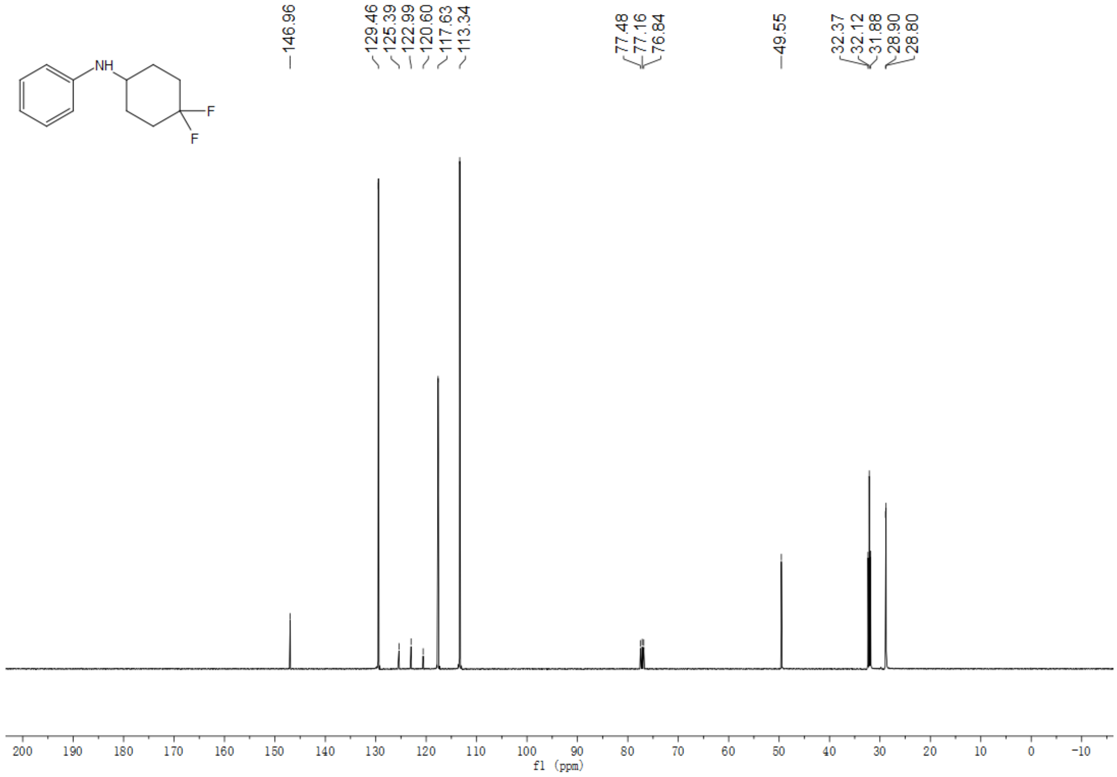

Supplementary Figure 91. ^{13}C NMR (101 MHz, room temperature, $CDCl_3$) spectra of product **38**

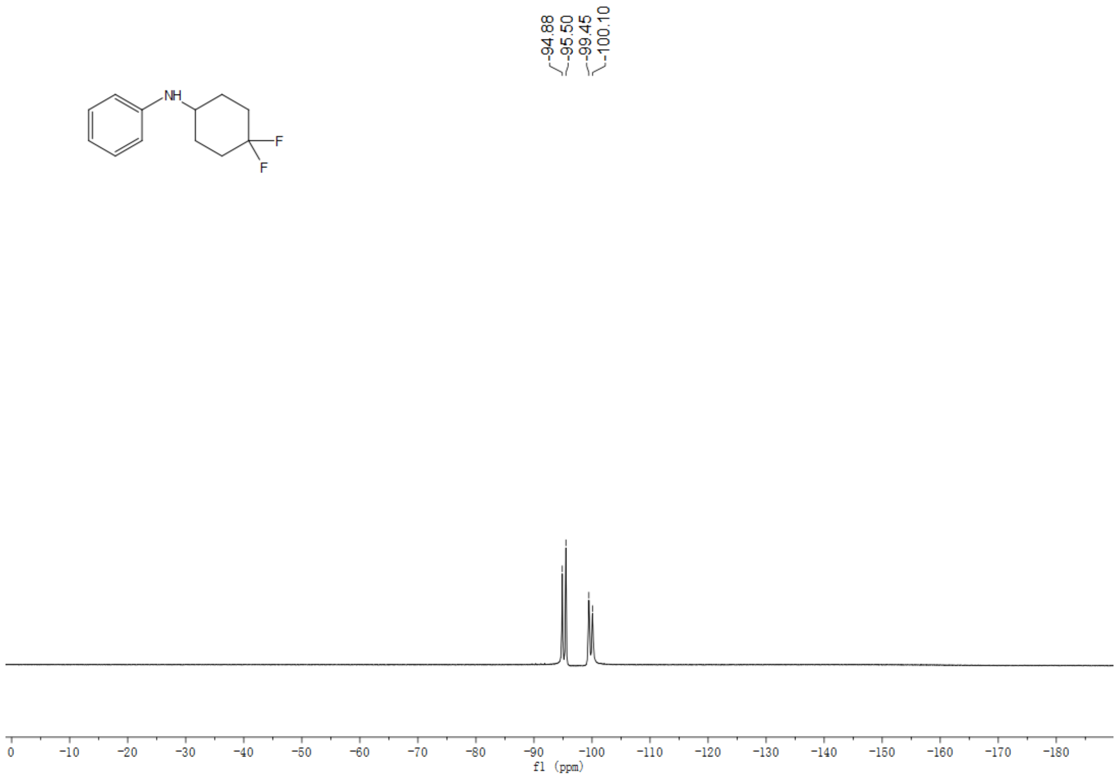

Supplementary Figure 92. ^{19}F NMR (376 MHz, room temperature, $CDCl_3$) spectra of product **38**

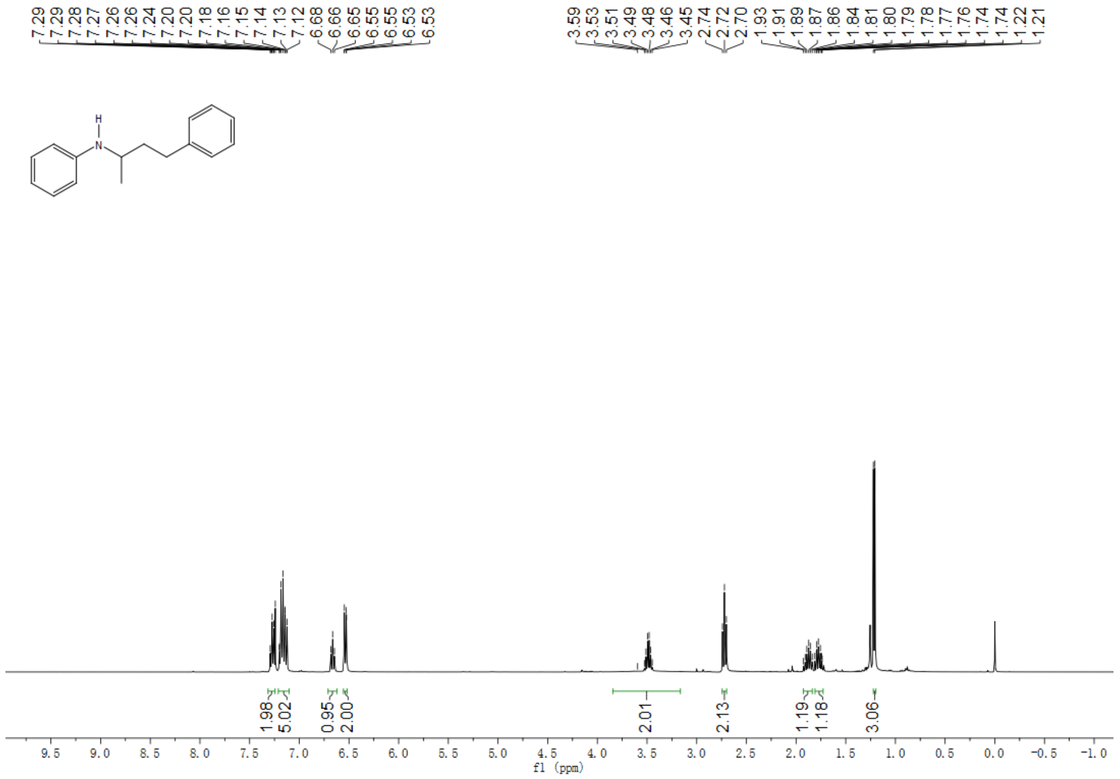

Supplementary Figure 93. ^{1}H NMR (400 MHz, room temperature, $CDCl_3$) spectra of product **39**

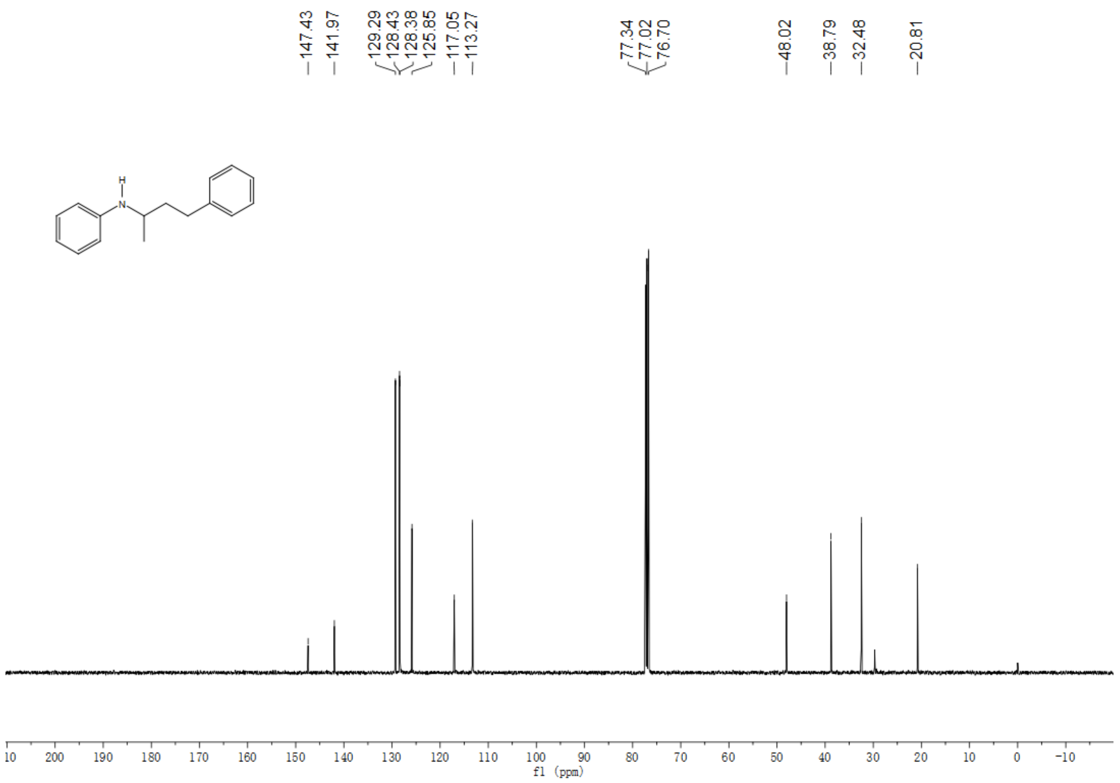

Supplementary Figure 94. ^{13}C NMR (101 MHz, room temperature, $CDCl_3$) spectra of product **39**

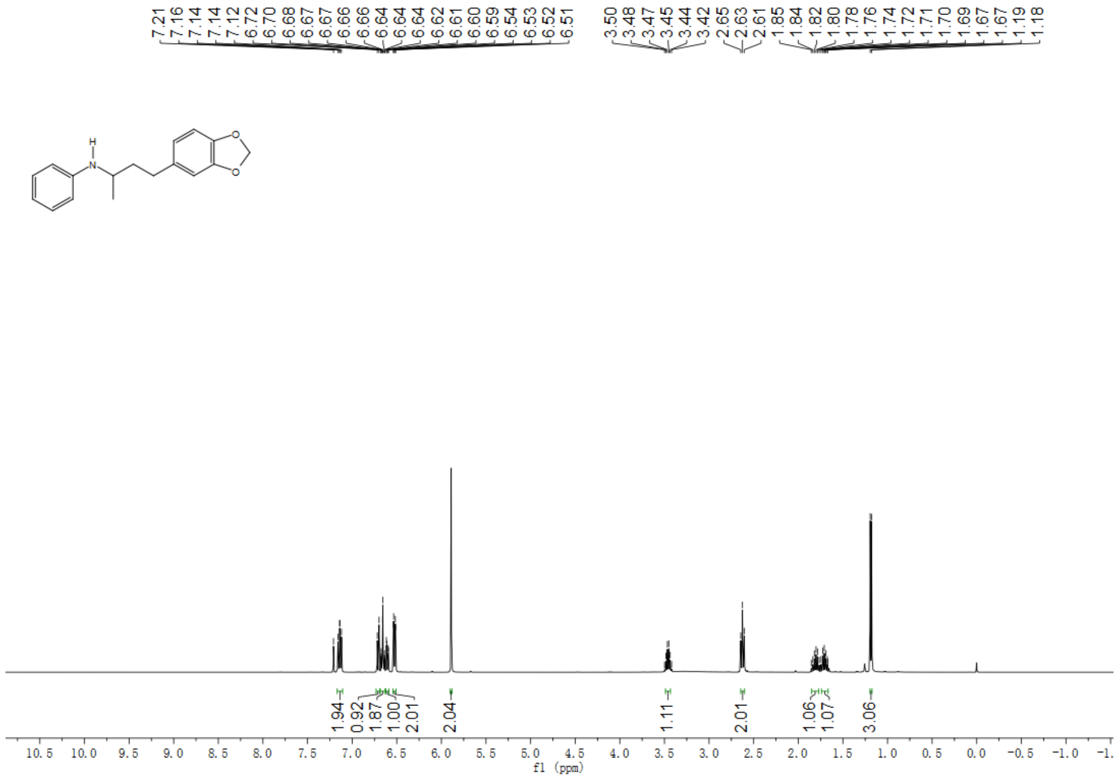

Supplementary Figure 95. ^{1}H NMR (400 MHz, room temperature, $CDCl_3$) spectra of product **40**

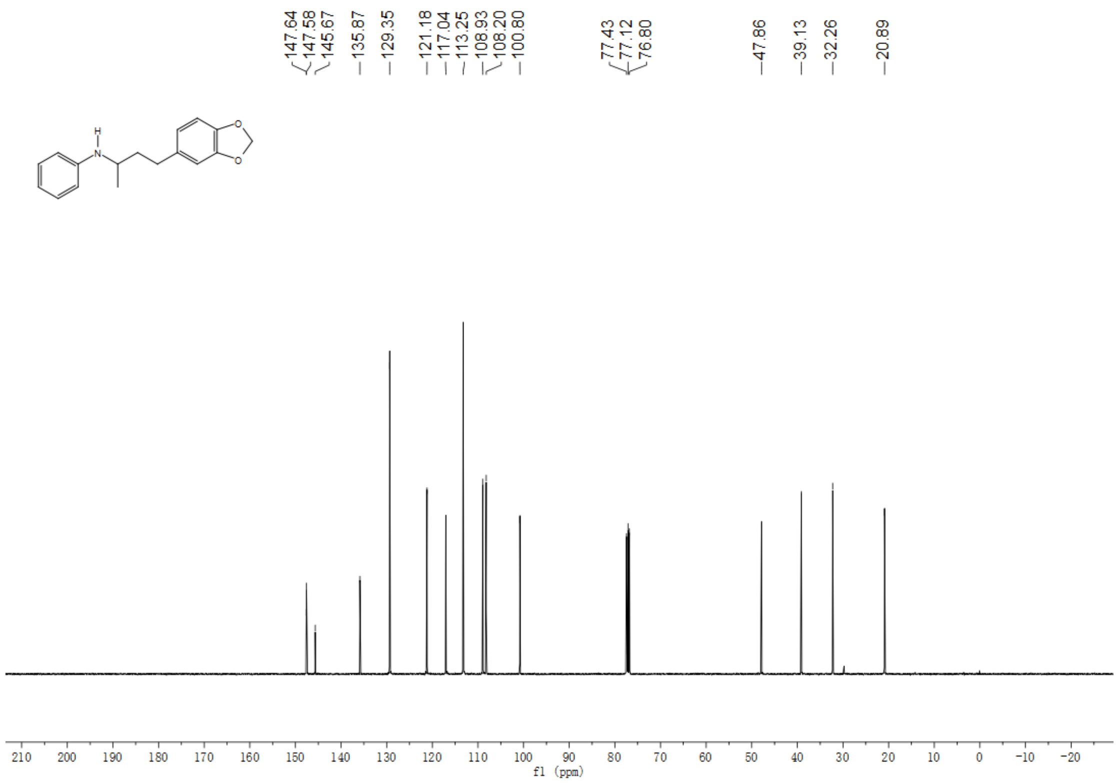

Supplementary Figure 96. ^{13}C NMR (101 MHz, room temperature, $CDCl_3$) spectra of product **40**

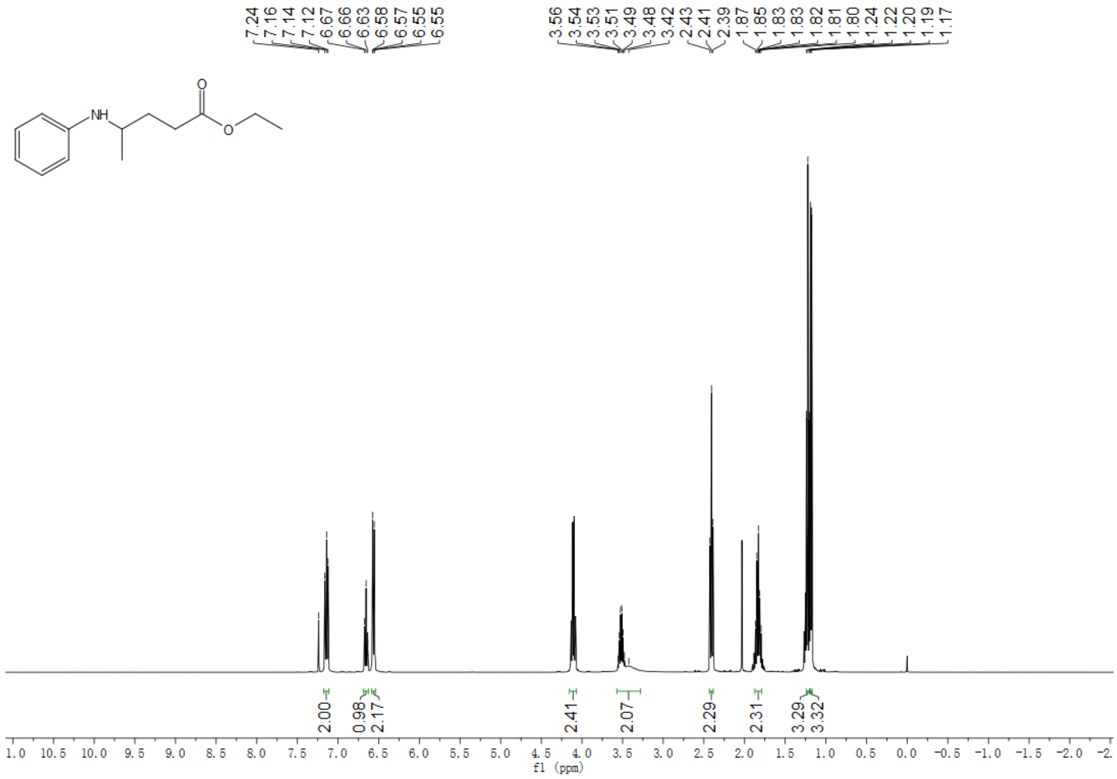

Supplementary Figure 97. ^{1}H NMR (400 MHz, room temperature, $CDCl_3$) spectra of product **41**

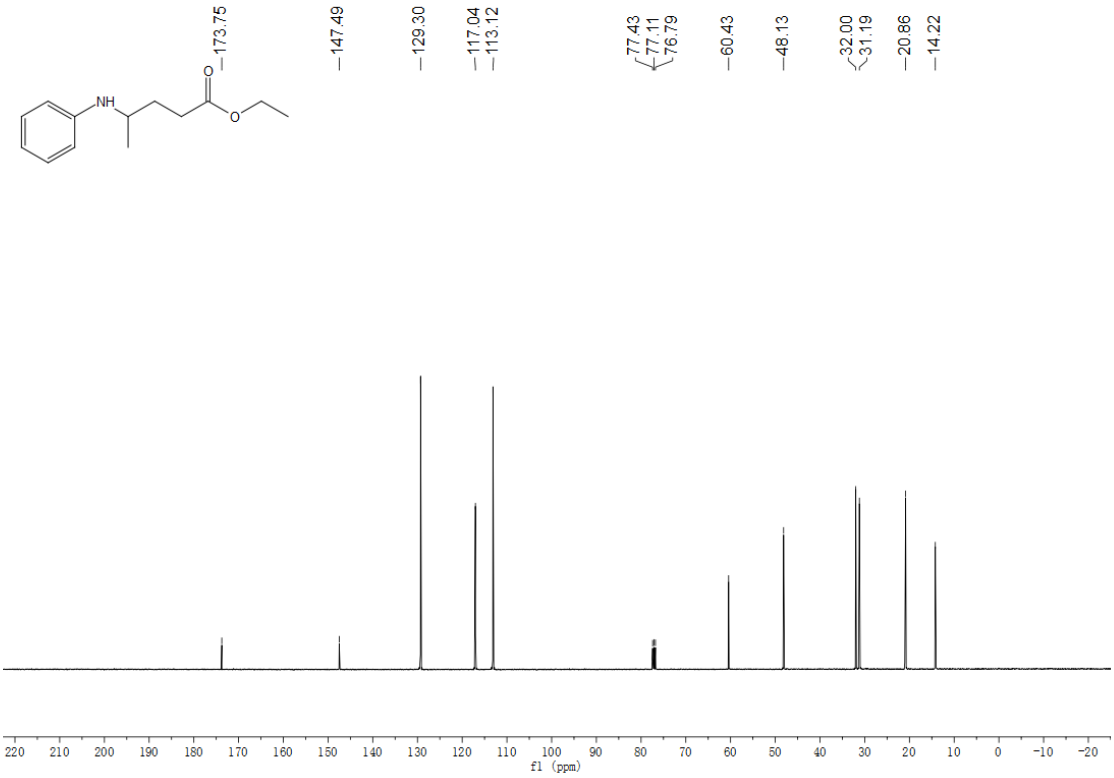

Supplementary Figure 98. ^{13}C NMR (101 MHz, room temperature, $CDCl_3$) spectra of product **41**

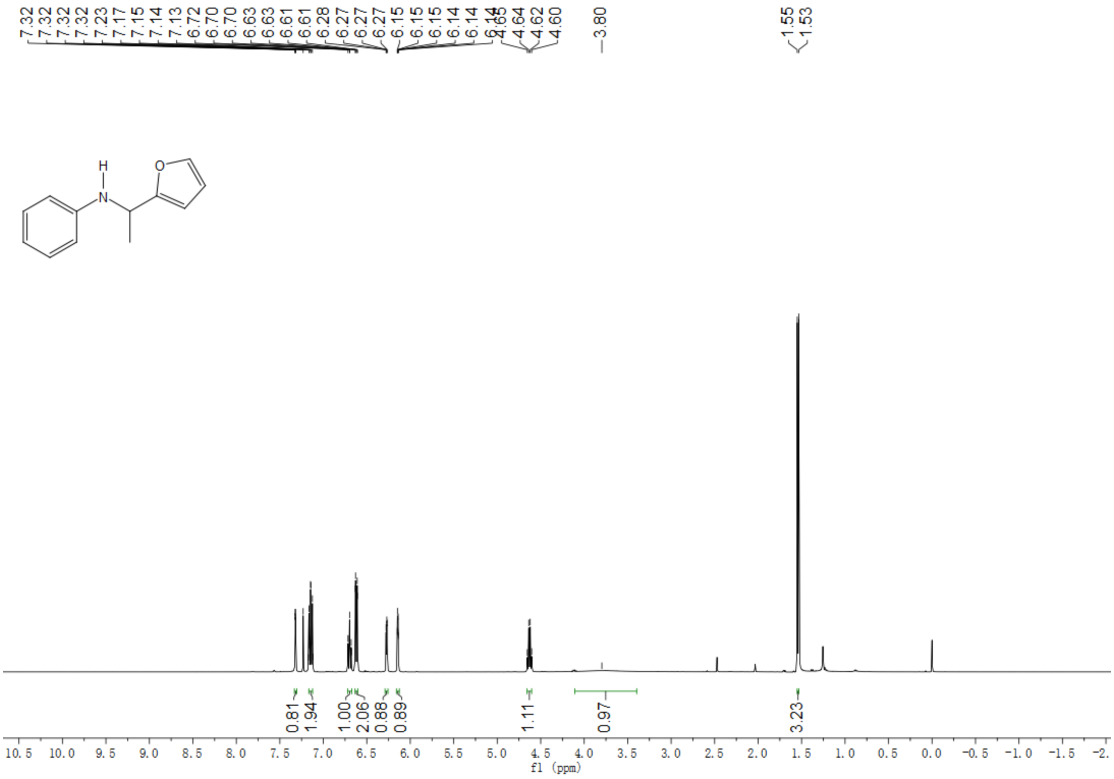

Supplementary Figure 99. ^{1}H NMR (400 MHz, room temperature, $CDCl_3$) spectra of product **42**

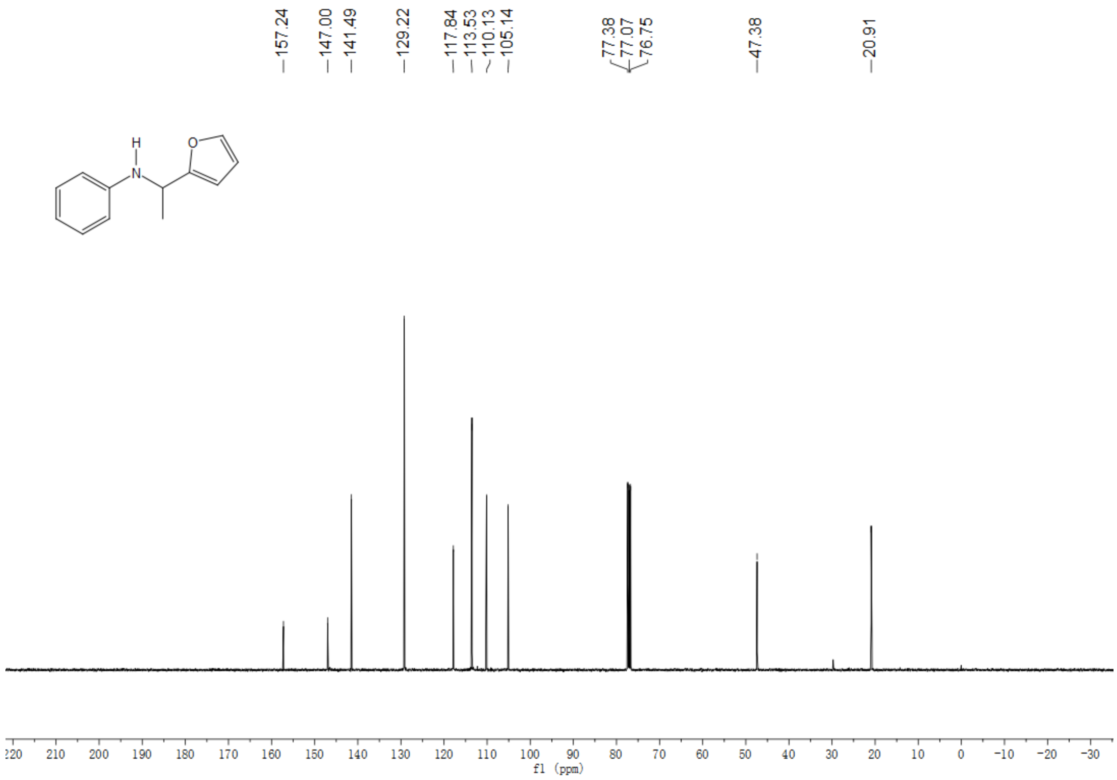

Supplementary Figure 100. ^{13}C NMR (101 MHz, room temperature, $CDCl_3$) spectra of product **42**

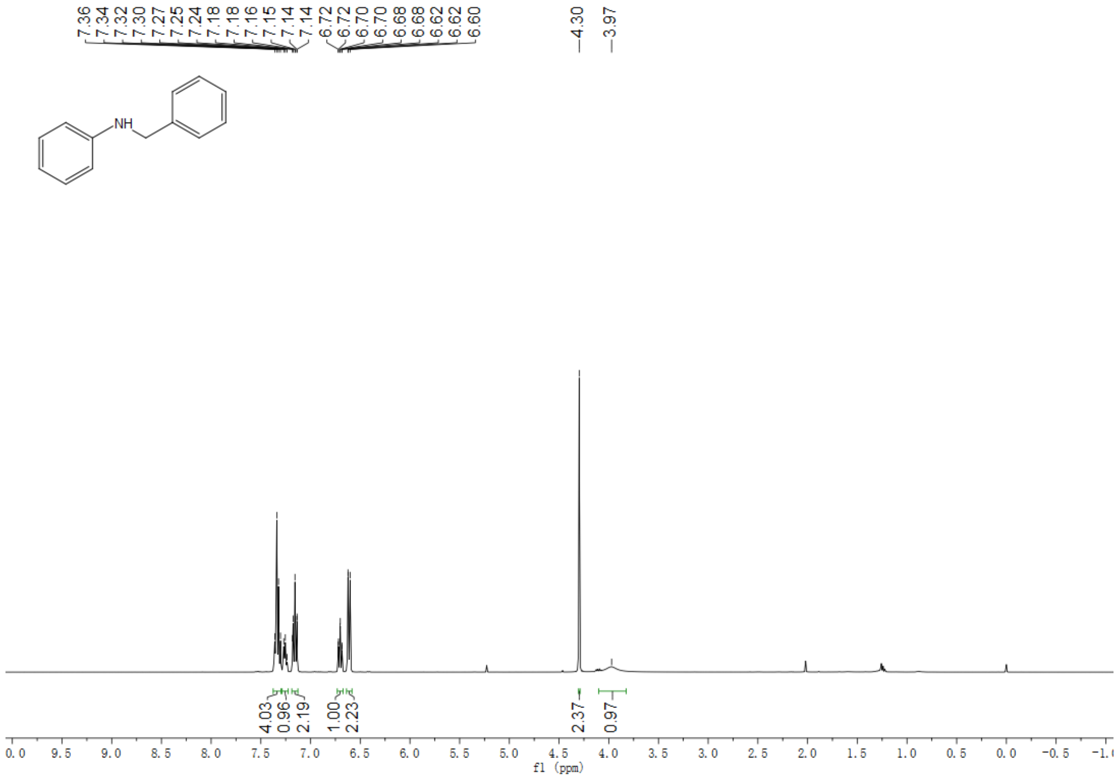

Supplementary Figure 101. ^{1}H NMR (400 MHz, room temperature, $CDCl_3$) spectra of product **43**

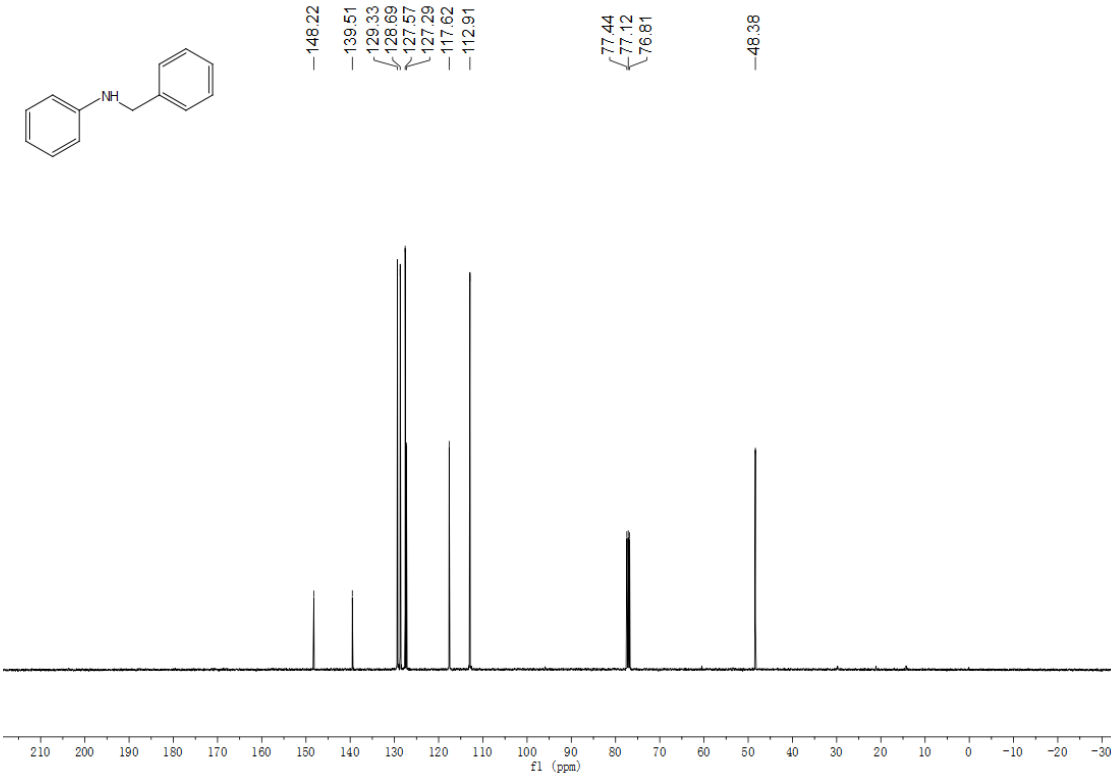

Supplementary Figure 102. ^{13}C NMR (101 MHz, room temperature, $CDCl_3$) spectra of product **43**

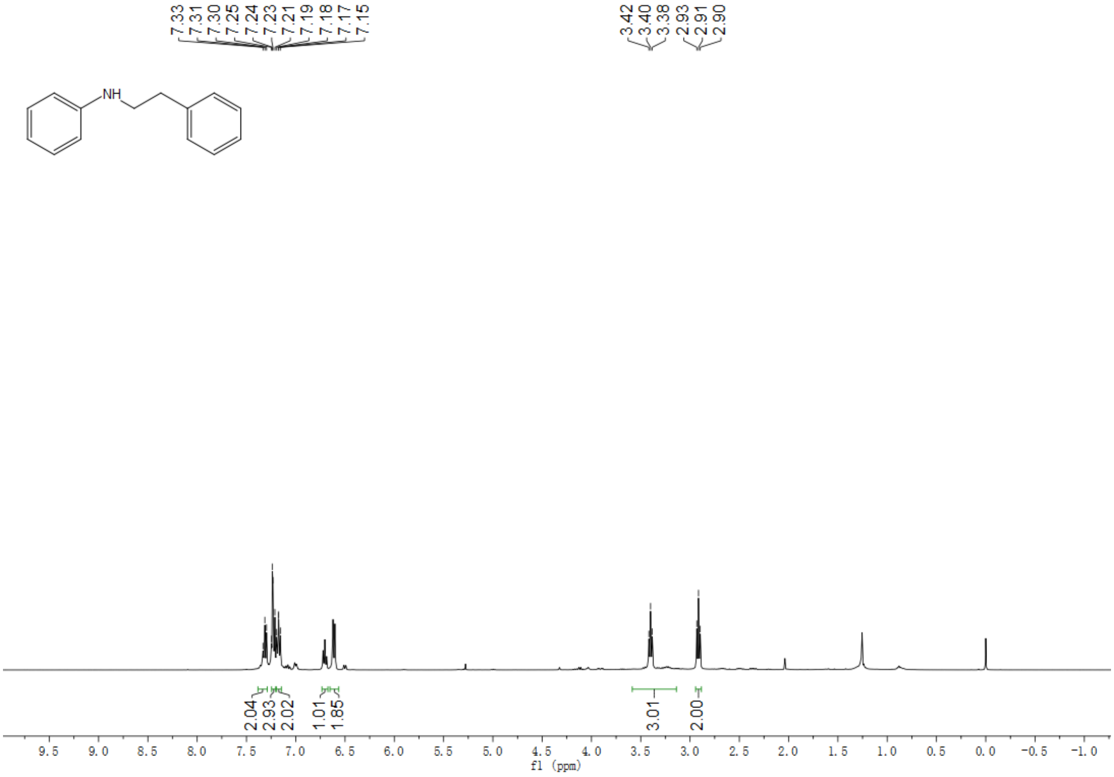

Supplementary Figure 103. ^{1}H NMR (400 MHz, room temperature, $CDCl_3$) spectra of product **44**

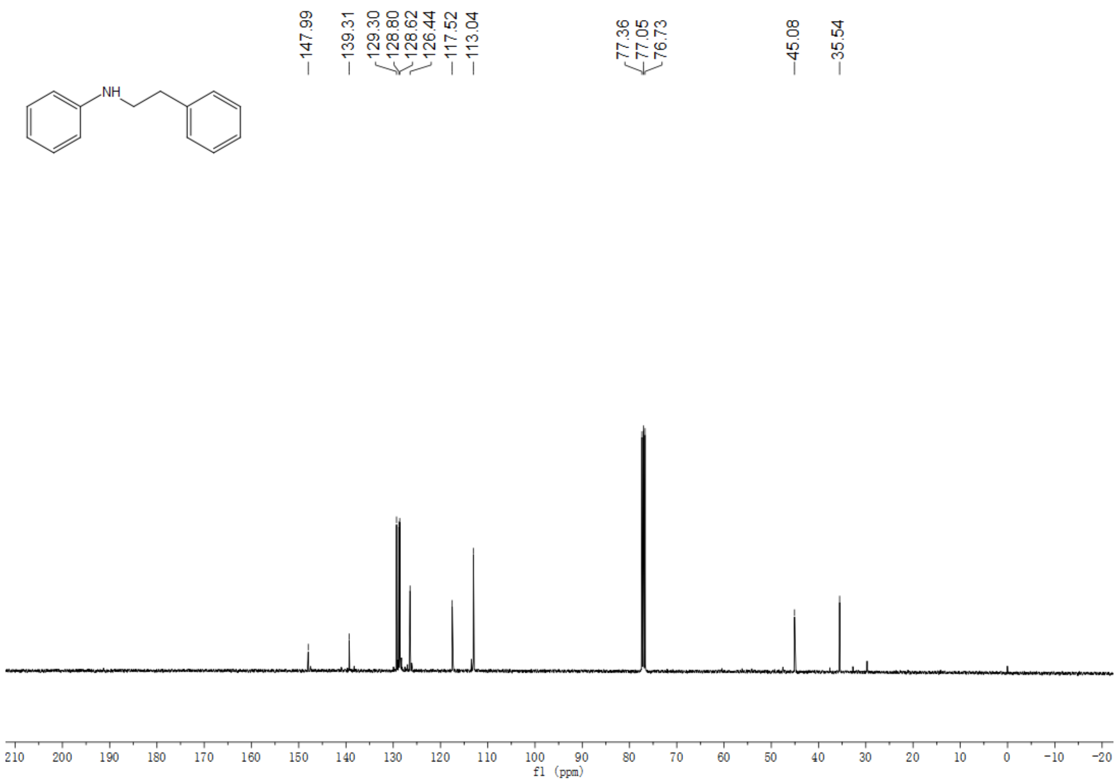

Supplementary Figure 104. ^{13}C NMR (101 MHz, room temperature, $CDCl_3$) spectra of product **44**

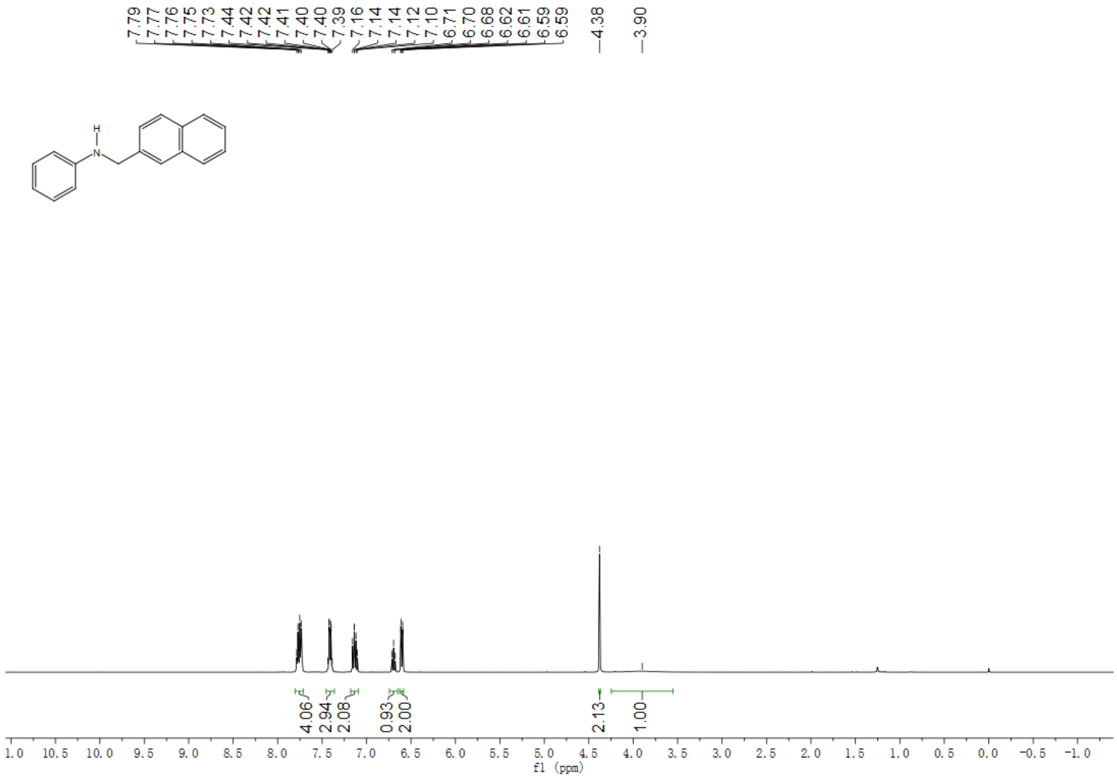

Supplementary Figure 105. ^{1}H NMR (400 MHz, room temperature, $CDCl_3$) spectra of product **45**

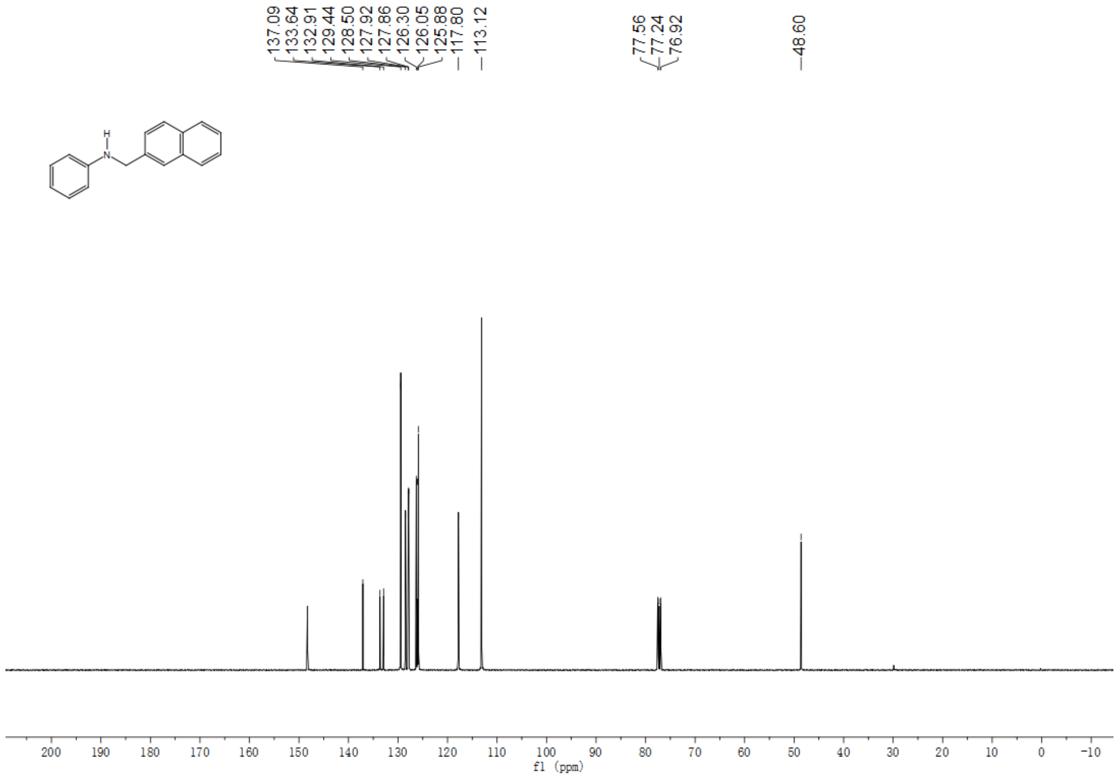

Supplementary Figure 106. ^{13}C NMR (101 MHz, room temperature, $CDCl_3$) spectra of product **45**

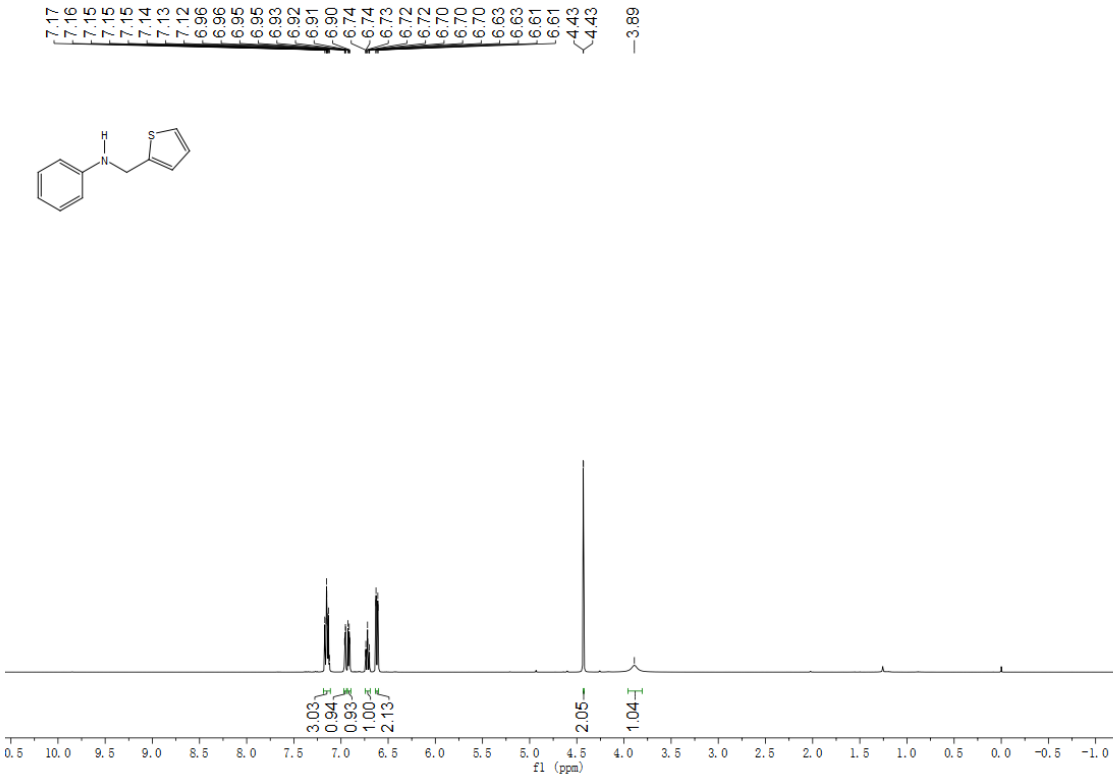

Supplementary Figure 107. ^{1}H NMR (400 MHz, room temperature, $CDCl_3$) spectra of product **46**

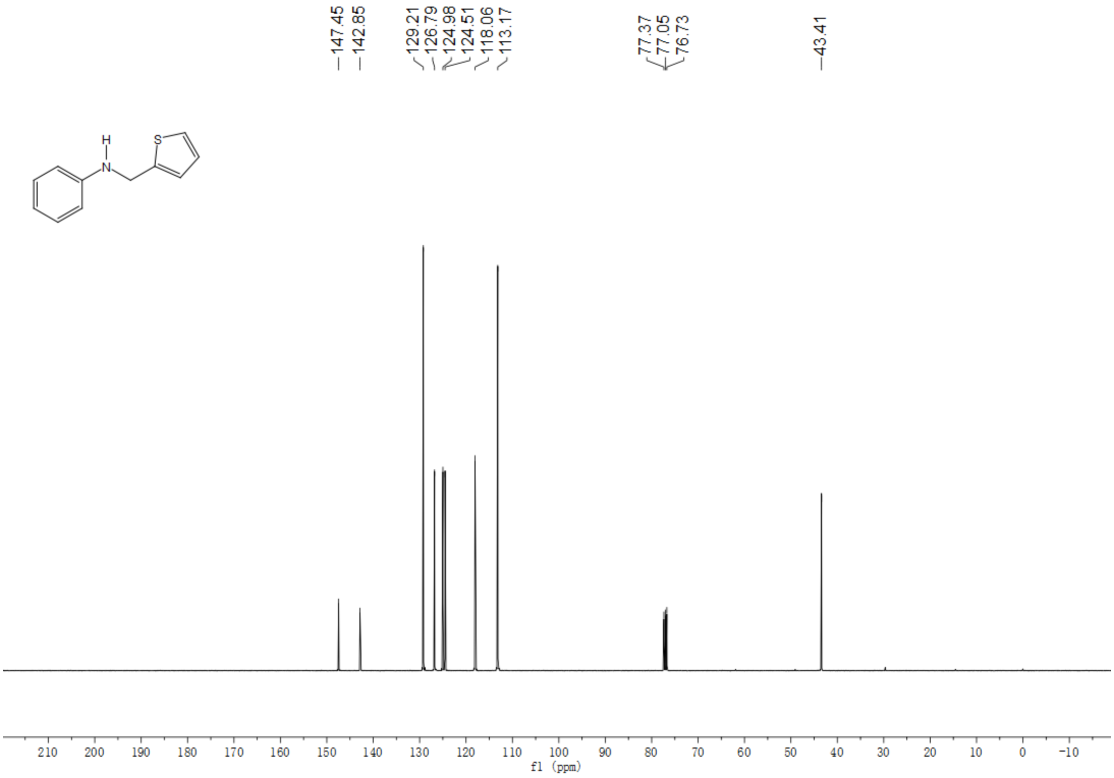

Supplementary Figure 108. ^{13}C NMR (101 MHz, room temperature, $CDCl_3$) spectra of product **46**

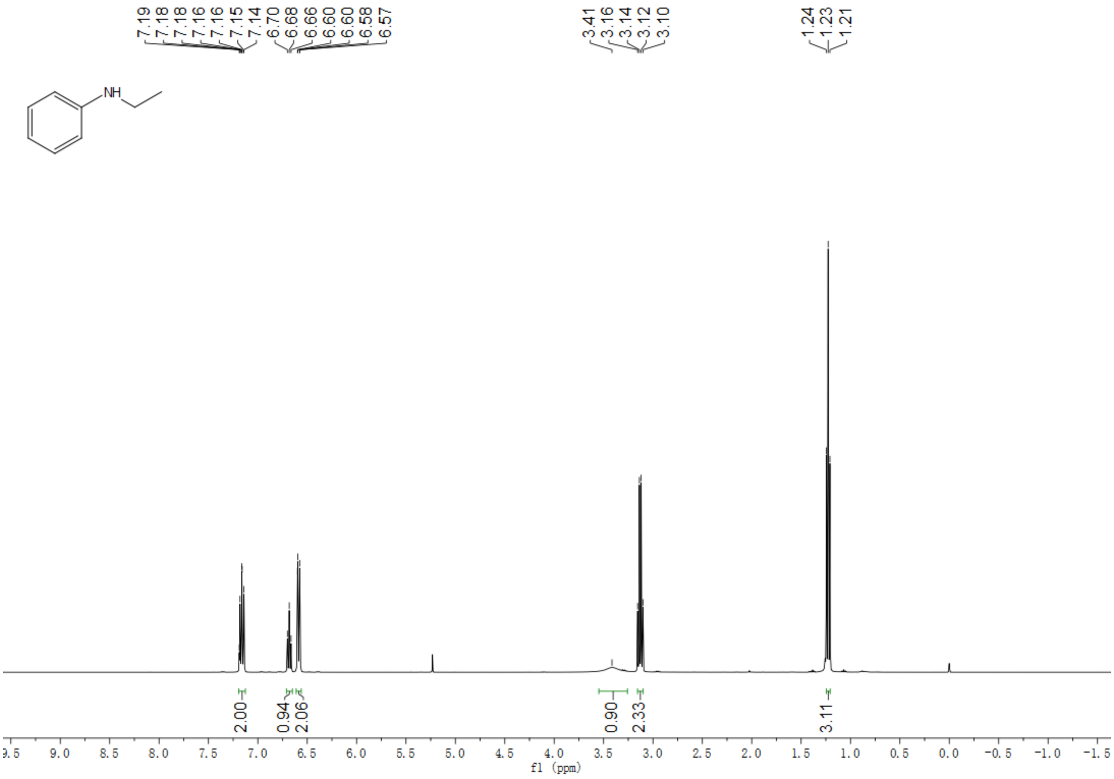

Supplementary Figure 109. ^{1}H NMR (400 MHz, room temperature, $CDCl_3$) spectra of product **47**

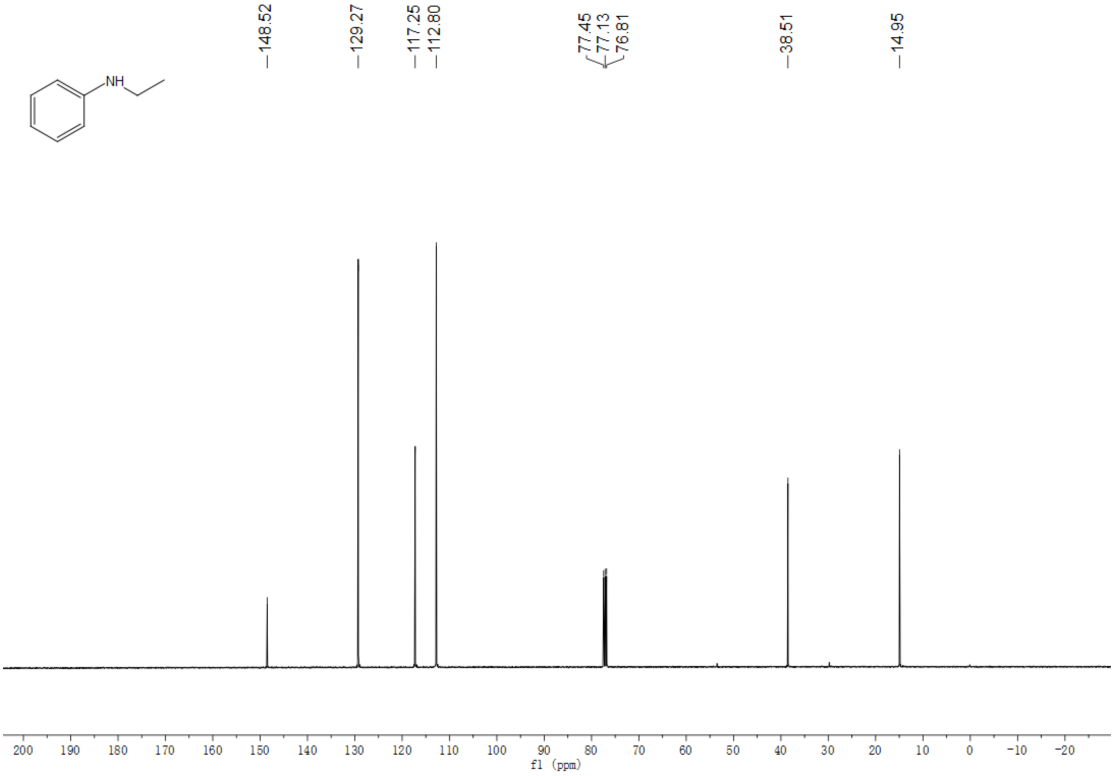

Supplementary Figure 110. ^{13}C NMR (101 MHz, room temperature, $CDCl_3$) spectra of product **47**

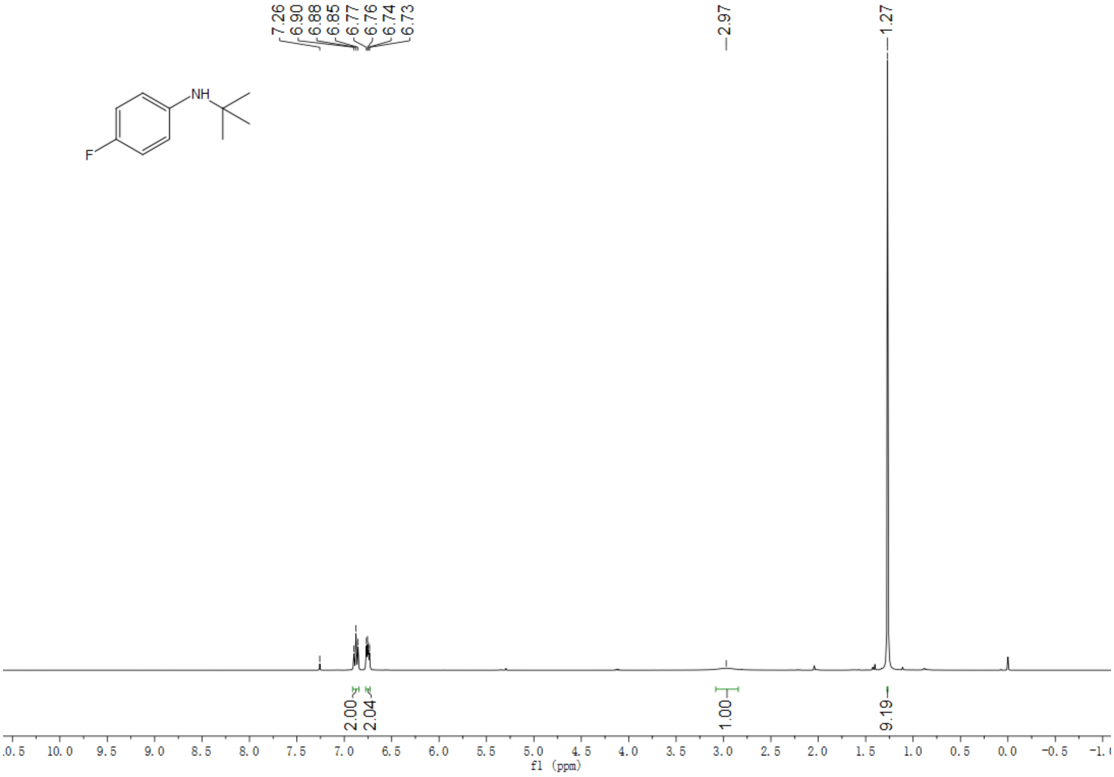

Supplementary Figure 111. ^{1}H NMR (400 MHz, room temperature, $CDCl_3$) spectra of product **48**

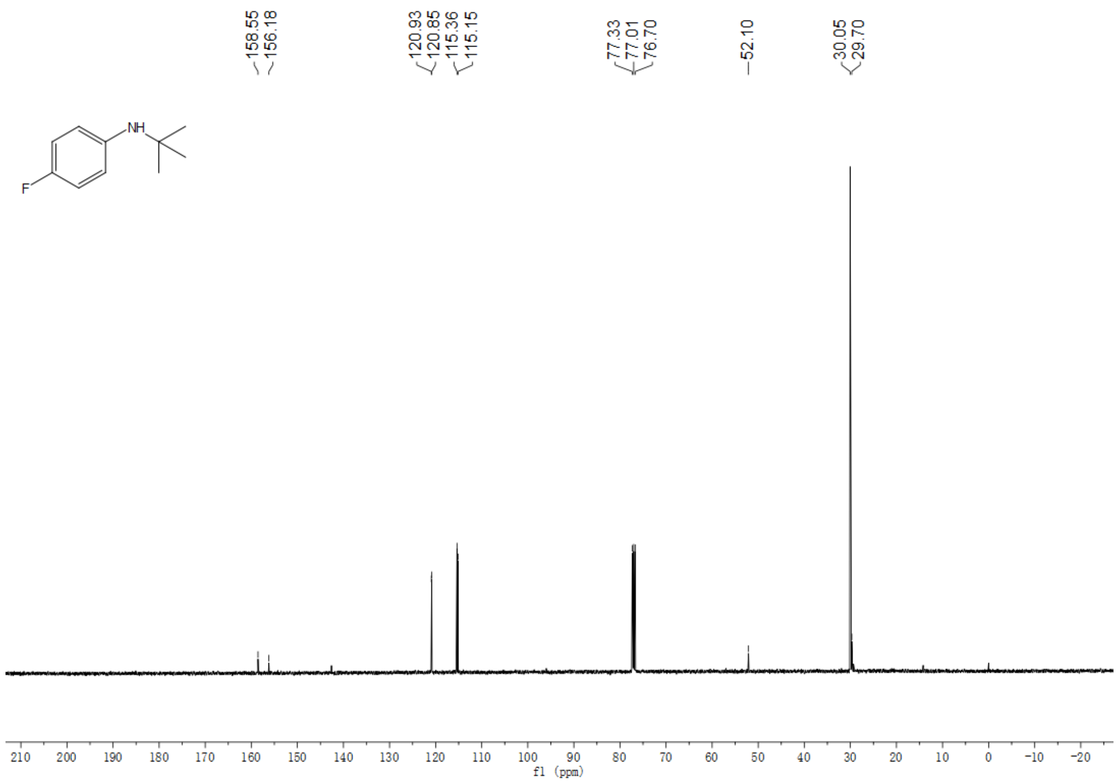

Supplementary Figure 112. ^{13}C NMR (101 MHz, room temperature, $CDCl_3$) spectra of product **48**

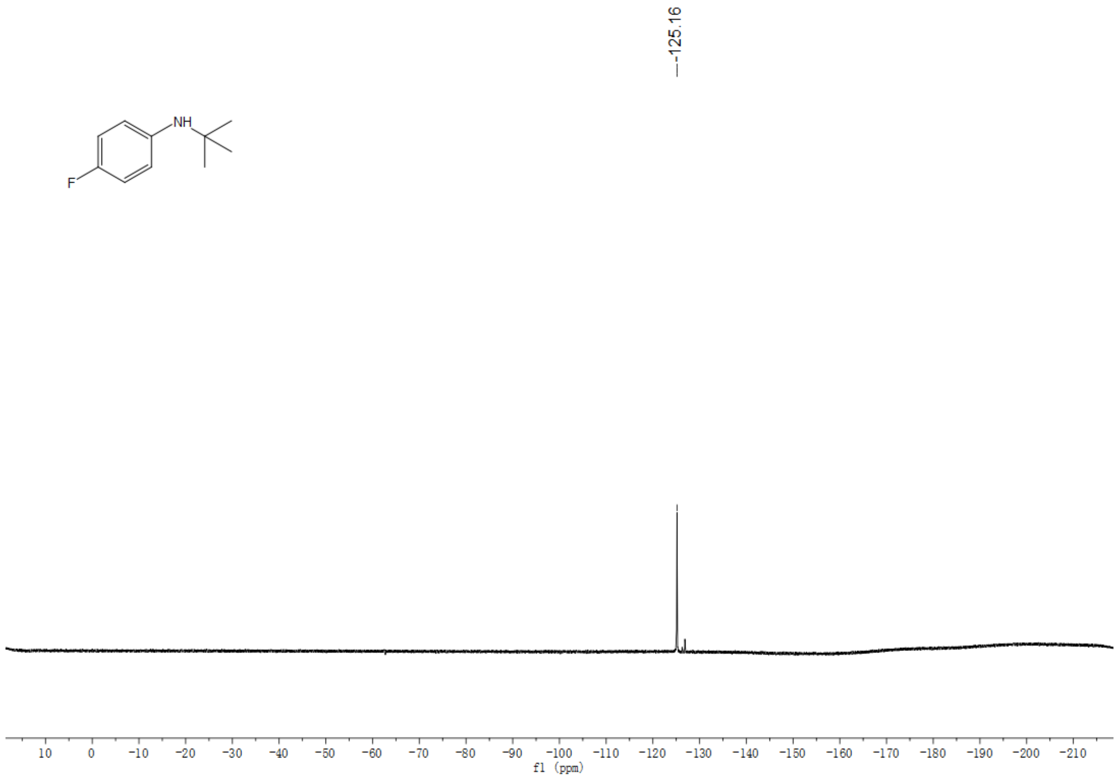

Supplementary Figure 113. ^{19}F NMR (376 MHz, room temperature, $CDCl_3$) spectra of product **48**

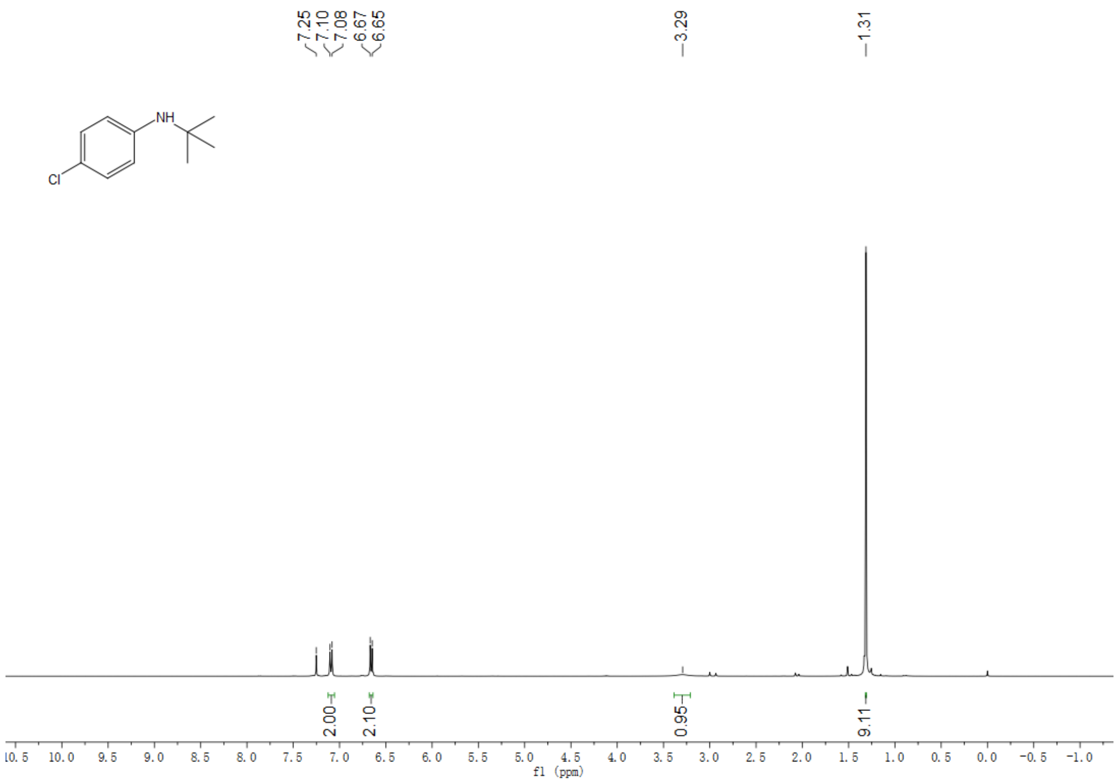

Supplementary Figure 114. ^{1}H NMR (400 MHz, room temperature, $CDCl_3$) spectra of product **49**

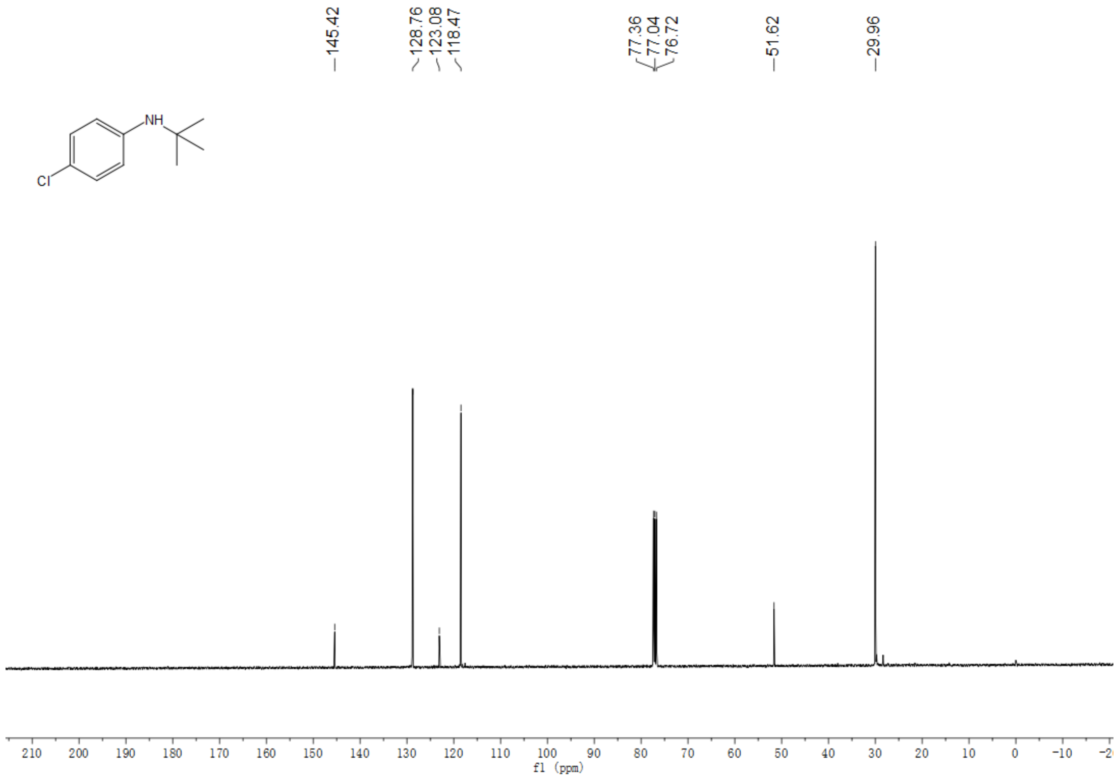

Supplementary Figure 115. ^{13}C NMR (101 MHz, room temperature, $CDCl_3$) spectra of product **49**

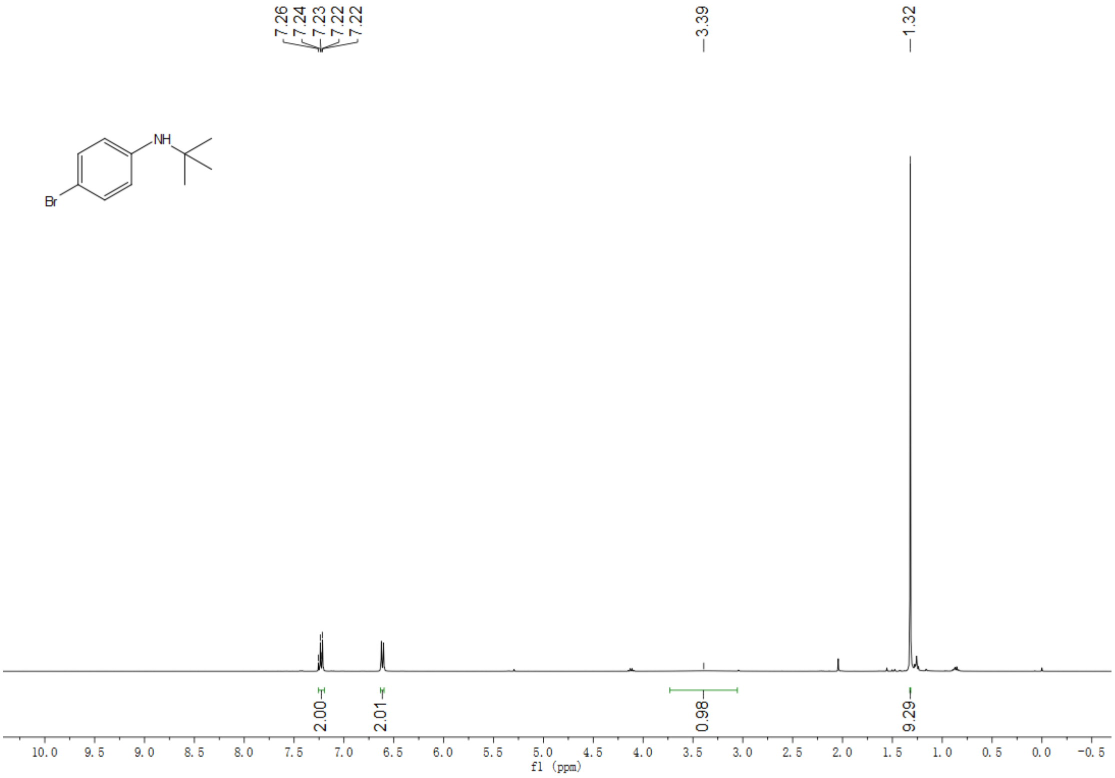

Supplementary Figure 116. ^{1}H NMR (400 MHz, room temperature, $CDCl_3$) spectra of product **50**

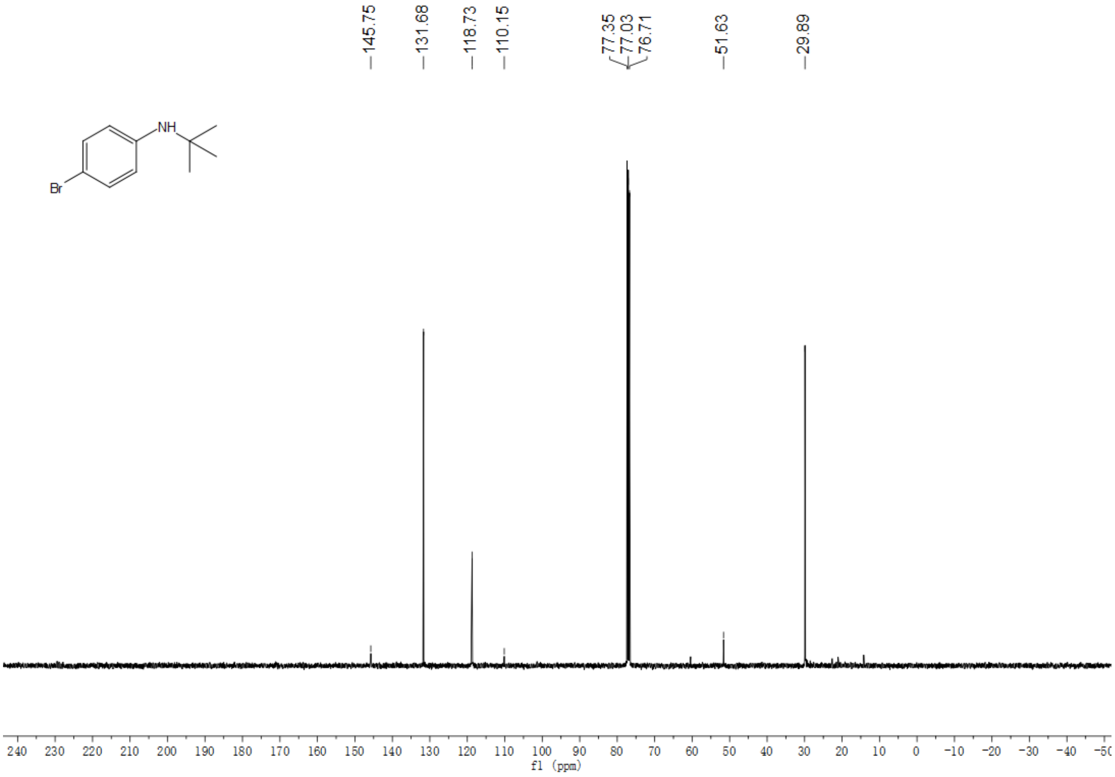

Supplementary Figure 117. ^{13}C NMR (101 MHz, room temperature, $CDCl_3$) spectra of product **50**

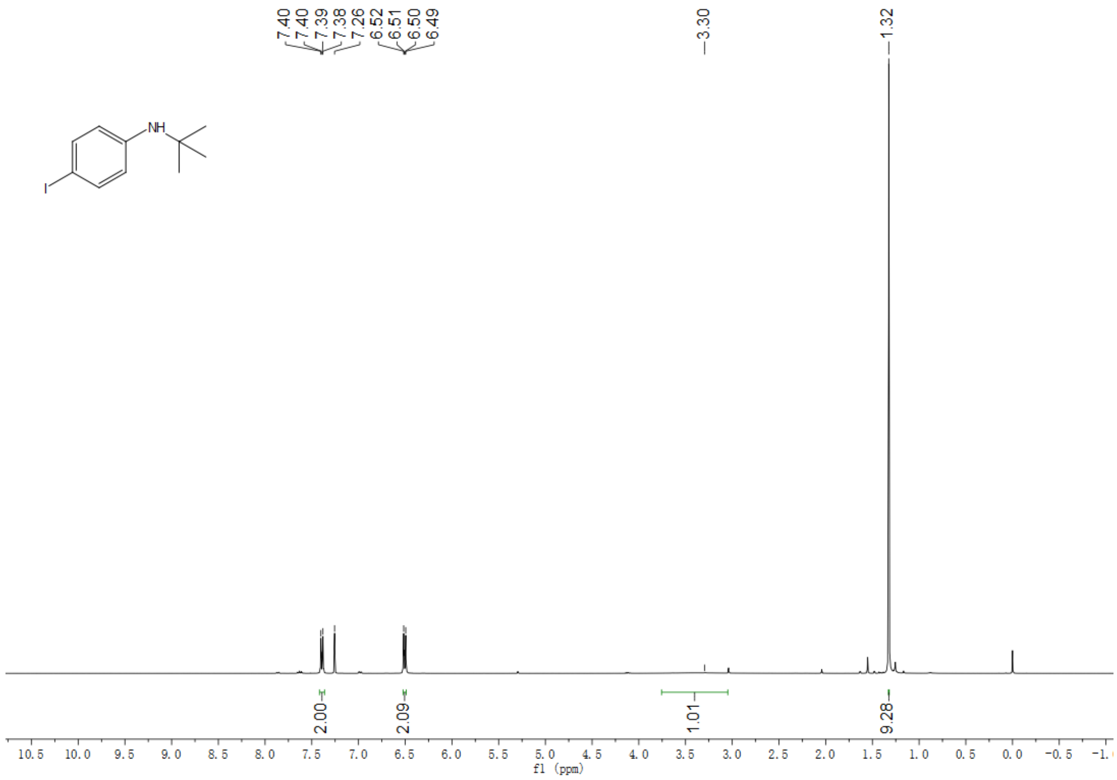

Supplementary Figure 118. ^{1}H NMR (400 MHz, room temperature, $CDCl_3$) spectra of product **51**

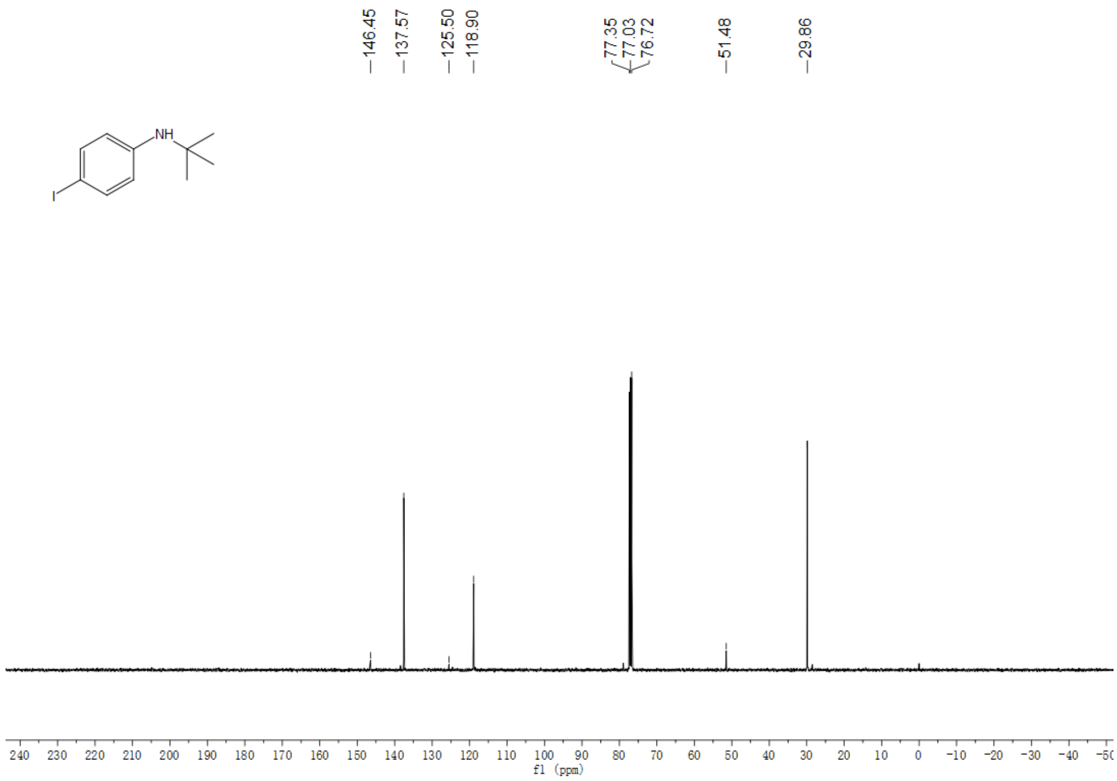

Supplementary Figure 119. ^{13}C NMR (101 MHz, room temperature, $CDCl_3$) spectra of product **51**

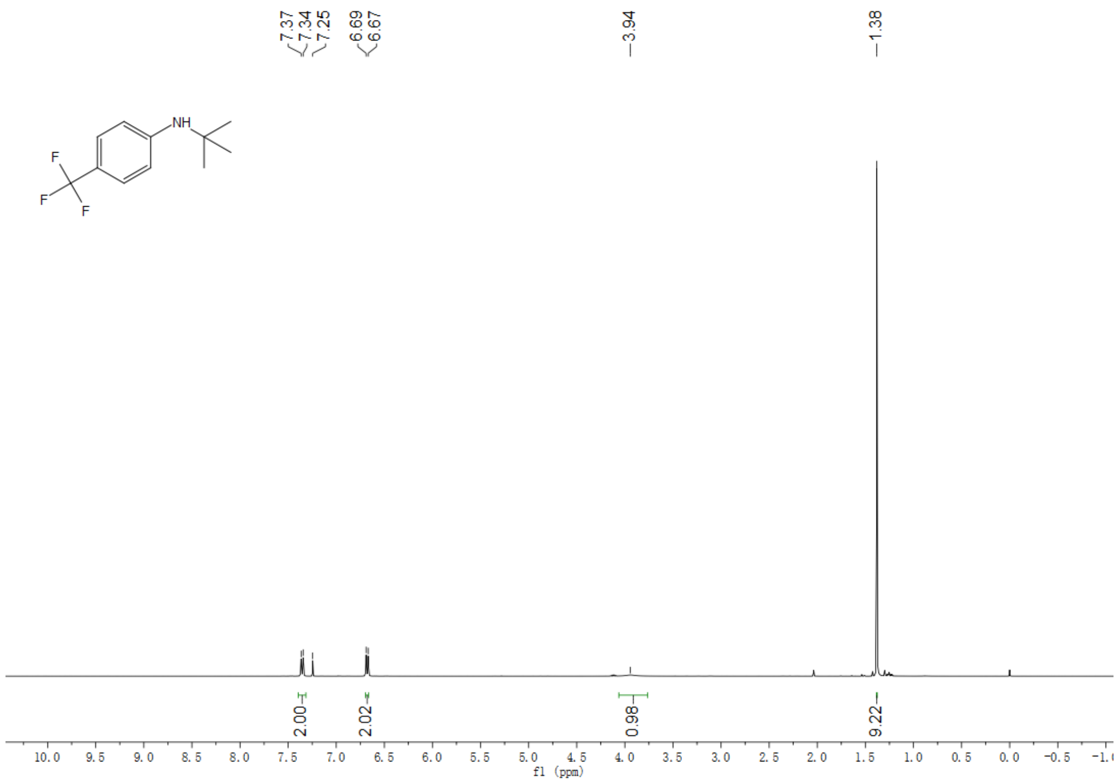

Supplementary Figure 120. ^{1}H NMR (400 MHz, room temperature, $CDCl_3$) spectra of product **52**

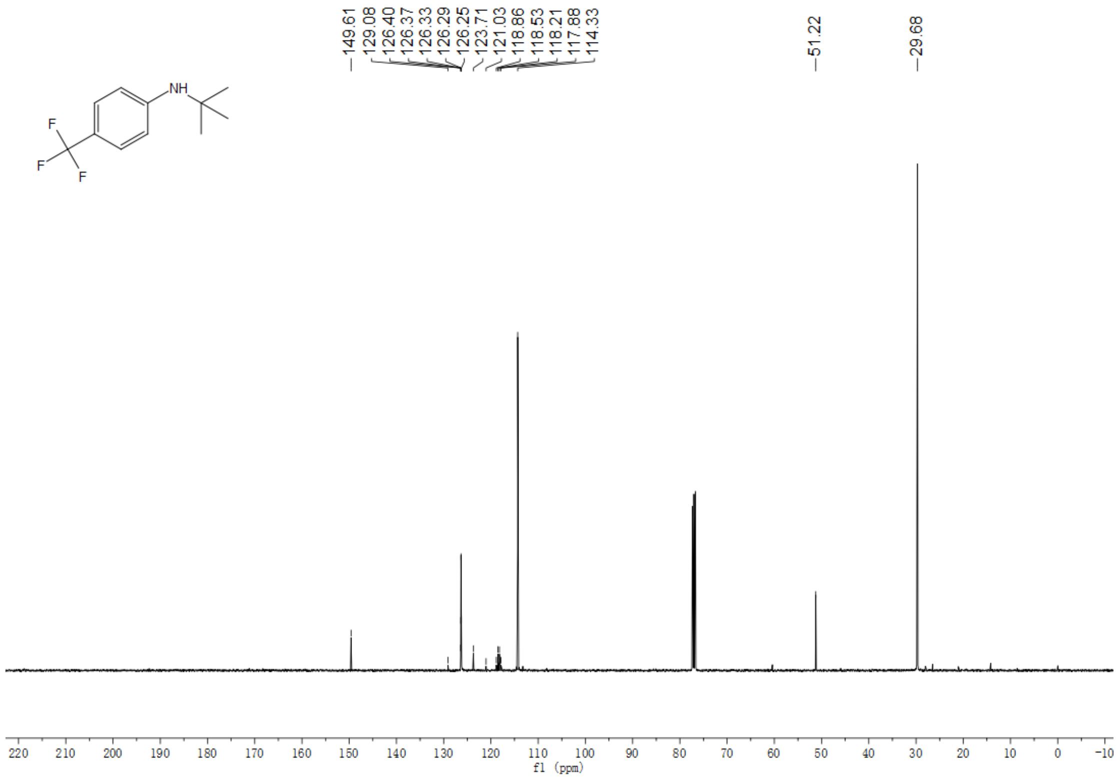

Supplementary Figure 121. ^{13}C NMR (101 MHz, room temperature, $CDCl_3$) spectra of product **52**

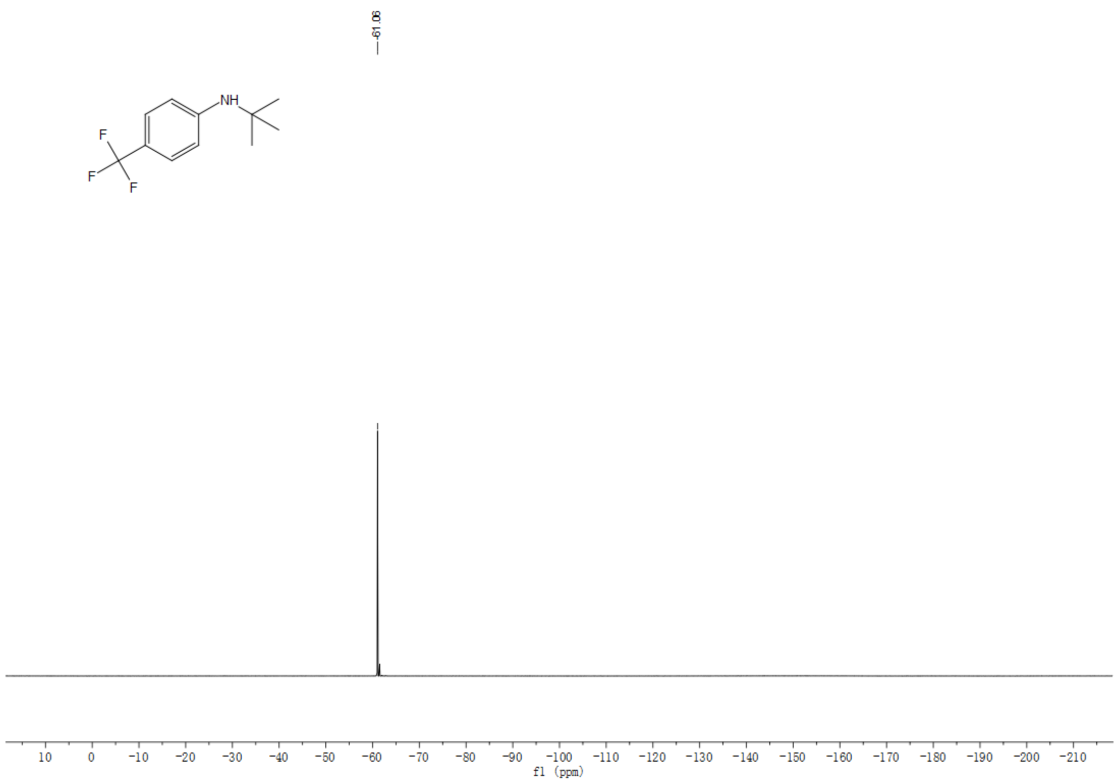

Supplementary Figure 122. ^{19}F NMR (376 MHz, room temperature, $CDCl_3$) spectra of product **52**

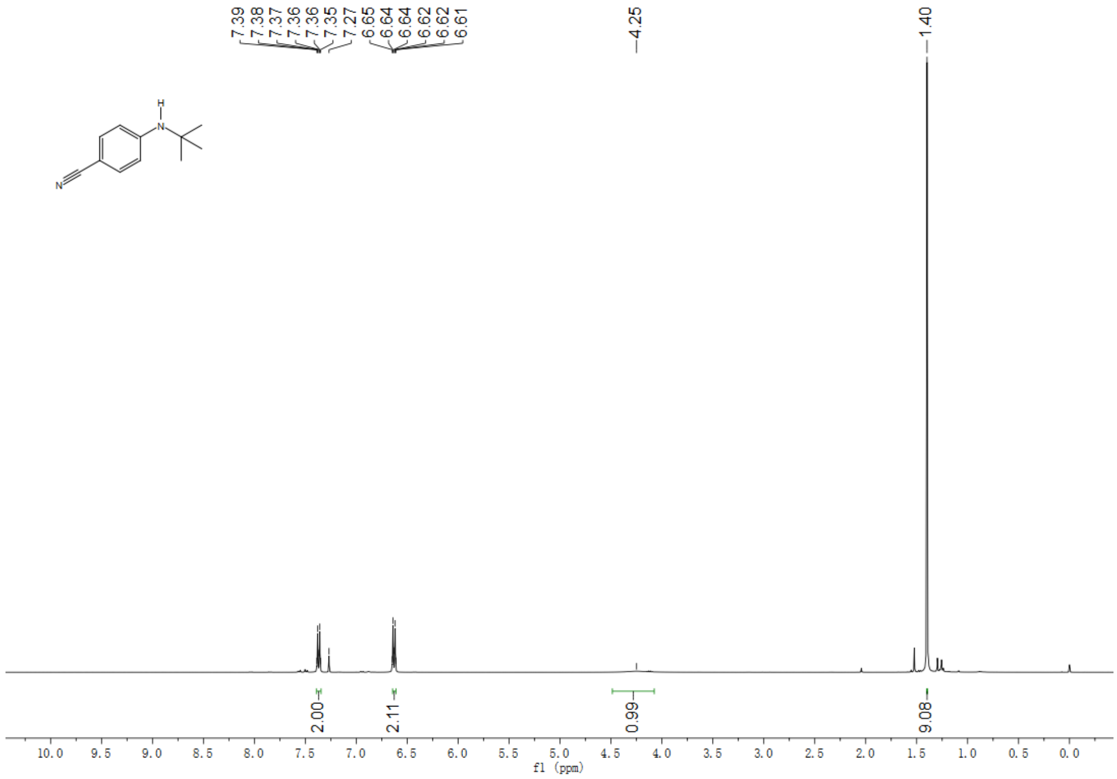

Supplementary Figure 123. ^{1}H NMR (400 MHz, room temperature, $CDCl_3$) spectra of product **53**

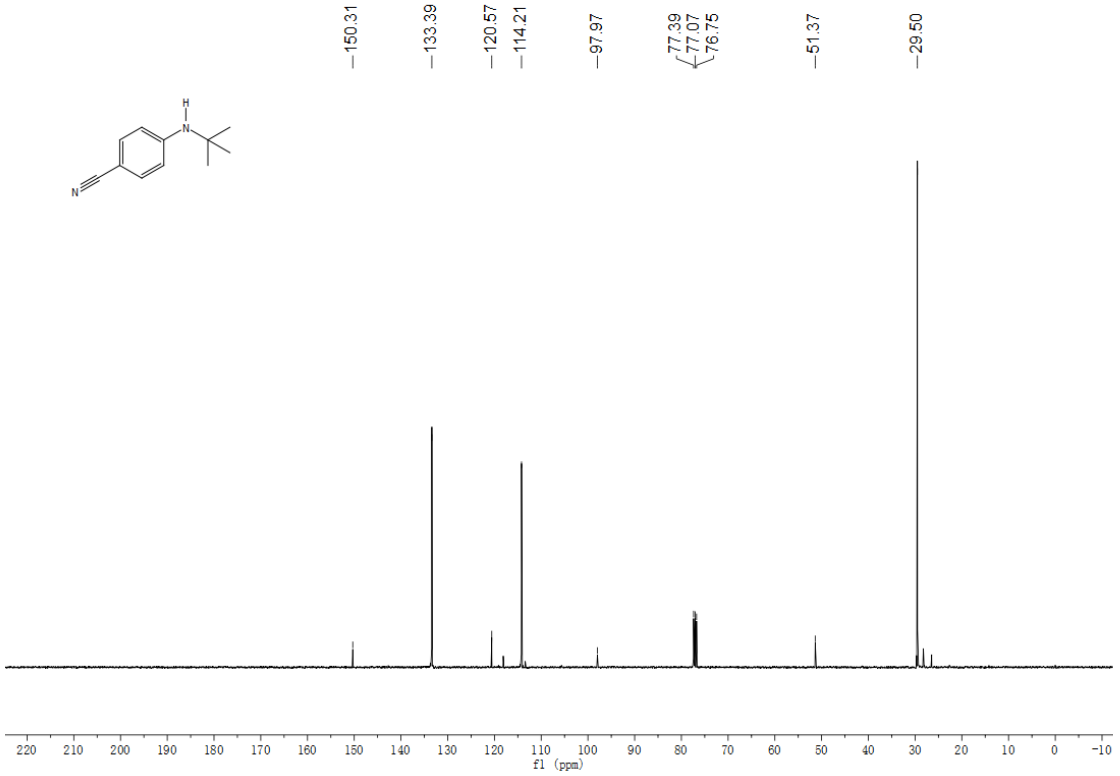

Supplementary Figure 124. ^{13}C NMR (101 MHz, room temperature, $CDCl_3$) spectra of product **53**

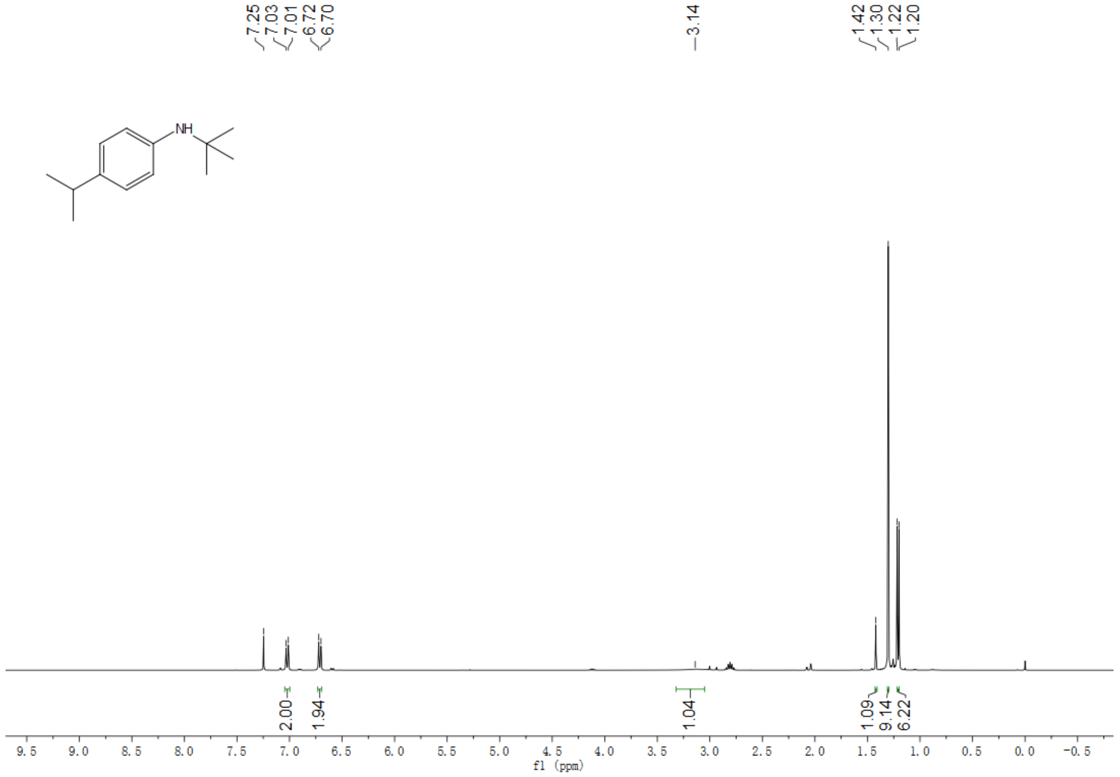

Supplementary Figure 125. ^{1}H NMR (400 MHz, room temperature, $CDCl_3$) spectra of product **54**

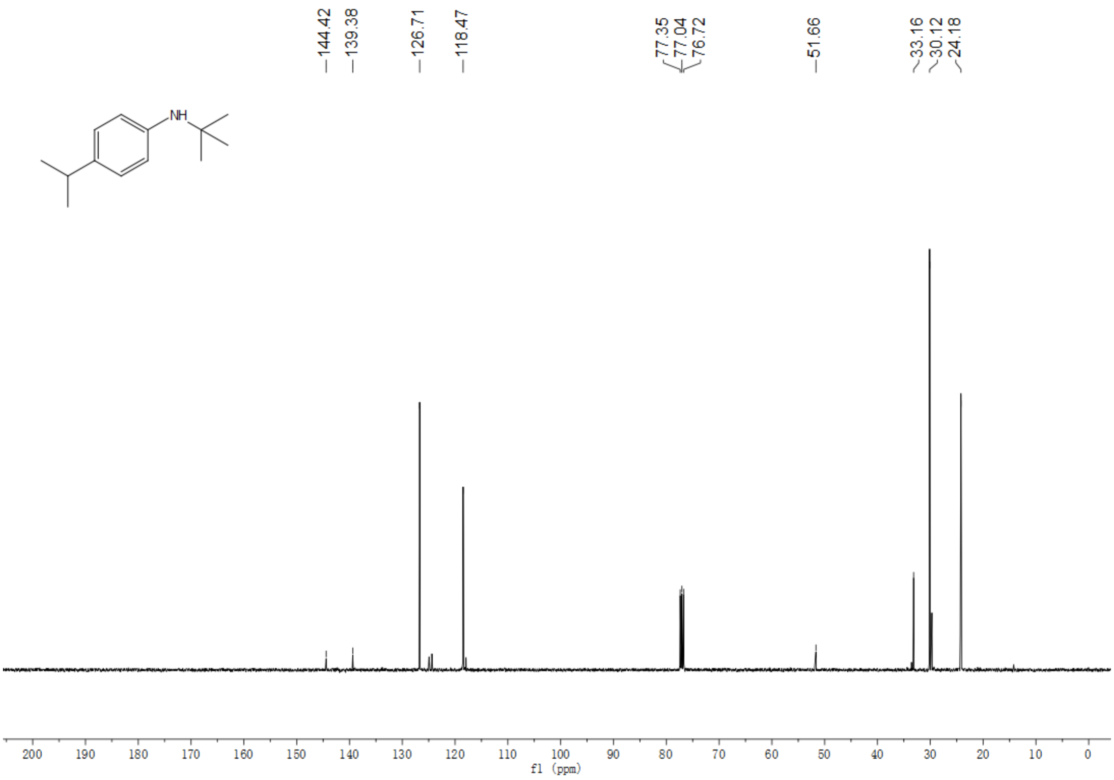

Supplementary Figure 126. ^{13}C NMR (101 MHz, room temperature, $CDCl_3$) spectra of product **54**

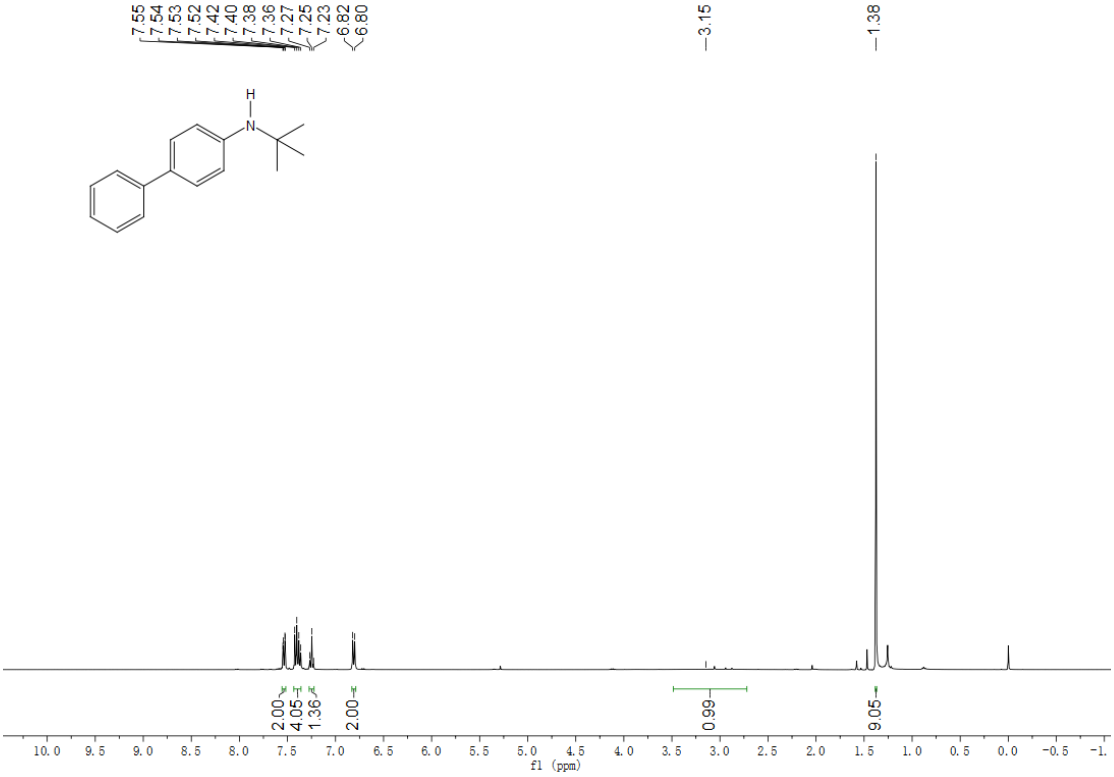

Supplementary Figure 127. ^{1}H NMR (400 MHz, room temperature, $CDCl_3$) spectra of product **55**

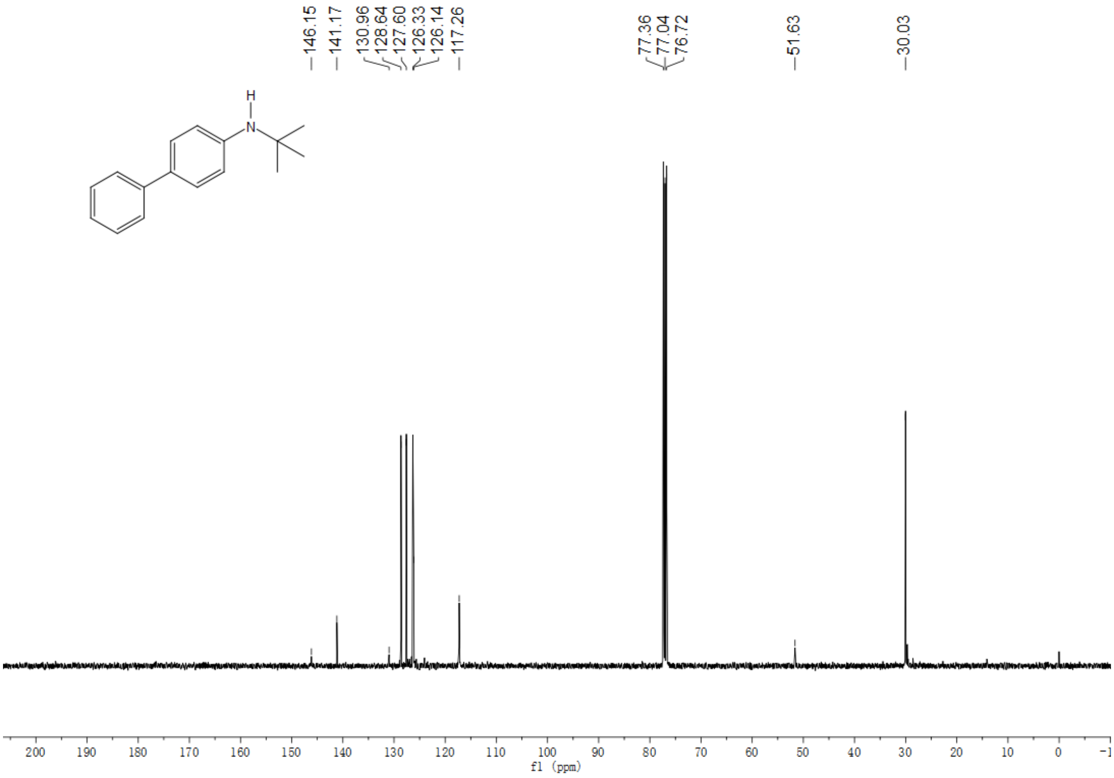

Supplementary Figure 128. ^{13}C NMR (101 MHz, room temperature, $CDCl_3$) spectra of product **55**

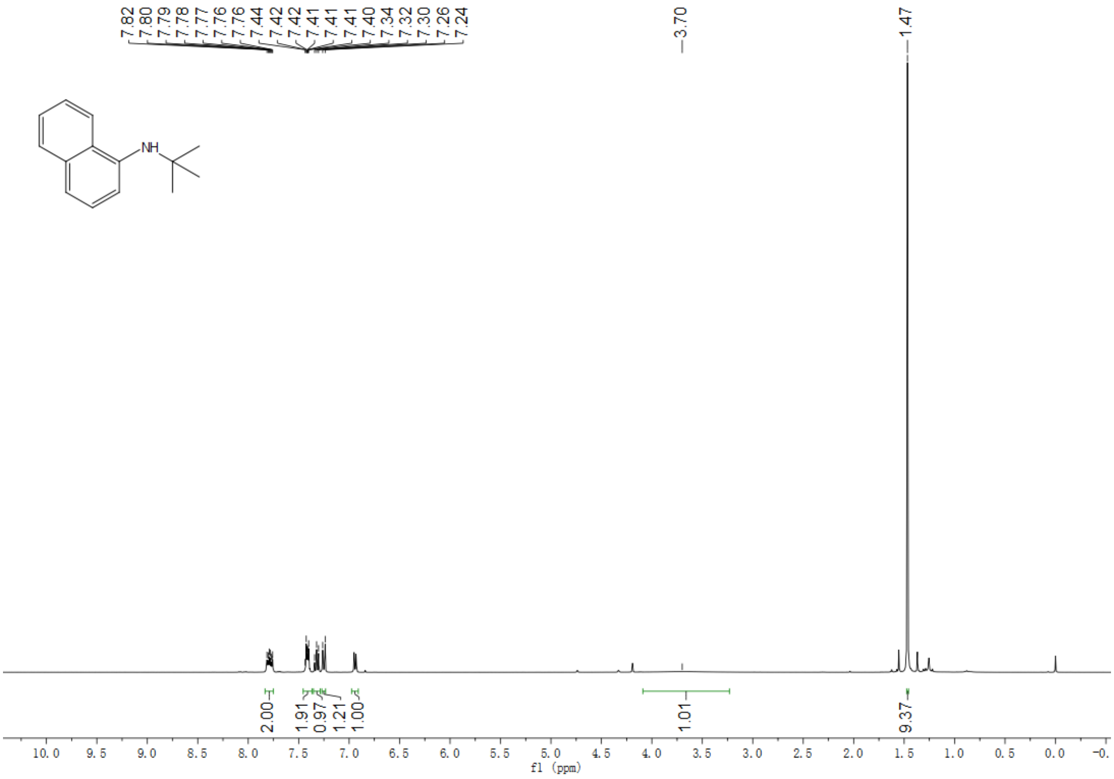

Supplementary Figure 129. ^{1}H NMR (400 MHz, room temperature, $CDCl_3$) spectra of product **56**

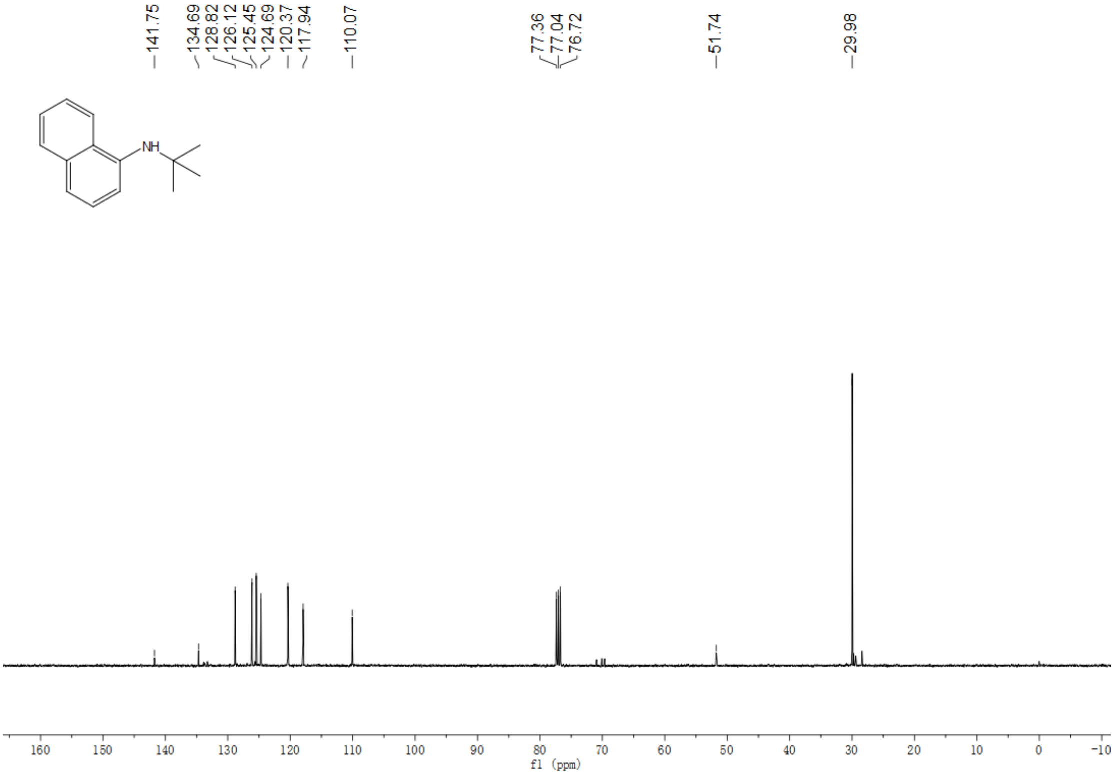

Supplementary Figure 130. ^{13}C NMR (101 MHz, room temperature, $CDCl_3$) spectra of product **56**

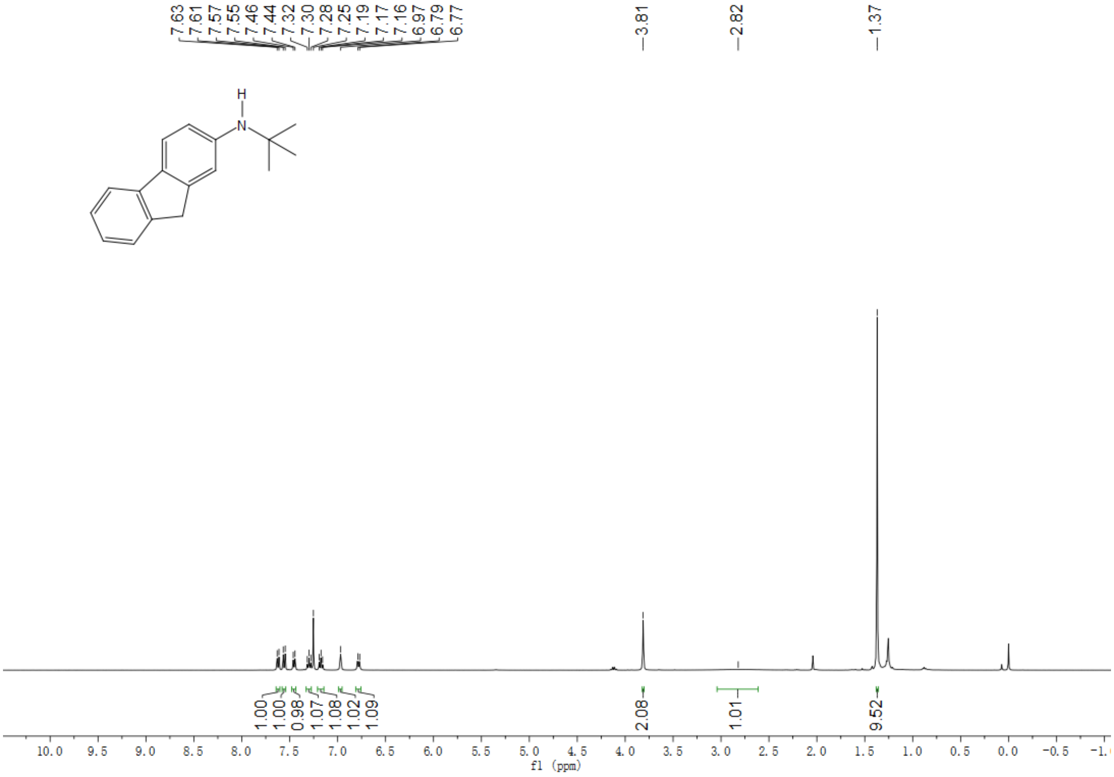

Supplementary Figure 131. ^{1}H NMR (400 MHz, room temperature, $CDCl_3$) spectra of product **57**

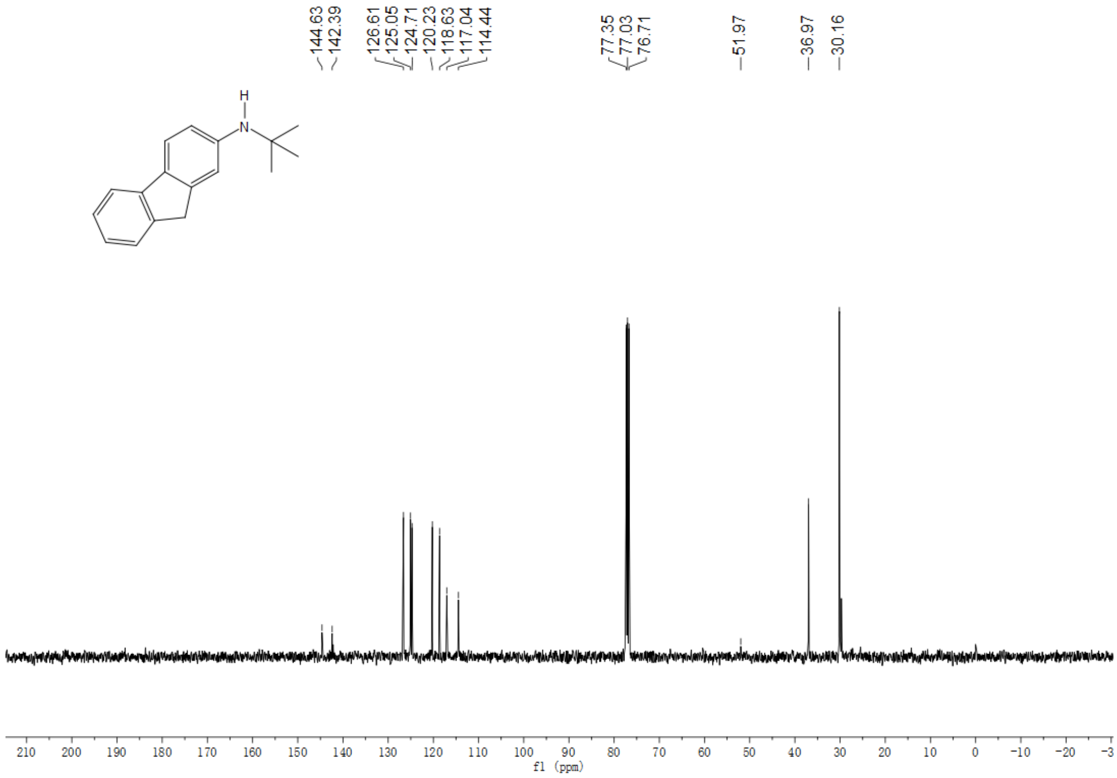

Supplementary Figure 132. ^{13}C NMR (101 MHz, room temperature, $CDCl_3$) spectra of product **57**

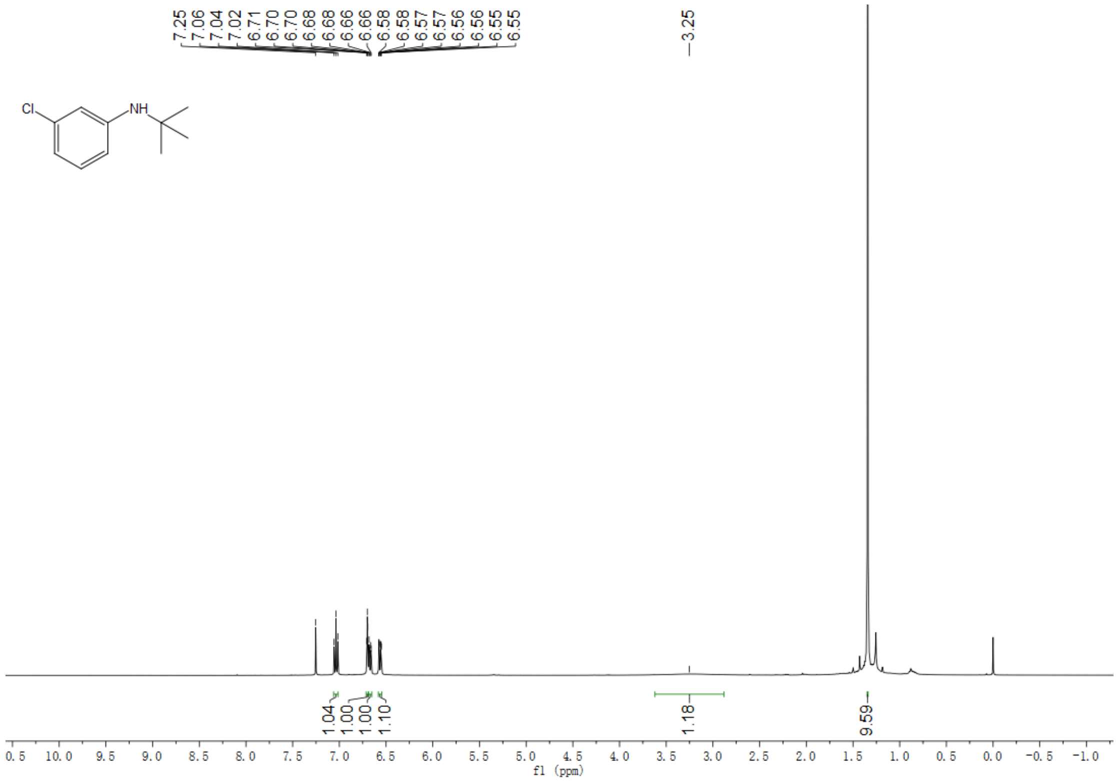

Supplementary Figure 133. ^{1}H NMR (400 MHz, room temperature, $CDCl_3$) spectra of product **58**

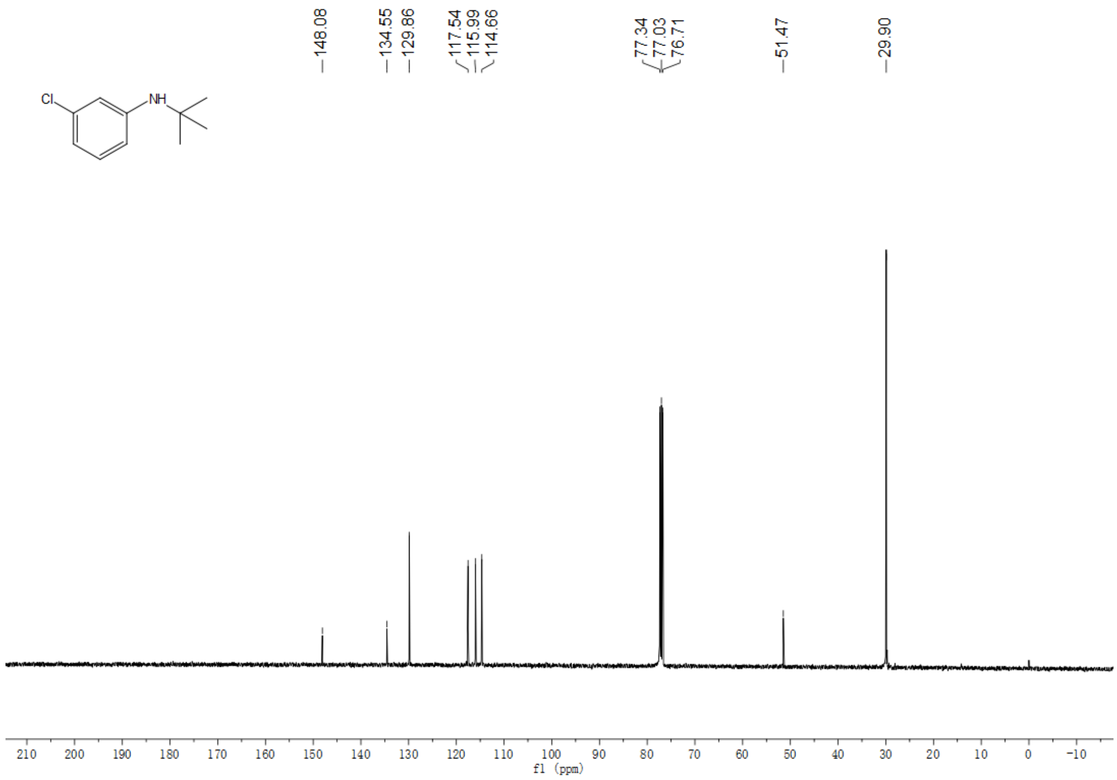

Supplementary Figure 134. ^{13}C NMR (101 MHz, room temperature, $CDCl_3$) spectra of product **58**

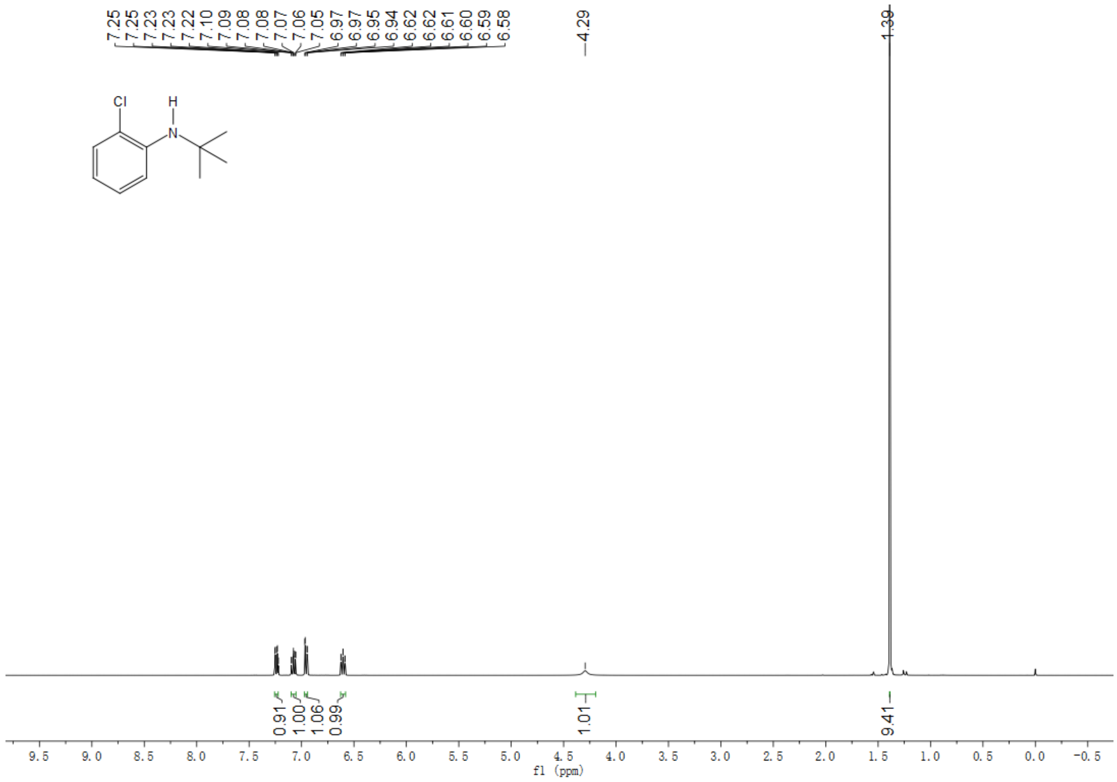

Supplementary Figure 135. ^{1}H NMR (400 MHz, room temperature, $CDCl_3$) spectra of product 5**9**

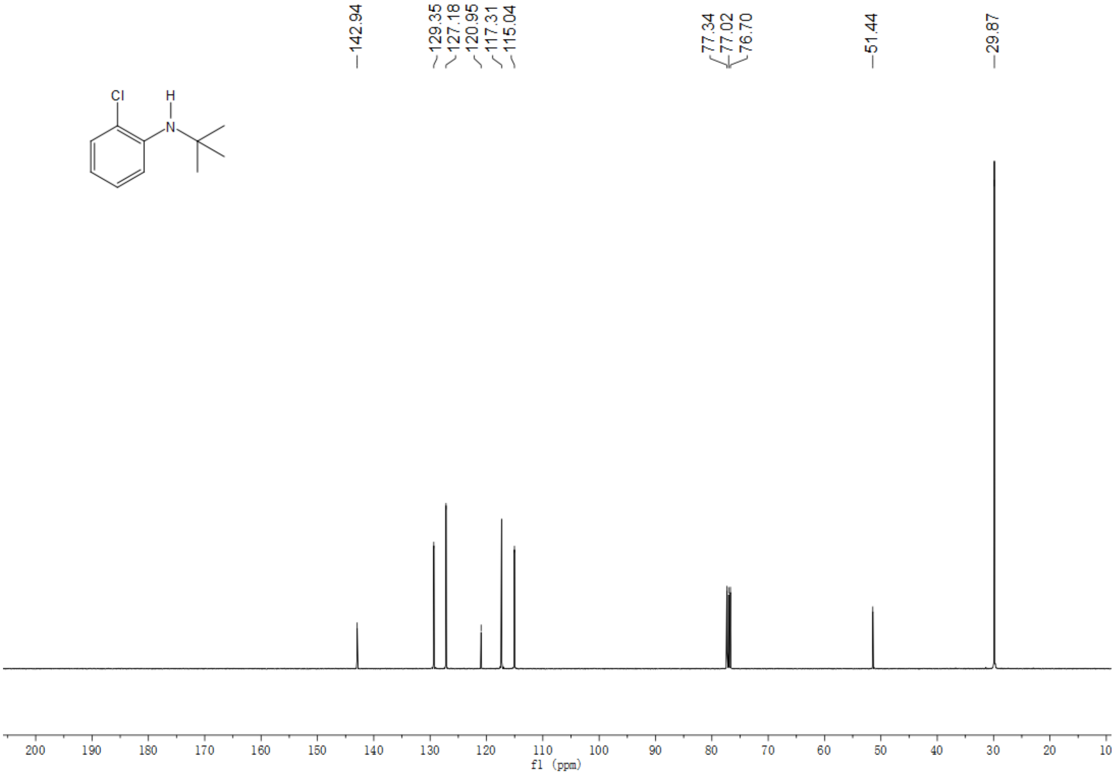

Supplementary Figure 136. ^{13}C NMR (101 MHz, room temperature, $CDCl_3$) spectra of product **59**

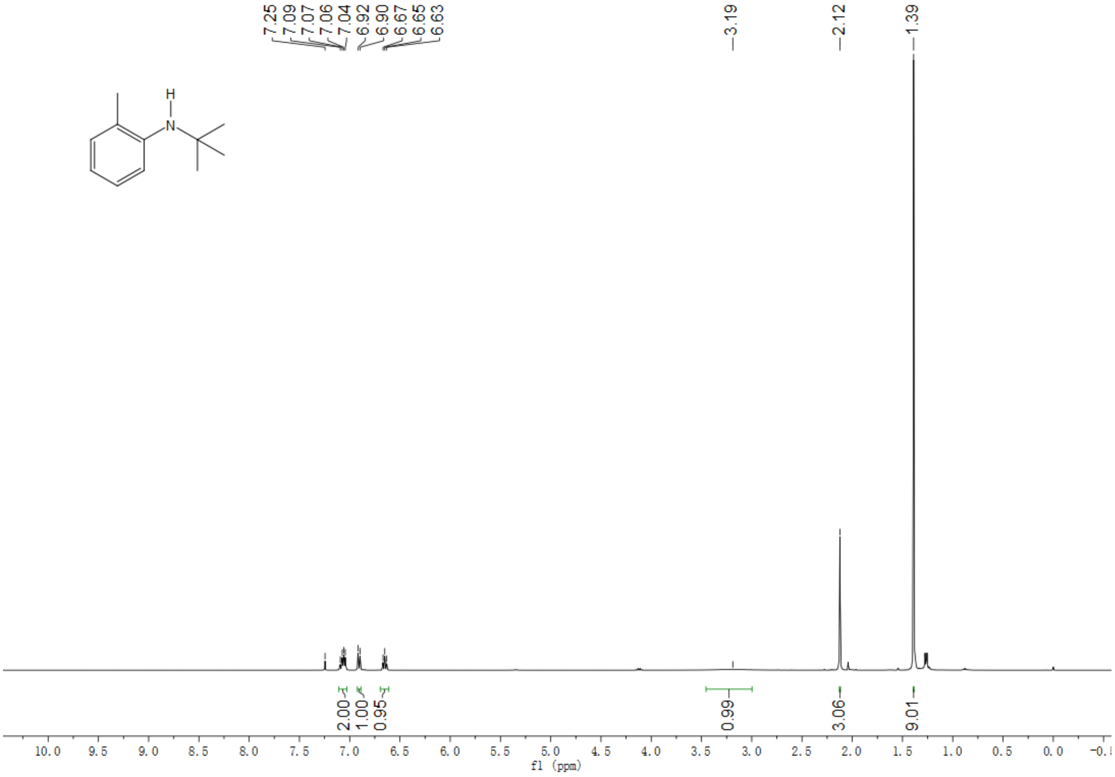

Supplementary Figure 137. ^{1}H NMR (400 MHz, room temperature, $CDCl_3$) spectra of product **60**

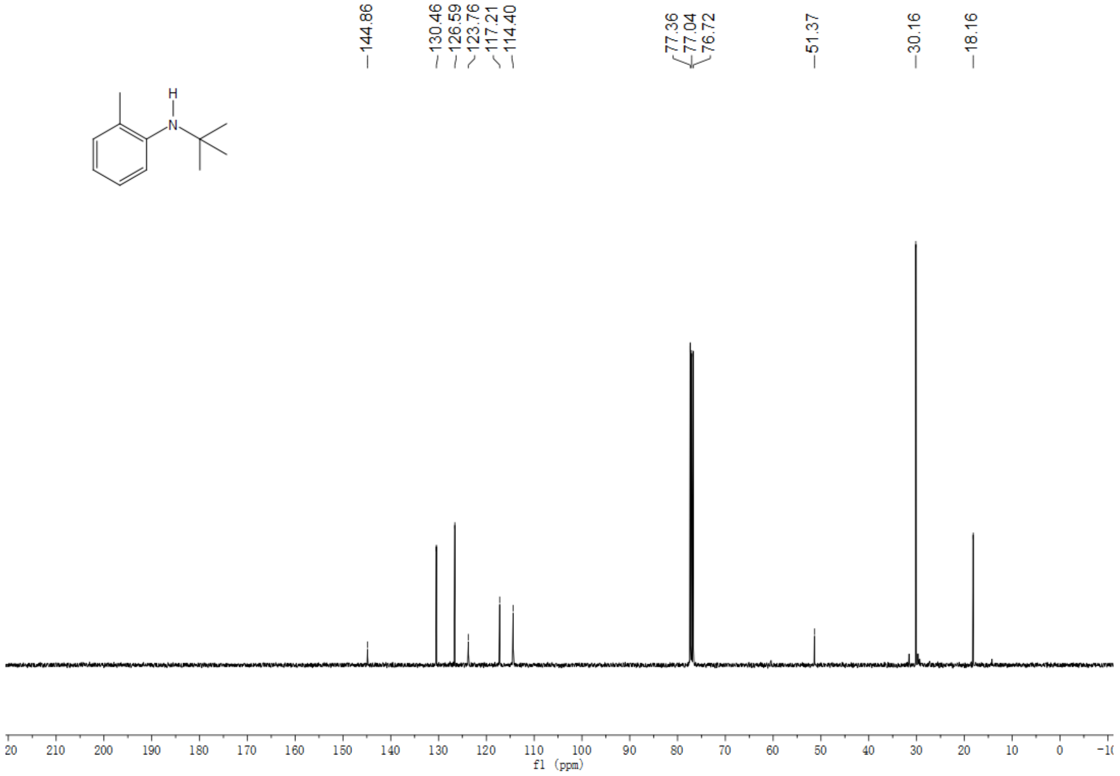

Supplementary Figure 138. ^{13}C NMR (101 MHz, room temperature, $CDCl_3$) spectra of product **60**

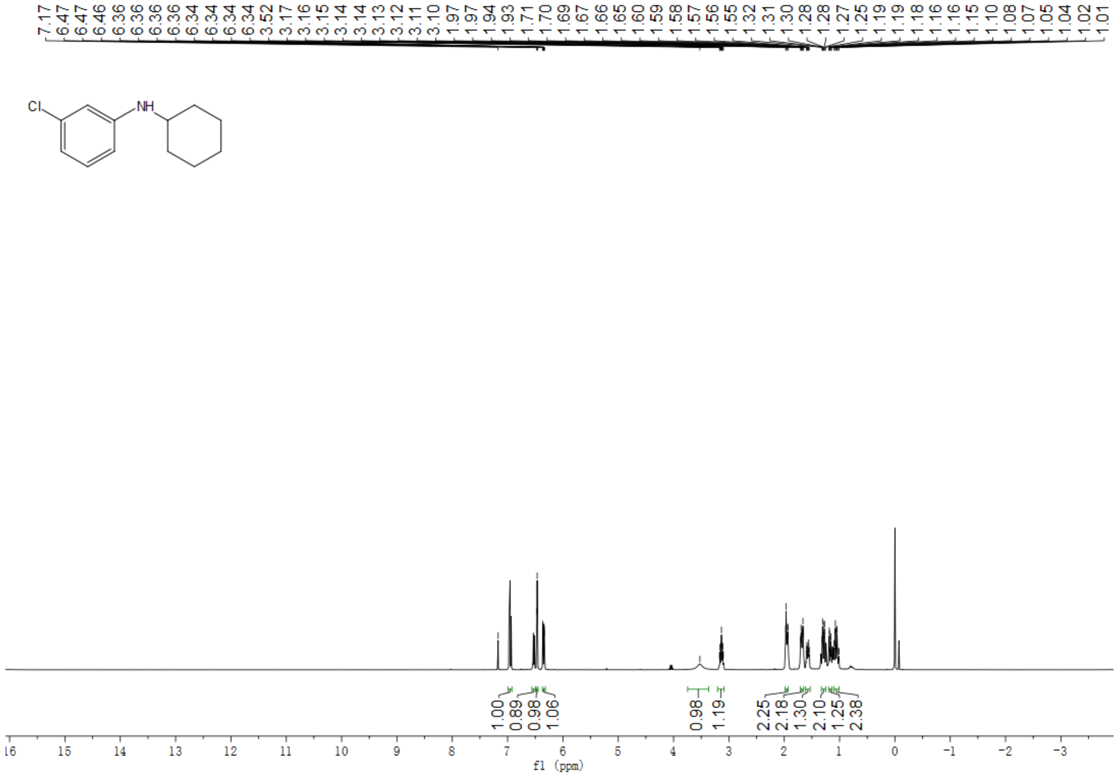

Supplementary Figure 139. ^{1}H NMR (400 MHz, room temperature, $CDCl_3$) spectra of product **61**

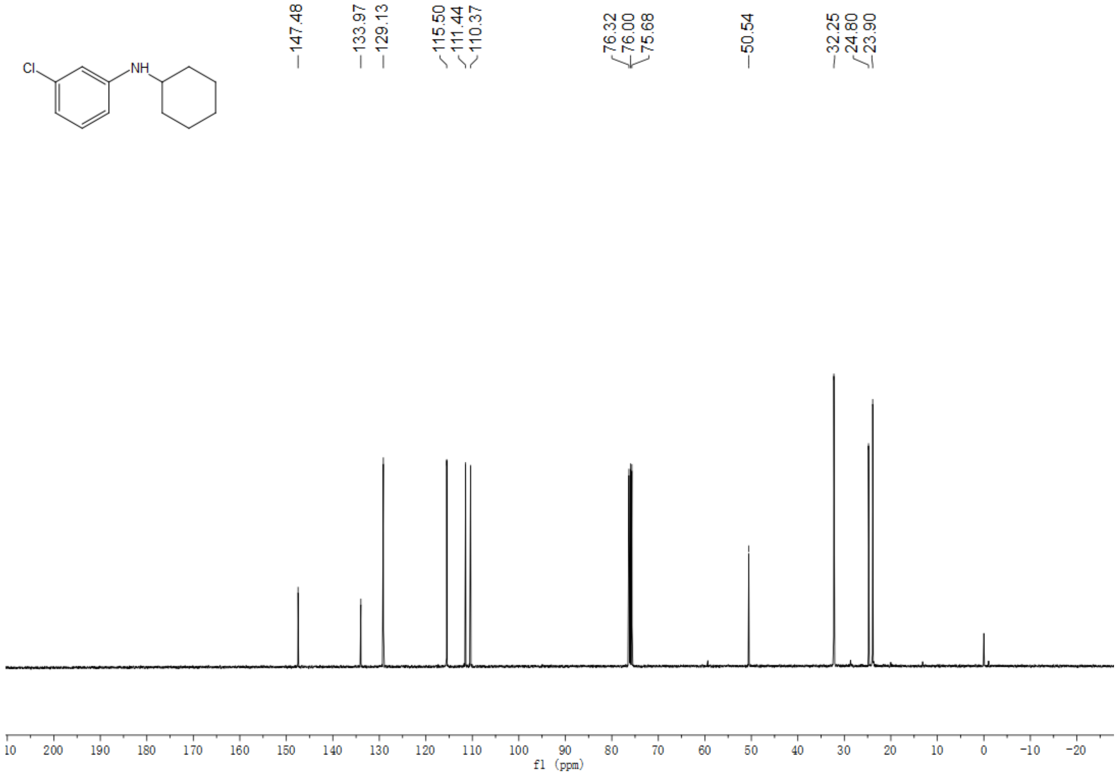

Supplementary Figure 140. ^{13}C NMR (101 MHz, room temperature, $CDCl_3$) spectra of product **61**

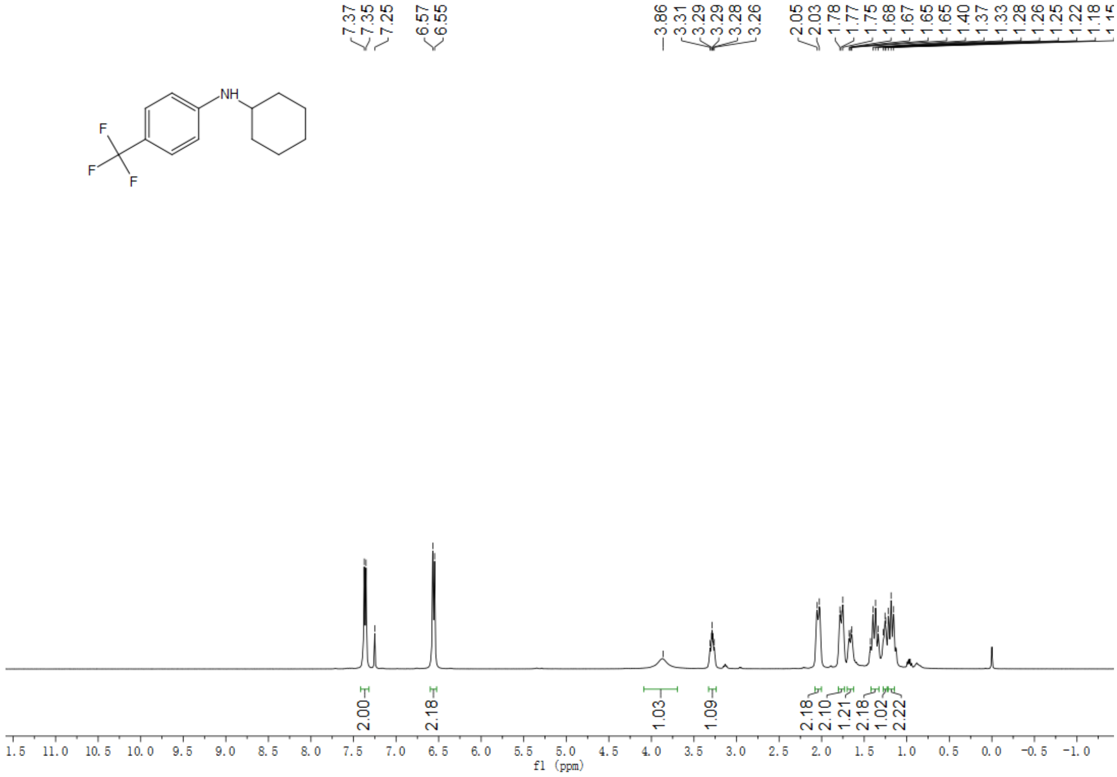

Supplementary Figure 141. ^{1}H NMR (400 MHz, room temperature, $CDCl_3$) spectra of product **62**

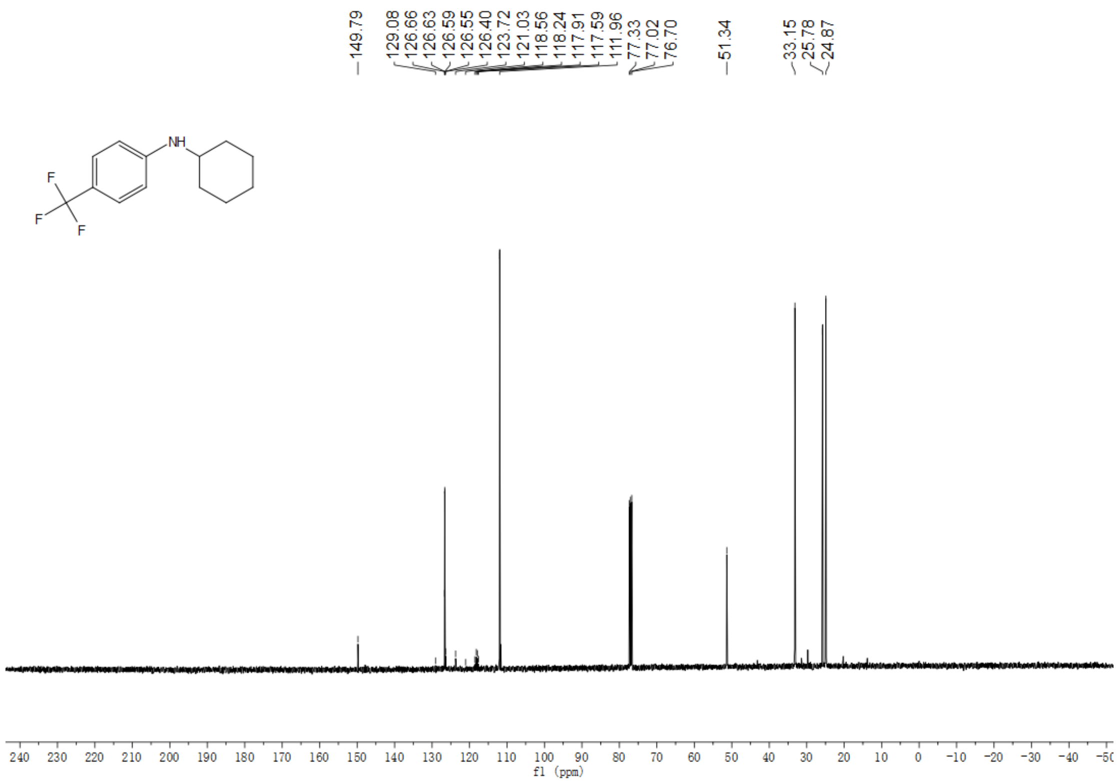

Supplementary Figure 142. ^{13}C NMR (101 MHz, room temperature, $CDCl_3$) spectra of product **62**

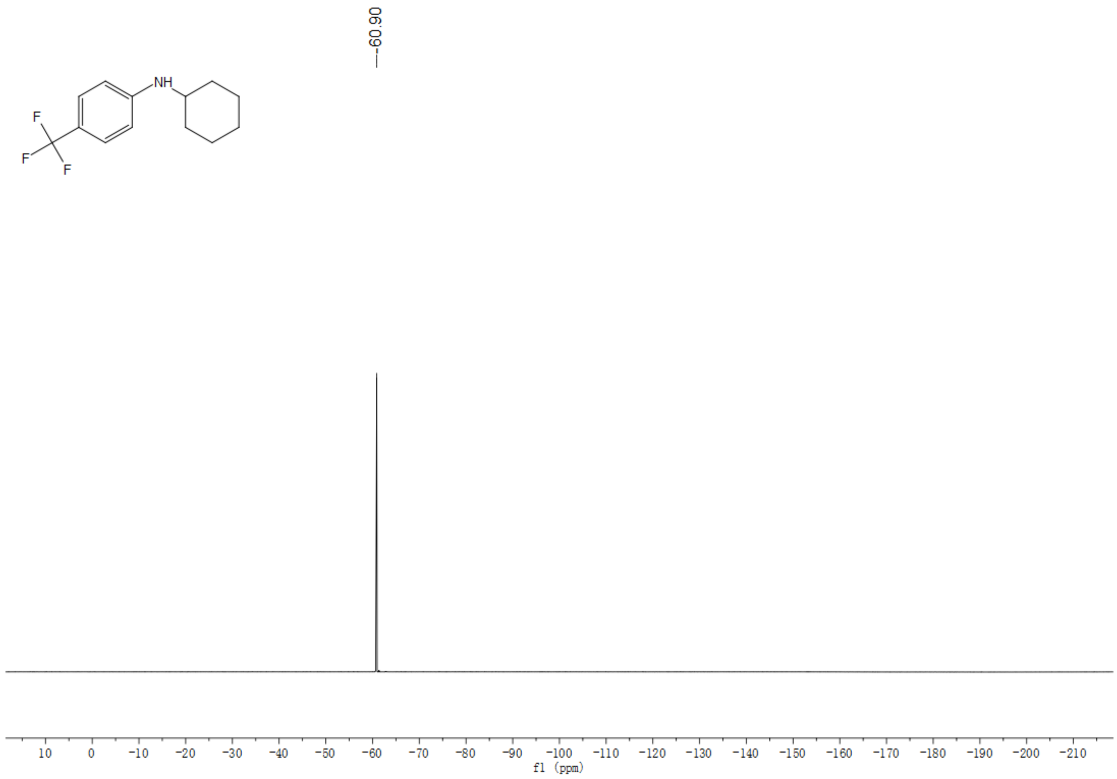

Supplementary Figure 143. ^{19}F NMR (376 MHz, room temperature, $CDCl_3$) spectra of product **62**

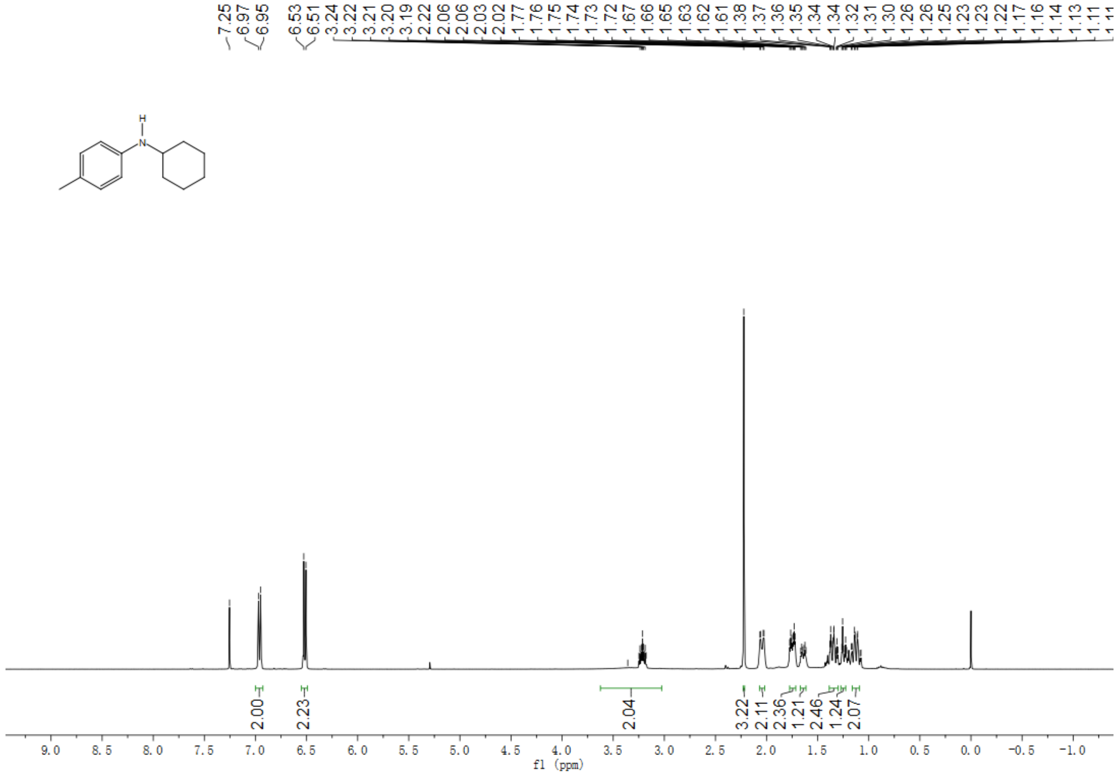

Supplementary Figure 144. ^{1}H NMR (400 MHz, room temperature, $CDCl_3$) spectra of product **63**

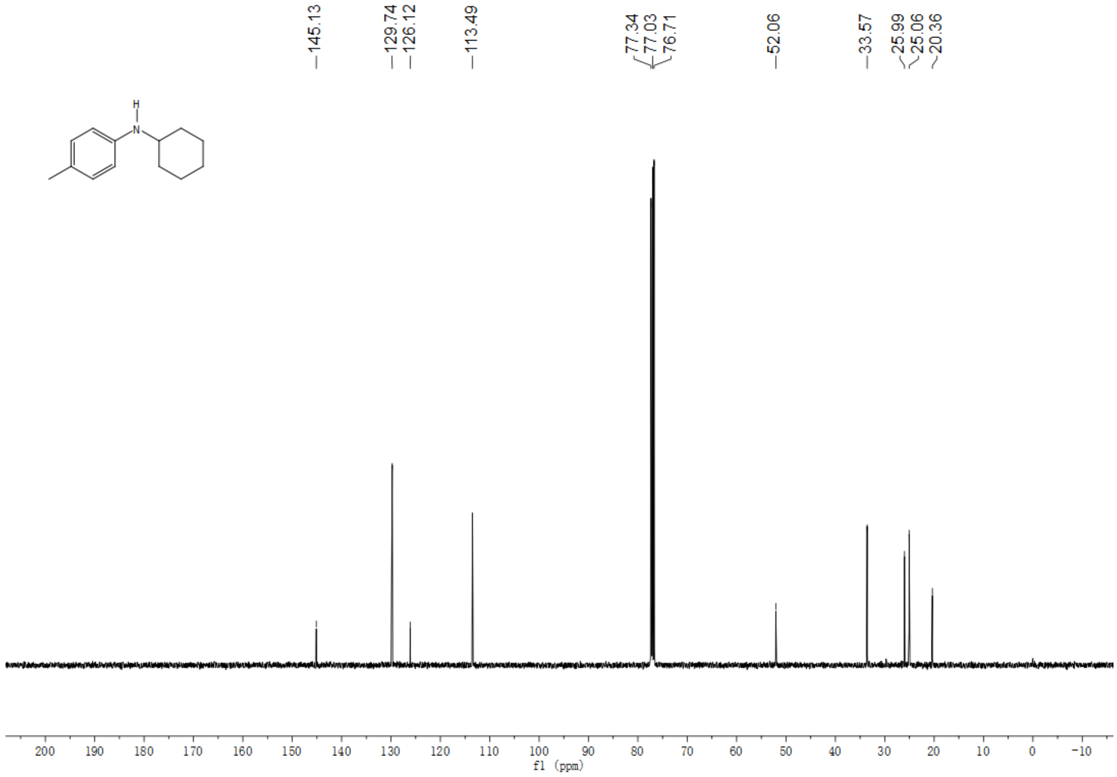

Supplementary Figure 145. ^{13}C NMR (101 MHz, room temperature, $CDCl_3$) spectra of product **63**

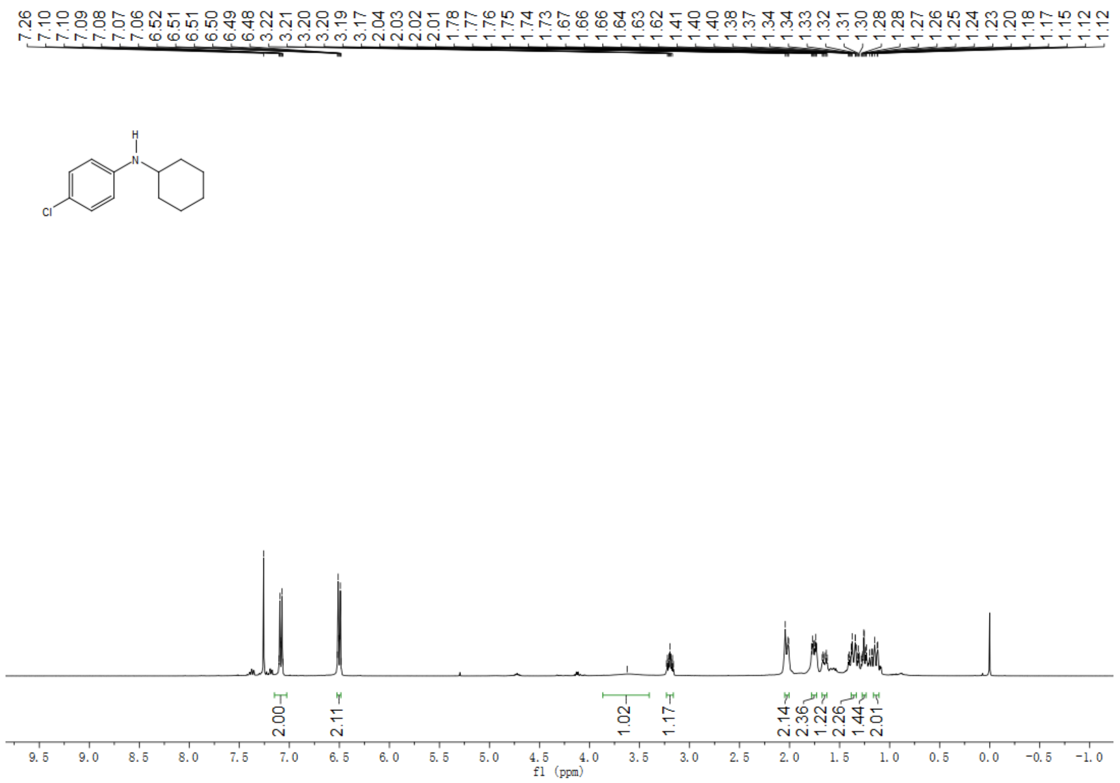

Supplementary Figure 146. ^{1}H NMR (400 MHz, room temperature, $CDCl_3$) spectra of product **64**

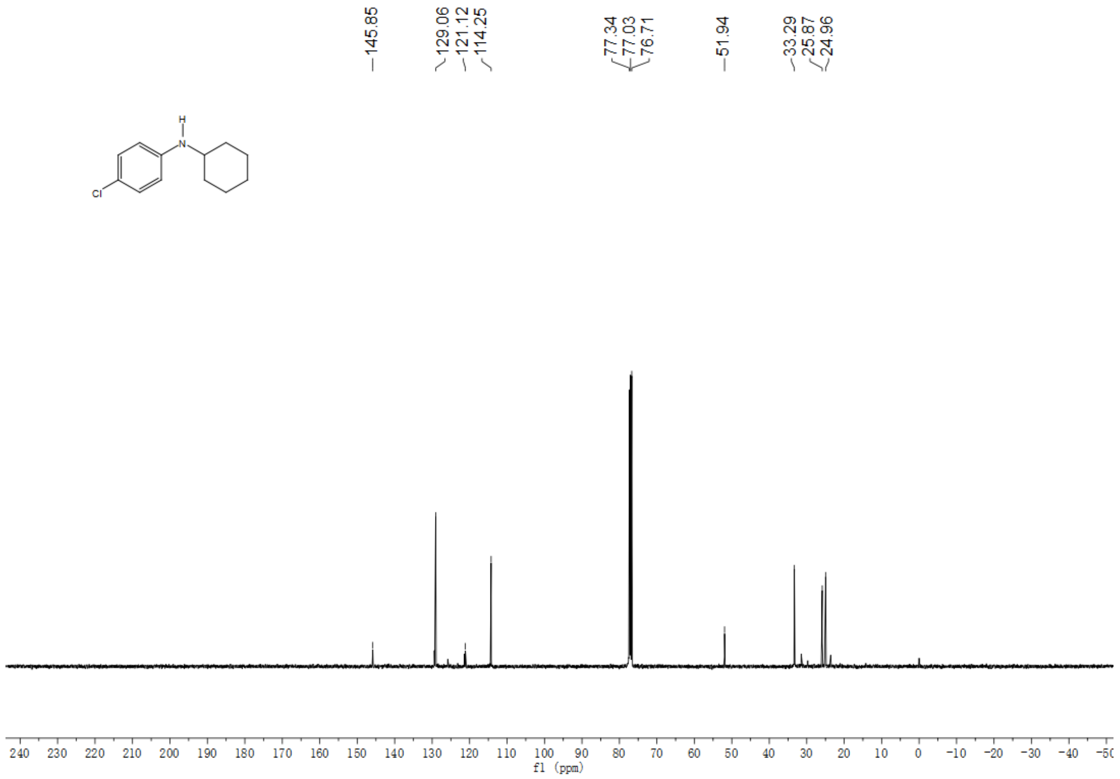

Supplementary Figure 147. ^{13}C NMR (101 MHz, room temperature, $CDCl_3$) spectra of product **64**

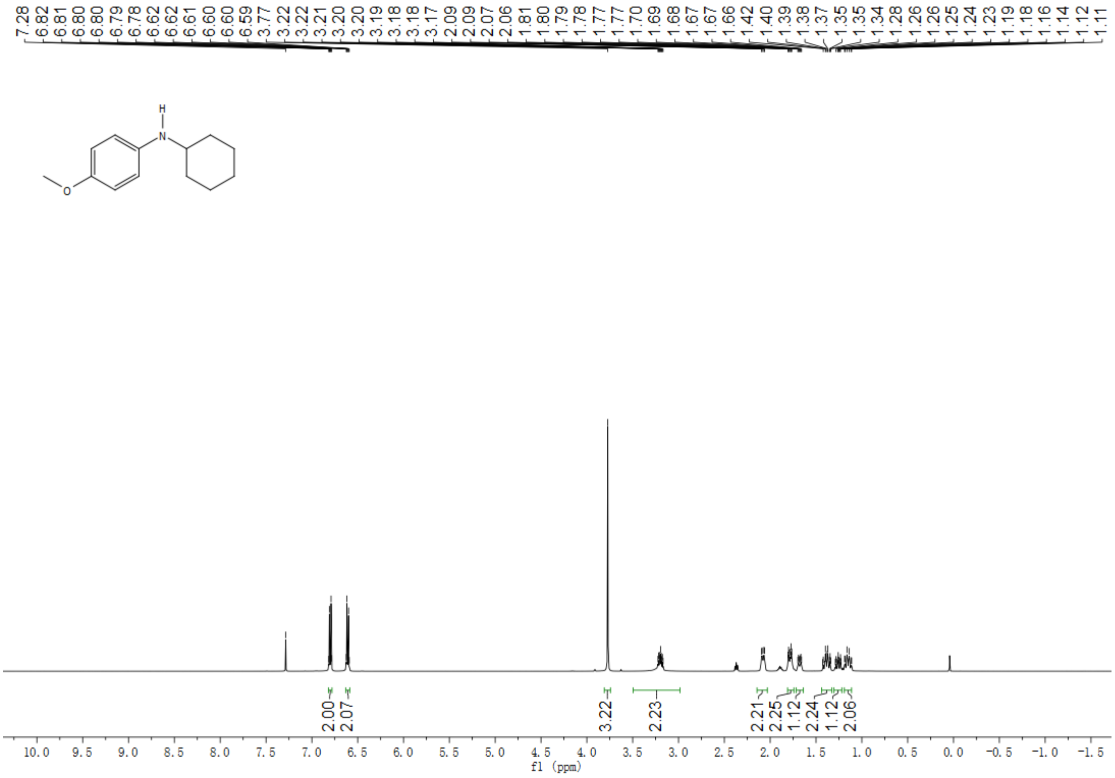

Supplementary Figure 148. ^{1}H NMR (400 MHz, room temperature, $CDCl_3$) spectra of product **65**

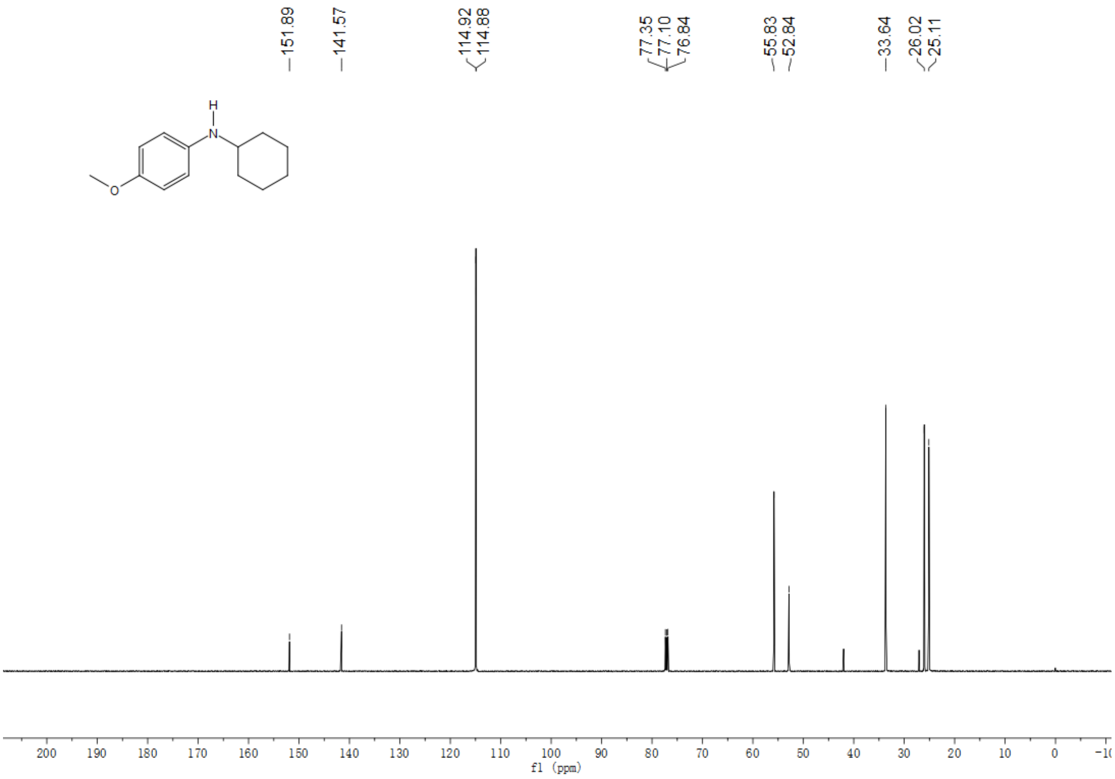

Supplementary Figure 149. ^{13}C NMR (101 MHz, room temperature, $CDCl_3$) spectra of product **65**

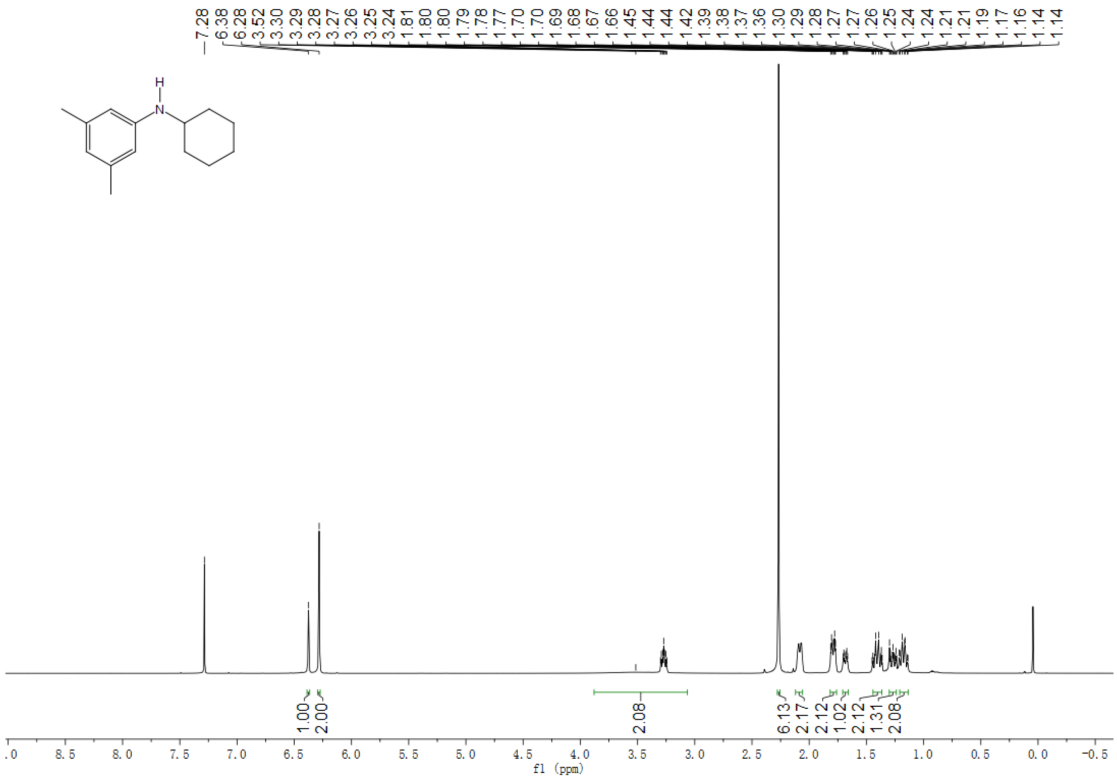

Supplementary Figure 150. ^{1}H NMR (400 MHz, room temperature, $CDCl_3$) spectra of product **66**

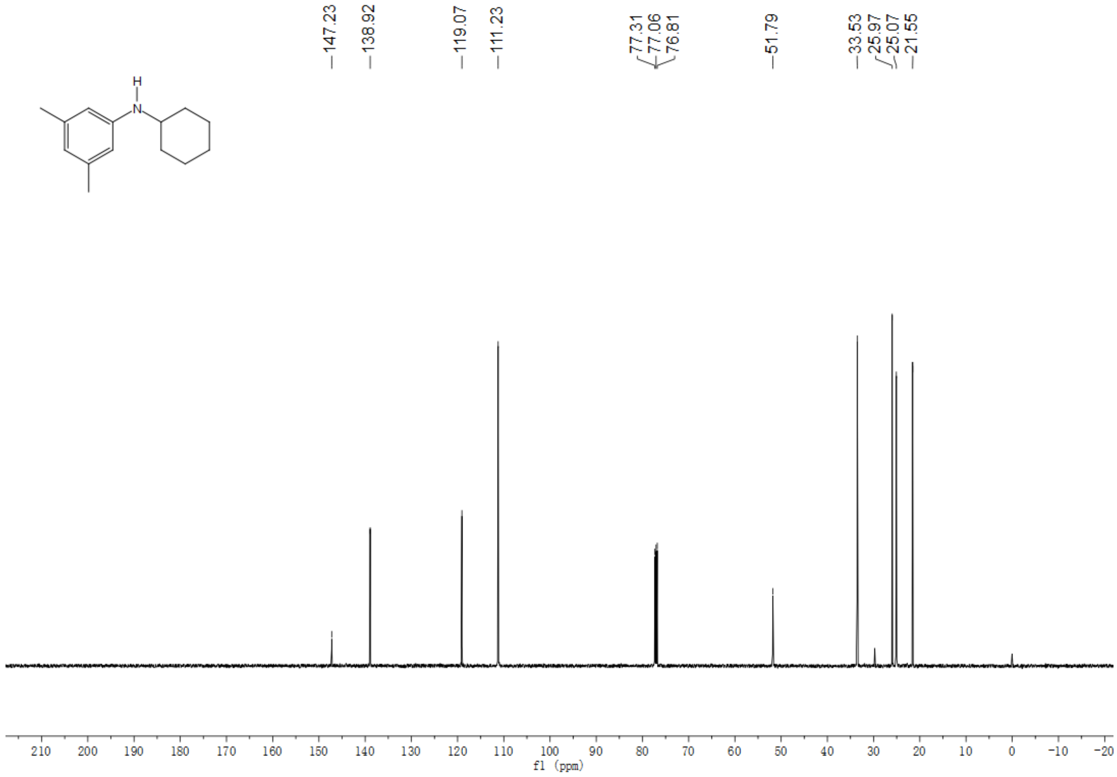

Supplementary Figure 151. ^{13}C NMR (101 MHz, room temperature, $CDCl_3$) spectra of product **66**

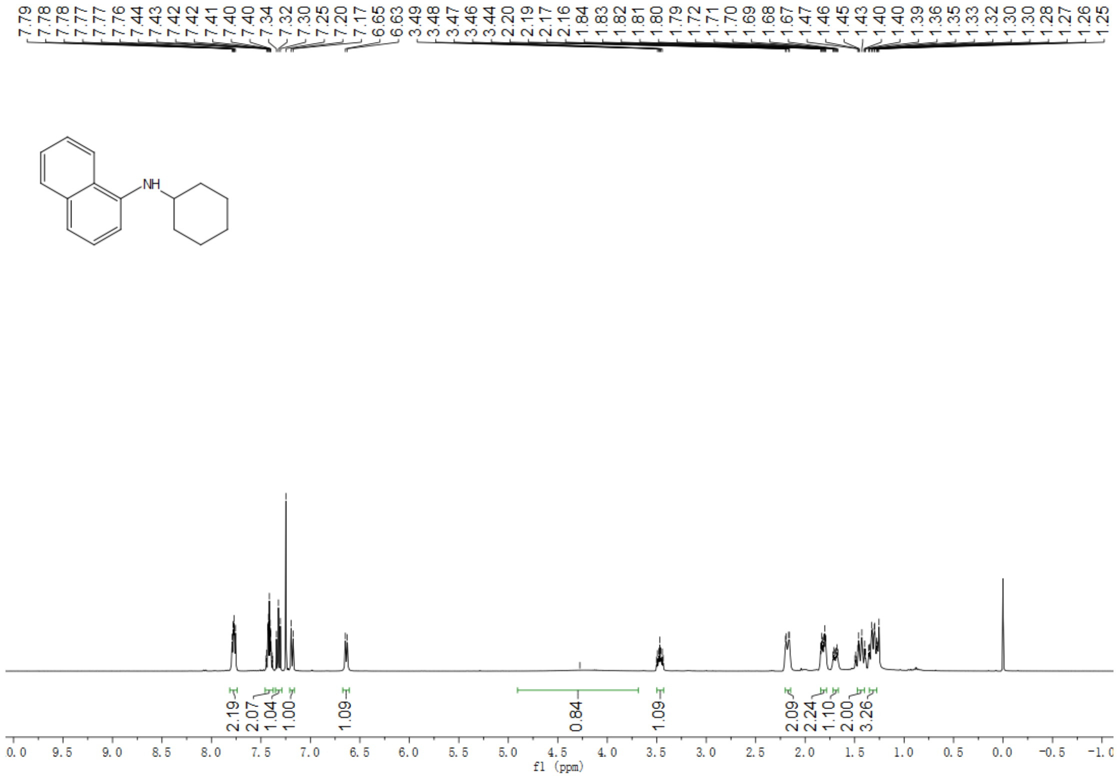

Supplementary Figure 152. ^{1}H NMR (400 MHz, room temperature, $CDCl_3$) spectra of product **67**

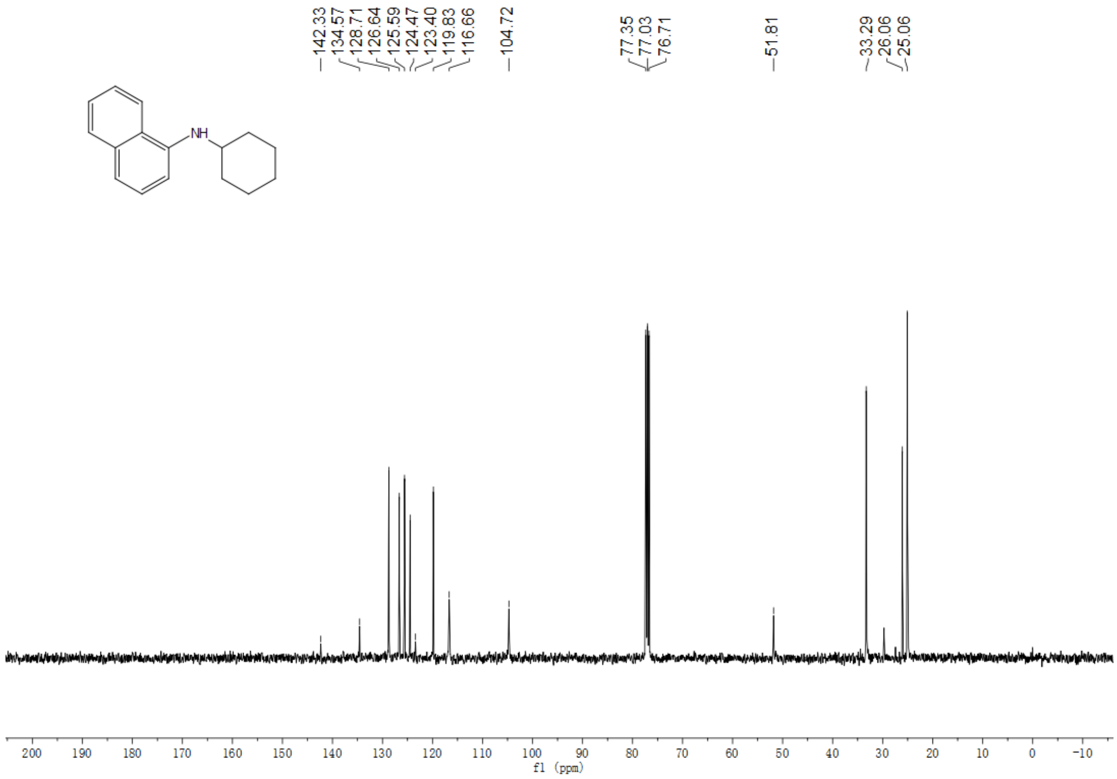

Supplementary Figure 153. ^{13}C NMR (101 MHz, room temperature, $CDCl_3$) spectra of product **67**

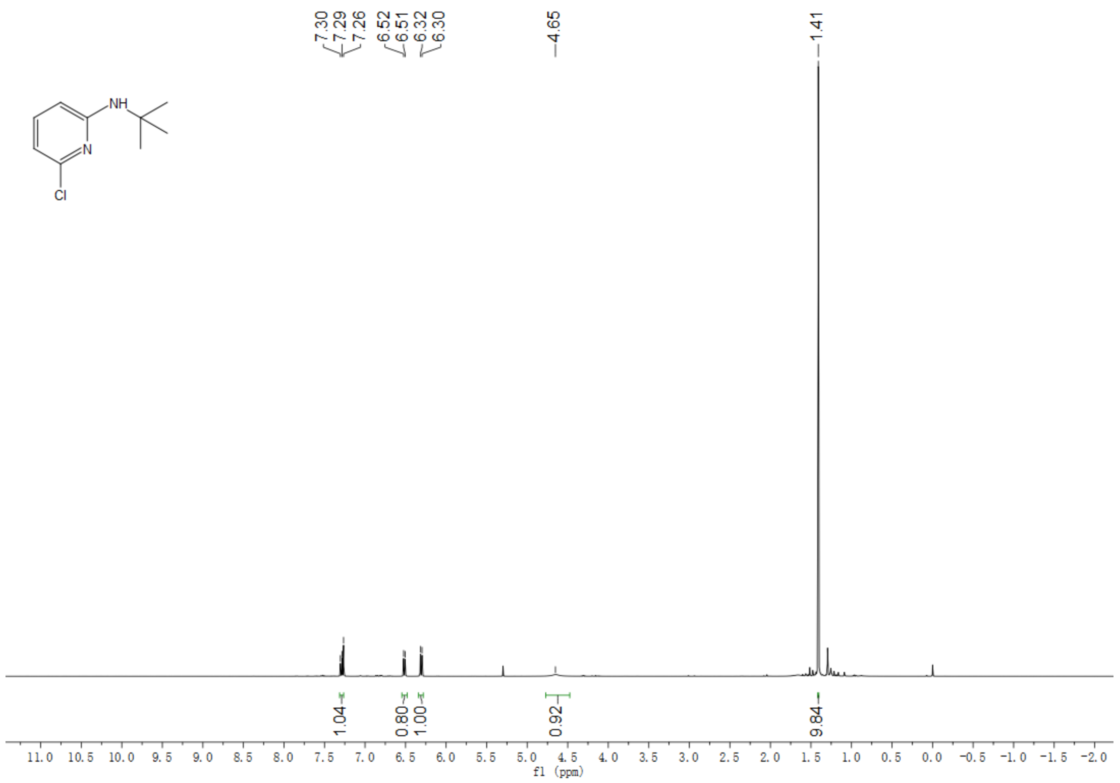

Supplementary Figure 154. ^{1}H NMR (400 MHz, room temperature, $CDCl_3$) spectra of product **68**

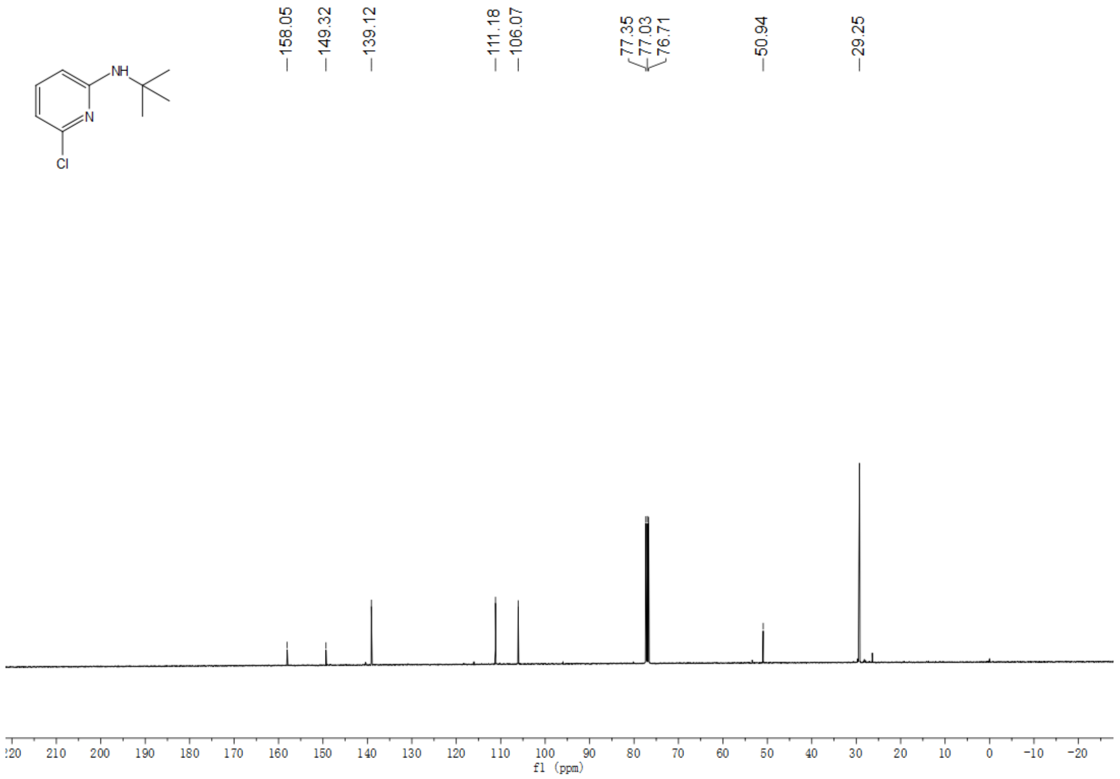

Supplementary Figure 155. ^{13}C NMR (101 MHz, room temperature, $CDCl_3$) spectra of product **68**

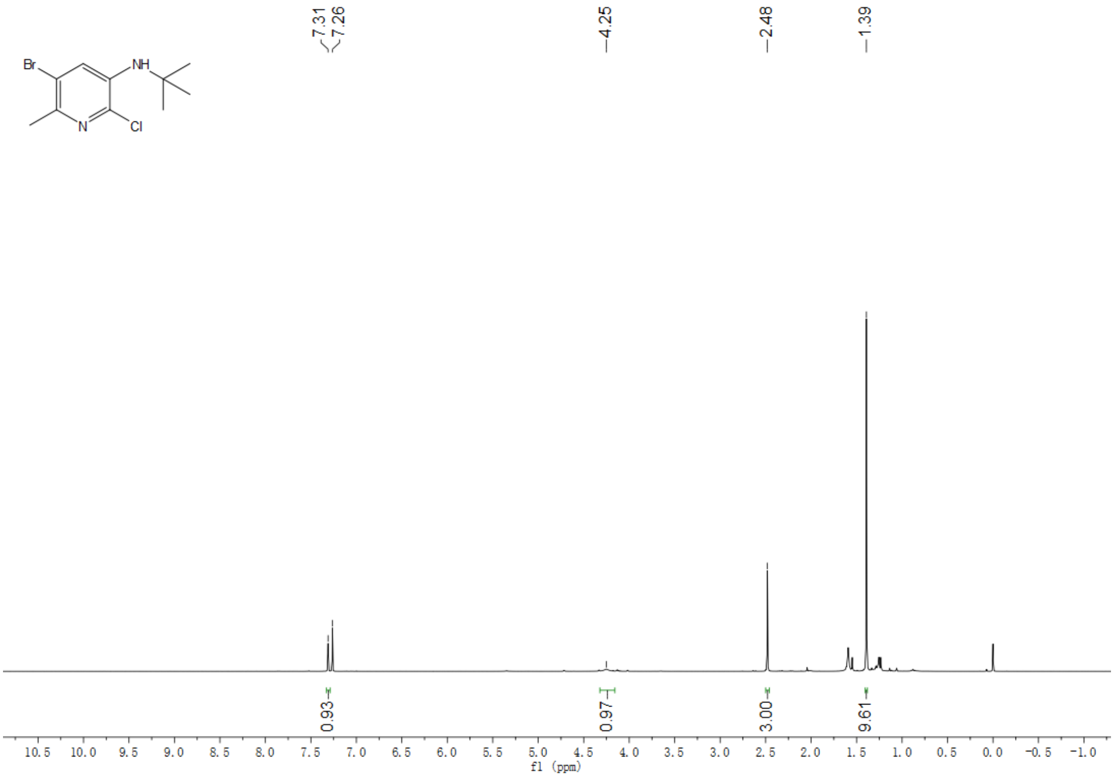

Supplementary Figure 156. ^{1}H NMR (400 MHz, room temperature, $CDCl_3$) spectra of product 6**9**

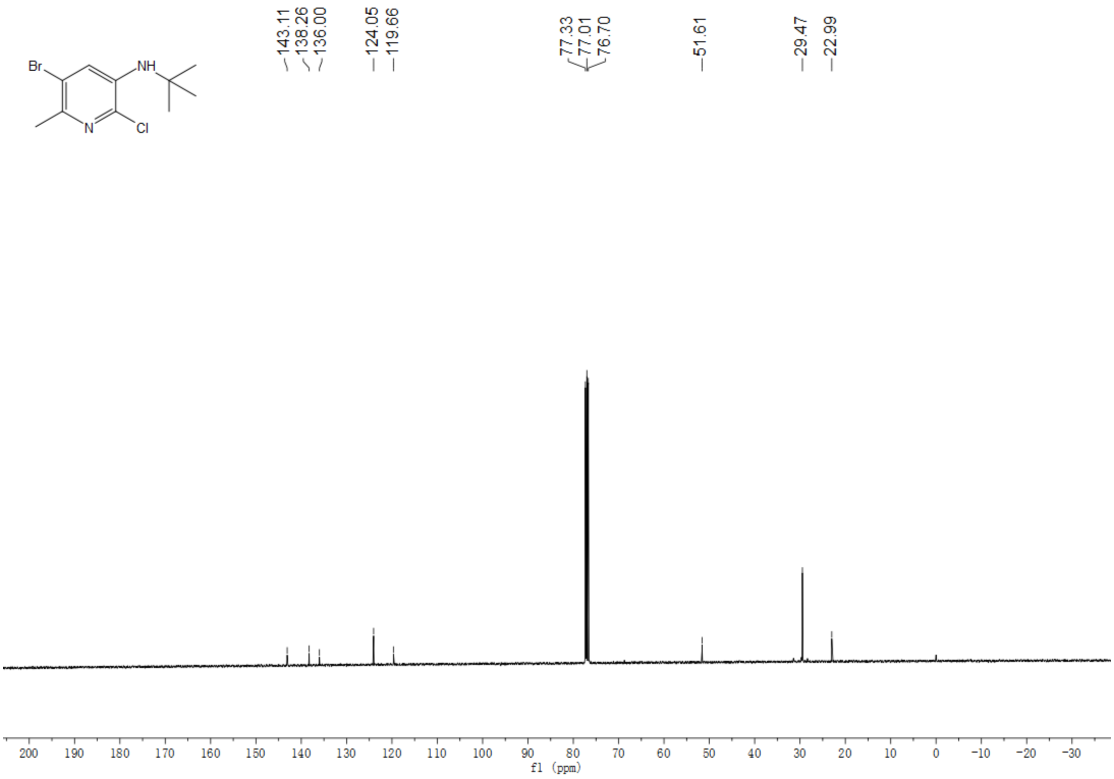

Supplementary Figure 157. ^{13}C NMR (101 MHz, room temperature, $CDCl_3$) spectra of product **69**

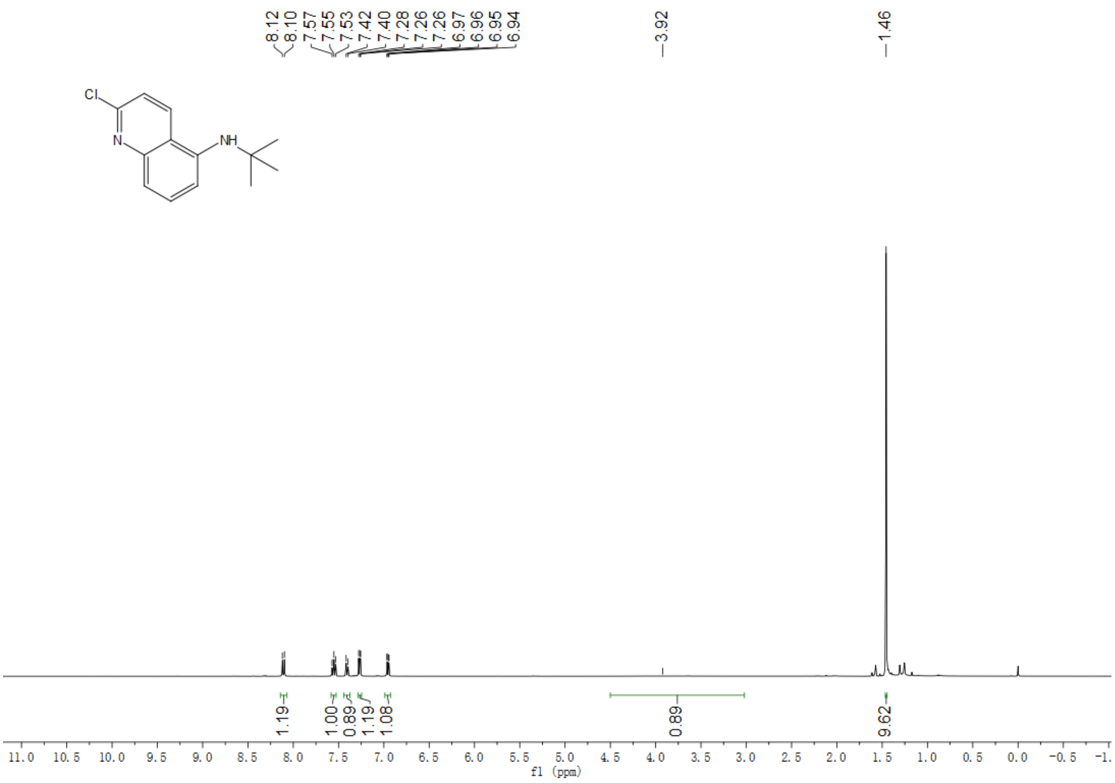

Supplementary Figure 158. ^{1}H NMR (400 MHz, room temperature, $CDCl_3$) spectra of product **70**

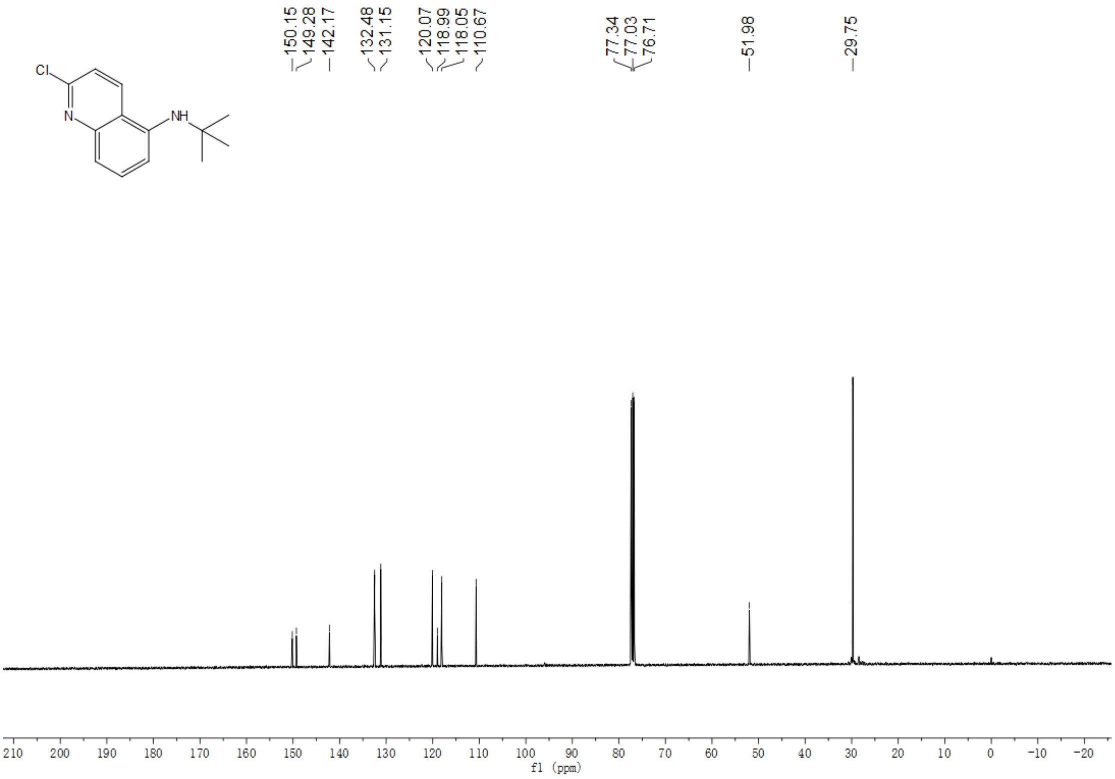

Supplementary Figure 159. ^{13}C NMR (101 MHz, room temperature, $CDCl_3$) spectra of product **70**

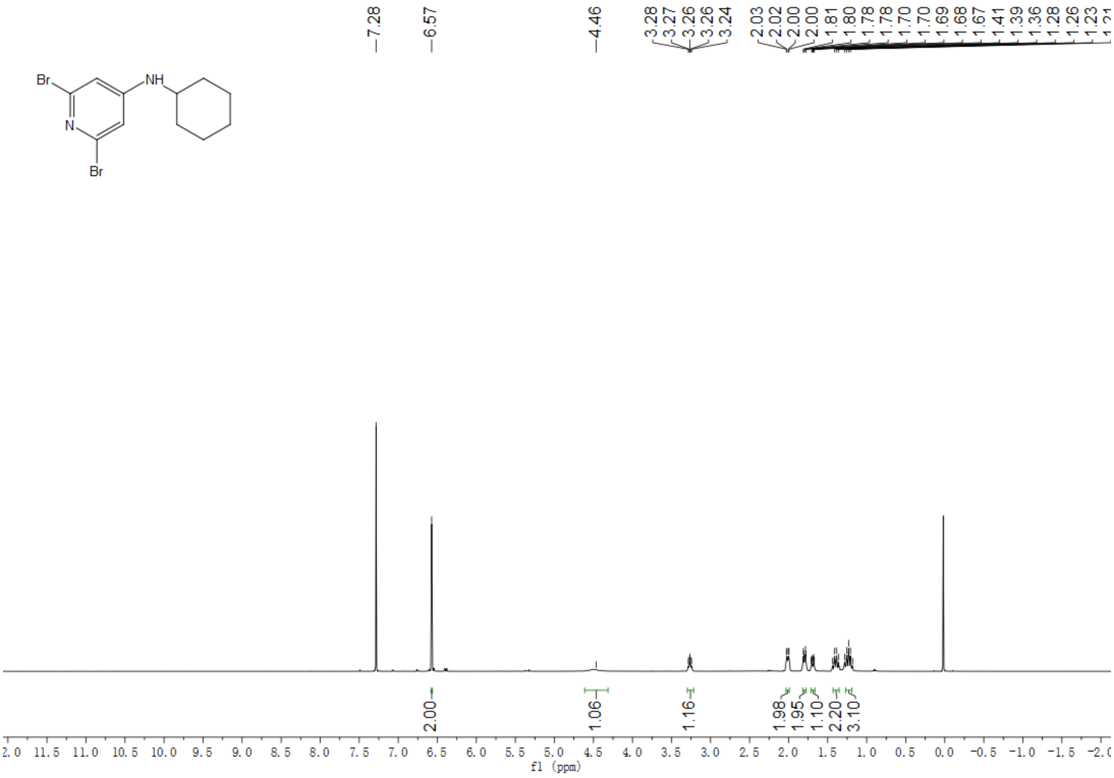

Supplementary Figure 160. ^{1}H NMR (400 MHz, room temperature, $CDCl_3$) spectra of product **71**

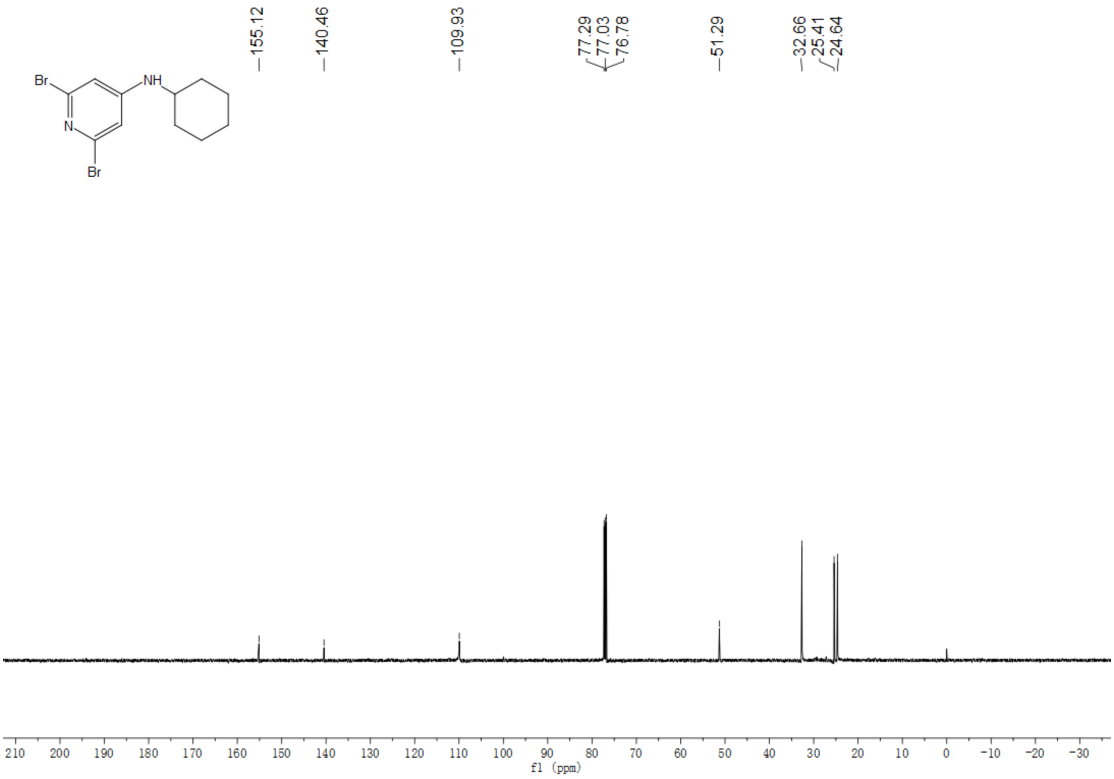

Supplementary Figure 161. ^{13}C NMR (101 MHz, room temperature, $CDCl_3$) spectra of product **71**

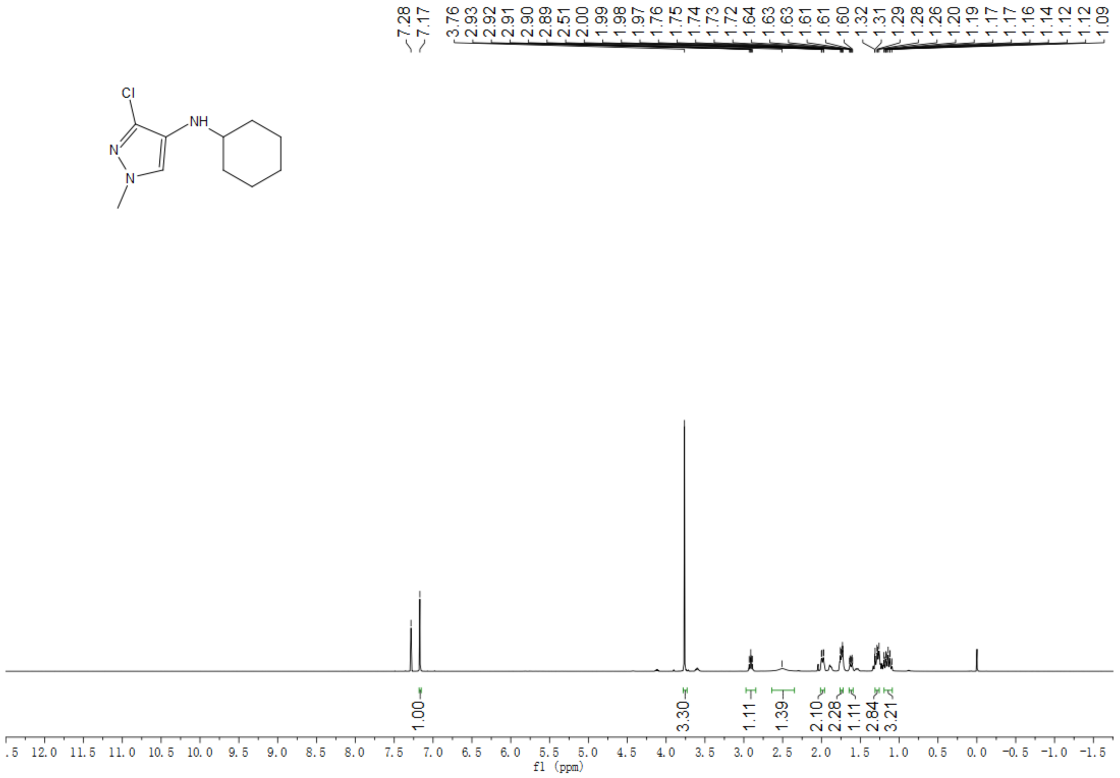

Supplementary Figure 162. ^{1}H NMR (400 MHz, room temperature, $CDCl_3$) spectra of product **72**

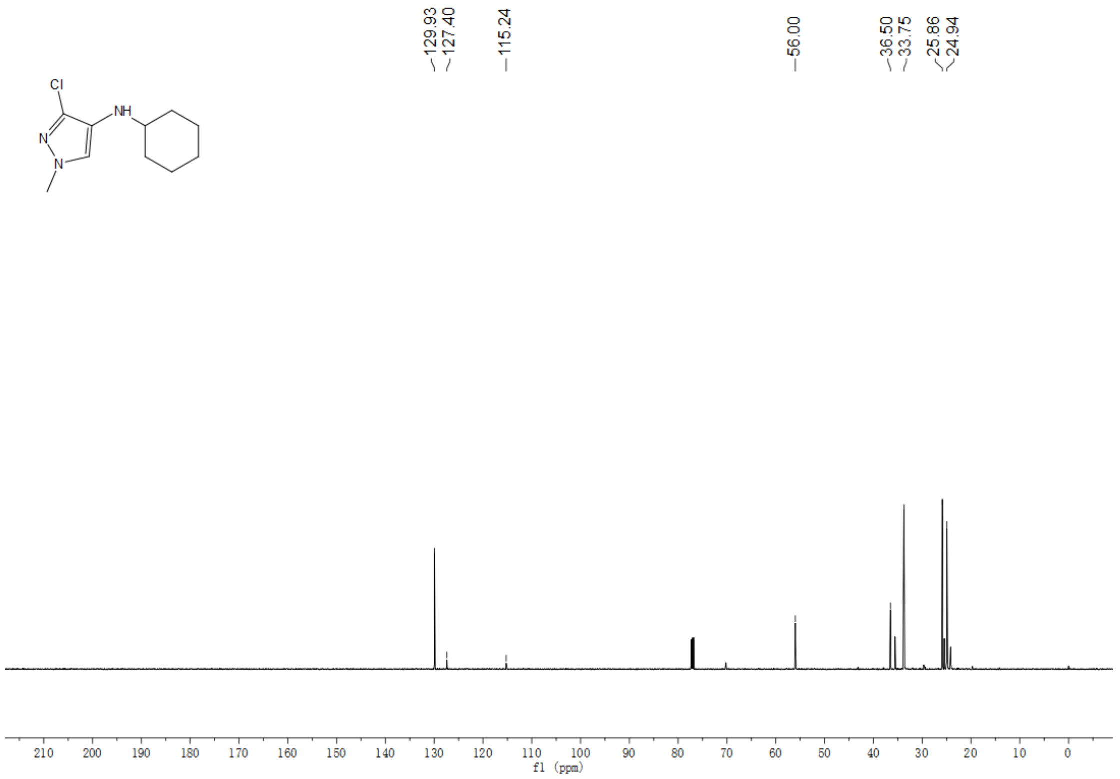

Supplementary Figure 163. ^{13}C NMR (101 MHz, room temperature, $CDCl_3$) spectra of product **72**

Supplementary References

[1] Y. Gao, Z. Wu, L. Yu, Y. Wang, Y. Pan, *Angew. Chem. Int. Ed.* **2020**, *59*, 10859-10863.

[2] V. Dhayalan, C. Sämann, P. Knochel, *Chem. Commun.* **2015**, *51*, 3239-3242.

[3] Y. Wu, Y. Huang, X. Dai, F. Shi, *ChemSusChem* **2019**, *12*, 3185-3191.

[4] V. Hardouin Duparc, G. L. Bano, F. Schaper, *ACS Catal.* **2018**, *8*, 7308-7325.

[5] K. Gao, H. Yorimitsu, A. Osuka, *Eur. J. Org. Chem.* **2015**, *2015*, 2678-2682.

[6] J. W. Cran, D. V. Vidhani, M. E. Krafft, *Synlett* **2014**, *25*, 1550-1554.
